# Oncotransformation in Bhas 42 Cell Transformation Assay by Typical Non-Genotoxic Carcinogens, PFOA and PFOS, and Time-Course Transcriptome Analysis

**DOI:** 10.3390/biom15101431

**Published:** 2025-10-09

**Authors:** Kiyomi Ohmori

**Affiliations:** 1 Chemical Division, Kanagawa Prefectural Institute of Public Health, Chigasaki 2530087, Japan; ohmori.n4yf@pref.kanagawa.lg.jp or ohmori-kiyomi-kz@ynu.ac.jp; Tel./Fax: +81-46-783-4400; 2 Institute of Advanced Science, Yokohama National University, Yokohama 2408501, Japan

**Keywords:** Bhas 42 cell transformation assay, non-genotoxic carcinogen, PFAS, PFOA, PFOS, cell transformation, transcriptome analysis, CAGE analysis

## Abstract

Perfluorinated alkyl substances and polyfluorinated alkyl substances (PFASs) are long-chain compounds, with perfluorooctanoic acid (PFOA) and perfluorooctanesulfonic acid (PFOS) being the most well-known examples. Both are considered typical non-genotoxic carcinogens (NGTxCs). In this study, we verified whether the Bhas 42 cell transformation assay (Bhas 42 CTA) can be used as an effective in vitro method to predict carcinogenicity of NGTxCs using both PFOA and PFOS as typical representatives. Transcriptome analysis during the PFOA-induced transformation process showed that many factors related to the effects of PFOA on the immune system and cancer hallmarks increased or decreased. Thus, we demonstrated that mechanistic analyses such as transcriptome analyses in combination with the transformation focus formation results from the Bhas 42 CTA may be useful tools when assessing the carcinogenicity and other biological effects of NGTxCs such as PFOA. We propose that the Bhas 42 CTA is a simple in vitro test for the detection of NGTxCs, that it has in vitro oncotransformation as an endpoint, and that it can also detect the activation of factors involved in malignant progression, such as invasion and metastasis. It allows for the comprehensive detection of subtle mechanisms in parallel with focus formation throughout the transformation process, from the early stages to malignancy.

## 1. Introduction

Perfluorooctanoic acid (PFOA) and perfluorooctanesulfonic acid (PFOS) are difficult-to-decompose substances that have been used for a long time, and their accumulation and carcinogenicity have become serious problems due to their widespread dispersion in the ecosystem. International Agency for Research on Cancer (IARC) monograph No. 135 [1] reports on the contamination situation with PFOA and PFOS as follows: PFOA and PFOS are ubiquitously distributed across environmental matrices, encompassing atmospheric, aquatic, terrestrial, and dietary compartments. Atmospheric and hydrological transport mechanisms facilitate the translocation of PFOA and PFOS, resulting in their accumulation in marine environments, terrestrial soils, and groundwater. Dietary exposure to PFOA and PFOS arises from atmospheric deposition and bioaccumulation via contaminated water and soil, with additional contributions from agricultural application of biosolids as fertilizer. Animal-derived food products acquire PFOA and PFOS contamination through multiple exposure pathways, including ingestion of contaminated water, feed, soil and air. Elevated concentrations of PFOA and PFOS have been quantified in aquatic organisms and avian products, particularly fish, seafood, and eggs, with levels ranging from 10^4^ to 10^5^ pg/g. Occupational cohorts experience elevated exposure levels to PFOA and PFOS, primarily via inhalation pathways. Additional exposure may occur through dermal absorption and ingestion of contaminated particulate matter. Biomonitoring data indicate exposure in diverse occupational settings, with the highest levels in primary manufacturing (up to median values of thousands of nanograms per milliliter of serum), and in one example a fluorinated chemicals manufacturing worker had a PFOA concentration of serum measured at 32,000 ng/mL.

The IARC reclassified PFOA from Group 2 (possibly carcinogenic to humans) to Group 1 (carcinogenic to humans) and newly classified PFOS into Group 2B in 2023 [1,2]. The Group 1 classification of PFOA was based on sufficient evidence of carcinogenicity in experimental animals and strong mechanistic evidence indicating epigenetic alterations and immunosuppression in exposed humans. For PFOA, there was “limited” evidence for renal cell carcinoma and testicular cancer in humans. PFOA and PFOS induce epigenetic alterations and are immunosuppressive in exposed humans. Epidemiological studies in diverse human cohorts, including children and adults, have shown that exposure to PFOA is associated with increased risk of infectious diseases and attenuated immunogenic responses to a wide range of vaccine antigens. These observations are further substantiated by evidence demonstrating suppressed cytokine secretion, reduced lymphoproliferative capacity in human primary immune cells, and dysregulated humoral responses to T-cell-dependent antigens and leukocyte in rodents. PFOA has been shown to induce oxidative stress and modulate signaling pathways mediated by nuclear receptors, including peroxisome proliferator-activated receptors (PPARα and PPARγ) and the homeostatic androstane/pregnane X receptor (CAR/PXR), altering cell proliferation, cell death, and nutrient and energy supply in human primary cells and various experimental platforms [1,2]. PFOS is possibly carcinogenic to humans (Group 2B). The Group 2B evaluation for PFOS was based on strong mechanistic evidence across test systems, including in exposed humans. However, PFOS is classified into Group 2B because the evidence for carcinogenicity is inadequate in humans and limited in experimental animals. Although there have been reports of oxidative stress effects due to PFOA and PFOS, no evidence has been found to support a direct genotoxic effect for either substance. Therefore, both PFOA and PFOS are non-genotoxic but are considered carcinogenic or suspected carcinogens, i.e., non-genotoxic carcinogens (NGTxCs) [3].

To address regulatory gaps regarding the identification of NGTxCs, Jacobs and other members of an expert group reported on the establishment of a policy by the Organization of Economic Cooperation and Development (OECD) regarding an integrated approach to the testing and assessment of NGTxCs (NGTxC IATA) [4].

While carcinogenicity testing requirements differ across sectors and jurisdictions, the standard protocol generally begins with a series of genotoxicity assays, including tests for mutagenicity. In the event of positive findings in in vivo genotoxicity testing, a long-term rodent bioassay for carcinogenicity may be required. However, this is rarely the case under most chemical regulations, except for those pertaining to plant protection, biocides, and pharmaceuticals. The decision to conduct further testing based on genotoxicity test outcomes creates a regulatory gap in identifying NGTxCs. To address this gap, the OECD established an expert group in 2016 to develop an NGTxC IATA. It focused on the cell transformation assay (CTA) in the context of the NGTxC IATA.

According to a review by Colacci et al. [5], despite nearly six decades since the first in vitro model for assessing chemical carcinogenesis was introduced, the Cell Transformation Assay (CTA) remains the sole method providing a measurable endpoint for oncotransformation. Recent advances in omics technologies have begun to elucidate the underlying mechanisms, revealing a multistep transformation process consistent with human carcinogenesis. These insights identify key events that align with the established stages of tumor development in humans, which is a long and complex process characterized by the disruption of multiple biological systems responsible for defense, repair, and homeostatic regulation. The CTA can capture several key events that lead to the disruption of specific biological characteristics, known as the hallmarks of cancer, as well as the step that marks commitment, namely, the point of no return leading to malignant transformation. Collectively, the CTA is ready to be included in the NGTxC IATA, allowing us to address the key data gap identified for the NGTxC IATA.

As reviewed by Louekari et al. [6], current regulatory tools for identifying and managing non-genotoxic carcinogens (NGTxCs) remain inadequate, posing a significant burden on public health. Furthermore, no internationally agreed-upon specific test guidelines (TGs) exist to address this toxicity endpoint or the mechanisms of carcinogenesis. Therefore, there is a critical need to develop validated TGs and testing strategies informed by contemporary insights into carcinogenic mechanisms.

The Bhas 42 CTA is a test method for detecting NGTxCs. The promotion test was developed by Ohmori et al. and reported in 2004 [7]. Furthermore, a collaborative study of the promotion test for Bhas 42 CTA was conducted among 14 laboratories effort resulted in a highly robust framework for the protocol [8]. An initiation test was subsequently added and then approved as OECD GD231 in 2016 [9]. There is ongoing demand for an international testing strategy for detecting NGTxCs.

Therefore, we evaluated whether PFOA and PFOS, typical NGTxCs for which international contamination and spread are a problem, can be detected using the Bhas 42 CTA. In addition, for PFOA, which is in IARC Group 1 and for which carcinogenicity in humans has been confirmed, we analyzed the mechanism of the cell transformation process via time-course transcriptome analyses during the transformation process using the Bhas 42 CTA. This mechanism is compared with the mechanism of human carcinogenesis.

Based on these findings, the Bhas 42 CTA has in vitro oncotransformation as an endpoint and has demonstrated its usefulness as a test method capable of comprehensively detecting subtle mechanisms in parallel with focus formation throughout the entire transformation process from the early stage to malignant progression.

## 2. Materials and Methods

### 2.1. Cell Culture

Bhas 42 cells were obtained from the Japanese Collection of Research Bioresources (JCRB) Cell Bank. The stock cells used were the second passage in our laboratory. Minimum Essential Medium (MEM) was obtained from Nissui Pharmaceutical Co. (Tokyo, Japan). Dulbecco’s Modified Eagle Medium/Nutrient Mixture F-12 (DMEM/F12) was purchased from GIBCO Laboratories (Grand Island, NY, USA). Fetal bovine serum (FBS) was obtained from Morgate Bio Tech (Lot No. 8301104, Bulimba, Australia). Bhas 42 cells were cultured in MEM supplemented with 10% FBS at 37 °C in a humidified atmosphere of 95% air and 5% CO_2_. For subculturing, cells were detached using 0.25% trypsin (Wako Pure Chemical Industries, Osaka, Japan) and passaged to maintain approximately 70% confluence. Expanded cells were aliquoted and stored at −80 °C. Each experiment was conducted using a fresh aliquot of these stock cells. Plastic culture dishes and plates were obtained from Sumitomo Bakelite (Tokyo, Japan).

### 2.2. CTA Using Bhas 42 Cells in the Stationary Phase (Bhas 42 CTA Promotion Test)

The Bhas 42 CTA promotion test of OECD GD231 [9] was performed with a minor modification in that the mother culture medium was the same as that used from day 0 onwards. The frozen working stock cells were rapidly thawed and suspended in DMEM/F12 medium supplemented with 5% FBS (DF5F), then cultured in 90 mm dishes with 10 mL medium. Upon reaching ~70% confluence, cells were trypsinized and seeded in DF5F at 10,000 cells/mL in 90 mm dishes (day 3). When these cultures again reached ~70% confluence, a suspension of 7 × 10^3^ cells/mL in DF5F was prepared from the mother culture, and 2 mL was plated into each well (1.4 × 10^4^ cells/well) of six-well plates (day 0).

To assay the test chemicals, each dose group consisted of six wells. After the cells were cultivated for 4 days, the medium was replaced with fresh DF5F medium containing six concentration test chemicals (PFOA or PFOS), solvent control (dimethyl sulfoxide (DMSO) was purchased from Sigma-Aldrich in Merck, St. Louis, MO, USA), or positive control (50 ng/mL of 12-O-tetradecanoyl phorbol 13-acetate (TPA) at the final concentration). PFOA (CAS 335-67-1, >98.0% purity, A5720) was purchased from Tokyo Kasei Kogyo Inc., Tokyo, Japan. PFOS (PFOS-K, CAS 2795-39-3, >98.0% purity, 77282) was purchased from Sigma-Aldrich in Merck. TPA (CAS 16561-29-8, >99%, P8139) was purchased from Sigma-Aldrich in Merck. The final DMSO concentration in the medium of the group treated with the test substance and the control was 0.1%. The medium was replaced with fresh medium containing a test chemical or 0.1% DMSO on days 4, 7, and 11. Then, fresh DF5F medium without a test chemical or DMSO was replaced on day 14. On day 21, the cells were fixed with methanol for 30 min and stained with 5% Giemsa solution (for microscopy, 102904, Sigma-Aldrich in Merck) for 1 h. Transformed foci were characterized using the following morphological criteria: deep basophilic staining, the dense multi-layering of cells, the random orientation of cells at the edge of the foci, and more than 100 cells within a focus.

### 2.3. Statistical Analysis and Criteria of Judgment

As described in the OECD Guidance Document on the in vitro Bhas 42 CTA (Series on Testing & Assessment No. 231) [9], the quantifiable unit in the six-well format was the number of transformed foci per well. The test-chemical-induced transformation frequency was statistically analyzed via multiple comparisons using the one-sided Dunnett test (*p* < 0.05, upper-sided). If statistical significance was obtained at only one concentration, dose dependency was analyzed using the Jonckheere test (*p* < 0.05, upper-sided) for the six-well format. A chemical that satisfied both criteria was judged as positive. The chemical that met only the first criterion was considered equivocal. A chemical that did not induce a statistically significant increase in transformed foci at any concentration was deemed negative. The following procedure was used to evaluate the transformation results of the positive control for TPA; the statistical significance was evaluated using a one-sided Student *t*-test (*p* < 0.05, upper-sided) depending on the results of the F-test for homoscedasticity.

### 2.4. Isolation of Total RNA

To support the Cap Analysis of Gene Expression (CAGE) analysis, three biological replicates from the independent thawing of stock cells were prepared for each group of the test substance (PFOA) or solvent control (DMSO) treatments. Total RNA was subjected to CAGE analysis as follows. Bhas 42 cells treated with PFOA or DMSO were washed three times with 2 mL PBS (−) per well. Cells were lysed using ISOGEN (Nippon Gene, Tokyo, Japan), and total RNA was extracted and purified according to the protocol for the product. Further purification was performed using the RNeasy Mini Kit (Qiagen, Tokyo, Japan) following the QIAGEN Supplementary Protocol for the purification of cytoplasmic RNA from animal cells using the RNeasy^®^ Mini Kit for the product.

The absorbance of the extracted and purified RNA samples was measured using Nanodrop, and RNA samples with values of 1.8 or higher at 260 nm/280 nm and 260 nm/230 nm were used for the CAGE analysis.

### 2.5. CAGE Analysis

Native Elongating Transcript Cap Analysis of Gene Expression (NET-CAGE) library preparation, sequencing, read mapping, gene expression profiling, and GO enrichment analyses were conducted by DNAFORM (Yokohama, Japan). RNA quality was verified using a Bioanalyzer (Agilent Technologies, Santa Clara, CA, USA), ensuring an RNA integrity number (RIN) > 7.0. cDNA synthesis was performed from total RNA using random primers. The ribose diols at the 5’cap structures were oxidized and subsequently biotinylated. The biotinylated RNA/cDNAs were selected using streptavidin beads (cap-trapping). After RNA digestion by RNaseONE/H and adaptor ligation to both ends of cDNA, double-stranded cDNA libraries (CAGE libraries) were constructed. CAGE libraries were sequenced using single-end reads of 75 nt on a NextSeq 500 instrument (Illumina, San Diego, CA, USA). The obtained reads (CAGE tags) were mapped to the mouse GRCm39 genome using STAR (version 2.7.9a).

CAGE tag clustering and the detection of differentially expressed genes were performed using the pipeline RECLU [10]. Tag count data were clustered using the modified Paraclu algorithm, and clusters with counts per million <0.1 were excluded. Regions with ≥90% overlap between replicates were extracted using BEDtools (v2.29.2). Clusters with an irreproducible discovery rate ≥ 0.1 or length > 200 bp were removed. Differentially expressed genes were detected using the edgeR package. (version 3.14.0). The list of differentially expressed genes detected using RECLU with a False Discovery Rate (FDR) of ≤0.05 was used for GO enrichment analysis while using the clusterProfiler package [11] (version 4.4.4).

### 2.6. Venn Diagram Analysis

In the CAGE analysis, data for upregulated and downregulated genes were extracted at every five points with an FDR of less than 0.05 (FDR < 0.05) for the PFOA-treated and solvent (DMSO)-treated groups. The overlap in upregulated or downregulated genes between treatment time groups was analyzed using the “Calculate and draw custom Venn diagrams” feature on the Bioinformatics & Evolutionary Genomics website (https://bioinformatics.psb.ugent.be/webtools/Venn/, accessed on 17 January 2024).

### 2.7. GO Analysis

According to the CAGE analysis results at each PFOA treatment time, GO analysis was performed on genes whose gene expression significantly changed (FDR < 0.05) in the PFOA treatment group compared with the solvent control group.

Comparisons between the two groups were performed using the edgeR software (version 3.22.5), genes with variable expression were detected, and significant differences were tested. The analysis only includes genes registered in the GO consortium database (http://geneontology.org/).

Data for upregulated or downregulated genes were extracted at every RNA sampling point with FDR < 0.05 between the PFOA-treated and solvent-treated groups. The extracted genes were functionally classified according to the BPs in GO with the “Cluster Profiler Version 4.4.4” software and “OrgDb 3.15” database.

The statistically overrepresented GO terms in each group of differentially expressed genes were extracted using a Benjamini–Hochberg FDR-corrected *p*-value of <0.01 (Tables S1 and S2).

To recognize the hierarchical structure of the extracted GO terms, we extracted GO terms with an FDR-corrected *p*-value of <0.01 and used QuickGO (https://www.ebi.ac.uk/QuickGO/, accessed on 14 August 2024), an online analysis tool. A Benjamini–Hochberg FDR-corrected *p*-value of <0.01 indicated a significantly enriched GO term. To recognize the hierarchical structure of the selected GO terms, we used QuickGO, an online analysis tool.

### 2.8. Functional Analysis and Pathway Analysis

According to the CAGE analysis results for each PFOA treatment time, the QIAGEN Ingenuity Pathway Analysis software package (version Spring 2023.I, QIAGEN, Redwood City, CA, USA) was used to analyze genes whose expression significantly changed (FDR < 0.05) in the PFOA treatment group compared with the solvent control group. The values of the log2 fold change in the PFOA-treated group relative to the solvent control group and the gene names were also analyzed using this software.

## 3. Results

### 3.1. Focus Formation in the CTA of Bhas 42 Cells in the Stationary Phase (Bhas 42 CTA Promotion Test)

**Figure 1:**
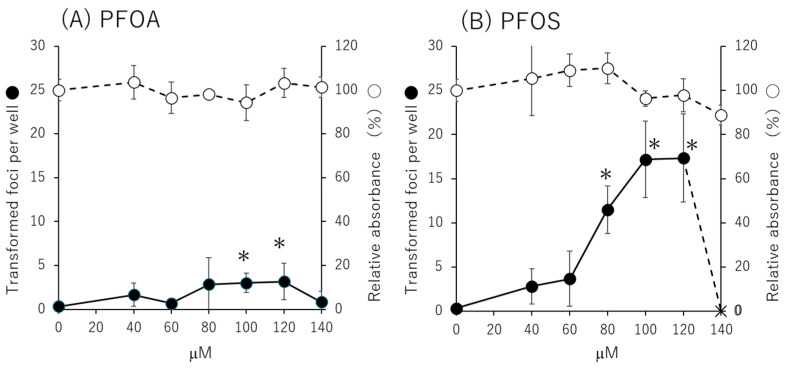
Transformation assay and cell growth assay results in the Bhas 42 cell transformation assay (CTA). Promotional study (stable-stage study) results of the Bhas 42 CTA for treatment with (**A**) perfluorooctanoic acid (PFOA) and (**B**) perfluorooctanesulfonic acid (PFOS). Transformation assays were performed in 6 wells per concentration, and cell proliferation assays were performed in 3 wells per concentration. ● Average number of transformed foci per well; × no cells in the well; ◯ average relative absorbance percentage in cell growth assay; * Dunnett’s test, one-sided, with *p* < 0.05.

The Bhas 42 CTA was performed using PFOA and PFOS at concentrations ranging from 20 μM to 140 μM at 20 μM intervals. As a result (Figure 1), the number of transformed foci statistically significantly increased at 100 μM and 120 μM for PFOA compared with the solvent control group. For PFOS, the number of transformed foci statistically significantly increased at 80 μM, 100 μM, and 120 μM compared with the solvent control group. The number of foci per well was 0.3 ± 0.5 for 0.1% dimethyl sulfoxide (DMSO) as a negative control and 10.5 ± 3.3 for 50 ng/mL phorbol 12-myristate 13-acetate (TPA) as a positive control.

### 3.2. Detection of Differentially Expressed Genes in Cap Analysis of Gene Expression (CAGE) Analysis

#### 3.2.1. Venn Diagram of the Number of Upregulated or Downregulated Genes

**Figure 2:**
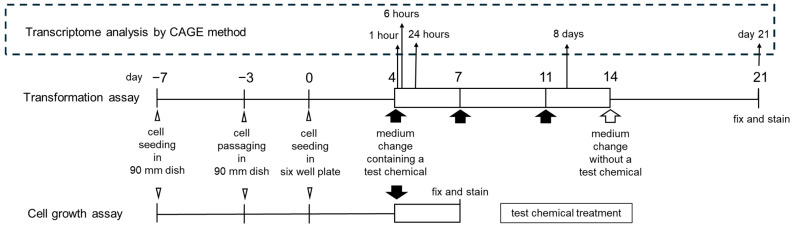
Timeline for the Bhas 42 CTA promotion test and transcriptome analysis with the Cap Analysis of Gene Expression (CAGE) method. RNA samples for CAGE analysis were prepared from three biological replicates from the independent thawing of stock cells to prepare 3 samples (*n* = 3) for each point.

To determine the gene expression profiles of Bhas 42 cells treated with 100 μM (41.4 μg/mL) PFOA, three biological replicates were prepared for each PFOA treatment time (i.e., 1, 6, and 24 h and 8 days) and the assay endpoint (day 21) (Figure 2).

**Figure 3:**
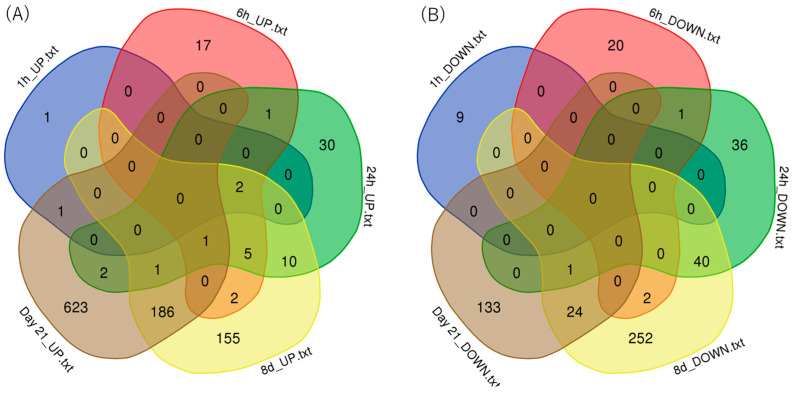
Venn diagrams of the numbers of genes upregulated or downregulated in the CAGE analysis: (**A**) upregulated genes and (**B**) downregulated genes.

The numbers of upregulated or downregulated genes between treatment time groups are shown in Venn diagrams (Figure 3). The number of genes upregulated by PFOA was the highest on day 21 at 814, and the number of downregulated genes was the highest with the treatment for 8 days at 319.

#### 3.2.2. Gene Ontology (GO) Terms

**Figure 4:**
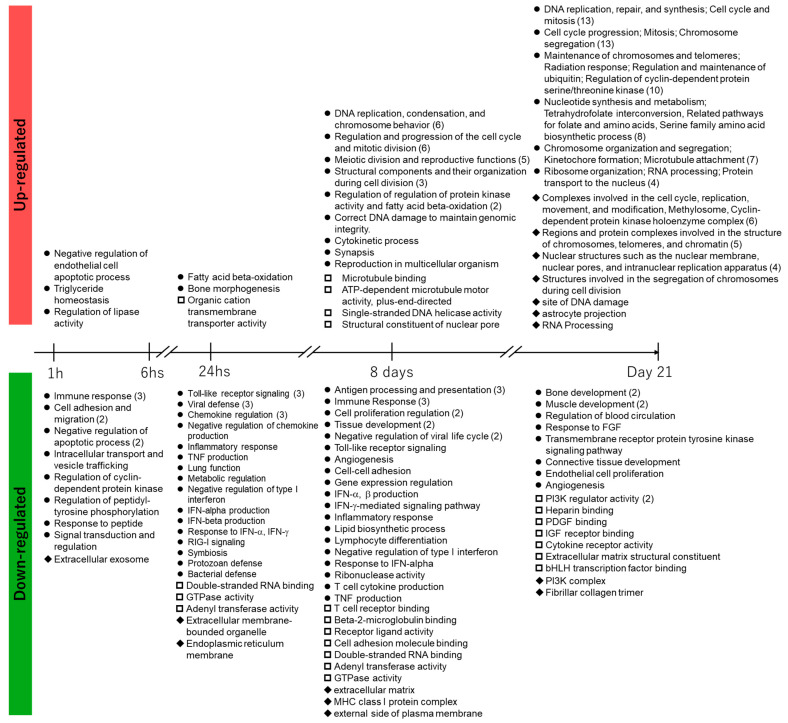
Summary of Gene Ontology terms in chronological order. ● biological process; □ molecular function; ◆ cellular component; ( ) number of classified GO terms.

Data for significantly upregulated or downregulated genes were extracted at every RNA sampling point. The extracted genes were functionally classified according to biological processes (BPs) in GO. The statistically significant GO terms were extracted in each group of differentially expressed genes using the Benjamini–Hochberg method (Tables S1 and S2). To determine the hierarchical structure of the extracted GO terms, QuickGO, an online analysis tool, was used. GO terms in red in the table show the GO terms and false discovery rate (FDR)-corrected *p*-values at the bottom of the hierarchical tree output created using the Explore Biology feature in QuickGO. Among the red GO terms in Tables S1 and S2, similar GO terms for each time point were classified and summarized in chronological order (Figure 4).

The BPs associated with gene upregulation due to 1 h of treatment with PFOA were negative regulation of endothelial cell apoptotic process, triglyceride homeostasis, and regulation of lipase activity, which is related to the upregulation Angptl4 considering its role in lipid metabolism.

After 6 h of treatment with PFOA, no GO terms were enriched with an FDR-corrected *p*-value of <0.01. After 24 h of treatment with PFOA, the GO terms for fatty acid beta-oxidation, bone morphogenesis, and organic cation transmembrane transporter activity were enriched. The corresponding upregulated genes were *Etfb*, *Eci2*, *Cpt1a*, and *Acaa2* (fatty acid beta-oxidation), *Has2*, *Ski*, *Chsy1*, and *Cyp26b1* (bone morphogenesis), and *Slc25a42*, *Atp13a3*, and *Slc25a20* (organic cation transmembrane transporter activity).

The genes upregulated after 8 days of treatment with PFOA led to the enrichment of associated GO terms that were mostly related to DNA replication and mitosis, in addition to the regulation of ubiquitin–protein ligase activity and fatty acid beta-oxidation. The upregulated genes included *Etfb*, *Aldh1l2*, *Cpt1a*, *Acadl*, *Decr1*, *Etfa*, *Acaa2*, and *Mtln* (in fatty acid beta-oxidation) and *Cdc20*, *Fzr*1, *Mastl*, *Mad2l1*, *Plk1*, and *Ube2s* (in regulation of ubiquitin–protein ligase activity).

The genes upregulated on day 21 after PFOA treatment led to the enrichment of associated GO terms that were mostly related to DNA replication and mitosis, in addition to the serine family amino acid biosynthetic process, tetrahydrofolate interconversion, telomere maintenance, positive regulation of establishment of protein localization to telomere, cellular response to ionizing radiation, astrocyte projection, and methylosomes.

The GO analysis of the PFOA treatment for 8 days and that on day 21 showed enrichment of GO terms related to cell division and protein phosphorylation. Regarding protein phosphorylation, “regulation of protein kinase activity” was enriched after 8 days of PFOA treatment, and on day 21, “regulation of cyclin-dependent protein serine/threonine kinase activity” and “cyclin-dependent protein kinase holoenzyme complex” were enriched.

Various genes were downregulated by 1 h of treatment with PFOA, leading to the enrichment of two associated GO terms related to cell adhesion. After 6 h of treatment with PFOA, there was no enrichment of GO terms with an FDR-corrected *p*-value of <0.01. The enriched GO terms corresponding to gene downregulation due to 24 h of treatment with PFOA were related to inflammation and immunity, such as cytokines and chemokines, including interferons. For gene downregulation after 8 days of treatment with PFOA, many GO terms related to immunity were enriched. For gene downregulation on day 21 after PFOA treatment, the *p*-values were low for the molecular function (MF) categories of extracellular matrix structural constituents and 1-phosphatidylinositol-3-kinase regulator (PI3K) activity, as well as the BP category of the transmembrane receptor protein tyrosine kinase signaling pathway.

#### 3.2.3. Collation of the Pathways of Cell Transformation and the Hallmarks of Cancer

##### Xenobiotic Metabolism (Table 1)

- Phase 1 Functionalization of Compounds

The CAGE analysis showed increased gene expression of *CYP26B1* after 24 h of PFOA treatment. Osanai et al. showed that CYP26A1-mediated retinoic acid depletion enhances tumor malignancy, suggesting CYP26A1 as a candidate oncogene. They also found increased CYP26 expression in various cancers and provided evidence for its oncogenic and cell survival properties of CYP26 enzymes [12].

- Aryl Hydrocarbon Receptor Signaling

After 8 and 21 days of PFOA treatment, increased gene expression of *ALDH* was shown. ALDH1 is a recognized marker of cancer stem cells (CSCs) and is highly expressed in various cancers. It contributes to tumorigenesis by maintaining CSC properties, altering metabolism, and promoting DNA repair [13]. The ALDH1 gene family serves as a robust but limited predictor for several solid tumors, including breast, colon, non-small-cell lung cancer (NSCLC), ovarian, and other cancers [14].

- Xenobiotic Metabolism Signaling

On day 21 after the PFOA treatment, decreased gene expression of extracellular superoxide dismutase (SOD3) and increased gene expression of *CYP3A7* and *RAS* were shown. SOD3 is an antioxidant enzyme that is usually repressed in the tumor milieu. Low SOD3 levels are associated with increased cancer incidence and poor prognosis [15,16,17,18,19,20]. *CYP3A7* is reportedly overexpressed in hepatocellular carcinoma [21,22].

##### Evading Anti-Growth Signaling (Table 2)

- Gap Junction

Regarding gap junction signaling, the CAGE analysis showed increased gene expression of the connexin gap junction beta-4 (*GJB4*) after 6 h of exposure to PFOA. The relationship between *GJB4* and cancer was already discussed in the section titled “Transcription, Invasion, and Malignant Transformation of Cancer”.

- Hippo Signaling

The CAGE analysis on day 21 showed increased gene expression of transcriptional enhanced associate domain (*TEAD*). Huh et al. reviewed the relationship between TEAD and cancer [23], concluding that high levels of TEAD expression have been associated with poor prognosis and functions as a biomarker across multiple solid tumors, including prostate, colorectal, gastric, breast, germ cell, head and neck squamous cell, renal, and medulloblastoma cancers [24,25,26,27,28,29,30,31,32,33,34,35]. Epithelial–mesenchymal transition (EMT) plays a pivotal role in regulating cancer stem cell (CSC) traits and is essential for metastatic progression [36]. TEADs act as key effectors of EMT and metastasis during tumor development. Extensive research indicated that TEAD-dependent transcription, activated by Yes-Associated Protein (YAP) and tafazzin (TAZ), promotes cellular transformation through the induction of epithelial–mesenchymal transition (EMT) [37,38,39,40,41]. TEAD activation disrupts cell–cell adhesion, upregulates mesenchymal gene expression, and facilitates enhanced cell migration and invasion. TEAD hyperactivation promotes metastasis in mammary carcinoma and melanoma through a YAP-dependent mechanism, indicating that TEAD–YAP interaction is essential for EMT and metastasis [41].

##### Resisting Programmed Cell Death (Table 2)

- Apoptosis Signaling

After 8 days of PFOA treatment, decreased gene expression of *BID* and *TBID* was shown. When *BID* expression is low, tumor cells survive and enter senescence [42]. Therefore, at 8 days, the tumor cells may have survived and entered senescence rather than undergoing cell death. At day 21, the increase in casp3 gene expression suggested that the tumor cells may have begun to undergo cell death.

- Autophagy

On day 21 after the PFOA treatment, increased gene expression of autophagy-related protein 5 (*ATG5*) and autophagy-related protein 9B (*ATG9B*) was observed. *ATG9B* is an important potential target gene for CRC metastasis. Mechanistically, ATG9B promoted CRC invasion primarily in an autophagy-independent manner [43].

- Microautophagy Signaling Pathway

On day 21 after the PFOA treatment, increased gene expression of translocase of outer mitochondrial membrane 20 (*TOMM20*) was shown.

*TOMM20* overexpression enhances mitochondrial ATP production, thereby promoting proliferation, migration, and invasion of CRC cells. Elevated *TOMM20* levels in CRC tissues are associated with cell cycle dysregulation, causing increased cell proliferation, as well as the invasiveness of cancer cells. Furthermore, EMT activation, followed by increased *TOMM20* expression, leads to increased migration and invasiveness of CRC cells [44].

##### Avoiding Immune Destruction (Table 3)

- Immunogenic Cell Death Signaling

In terms of immunogenic cell death signaling, the CAGE analyses showed increased *HSP90* and *70* after 8 days of PFOA treatment and on day 21. HSP90 and HSP70 are essential chaperone proteins involved in almost all stages of tumor development. HSP90 and HSP70 homologs modulate diverse cellular processes, including apoptosis, the unfolded protein response, lipid metabolism, metastasis, angiogenesis, autophagy, and both innate and adaptive immunity, through distinct signaling pathways [45].

The CAGE analysis showed increased gene expression of *CASP3*, *CASP8*, and *ATG12/ATG5/ATG16L1* after 8 days of PFOA treatment. The association between cancer and increases in *CASP3*, *CASP8*, and *ATG12/ATG5/ATG16L1* is described in the sections titled “Apoptosis”, “Exocytosis”, and “Autophagy”, respectively.

- Interferon Signaling

In terms of interferon signaling, our CAGE analysis showed that *interferon-induced transmembrane protein 3 (IFIT3)*, *ISG15*, *OAS1*, and *STAT1* expression decreased after 24 h and 8 days of PFOA treatment. In addition, the expression of *interferon-induced transmembrane protein 1 (IFIT1)* and *TAP1* was decreased after 24 h of PFOA treatment, and *IFI35*, *IRF9*, and *STAT2* were decreased after 8 days of PFOA treatment.

Wan et al. reviewed the role of STAT1 in cancer biology and reported the following [46]. STAT1 generally acts as a tumor suppressor. *STAT1* target genes, reflecting their physiological role in antiviral and innate immunity, regulate processes such as cell cycle arrest, apoptosis, anti-angiogenesis, and immune recognition.

- Programmed cell death-1 (*PD-1*) and programmed cell death ligand 1 (*PDL-1*) Cancer Immunotherapy Pathway

In terms of the *PD-1* and *PDL-1* cancer immunotherapy pathway, the CAGE analysis showed increased gene expression of *protein phosphatase 2A (PP2A) inhibitor (cancerous inhibitor of protein phosphatase 2A (CIPA2))* after 8 days of PFOA treatment and on day 21. Soofiyani et al. reviewed the association between CIPA2 and cancer, and their findings are summarized in the following lines [47]. CIP2A is an endogenous protein in human cancer cells that directly interacts to c-Myc and hinders the function of PP2A toward c-Myc S62 induction. CIP2A promotes anchorage-independent growth and is essential for malignant cell proliferation. Notably, KI-AA1524/CIP2A also interacts with several phosphorylated PP2A substrates involved in tumor growth, including c-Myc, polo-like kinase 1 (Plk1), E2F1, Akt, and death-associated protein kinase 1 (DAPK1) [48]. Further details regarding the relationship between CIPA2 and cancer are provided in the Section 4.

##### Tumor-Promoting Inflammation (Table 3)

- IL-1 Signaling

In terms of IL-1 signaling, the CAGE analysis showed decreased gene expression of *Ikb* after 1 h and increased gene expression of *JUN* on day 21 after PFOA treatment. In PPAR Signaling which will be described later, the gene expression of *Il1 receptor* was increased on day 21 after PFOA treatment.

- Activin–Inhibin Signaling Pathway

In terms of the activin–inhibin signaling pathway, the CAGE analysis after 6 h of PFOA treatment showed increased gene expression of activin and inhibin A. Regarding the relationship between activin and cancer, Mancinelli et al. [49] reported evidence indicating that activin A signaling is important in several cancers, including CRC [50,51,52] and mammary [53], lung [54], esophageal [55], and pancreatic cancers [56].

Staudacher et al. demonstrated that transforming growth factor-beta (TGFβ) stimulates activin A secretion from colon stromal cells, thereby promoting activin A–dependent migration of colorectal cancer epithelial cells in CRC models [57]. Bauer et al. showed that elevated TME stiffness enhances activin A secretion from colon stromal cells, facilitating the migration of colorectal cancer epithelial cells [51]. Mancinelli et al. reported that stromal cells within the pancreatic TME produce activin A [49]. The dense stromal TME contributes to elevated activin A levels, which in turn promote epithelial–mesenchymal transition (EMT), cell migration, and metastasis in tumor epithelial cells.

In the CAGE analysis, *lipase E (LIPE*) gene expression was increased after 24 h and 8 days of PFOA treatment.

Staege et al. reviewed the involvement of LIPE in prostate cancer and cancer cachexia [58]. LIPE (also known as hormone-sensitive lipase) seems to be directly involved in the pathobiology of castration-resistant prostate cancer cells [59], promotes androgen synthesis in castration-resistant prostate cancer cells by converting cholesteryl esters into cholesterol and free fatty acids, including arachidonic acid. This process supports androgen independence and is also linked to cancer cachexia [60,61]. The presence of factors that increase the expression of LIPE in adipocytes in patients with cancer has been observed [62].

- IL-4 Signaling

The CAGE analysis showed increased *IL4R* gene expression after 24 h of PFOA treatment and increased gene expression of *IL13RA1* and *PI3K* after 8 days of PFOA treatment. The relationship of IL4 and IL13 with cancer was reviewed in [63]. IL-4 bind to three distinct receptor subunits in various combinations, resulting in the formation of three unique IL-4 receptor complexes [64,65]. Both IL-4 and its receptor contribute to the maintenance and proliferation of CSCs. Francipane et al. reported that co-administration of an IL-4Rα antagonist or an IL-4 neutralizing antibody significantly and durably improved the therapeutic efficacy of oxaliplatin and/or 5-fluorouracil in mice bearing human colorectal cancer spheroids enriched with CSCs [66,67]. IL-4Rα is highly expressed in human bladder cancer tissues, and its overexpression is associated with a more advanced grade and stage [68].

- Role of Janus Kinase (JAK) Family Kinases in IL-6-Type Cytokine Signaling

Regarding the role of JAK family kinases in IL-6-type cytokine signaling, in the CAGE analysis on day 21 after PFOA treatment, *IL11* gene expression was increased. The relationship between IL11 and cancer was reported in [69]. IL-11 is scarcely detectable in the body fluids of healthy individuals, yet its serum levels are elevated in various pathological conditions, such as arthritis [70], acute pancreatitis [71], pancreatic cancer [72], lipedema [73], polycythemia vera [74], lung disease in patients with rheumatoid arthritis [75], and major cardiac events in chronic heart failure [76]. These findings underscore the involvement of IL-11 in diverse disease states, particularly in cancer and inflammation. A few preclinical studies have explored therapeutic targeting of IL-11 or its signaling pathways in different types of cancers [77,78,79,80,81].

- Tumor necrosis factor receptor 2 (TNFR2) Signaling

On day 21 after PFOA treatment, *tumor necrosis factor alpha-induced protein 3 (TNFAIP3)* gene expression was increased. Gao et al. reported that TNFAIP3 is necessary for the promotion of tumor growth and progression in breast cancer by fibroblast growth factor receptor 1 signaling [82]. Furthermore, Feng et al. reported that an in vivo analysis displayed that TNFAIP3-silenced MDA-MB-231 xenografts developed smaller tumors, and ALDH immunostaining levels were significantly lower in TNFAIP3-depressing or TNFAIP3-knockout tumor tissues [83].

##### Tumor Environment (Table 4)

- Tumor Microenvironment Pathway

On day 21 after PFOA treatment, *CSF2*, *PTGF2*, and *Ap1* gene expression were increased.

CSF2 (granulocyte–macrophage colony-stimulating factor, GM-CSF), a member of the CSF family, is produced and secreted by various cell types, including cancer cells [84,85]. In gastric cancer, CSF2 mediates chemotherapy responses and leads to tumor progression [86]. In pancreatic cancer, CSF2 promotes polarization of cancer-associated macrophages and supports maintain metabolic homeostasis via the PI3K/AKT signaling pathway [87].

PTGS2 (prostaglandin–endoperoxide synthase 2, COX-2) is upregulated in a variety of premalignant and malignant solid tumors, including those of the stomach, esophagus, liver, pancreas, head and neck, lung, breast, and prostate [88]. PTGS2 (COX-2) is critically involved in CRC progression, while its inhibition suppresses tumor growth and enhances overall survival [89]. As an immediate-early response gene, PTGS2 is typically undetectable in most cells but is rapidly induced at inflammatory sites by stimuli such as pro-inflammatory cytokines (IL1A/B, IFNG, TNFα) released from inflammatory cells, as well as by tumor promoters like TPA and RAS, both in vitro and in vivo [90,91]. The transcription of PTGS2 is regulated by several transcription factors, including AP-1.

- JAK/Signal transducer and activator transcription (STAT) Signaling

JAK/STAT signaling and the CAGE analysis showed increased gene expression of *PTPN*6 on day 21 after PFOA treatment. The *PTPN6* gene may act as an oncogene in promoting colon cancer, and it also plays an important role in regulating colon cancer cell proliferation, migration, and invasion [92].

- Cancer-Associated Fibroblasts (CAFs)

Wright et al. presented a review titled “Cancer-Associated Fibroblasts: Master Tumor Microenvironment Modifiers” in which they described signaling molecules and growth factors secreted by CAFs into the TME [93]. Among these factors, although not presented as a CAF pathway or table, our CAGE analysis showed increased gene expression of IL-11 on day 21 after PFOA treatment.

In their review, Zhao et al. described the role of CAFs labeled with cancer-promoting markers in various kinds of cancer [94]. Of these cancer-promoting markers, we observed increases in *POSTN* gene expression after 8 days of PFOA treatment. POSTN is a stromal cell protein with roles in cancers. In pancreatic cancer, it is associated with highly cellular tumors, macrophage infiltration, and shorter overall survival. In breast cancer, it plays an important role in in situ carcinogenesis and, perhaps, subsequently, cancers that become invasive. In CRC, it is correlated with tumor progression, lymph node and distant metastases, and poor clinical outcomes. In lung cancer, it serves as a prognostic marker.

##### Tissue Invasion and Metastasis (Table 4)

- Glioma Invasiveness Signaling

After 8 days of PFOA treatment and on day 21, the gene expression of *hyaluronan-mediated motility receptor (HMMR)* and plasminogen activator, *urokinase receptor (PLAUR)* was increased. HMMR, which is related to glycosaminoglycan hyaluronic acid, is highly expressed in various malignancies, including breast, bladder, and prostate cancers [95,96,97,98]. Increased HMMR expression has been associated with poor prognosis, as it promotes tumor growth and metastasis. Regarding PLAUR, small interfering RNA (siRNA)-mediated knockdown of PLAUR demonstrated notable inhibition of cell proliferation and migration in clear-cell renal cell carcinoma [99].

- Focal Adhesion Kinase (FAK) Signaling

Regarding FAK signaling, *EPH* and *ETV4* gene expression was increased on day 21 after PFOA treatment.

Regarding Eph, Pasquale reported the following in a review on Eph receptors and ephrins in cancer progression [100]. The Eph system controls tumor expansion, invasiveness, and metastasis. In addition to functioning within cancer cells, the Eph system mediates the reciprocal communication between cancer cells and cells of the TME. The involvement of the Eph system in tumor angiogenesis is well established.

ETV4 is upregulated in various malignancies and exerts pro-tumoral effects [101,102,103,104,105]. In breast cancer, ETV4 promotes metastasis by transcriptionally activating EMT inducers [106,107] and extracellular matrix–degrading proteinases [108].

- Cold-shock-domain-containing E1 (CSDE1) Signaling Pathway

In terms of CSDE1 signaling, after PFOA treatment in *heterogeneous nuclear pibonucleoprotein C (HNRNPC)*, there were increases in gene expression on day 21 according to the CAGE analysis. In patients with NSCLC, HNRNPC predicted poor prognosis and was correlated with tumor invasion and lymph node metastasis. RNA-seq data revealed that HNRNPC is involved in cell growth and migration, extracellular matrix organization, and angiogenesis [109]. Liyi et al. reviewed the role of HNRNPC dysregulation in cancer [110]. High expression of HNRNPC has been found in many kinds of cancers and is always an indicator of poorer prognosis; this has been demonstrated in breast cancer [111], glioblastoma multiforme [112], and gastric cancer [113]. Therefore, HNRNPC is a candidate biomarker and is potentially valuable for prognostic evaluation. The relationship between HNRNPC and proteins is summarized in the following. p53 acts as a tumor suppressor in many tumor types [114]. HNRNPC was also discovered to interact with p53 by directly binding to p53 and could make p53 unstable, prevent its activation, and downregulate its protein level [115]. Increased HNRNPC expression was found to be significantly associated with advanced tumor stage and metastasis. HNRNPC overexpression significantly promotes lung cancer cell proliferation, migration, and invasion in vitro and in vivo.

##### Sustained Growth Signaling (Table 5)

- Cyclins and Cell Cycle Regulation

After 8 days of PFOA treatment and on day 21, the gene expression of cyclin-dependent kinases (CDKs) and cyclins in the category of cyclins and cell cycle regulation was increased. The gene expression of *cyclins A*, *B,* and *E* and *CDK1* and *2* increased on day 21 after PFOA treatment. Various reviews have described the relationships between these CDKs, cyclins, and cancer [116,117,118].

- p21-Activated Protein Kinase (PAK) Signaling

Radu et al. reviewed PAK signaling in cancer development and progression, as described in [119]. PAKs are serine/threonine kinases that function at the intersection of multiple oncogenic signaling pathways. Activation of PAK isoforms—via mutation, overexpression, or upstream regulators such as Rac and Cdc42—elicits oncogenic effects, including growth signal independence, resistance to apoptosis, and enhanced invasion and metastasis. These processes are regulated through various cytoskeletal effectors, such as guanine nucleotide exchange factors (GEFs), GTPase-activating proteins (GAPs), guanine nucleotide dissociation inhibitors, and actin-associated proteins that modulate Rho family GTPase activity.

Our CAGE analysis showed increased gene expression of Rac GTPase-activating protein 1, encoding a GTPase-activating protein *(GAP)*, Rho Guanine Nucleotide Exchange Factor (GEF) 39 (*ARHGEF39*), Rho GTPase Activating Protein 19 (*ARHGAP19*) and Rac GTPase Activating Protein 1 (*RACGAP1*) after 8 days of PFOA treatment and on day 21, in addition to increased *ARHGAP33* at 8 days after PFOA treatment. On day 21 *ARHGAP22* increased and *AHRGAP44* decreased (not included in S87 and S88 pathways).

- Hox transcript antisense intergenic RNA (HOTAIR) Regulatory Pathway

In terms of the HOTAIR regulatory pathway, the CAGE analysis showed increased gene expression of *Forkhead Box M1 (FOXM1)* after 8 days of PFOA treatment and on day 21. The protein encoded by the *FOXM1* gene is a transcriptional activator involved in cell proliferation. FOXM1 transcriptionally activates HMMR expression via promoter binding. FOXM1 promotes pEMT and the growth of bladder cancer cells partly via HMMR [97].

Other signals participating in sustained growth signaling are also listed in Table 5.

##### Genetic Instability (Table 6)

- Cell Cycle: G2/M DNA Damage Checkpoint Regulation

In terms of G2/M DNA damage checkpoint regulation, the CAGE analysis showed increased gene expression of *TOP2a* after 8 days of PFOA treatment and on day 21. Chen et al. reviewed the role of TOP2A in human cancer [120]. Abnormal alterations in TOP2A, its interacting proteins, and its modifications may have a critical role in chromosomal instability (CIN) in human cancers. Further details regarding the relationship between CIN and cancer involving TOP2A are provided in the Section 4.

- DNA Methylation and Transcriptional Repression

In terms of DNA methylation and transcriptional repression signaling, the gene expression levels of *Sin3A-associated protein 30* and transcription factors were increased after 8 days of PFOA treatment.

On day 21, the gene expression of *DNMT1*, *UHRF1*, *transcription factors*, and *SUV39H1* was increased. SUV39H1 mediated the enrichment of H3K9me3 at the promoter region of MCPIP1, repressing the MCPIP1-mediated degradation of AURKA and facilitating the subsequent accumulation of AURKA [121]. As shown in the section titled “Cell Cycle: G2/M DNA Damage Checkpoint Regulation”, the gene expression of *AURKA* was increased after 8 days of PFOA treatment and on day 21.

Other signals related to genetic instability are also listed in Table 6. The expression of genes related to genetic instability was upregulated after 8 days of PFOA treatment and on day 21.

##### Enabled Replication Immortality (Table 7)

- Senescence-Associated Secretory Phenotype (SASP)

In terms of the SASP, the CAGE analysis showed increased gene expression of the nucleosome proteins *IL-6* and *IL-8* after 8 days of PFOA treatment and on day 21.

Wang et al. reviewed senescent cells in cancer therapy, and their findings can be summarized as follows. IL-6 is a key SASP factor that can promote proliferation in a paracrine manner by binding to the IL-6 receptor and subsequently activating STAT3. SASP factors function in the senescence of cells by activating the NF-κB, C/EBPβ, and p38MAPK pathways. SASP factors contribute to various aspects of cancer progression; IL-6 and IL-8 promote EMT.

IL-6 and IL-8 are key mediators of cellular invasiveness, as neutralizing antibodies targeting these interleukins suppress invasion, and supplementation with recombinant IL-6 or IL-8 enhances the invasiveness of preneoplastic epithelial cells co-cultured with non-senescent fibroblasts [122]. Both cytokines activate STAT3 signaling, which in turn induces the transcription of multiple MMPs and facilitates cancer cell invasion [123,124].

SASP also contributes to tumorigenesis by inducing EMT. Exposure of non-aggressive breast cancer cells to conditioned media from senescent fibroblasts induces EMT hallmarks, including downregulation of β-catenin and E-cadherin and upregulation of vimentin, reflecting a mesenchymal phenotype [125]. IL-6 and IL-8 have been shown to induce EMT in cancer cells in vitro [126,127].

- Telomerase Signaling

In terms of telomerase signaling, the CAGE analysis showed increased gene expression of *dyskeratosis congenita 1 (DKC1)* after 8 days of PFOA treatment.

Kan et al. reported the relationship between DKG1 and lung adenocarcinoma (LUAD) [128]. *DKC1* is upregulated in LUAD relative to adjacent normal tissues, and its high expression is associated with poor overall survival. Knockdown of *DKC1* in LUAD cell lines induces G1 phase arrest and suppresses cell proliferation, whereas ectopic *DKC1* expression restores cell growth. *DKC*1 abundance positively correlates with levels of telomerase RNA component (TERC) and telomerase reverse transcriptase in LUAD. Downregulation of *DKC1* reduces TERC expression, diminishes telomerase activity, and leads to telomere shortening, ultimately resulting in cellular senescence and apoptosis. Collectively, these findings indicate that elevated *DKC1* expression predicts poor prognosis in LUAD, and that *DKC1* downregulation triggers telomere-dependent senescence and apoptosis.

- Telomere Extension by Telomerase

In terms of telomere extension by telomerase, the CAGE analysis showed increased gene expression of *MRE11* on day 21. It has been reported that MRE11 promotes tumorigenesis by promoting resistance to oncogene-induced replication stress [129].

- Wnt/β-Catenin Signaling

In terms of Wnt/β-catenin signaling, the CAGE analysis showed increased expression of *Wnt* genes after 8 days of PFOA treatment. Therefore, Wnt is ON in Wnt/β-catenin signaling, and this suggests that it could translocate into the nucleus in a stable state. The CAGE analysis showed increased gene expression of *GJA1*, *CCND1*, and *JUN* on day 21 after PFOA treatment.

Other signals related to enabled replication immortality are also listed in Table 7.

##### Inducing New Blood Flow (Table 7)

- Angiopoietin Signaling

In terms of angiopoietin signaling, the CAGE analysis showed increased *BIRC5* (survivin) gene expression after 8 days of PFOA treatment and on day 21. Regarding the relationship between BIRC5 and angiogenesis, Wang et al. [130] reported that survivin promotes glioma angiogenesis via the upregulation of vascular endothelial growth factor (VEGF) and basic fibroblast growth factor in vitro and in vivo. In our CAGE analysis, the gene expression of *VEGF* increased on day 21, as described in the section titled “VEGF signaling”.

- VEGF Signaling

In terms of VEGF signaling, the CAGE analysis confirmed the increased gene expression of *VEGF* and *eukaryotic translation initiation factor (EIF)* related to angiogenesis on day 21.

##### Deregulated Cellular Metabolism (Table 8)

- Fatty Acid Beta-Oxidation I

In terms of fatty acid beta-oxidation I, the CAGE analysis showed increases in the gene expression of *dodecanoyl-CoA D-isomerase and acetyl-CoA C-acyltransferase* after 24 h of PFOA treatment, the gene expression of *long-chain-fatty-acid-CoA ligase and acetyl-CoA C-acyltransferase* after 8 days of PFOA treatment, and the gene expression of *long-chain-fatty-acid-CoA* on day 21 after PFOA treatment.

- Mitochondrial Fatty Acid Beta-Oxidation

In terms of mitochondrial fatty acid beta-oxidation, the CAGE analysis showed increased gene expression of *ACCA2* tetramer after 24 h and 8 days of PFOA treatment, with *ACADL tetramer* and *DECR1 tetramer* also being increased after 8 days of PFOA treatment, and there were increases in *ACHOT2,9* and *THEM4,5 dimers* on day 21 after PFOA treatment.

- PPAR Signaling

Regarding oxidative stress caused by PFOA in terms of PPAR signaling, the gene expression of *NRIH3*, which suppresses *PPARA*, was decreased in the CAGE analysis after 8 days of PFOA treatment. On the other hand, the gene expression of *NCOA*, which activates *RXRA*, was also decreased. The gene expression of *Il1 receptor*, *RAS*, *JUN* and *PTGS2* were increased on day 21 after PFOA treatment.

- Adenosine Monophosphate-Activated Protein Kinase (AMPK) Signaling

In terms of AMPK signaling, the CAGE analysis showed increases in the gene expression of *hydroxymethylglutaryl-CoA reductase*, which is the rate-limiting enzyme for cholesterol synthesis, after 8 days of PFOA treatment and on day 21. The relationship between cholesterol synthesis and cancer is discussed in the section titled “Superpathway of Cholesterol Biosynthesis”.

The CAGE analysis showed increased expression of *carnitine palmitoyltransferase 1A (CPT1A)* after 24 h of PFOA treatment, as well as after 6 h and 8 days. Carnitine palmitoyltransferase 1, particularly its liver isoform CPT1A, is the rate-limiting enzyme of fatty acid beta-oxidation in many tissues that catalyzes the transfer of long-chain acyl group of acyl-CoA ester to carnitine, thereby shuttling long-chain fatty acids into the mitochondrial matrix through the carnitine transporter for beta-oxidation [131]. Several studies have shown that CPT1A-driven fatty acid beta-oxidation can facilitate cancer cell proliferation and survival, as well as tumor invasion [132,133,134]. Liu et al. revealed that CPT1A-mediated fatty acid beta-oxidation is required for pro-survival signaling in cancer cells under cytolytic immune pressure [135].

After 24 h and 8 days of PFOA treatment, the gene expression of *LIPE* was increased. The relationship between LIPE and cancer is discussed in a previous section titled “Activin Inhibin Signaling Pathway”.

The role of AMPK in cancer metabolism and its impact on the immunomodulation of the TME has been reviewed by Keerthana et al. [136]. AMPK serves as a key metabolic sensor essential for cellular energy homeostasis and exerts broad metabolic and physiological effects beyond its primary roles in glucose and lipid metabolism. Activation of AMPK and its downstream pathways induces significant alterations in tumor cell bioenergetics. Substantial evidence indicates that AMPK functions as a tumor suppressor by modulating inflammatory and metabolic signaling. Moreover, AMPK is crucial for the phenotypic and functional reprogramming of diverse immune cell populations within the TME. AMPK-driven inflammatory responses promote the recruitment of specific immune cells to the TME, thereby inhibiting tumor initiation, progression, and metastasis. Therefore, AMPK appears to play an important role in regulating the antitumor immune response by regulating the metabolic plasticity of various immune cells

- Superpathway of Cholesterol Biosynthesis

In terms of the superpathway of cholesterol biosynthesis, the CAGE analysis indicated that the gene expression in many cholesterol biosynthesis enzyme proteins was increased after 8 days of PFOA treatment and on day 21.

Regarding the relationship between cholesterol and cancer, cholesterol biosynthesis is important in tumor stem cell maintenance, as stated in a review by Xiao et al. [137]. Recent studies have identified a mechanism by which hypercholesterolemia-driven production of oxidized low-density lipoprotein (ox-LDL) promotes bladder cancer progression via the regulation of tumor cell stemness [138]. In hypercholesterolemic mouse models—generated either by high-fat, high-cholesterol diets or *Ldlr* gene knockout—elevated serum cholesterol was shown to enhance tumor cell stemness and accelerate bladder cancer development. Conversely, treatment with the cholesterol absorption inhibitor ezetimibe in hormone-induced hypercholesterolemic mice significantly reduced tumor cell stemness and slowed tumorigenesis, indicating a critical role for cholesterol in the malignancy of bladder cancer in these models.

- Folate Signaling Pathway

In the folate signaling pathway, the CAGE analysis after 8 days of PFOA treatment showed increased gene expression of *SHMT2* and *ALDH1L2* in mitochondria. At the same time, there was increased cytoplasmic gene expression of *TYMS*, the target of the anticancer drug 5-fluorourasil. Regarding the relationship of SHMT2 and ALDH1L2 with cancer, Miyo et al. reported on the importance of mitochondrial folate enzymes in human CRC [139]. Expression of the mitochondrial folate metabolism enzymes SHMT2, MTHFD2, and ALDH1L2 was upregulated in colorectal tumor tissues, and their high expression correlated with poor patient prognosis. The mitochondrial folate metabolic pathway, involving SHMT2, MTHFD2, and ALDH1L2, forms a functional cycle that supports CRC cell survival and proliferation. Continuous activation of this pathway confers a growth advantage in CRC. Additionally, SHMT2 suppresses pyruvate kinase activity, thereby reducing carbon entry into the TCA cycle, a mechanism that enhances glioma cell survival under hypoxic conditions [140]. Beyond its importance in methylation, nucleotide synthesis, and DNA repair, the mitochondrial folate pathway also influences tumor biology, contributing to malignancy. The formyl-tetrahydrofolate synthase activity of MTHFD1L has been reported to play an important role in the proliferation of breast cancer cells [141].

##### Transcription, Invasion, and Malignant Transformation of Cancer

After 1 h of PFOA treatment, the gene expression of *nuclear factor kappa B subunit (NFKB) inhibitor alpha*, *Ikb*, *LIF interleukin 6 family cytokine*, *intercellular adhesion molecule 1 (ICAM1*), *TNFAIP3*, *G-protein-coupled receptor*, and *Rho* was decreased.

ICAM1 is upregulated in inflamed tissues and functions as a key receptor mediating immune cell adhesion to endothelial, epithelial, and occasionally other immune cells, thereby facilitating the initiation and progression of inflammatory responses [142].

TNFAIP3 encodes a zinc finger protein and ubiquitin enzyme and has been shown to inhibit NF-kappa B activation, as well as TNF-mediated apoptosis. The Rho family is a low-molecular-weight GTP-binding protein similar to Ras that acts as a monomer and is a main regulator of cell morphology [143].

Cancer malignancy factors were activated after 6 h of PFOA treatment in the Bhas 42 CTA. Regarding gap junction signaling, the CAGE analysis showed increased gene expression of the *connexin GJB4* after 6 h of exposure to PFOA. Muramatsu et al. reported on the role of the GJB4 protein in pancreatic cancer [144], where GJB4 was more highly expressed in pancreatic cancer tissues and could, thus, serve as a valuable biomarker for predicting patients with pancreatic cancer. GJB4 promotes cell proliferation and metastatic activities by activating the MET–AKT pathway and was suggested as a novel target for this deadly cancer. In terms of gap junction signaling, the CAGE analysis on day 21 of PFOA treatment showed increased gene expression of the *connexins gap junction alpha-1* and *gap junction beta-5*, as well as of *F-actin*.

There was increased gene expression of *SNAI1*, a transcription factor that regulates *EMT* in the EMT pathway, after 6 h of PFOA treatment.

After 6 h of PFOA treatment and at day 21, increased transcription of *F-actin*, which is involved in actin cytoskeleton signaling, was shown. Numerous scientific studies have indicated the participation of GTPases from the Rho family, including Rho, Rac, and Cdc42 proteins, in the formation of the leading edge by inducing the accumulation of F-actin in the front of cells [145].

F-actin is a microfilament, and the involvement of actin and actin-binding proteins in carcinogenesis has been reviewed by Izdebska et al. [146]. In cancer cells, actin and actin-binding proteins participate throughout all stages of carcinogenesis. Cellular migration and invasion rely on the formation of actin-rich protrusive structures, mediated by components such as the Arp2/3 complex, filamin A, fascin, α-actinin, and cofilin.

The CAGE analysis after 24 h of PFOA treatment and on day 21 for glioma invasiveness signaling showed an increase in *PLAUA (plasminogen activator, urokinase receptor)* gene expression. Many proteases play essential roles in the invasion mechanism of cancer cells [147]. Among proteases, urokinase-type plasminogen activator (uPA) plays a pivotal role in cancer invasion and metastasis [148,149]. The binding of its amino-terminal fragment to the uPA receptor (uPAR) on cancer cell surfaces is considered a trigger for promoting cancer invasion. In cancer cells, uPARs are assembled in the direction of movement, with the binding of uPA to uPAR. uPA, upon binding to uPAR, efficiently converts inactive plasminogen present on the surface of cancer cells into the active serine protease plasmin, which directly or indirectly dissolves extracellular matrix (ECM) components [150].

After 8 days of PFOA treatment in the Bhas 42 CTA, the CAGE analysis revealed increased gene expression of *CYP26A1*, *B1*, and *C1* in the Phase 1 functionalization of compounds. Osanai et al. reported that retinoic acid depletion caused by *CYP26A1* expression promotes the malignant behavior of tumor cells derived from various tissues, implicating *CYP26A1* as a candidate oncogene [12]. It was shown that the expression levels of CYP26 enzymes are elevated in various types of cancer. Overall, their study provided evidence of the oncogenic and cell survival properties of CYP26 enzymes.

The CAGE analysis after 8 days of exposure to PFOA and on day 21 showed that there was increased expression of *CDK1* and *cyclin B*, which are involved in the cell cycle, specifically at the G2/M DNA damage checkpoint. The tumor promoter substrates of CDK1 have been reviewed by Wang et al. [117].

The CAGE analysis after 8 days of PFOA treatment and on day 21 showed increased expression of *CIP2A* in the *PD-1* and *PDL-1* cancer immunotherapy pathway. CIP2A is discussed in the section titled “PD-1, PDL-1 Cancer Immunotherapy Pathway” in *the Hallmarks of Cancer* above, and in the Section 4 below.

On day 21 after PFOA treatment, the CAGE analysis showed increased gene expression of *RAS*, and cancer-related gene expression and associated signals were also activated.

**Figure 5:**
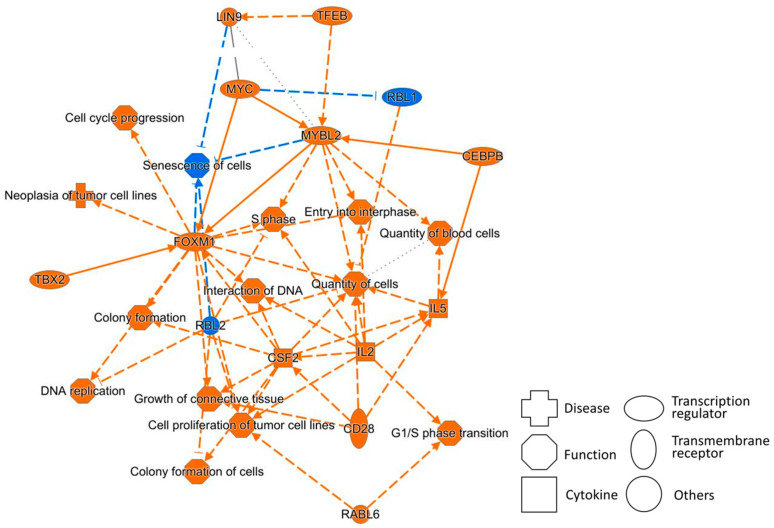
A graphical summary of the results of the CAGE analysis corresponding to day 21 after PFOA treatment. Orange legend, prediction of activation; blue legend, prediction of inhibition; orange line, leading to activation; blue line, leading to inhibition; dotted line, indirect interaction.

Figure 5 shows a graphical summary of the CAGE analysis of Bhas 42 cells on day 21 after PFOA treatment in the Bhas 42 CTA. A graphical summary is used to select and connect a subset of the most significant entities predicted in the analysis, creating a coherent and comprehensible synopsis of the analysis.

The summary shows that the functions of cell cycle progression, the G1/S phase transition, the S phase, and cell proliferation of tumor cell lines are upregulated, in addition to the downregulation of senescence of cells, all of which promote cancer.

In addition, the transcription regulators *MYC*, *MYBL2*, *FOXM1*, *TBX2*, and *RABL6* and the transmembrane receptor *CD28* are upregulated, and the tumor suppressors *RBL1* and *RBL2* are downregulated. These also promote cancer. These networks are also connected to the tables related to the hallmarks of cancer above.

## 4. Discussion

The report “Carcinogenicity of perfluorooctanoic acid and perfluorooctanesulfonic acid” was published in the online news of *The Lancet Oncology* at the end of November 2023 by the working members of the IARC Monograph Volume 135 [2]. PFOA was classified as “carcinogenic to humans” (Group 1), reflecting sufficient evidence of carcinogenicity in experimental animal models and robust mechanistic evidence in exposed human populations. The mechanistic plausibility is substantiated by PFOA-induced epigenetic dysregulation and immunosuppressive activity. Additionally, epidemiological data provide limited evidence of an association between PFOA exposure and increased risk of renal cell carcinoma and testicular cancer in humans.

However, in genotoxicity tests, which have mainly been used as carcinogenicity prediction tests, the Agency for Toxic Substances and Disease Registry (ATSDR) has concluded that PFOA is not mutagenic at noncytotoxic concentrations. Regarding PFOS, all tests were negative except for the micronucleus test. However, compared with the control group, there were higher frequencies of micronucleated polychromatic erythrocytes in the bone marrow at the two highest doses, although the increase was not statistically significant. Additionally, the greater effect in the highest dose group may have been because of an outlier, and suggesting genotoxicity may have been because of tissue toxicity. Therefore, the ATSDR and IARC concluded that there was no evidence of genotoxicity of PFOS [1,3].

The CTA is considered a test method that can detect NGTxCs [4,5,6]. The three CTAs evaluated in the NGTxC IATA [5] are the SHE CTA [151], Bhas 42 CTA [4], and Balb CTA. Two of these CTAs, the SHE CTA and Balb CTA [152,153], have shown positive results for PFOS, as did the Bhas 42 CTA in this study. However, for PFOA, the SHE CTA yielded positive results for treatment with BaP but negative results for treatment with PFOA alone, according to GD214 [154]. The Balb CTA also yielded positive results for PFOS but negative results for PFOA [153].

In the Bhas 42 CTA of this study, positive results were obtained with individual treatments of PFOA and PFOS. Therefore, among the current OECD-accredited tests, the Bhas 42 CTA is the only test method that can correctly assess the carcinogenicity of both IARC Group 1 PFOA and IARC Group 2B PFOS as positive. The results of this study indicate that the Bhas 42 CTA can contribute as a predictive test for NGTxCs not only to evaluate the carcinogenicity of thousands of PFASs, which is a challenge internationally, but also to prevent the future recurrence of problems due to NGTxC exposure.

In addition, the carcinogenicity of the NGTxC PFOA is supported by the positive transformation focus formation results in the Bhas 42 CTA, as well as mechanistic data from transcriptome analyses. This is a significant example that forces us to recognize that PFOA’s carcinogenicity cannot be predicted by means of genotoxicity tests alone, even though it is scientifically classified into Group 1 by the IARC. From this point of view, this is a step toward a solution to the international problem of NGTxCs.

The mechanistic data in Bhas 42 CTA show changes in the expression of various genes in hallmarks of cancer during cell transformation, thus making it possible to perform a detailed analysis over time. These are the advantages of the Bhas 42 CTA is performed in vitro and highly sensitive.

PFOA has immunosuppressive properties. Extensive studies across various exposed populations, including both children and adults, have demonstrated an association between PFOA exposure and heightened susceptibility to infectious diseases, as well as diminished vaccine responsiveness to multiple antigens [155,156]. These findings are supported by experimental data showing reduced cytokine production, impaired lymphoproliferation in human primary cells, and altered antibody responses to T-cell-dependent antigens and leukocyte profiles in rodent models.

The immunosuppressive effect of PFOA is related to evading immune destruction, which was included in the hallmarks of cancer in our CAGE analyses. Regarding interferon signaling, the overall gene expression decreased after 24 h and 8 days of PFOA treatment.

Furthermore, in GO, GO terms related to immunity were found to be significantly depleted after 8 days of PFOA treatment and on day 21.

In the final evaluation of the carcinogenicity of PFOA and PFOS in November 2023 [2], PFOA induced oxidative stress, modulated receptor-mediated effects (via PPARα, CAR/PXR, and PPARγ), and altered cell proliferation, cell death, and nutrient and energy supply in human primary cells and experimental systems.

The European Food Safety Authority states that, in laboratory animal toxicity tests on rodents, PFOA acts as a carcinogenic promoter through PPARα in the liver, though this mechanism does not apply to humans [157]. Although our study used rodent cells, namely, Bhas 42, the CAGE analysis did not show any increase in *PPARα* gene expression at any time point of PFOA treatment.

Fatty acid beta-oxidation and mitochondrial fatty acid beta-oxidation were found to be activated at 24 h after PFOA treatment and at a later time point through transcriptome analysis. Based on GO, genes related to fatty acid beta-oxidation were significantly upregulated by 24 h of treatment with PFOA.

In its final evaluation of the carcinogenicity of PFOA and PFOS [2], the IARC identified epigenetic alterations as a key mechanistic pathway for PFOA. Multiple human studies have reported associations between maternal serum PFOA levels and gene-specific DNA methylation in offspring. A robust human-epigenome-wide association study further demonstrated that PFOA-related CpG methylation persist from birth through adolescence [151,158]. These findings are particularly relevant to carcinogenicity, as they implicate developmental epigenetic reprogramming that may modulate cancer susceptibility in humans.

In our CAGE analyses with PFOA treatment in the Bhas 42 CTA, epigenetic changes were described as a hallmark of cancer, namely, genetic instability, in the section titled “DNA Methylation and Transcriptional Repression Signaling”.

In male Sprague Dawley rats, dietary exposure to PFOA induced hepatocellular adenoma, combined hepatocellular adenoma or carcinoma, pancreatic acinar cell adenoma, and combined pancreatic acinar cell adenoma or adenocarcinoma, with a statistically significant positive trend in hepatocellular carcinoma incidence. In females, PFOA exposure led to uterine adenocarcinoma and a significant positive trend in the incidence of combined pancreatic acinar cell adenoma or adenocarcinoma [159].

As a marker related to pancreatic cancer, gene expression of *mesothelin (MSLN)* was increased in our CAGE analysis after 8 days of PFOA treatment and on day 21. *MSLN* is particularly highly expressed in several types of human malignant tumors, such as ovarian serous carcinoma, pancreatic adenocarcinoma, and malignant pleural mesothelioma [160].

Regarding the collation of the pathways of cell transformation and the hallmarks of cancer, in addition to the reports mentioned in Section 3.2.3, there are detailed reports below on the important associations between the increased genes in our comprehensive analyses and cancer.

In the PD-1/PD-L1 cancer immunotherapy pathway, CIP2A has been identified as a critical regulator, as reviewed by Soofiyani et al. [47]. CIP2A suppresses PP2A phosphatase activity and engages in a positive feedback loop with c-Myc, wherein c-Myc upregulates CIP2A expression, and CIP2A stabilizes c-Myc by preventing its S62 dephosphorylation. Additionally, CIP2A activates mTORC1 signaling, thereby further inhibiting PP2A and suppressing autophagy. CIP2A activates E2F1, Akt, and Plk1 and inhibits DAPK1, MEK, and ERK. PP2A acts as a negative regulator of MEK and ERK, which are conversely activated following PP2A inhibition by CIP2A. Furthermore, CIP2A contributes to tumor progression by promoting cell proliferation, survival, self-renewal, and resistance to senescence and apoptosis, partly through MEK-mediated activation of ERK signaling [161]. It protects cancer cells from therapy-induced apoptosis and enhances progenitor cell renewal. CIP2A also regulates cell cycle progression and mitosis, with Plk1 identified as a downstream target [162]. Notably, siRNA-mediated silencing of CIP2A has been shown to suppress the growth of xenografted tumors across multiple cancer types [163].

The role of CAFs in tumor progression was reviewed by Ermakov et al. [164]. Tumor stromal elements play a critical role in promoting cancer progression and metastasis. The stroma comprises connective tissue, vasculature, neural elements, and the ECM. Among its cellular constituents, CAFs are key regulators that synthesize collagen, the principal structural protein of the ECM. Collagen deposition induces fibrosis, increases tissue stiffness, and interferes with signaling pathways governing cell proliferation and differentiation. CAFs control tumor angiogenesis, cell motility, tumor immunogenicity.

Regarding the relationship between TOP2A-related chromosomal instability (CIN) and cancer, in addition to a report titled “Cell Cycle: G2/M DNA Damage Checkpoint Regulation”, there is also the following detailed report. Cancer cells exhibit extensive genetic alterations, including aneuploidy and CIN, the role of which—whether causal or consequential—remains debated. Most researchers view CIN as an early event in tumorigenesis that disrupts genome integrity and facilitates the loss or inactivation of tumor-suppressor genes [165,166], others suggest it arises as a byproduct of neoplastic proliferation and frequent chromosomal missegregation [167]. CIN-driven genomic alterations in subclonal populations confer selective advantages, promoting their expansion within the tumor microenvironment. As a cell-autonomous trait, CIN fosters continuous genomic and mutational plasticity, thereby enhancing cell survival, proliferation, and malignant progression [168]. By accelerating the acquisition of oncogenic loci and the loss of tumor-suppressor loci, CIN serves as a key driver of tumor evolution. Subclones lacking such advantageous alterations fail to contribute to tumor advancement, whereas successive expansions of CIN-enriched subclones underpin multistep tumor progression [169].

## 5. Conclusions

In this study, in the Bhas 42 CTA, positive results were obtained with individual treatments of PFOA and PFOS, typical NGTxCs. Therefore, among the current OECD-accredited tests, the Bhas 42 CTA is a phenomenal test method that can correctly assess the carcinogenicity of both PFOA (IARC Group 1) and PFOS (IARC Group 2B) as positive. The results of this study indicate that the Bhas 42 CTA is expected to help prevent the overlooking of NGTxC when genotoxicity is not positive in the carcinogenicity assessment of chemicals such as existing compounds like PFAS that require evaluation of carcinogenicity, as well as new compounds.

In addition, the carcinogenicity of the NGTxC PFOA is supported by the positive transformation focus formation results in the Bhas 42 CTA, as well as mechanistic data from transcriptome analyses. The mechanistic data show changes in the expression of various genes in hallmarks of cancer, thus making it possible to perform a detailed analysis over time. This is an advantage of the fact that the experiment was performed in vitro. In the time-course transcriptome analyses of PFOA treatment in the Bhas 42 CTA, in particular, after 8 days of PFOA treatment and on day 21, factors related to the promotion of significant tumor formation and enhanced malignancy were observed.

We demonstrated that the Bhas 42 CTA is a test method that can detect NGTxCs simply and can be used to search for comprehensive and delicate mechanisms in parallel with the process of transformation focus formation from the early stages to malignancy. It is necessary to accumulate and publish results using Bhas 42 CTA at many more NGTxCs, including the results of transcriptome analysis.

In this study, the results of transcriptome analysis of Bhas 42 CTA induced by PFOA showed that many GO terms associated with protein phosphorylating enzymes were increased. As a next step, we will conduct phosphoproteome analysis to elucidate a comprehensive mechanism that parallels the process of formation of focal points of malignant transformation.

## Figures and Tables

**Table 1:** Pathways for the cancer hallmark of xenobiotic metabolism.

Hallmark	Pathway	Supplementary Data (Figure)	1 h	6 h	24 h	8 Days	Day 21
Xenobiotic Metabolism	Phase 1- Functionalization of Compounds	S1, S2	—	—	CYP26B1 ↑	—	CYP3A13 ↑
	Aryl Hydrocarbon Receptor Signaling	S3, S4, S5	—	—	ALDH ↑	CHEK2 ↑, RB ↑ CDK2 ↑, Cyclin E ↑ Cyclin A ↑, ALDH ↑ Gst ↑ Tgfbeta ↓	CDK2 ↑, CHEK1 ↑ DHFR ↑, JUN ↑ MCM7 ↑, POLA1 ↑ TP53 ↑, CYP2C/3A ↑ Gst ↑, Cyclin A ↑ Cyclin E ↑, Cyclin D ↑ ALDH ↓, NQO ↓
	Xenobiotic Metabolism Signaling	S6, S7, S8, S9	—	SOD3 ↑	ALDH ↑	PI3K ↑, UGT ↑ Gst ↑ MAF ↓, MAP3K ↓ NCOA ↓	CYP3A7 ↑, RAS ↑ Gst ↑ SOD3 ↓, NQO ↓ ALDH ↓, MAO ↓

↑: up-regulated, ↓: down-regulated, —: not differentially regulated.

**Table 2:** Pathways for the cancer hallmark of evading anti-growth signaling and resisting programmed cell death.

Hallmark	Pathway	Supplementary Data (Figure)	1 h	6 h	24 h	8 Days	Day 21
Evading Anti-growth Signaling	HIPPO Signaling	S10, S11	—	—	—	Scf Trcp beta ↑	TEAD ↑ DLG ↓
	Gap Junction Signaling	S12, S13, S14	—	Connexin (GJB4) ↑ F Actin ↑	—	TNF receptor ↑ Tublin ↑ BIRC5 ↑ PI3K ↑ SKP2 ↑ TCF/LEF ↓	Connexin (GJB5) ↑ GJA1 ↑, F Actin ↑ PKG ↑, BIRC5 ↑ Tublin ↑, CCND1 ↑ RAS ↑, Caspase3/7 ↑ DLG1 ↓, Ganylate cyclase ↓ CACNA1G ↓, RUNX2 ↓ CREB ↓, TCF/LEF ↓
Resisting Programmed Cell Death	Apoptosis Signaling	S15, S16, S17, S18	Ikb ↓	calpain ↓	—	CDK1 ↑ TNFR/Fas ↑ BID ↓ TBID ↓	CASP3 ↑, Caspase 8/10 ↑ RAS ↑, TP53 ↑ CDK1 ↑, CYCS ↑ BCL2L11 ↓
	Autophagy	S19, S20	—	—	—	PI3K ↑ Tnf-receptor ↑ SLC7A5 ↑ SLC1A5 ↓	SLC7A5 ↑, ATG5 ↑ ATG9B ↑, AP1 ↑ TP53 ↑ CREB ↓, SIRT1 ↓, IRS1/2 ↓
	Microautophagy Signaling Pathway	S21, S22	—	—	Proteasome ↓	—	TOMM20 ↑ ESCRT ↑, TP53 ↑

↑: up-regulated, ↓: down-regulated, —: not differentially regulated.

**Table 3:** Pathways for the cancer hallmark of avoiding immune destruction and tumor-promoting inflammation.

Hallmark	Pathway	Supplementary Data (Figure)	1 h	6 h	24 h	8 Days	Day 21
Avoiding Immune Destruction	Immunogenic Cell Death Signaling	S23, S24, S25	—	—	CXCL10 ↓ TLR3 ↓	Tnf receptor ↑ TLR3 ↓	CASP3 ↑, CASP8 ↑ ATG12/ATG5/ATG16L1 ↑
	Interferon Signaling	S26, S27	—	—	IFIT1 ↓ IFIT3 ↓ ISG15 ↓ OAS1 ↓ STAT1 ↓ TAP1 ↓	IFI35 ↓, IFIT3 ↓ IRF9 ↓, ISG15 ↓ OAS1 ↓, STAT1 ↓ STAT2 ↓	—
	PD-1, PDL-1 cancer immunotherapy pathway	S28, S29	—	—	—	CDK2 ↑, CIP2A ↑ SKP2 ↑, PI3K ↑ Tgf receptor ↑ STAT5 ↓, MHCI-α ↓ Tgf beta ↓	CD80 ↑, CDK2 ↑ CIP2A ↑ CSK ↓, PDCD4 ↓
Tumor-Promoting Inflammation	IL-1 Signaling	S30, S31	Ikb ↓	—	—	—	JUN ↑
	Activin Inhibin Signaling Pathway	S32, S33, S34, S35, S36	NFKBIA ↓ SKIL ↓	Activin A ↑ INHBA ↑ INHIBIN A ↑ PMEPA1 ↑ SNAI1 ↑	LIPE ↑ PMEPA1 ↑ SKI ↑ TLR ↓ MXD1 ↓	Tnf receptor ↑ PI3K ↑, LIPE ↑ PMEPA1 ↑, SKIL ↑ SERPINE ↑ IL1 ↓, TLR ↓ MAF ↓, Tgf beta ↓ Beta-catenin/TCF ↓	Il1 receptor ↑ AP1 ↑, ERK ↑ FOS ↑, JUN ↑ PTGS2 ↑, IL11 ↑ Beta-catenin/TCF ↓ Collagen type I ↓
	IL-4 Signaling	S37, S38, S39	—	—	IL4R ↑	IL13RA1 ↑, PI3K ↑ MAF ↓, Tgfbeta ↓	PTPN6 ↑, RAS ↑ IRS1/2 ↓, GREB ↓
	IL-6 Signaling	S40, S41, S42, S43	Ikb ↓	SOCS3 ↑	—	PI3K ↑ Tnfreceptor ↑ IL1 ↓	RAS ↑, Il1receptor ↑ JUN ↑ COL1A1 ↓, SOCS5 ↓
	Role of JAK family kinases in IL-6-type Cytokine Signaling	S44, S45, S46, S47, S48	LIF ↓	SOCS3 ↑	STAT1 ↓	OSMR ↑, BIRC5 ↑ STAT1/5 ↓ Tgfbeta ↓, VEGF ↓	IL11 ↑, VEGF ↑ BIRC5 ↑, CCND1 ↑ LIFR ↓, SOCS5 ↓
	IL-8	S49, S50, S51	Rho ↓ ICAM1 ↓	—	—	ANGPT1 ↑, PI3K ↑ Myosinii rlc ↓, MMP9 ↓ VEGF ↓, VCAM1 ↓	CyclinD ↑, VEGF ↑ PTGS2 ↑, RAS ↑ Ap1 ↑
	TNFR2 Signaling	S52, S53, S54	Ikb ↓ TNFAIP3 ↓	—	—	TNFRSF1B ↑	TNFAIP3 ↑ JUN ↑

↑: up-regulated, ↓: down-regulated, —: not differentially regulated.

**Table 4:** Pathways for the cancer hallmark of the tumor microenvironment (TME), tissue invasion, and metastasis.

Hallmark	Pathway	Supplementary Data (Figure)	1 h	6 h	24 h	8 Days	Day 21
Tumor Microenvironment	Tumor Microenvironment	S55, S56, S57, S58	ICAM1 ↓	—	FGF ↑	FGF ↑, PI3K ↑ Tgfbeta ↓, MPP9 ↓ VEGF ↓, TNC ↓ MHCCLASS I ↓ CFLAR ↓, MMP ↓	CSF2 ↑, VEGF ↑ RAS ↑, Ap1 ↑ CCND1 ↑, PTGF2 ↑ MMP ↓, collagentype I ↓
	JAK/STAT Signaling	S59, S60, S61, S62	—	SOCS3 ↑	STAT ↓	PI3K ↑ STAT ↓	PTPN6 ↑, RAS ↑ JUN ↑ SHC1 ↓, SOCS5 ↓
Tissue Invasion and Metastasis	Glioma Invasiveness Signaling	S63, S64, S65, S66	Rho ↓	—	PLAUR ↑	HMMR ↑, PI3K ↑ TIMP ↓, MMP9 ↓	HMMR ↑, PLAUR ↑ RAS ↑
	FAK Signaling	S67, S68, S69, S70, S71	GPCR ↓	SOCS3 ↑ GPCR ↓ Integrin ↓ calpain ↓	Cytokine recepror ↑	PI3K ↑ ECM ↓ Tgf beta ↓ MMP14 ↓ MMP9 ↓ TCF/LEF ↓	ECM ↑, EPH ↑ RAS ↑, AP1 ↑ CCND1 ↑, TP53 ↑ ETV4 ↑ Collagen type I ↓, SOCK ↓ CSK ↓, TCF/LEF ↓
	CSDE1 Signaling Pathway	S72, S73, S74	—	SNAI1 ↑ PABP ↓	—	TNC ↓	PTBP1 ↑ HNRNPC ↑

↑: up-regulated, ↓: down-regulated, —: not differentially regulated.

**Table 5:** Pathways for the cancer hallmark of sustained growth signaling.

Hallmark	Pathway	Supplementary Data (Figure)	1 h	6 h	24 h	8 Days	Day 21
Sustained Growth Signaling	Cyclins and Cell Cycle Regulation	S75, S76	—	—	—	CDK2 ↑, CyclinE ↑ E2F ↑, CDK1 ↑ CyclinA ↑, CyclinB ↑ SCF ↑ Tgf beta ↓, HDAC ↓	Cyclin D ↑, CDK2 ↑ Cyclin E ↑, TP53 ↑ E2F ↑, CDK1 ↑ Cyclin A ↑, Cyclin B ↑
	PAK Signaling	S77, S78, S79		Integrin ↓	—	PI3K ↑ MLC ↓	RAS ↑, CASP3 ↑ COFLIN ↑, ETK1/2 ↓
	HOTAIR Regulatory Signaling	S80, S81, S82	NFKBIA ↓ ICAM1 ↓	—	—	PI3K ↑, FOXM1 ↑ Wnt ↑ PRC2 ↓, Mmp ↓ TCF/LEF ↓	MIR130A ↑, FOXM1 ↑ RBM38 ↑ Collagen type I ↓ Mmp ↓, TCF/LEF ↓
	EGF Signaling	S83, S84, S85	—	—	STAT1 ↓	PI3K ↑, STAT1 ↓	JUN ↑
	FGF Signaling	S86, S87, S88	—	—	FGF ↑	FGF ↑, PI3K ↑	PTPN6 ↑ CREB ↓
	IGF Signaling	S89, S90, S91	—	SOCS3 ↑	—	PI3K ↑	RAS ↑, JUN ↑ IGFBP ↓, IRIS1/2 ↓ SOCS5 ↓
	TGF-β Signaling	S92, S93, S94, S95	—	Activins /Inhibins ↑ PMEPA1 ↑	SKI ↑ PMEPA1 ↑ IRF7 ↓	PMEPA1 ↑ SERPIN1 ↑ Tgf beta ↓, IRF7 ↓	RAS ↑, Ap1 ↑ JUN ↑, SMAD6 ↓ VDR ↓, RUNX2 ↓
	VEGF Signaling	S96, S97, S98	—	ACTIN ↑	—	PI3K ↑ Vegf ↓, VEGF ↓ VEGFC/D ↓, VCL ↓	Vegf ↑, VEGF ↑ EIF ↑, RAS ↑ SHP ↑, ACTIN ↑
	mTOR Signaling	S99, S100, S101	RHO ↓	—	—	PI3K ↑ VEGF ↓	RAS ↑, VEGF ↑ Ribosomal 40s subunit ↑ IRS1 ↓
	PI3K-AKT Signaling	S102, S103, S104, S105, S106	Ikb ↓	Integrin ↓	Cytokine receptor ↑	MAP3K8 ↓	RAS ↑, PTGS2 ↑ TP53 ↑, CCND1 ↑ PI3Kp85 ↓
	WNT/β-catenin Signaling	S107, S108	—	—	—	Wnt ↑ Tgf beta ↓ TCF/LEF ↓, TCF4 ↓	TP53 ↑, RUVBL2 ↑ JUN ↑, CCND1 ↑ GJA1 ↑ Frizzied ↓, TCF/LEF ↓

↑: up-regulated, ↓: down-regulated, —: not differentially regulated.

**Table 6:** Pathways for the cancer hallmark of genetic instability.

Hallmark	Pathway	Supplementary Data (Figure)	1 h	6 h	24 h	8 Days	Day 21
Genetic Instability	ATM Signaling	S109, S110, S111	NFKBIA ↓	—	—	MDC1 ↑, CHEK2 ↑ CDK1 ↑, CDK2 ↑ Cyclin B ↑, FANCD2 ↑ SMC ↑, H2AX ↑ TOPBP1 ↑ BID ↓	MDC1 ↑, CHEK2 ↑ CDK1 ↑, CDK2 ↑ TP53 ↑, GADD45 ↑ Cyclin B ↑, BRCA1 ↑ CHEK1 ↑, FANCD2 ↑ RAD5 ↑, BLM ↑ SMC ↑, RAD50 ↑ MRE11 ↑, H2AX ↑ TOPBP1 ↑, SUV39H1 ↑ CREB ↓
	BRCA1 in DNA Damage Response	S112, S113, S114	—	—	STAT1 ↓	MDC1 ↑, CHEK2 ↑ BRCA1 complex B ↑ MDC1 ↑, E2F ↑ RB ↑, PLK1 ↑ Swi-Snf ↑ FANCD2 ↑ BRCA2 ↑	MDC1 ↑, BRCA1 ↑ BARD1 ↑ BRCA1 complex A ↑ BRCA1 complex B ↑ BRCA1 complex C ↑ BRIP1 ↑, BLM ↑ TOPBP1 ↑, RAD50 ↑ MRE11 ↑, MDC1 ↑ E2F ↑, CHEK1 ↑ PLK1 ↑, P53 ↑ Swi-Snf ↑, FANCD2 ↑ BARD1↑, RAD51 ↑
	Role of CHK Proteins in Cell Cycle Checkpoint Control	S115, S116	—	—	—	MDC1 ↑, RFC ↑ CHEK2 ↑, CDK2 ↑ PLK1 ↑, CLSPN ↑ CDK1 ↑, E2F ↑	RFC ↑, MDC1 ↑ CLPSN ↑, BRCA1 ↑ CDK1 ↑, RAD50 ↑ MRE11 ↑, CHEK1 ↑ TP53 ↑, CDK2 ↑ PCNA ↑, E2F ↑
	Cell cycle: G2/M DNA Damage Checkpoint Regulation	S117, S118	—	—	—	CHK2 ↑, TOP2 ↑ BORA ↑, AURKA ↑ PLK1 ↑, PKMYT1 ↑ CDK1 ↑, CyclinB ↑ SCF ↑	BRCA1 ↑, CHK1 ↑ TOP2 ↑, BORA ↑ AURKA ↑, PLK1 ↑ PKMYT1 ↑, CDK1 ↑ CyclinB ↑, CKS1B ↑ CKS2 ↑, TP53 ↑
	DNA Methylation and Transcriptional Repression Signaling	S119, S120	—	—	—	CDK ↑, RB ↑ E2F ↑, SAP30 ↑ Transcription factor ↑	GAD45 ↑, TP53 ↑ CDK ↑, E2F ↑ DNMT1 ↑, UHRF1 ↑ Transcription factor ↑ SUV39H1 ↑
	Mismatch Repair in Eukaryotes	S121, S122	—	—	—	RFC ↑ POLD1 ↑ MutLa-MutSa-Exo1-Pold-RFC-RPA ↑	RFC ↑, PCNA ↑ RPA1 ↑, EXO1 ↑ POLD1 ↑, LIG ↑ MutLa-MutSa-Exo1-Pold-RFC-RPA ↑
	Mitochondrial Dysfunction	S123, S124, S125, S126	—	Calpain ↓	BBC3 ↓	OXPHOS ↑ Glutation peroxidase ↑ ACADL ↑ Cytochrome-c oxidase ↑ F0 ATP synthase ↑ PPARGC1A ↓ BID ↓	Casp3 ↑, CYCS ↑ TOM ↑, MCU ↑ TP53 ↑, UCP2 ↑ VDCC ↓, SIRT1 ↓ CREB ↓

↑: up-regulated, ↓: down-regulated, —: not differentially regulated.

**Table 7:** Pathways for the cancer hallmark of enabled replication immortality and inducing new blood flow.

Hallmark	Pathway	Supplementary Data (Figure)	1 h	6 h	24 h	8 Days	Day 21
Enabled Replication Immortality	Senescence Pathway	S127, S128, S129	—	—	PI3K ↑	PI3K ↑, FZR1 ↑ Rb1 ↑, E2F ↑ CDK2-Cyclin E ↑ DHCR24 ↑ CHEK2 ↑ SEPRINE1 ↑ Cyclin-b-Cdc2 ↑ Tgf beta ↓	RAS ↑, ERK ↑ JUN ↑, DHCR24 ↑ CHEK1 ↑, TP53 ↑ GADD45 ↑ Cyclin-b-Cdc2 ↑ E2F ↑, CDK4/6-CyclinD1 ↑ CDK1-CyclinB ↑ MCU ↑, Smad ↓ SIRT1 ↓
	Senescence-Associated Secretory Phenotype (SASP)	S130, S131, S132	—	—	IL6 gene:Nucleosome H3K9Me2 ↓ IL6 gene:Nucleosome ↓ IL8 gene:Nucleosome H3K9Me2 ↓ IL8 gene:Nucleosome ↓	Cyclin A:phospho-Cdk(Thr 160):Cdh1:phosho-APC/C complex ↑ CyclinA:Cdk2:p21/p27complex ↑ CCNA:p-T160-CDK2 Cdh1:phospho-APC/C complex ↑ EHMT1:EHMT2:Cdh1:p-APC/C ↑ Ub-EHMT1:Ub-EHMT2:Cdh1:p-APC/C ↑ IL6 gene:Nucleosome H3K9Me2 ↑ IL6 gene:Nucleosome ↑ IL8 gene:Nucleosome H3K9Me2 ↑ IL8 gene:Nucleosome ↑	Cyclin A:phospho-Cdk(Thr 160):Cdh1:phosho-APC/C complex ↑ CyclinA:Cdk2:p21/p27complex ↑ CCNA:p-T160-CDK2 Cdh1:phospho-APC/C complex ↑ EHMT1:EHMT2:Cdh1:p-APC/C ↑ Ub-EHMT1:Ub-EHMT2:Cdh1:p-APC/C ↑ IL6 gene:Nucleosome H3K9Me2 ↑ IL6 gene:Nucleosome ↑ IL8 gene:Nucleosome H3K9Me2 ↑ IL8 gene:Nucleosome ↑ p-2S-cJUN:p-2S,2T-cFOS ↑ p-2S-JUN:p-2S,2T-FOS:IL1A gene IGFBP7 gene ↓
	Telomerase	S133, S134	—	—	—	PI3K ↑ HDAC ↓	RAS ↑, TP53 ↑ DKC1 ↑
	Telomere Extension by Telomerase	S135	—	—	—	—	MRE11 ↑, RAD50 ↑ Ku ↑
	TGF-β Signaling	S92, S93, S94, S95	—	Activins /Inhibins ↑ PMEPA1 ↑	SKI ↑ PMEPA1 ↑ IRF7 ↓	PMEPA1 ↑ SERPIN1 ↑ Tgf beta ↓, IRF7 ↓	RAS ↑, Ap1 ↑ JUN ↑, SMAD6 ↓ VDR ↓, RUNX2 ↓
	WNT/β-catenin Signaling	S107, S108	—	—	—	Wnt ↑ Tgf beta ↓ TCF/LEF ↓, TCF4 ↓	TP53 ↑, RUVBL2 ↑ JUN ↑, CCND1 ↑ GJA1 ↑ Frizzied ↓, TCF/LEF ↓
Inducing New Blood Flow	Angiopoietin Signaling	S136, S137, S138	Ikb ↓	—	—	ANGPT1 ↑ BIRC5 ↑, STAT5 ↓	RAS ↑, BIRC5 ↑ PI3Kp85 ↓
	VEGF Signaling	S96, S97, S98	—	ACTIN ↑	—	PI3K ↑ Vegf ↓, VEGF ↓ VEGFC/D ↓, VCL ↓	Vegf ↑, VEGF ↑ EIF ↑, RAS ↑ SHP ↑, ACTIN ↑

↑: up-regulated, ↓: down-regulated, —: not differentially regulated.

**Table 8:** Pathways for the cancer hallmark of deregulated cellular metabolism.

Hallmark	Pathway	Supplementary Data (Figure)	1 h	6 h	24 h	8 Days	Day 21
Deregulated Cellular Metabolism	Fatty acid β-oxidation I	S139, S140, S141	—	—	dodecanoyl-CoA D-isomerase ↑ Acetyl-CoA C-acyltransferase ↑	long-chain-fatty-acid-CoA ligase ↑ Acetyl-CoA C-acyltransferase ↑	long-chain-fatty-acid-CoA ligase ↑
	Mitochondrial fatty acid beta oxidation	S142, S143, S144	—	—	ACCA2 tetramer ↑	ACCA2 tetramer ↑ ACADL tetramer ↑ DECR1 tetramer ↑	ACHOT2,9, THEM4,5 dimer ↑
	PPAR signaling	S145, S146, S147	Ikb ↓	—	—	Tnf receptor ↑ IL1 ↓, NRIH3 ↓ STAT5 ↓, NCOA ↓	RAS ↑, Il1 receptor ↑ JUN ↑, PTGS2 ↑
	AMPK signaling	S148, S149, S150, S151	—	Adenylate kinase ↑ CPT1 ↑	LIPE ↑ CPT1 ↑	PI3K ↑, PFK ↑ CPT1 ↑, CCNA2 ↑ LIPE ↑, HMGCR ↑ Swi-Snf ↑ PP2C ↓, PPARGC1A ↓	Nicotinic acetylcholine receptor ↑ Adenylate kinase ↑ PPAT ↑, CCNA2 ↑ CCND1 ↑, HMGCR ↑ IRS1/2 ↓
	Superpathway of Cholesterol Biosynthesis	S152, S153, S154	—	—	acetyl-CoA C-acyltransferase ↑	acetyl-CoA C-acyltransferase ↑ hydroximethylglutaryl-CoA synthase ↑ hydroximethylglutaryl-CoA reductase ↑ farnecyl-diphosphate farnecyltransferase ↑ squalene monooxygenase ↑ D24-sterol reductase ↑	acetyl-CoA C-acyltransferase ↑ hydroximethylglutaryl-CoA synthase ↑ hydroximethylglutaryl-CoA reductase ↑ diphosphomevalonate ↑ decarboxylase isopentenyl-diphosphate D-isomerase ↑ farnesyl-diphosphate farnesyltransferase ↑ D24-sterol reductase ↑ methylsterol monooxygenase ↑ 3beta-hydroxy-4alpha-methylcholestenecarboxylate ↑ 3-dehydrogenase (decarboxylating) ↑
	Folate signaling pathway	S155, S156, S157	—	—	OAS2 ↓	SHMT2 ↑ ALDH1L2 ↑ TYMS ↑, CCNA2 ↑ OAS2 ↓	SHMT2 ↑, SHMT1 ↑ MTHFD1L ↑, DHFR ↑ TYMS↑, Purino-some ↑ TP53↑, CCNA2 ↑

↑: up-regulated, ↓: down-regulated, —: not differentially regulated.

## Data Availability

The datasets presented in this article are not readily available because the data are part of an ongoing study. Requests to access the datasets should be directed to author.

## Supplementary Materials

The following supporting information can be downloaded at: https://www.mdpi.com/article/10.3390/biom15101431/s1, Table S1: Gene Ontology terms for selected upregulated genes; Table S2: Gene Ontology terms for selected downregulated genes; Figures S1–S9: Xenobiotic Metabolism; Figures S10–S14: Evading Antigrowth Signaling; Figures S15–S22: Resisting Programmed Cell Death; Figures S23–S29: Avoiding Immune Destruction; Figures S30–S54: Tumor-Promoting Inflammation; Figures S55–S62: Tumor Microenvironment; Figures S63–S74: Tissue Invasion and Metastasis; Figures S65–S108: Sustained Growth Signaling; Figures S109–S126: Genetic Instability; Figures S92–S95, S107, S108, S127–S135: Enabled Replication Immortality; Figures S96–S98, S136–S138: Inducing New Blood Flow; Figures S139–S157: Deregulated Cellular Metabolism.
