# Supplementary material for: Oncotransformation in Bhas 42 Cell Transformation Assay by Typical Non-Genotoxic Carcinogens, PFOA and PFOS, and Time-Course Transcriptome Analysis
Source: Biomolecules. 2025 Oct 9;15(10):1431. doi: 10.3390/biom15101431 (PMC12562413; doi:10.3390/biom15101431)
Supplement: Supplementary file 1 [file biomolecules-15-01431-s001.zip › biomolecules-3848492-supplementary.pdf]

Oncotransformation in Bhas 42 Cell Transformation Assay by Typical Non-Genotoxic Carcinogens, PFOA and PFOS, and Time-Course Transcriptome Analysis

Kiyomi Ohmori [1, 2]*

Chemical Division, Kanagawa Prefectural Institute of Public Health, Chigasaki 2530087, Japan
Institute of Advanced Science, Yokohama National University, Yokohama 2408501, Japan
Correspondence: ohmori.n4yf@pref.kanagawa.lg.jp or ohmori-kiyomi-kz@ynu.ac.jp; Tel./Fax: +81-46-783-4400 or +81-45-339-4448

Supplemental Materials

Table No.	page
S 1	2-5
S 2	6-8

Figure No.	page
S 1	9
S 2	10
S 3	11
S 4	11
S 5	12
S 6	13
S 7	14
S 8	15
S 9	16
S 10	17
S 11	18
S 12	19
S 13	20
S 14	21
S 15	22
S 16	23
S 17	24
S 18	25
S 19	26
S 20	27
S 21	28
S 22	29
S 23	30
S 24	31
S 25	32
S 26	33
S 27	34
S 28	35
S 29	36
S 30	37
S 31	38
S 32	39
S 33	40
S 34	41
S 35	42
S 36	43
S 37	44
S 38	45
S 39	46
S 40	47

Figure No.	page
S 41	48
S 42	49
S 43	50
S 44	51
S 45	52
S 46	53
S 47	54
S 48	55
S 49	56
S 50	57
S 51	58
S 52	59
S 53	60
S 54	61
S 55	62
S 56	63
S 57	64
S 58	65
S 59	66
S 60	67
S 61	68
S 62	69
S 63	70
S 64	71
S 65	72
S 66	73
S 67	74
S 68	75
S 69	76
S 70	77
S 71	78
S 72	79
S 73	80
S 74	81
S 75	82
S 76	83
S 77	84
S 78	85
S 79	86
S 80	87

Figure No.	page
S 81	88
S 82	89
S 83	90
S 84	91
S 85	92
S 86	93
S 87	94
S 88	95
S 89	96
S 90	97
S 91	98
S 92	99
S 93	100
S 94	101
S 95	102
S 96	103
S 97	104
S 98	105
S 99	106
S 100	107
S 101	108
S 102	109
S 103	110
S 104	111
S 105	112
S 106	113
S 107	114
S 108	115
S 109	116
S 110	117
S 111	118
S 112	119
S 113	120
S 114	121
S 115	122
S 116	123
S 117	124
S 118	125
S 119	126
S 120	127

Figure No.	page
S 121	128
S 122	129
S 123	130
S 124	131
S 125	132
S 126	133
S 127	134
S 128	135
S 129	136
S 130	137
S 131	138
S 132	139
S 133	140
S 134	141
S 135	142
S 136	143
S 137	144
S 138	145
S 139	146
S 140	147
S 141	148
S 142	149
S 143	150
S 144	151
S 145	152
S 146	153
S 147	154
S 148	155
S 149	156
S 150	157
S 151	158
S 152	159
S 153	160
S 154	161
S 155	162
S 156	163
S 157	164

Table S1

Gene Ontology terms for selected upregulated genes. These GO terms are enriched due to PFOA treatment for 1 or 24 hours. Shown in red are the GO terms at the bottom of the hierarchical tree output created using the Explore Biology feature in QuickGO.

	ID	Gene Ontology term	FDR-corrected q-value	Ontology
1 hour	GO:0000083	regulation of transcription involved in G1/S transition of mitotic cell cycle	8.63E-03	BP
	**GO:2000352**	**negative regulation of endothelial cell apoptotic process**	8.63E-03	BP
	GO:0055090	acylglycerol homeostasis	8.63E-03	BP
	**GO:0070328**	**triglyceride homeostasis**	8.63E-03	BP
	GO:2000351	regulation of endothelial cell apoptotic process	8.82E-03	BP
	GO:1904036	negative regulation of epithelial cell apoptotic process	8.82E-03	BP
	GO:0072577	endothelial cell apoptotic process	8.82E-03	BP
	**GO:0060191**	**regulation of lipase activity**	9.98E-03	BP
6 hours	NONE			
24 hours	GO:0072329	monocarboxylic acid catabolic process	7.28E-04	BP
	GO:0016042	lipid catabolic process	1.65E-03	BP
	GO:0009062	fatty acid catabolic process	1.65E-03	BP
	GO:0019395	fatty acid oxidation	1.65E-03	BP
	GO:0046395	carboxylic acid catabolic process	1.65E-03	BP
	GO:0016054	organic acid catabolic process	1.65E-03	BP
	GO:0034440	lipid oxidation	1.65E-03	BP
	GO:0044242	cellular lipid catabolic process	1.65E-03	BP
	GO:0044282	small molecule catabolic process	1.65E-03	BP
	**GO:0006635**	**fatty acid beta-oxidation**	6.39E-03	BP
	**GO:0060349**	**bone morphogenesis**	8.50E-03	BP
	**GO:0015101**	**organic cation transmembrane transporter activity**	3.52E-03	MF

BP: biological process; MF: molecular function; CC: cellular component

Table S1 (continued). Gene Ontology terms for selected upregulated genes. These GO terms are enriched following PFOA treatment for 8 days. Shown in red are the GO terms at the bottom of the hierarchical tree output created using the Explore Biology feature in QuickGO.

	ID	Gene Ontology term	FDR-corrected q-value	Ontology
8 days	GO:0000280	nuclear division	1.83E-39	BP
	GO:0048285	organelle fission	5.97E-37	BP
	GO:0007059	chromosome segregation	1.62E-36	BP
	GO:0098813	nuclear chromosome segregation	8.60E-34	BP
	GO:0140014	mitotic nuclear division	1.80E-30	BP
	GO:0000819	sister chromatid segregation	4.72E-25	BP
	GO:0000070	mitotic sister chromatid segregation	3.73E-24	BP
	GO:0051321	meiotic cell cycle	2.68E-23	BP
	GO:1903046	meiotic cell cycle process	3.76E-22	BP
	GO:0140013	meiotic nuclear division	1.30E-21	BP
	GO:0090068	positive regulation of cell cycle process	8.65E-17	BP
	GO:0044770	cell cycle phase transition	1.33E-15	BP
	GO:0044772	mitotic cell cycle phase transition	2.72E-15	BP
	GO:0007127	meiosis I	1.39E-14	BP
	GO:0061982	meiosis I cell cycle process	3.21E-14	BP
	GO:0000226	microtubule cytoskeleton organization	3.92E-14	BP
	GO:0051983	regulation of chromosome segregation	8.85E-14	BP
	GO:0045787	positive regulation of cell cycle	1.30E-13	BP
	GO:0007051	spindle organization	1.98E-13	BP
	GO:0051304	chromosome separation	2.20E-13	BP
	GO:0045132	meiotic chromosome segregation	1.28E-12	BP
	GO:1902850	microtubule cytoskeleton organization involved in mitosis	2.41E-12	BP
	GO:1905818	regulation of chromosome separation	2.45E-12	BP
	GO:0044839	cell cycle G2/M phase transition	1.62E-11	BP
	GO:0007052	mitotic spindle organization	2.54E-11	BP
	GO:0007346	regulation of mitotic cell cycle	2.61E-11	BP
	GO:0051783	regulation of nuclear division	4.59E-11	BP
	GO:0000075	cell cycle checkpoint	2.08E-10	BP
	GO:0010948	negative regulation of cell cycle process	2.26E-10	BP
	GO:0006260	DNA replication	5.58E-10	BP
	GO:1901987	regulation of cell cycle phase transition	5.69E-10	BP
	GO:0000086	G2/M transition of mitotic cell cycle	6.41E-10	BP
	GO:0006281	DNA repair	1.45E-09	BP
	GO:0007088	regulation of mitotic nuclear division	1.94E-09	BP
	GO:0010965	regulation of mitotic sister chromatid separation	1.94E-09	BP
	GO:0007093	mitotic cell cycle checkpoint	2.13E-09	BP
	GO:0031577	spindle checkpoint	2.95E-09	BP
	GO:0032465	regulation of cytokinesis	3.11E-09	BP
	GO:0051306	mitotic sister chromatid separation	3.89E-09	BP
	GO:0008608	attachment of spindle microtubules to kinetochore	5.48E-09	BP
	GO:0033045	regulation of sister chromatid segregation	6.16E-09	BP
	GO:1901990	regulation of mitotic cell cycle phase transition	6.36E-09	BP
	GO:0045786	negative regulation of cell cycle	8.66E-09	BP
	GO:0033044	regulation of chromosome organization	8.66E-09	BP
	GO:0071174	mitotic spindle checkpoint	1.46E-08	BP
	GO:0007091	metaphase/anaphase transition of mitotic cell cycle	1.46E-08	BP
	GO:0044784	metaphase/anaphase transition of cell cycle	1.85E-08	BP
	GO:0045143	homologous chromosome segregation	1.86E-08	BP
	**GO:1901988**	**negative regulation of cell cycle phase transition**	1.86E-08	BP
	GO:0051302	regulation of cell division	2.35E-08	BP
	GO:0051985	negative regulation of chromosome segregation	3.50E-08	BP
	GO:0006261	DNA-dependent DNA replication	8.70E-08	BP
	GO:0000910	cytokinesis	9.70E-08	BP
	GO:0033046	negative regulation of sister chromatid segregation	2.55E-07	BP
	GO:0033048	negative regulation of mitotic sister chromatid segregation	2.55E-07	BP
	GO:0070192	chromosome organization involved in meiotic cell cycle	2.55E-07	BP
	GO:2000816	negative regulation of mitotic sister chromatid separation	2.55E-07	BP
	GO:0051225	spindle assembly	2.57E-07	BP
	GO:0051303	establishment of chromosome localization	2.67E-07	BP
	GO:1905819	negative regulation of chromosome separation	3.25E-07	BP
	GO:0051310	metaphase plate congression	3.26E-07	BP
	GO:0050000	chromosome localization	3.74E-07	BP
	GO:1902749	regulation of cell cycle G2/M phase transition	5.14E-07	BP
	GO:0032467	positive regulation of cytokinesis	5.14E-07	BP
	GO:0033047	regulation of mitotic sister chromatid segregation	5.29E-07	BP
	**GO:0000281**	**mitotic cytokinesis**	5.66E-07	BP
	GO:0030071	regulation of mitotic metaphase/anaphase transition	6.67E-07	BP
	GO:0071459	protein localization to chromosome, centromeric region	7.22E-07	BP
	GO:0045930	negative regulation of mitotic cell cycle	7.34E-07	BP
	GO:0010389	regulation of G2/M transition of mitotic cell cycle	7.51E-07	BP
	GO:1902099	regulation of metaphase/anaphase transition of cell cycle	7.89E-07	BP
	GO:0045839	negative regulation of mitotic nuclear division	8.14E-07	BP
	GO:0007062	sister chromatid cohesion	1.04E-06	BP
	**GO:0007094**	**mitotic spindle assembly checkpoint**	1.15E-06	BP
	GO:0071173	spindle assembly checkpoint	1.15E-06	BP
	GO:0090329	regulation of DNA-dependent DNA replication	1.28E-06	BP
	GO:0051784	negative regulation of nuclear division	1.61E-06	BP
	GO:0061640	cytoskeleton-dependent cytokinesis	1.70E-06	BP
	GO:0045841	negative regulation of mitotic metaphase/anaphase transition	1.93E-06	BP
	GO:0006275	regulation of DNA replication	2.04E-06	BP
	GO:1902100	negative regulation of metaphase/anaphase transition of cell cycle	2.47E-06	BP
	GO:0007143	female meiotic nuclear division	2.49E-06	BP
	GO:0000212	meiotic spindle organization	3.60E-06	BP
	GO:0034502	protein localization to chromosome	6.11E-06	BP
	GO:1901991	negative regulation of mitotic cell cycle phase transition	6.11E-06	BP
	GO:0007098	centrosome cycle	6.95E-06	BP
	GO:0006310	DNA recombination	8.81E-06	BP
	GO:2001251	negative regulation of chromosome organization	1.50E-05	BP
	GO:0065004	protein-DNA complex assembly	1.82E-05	BP
	**GO:0034501**	**protein localization to kinetochore**	2.02E-05	BP
	GO:1903083	protein localization to condensed chromosome	2.02E-05	BP
	GO:0007292	female gamete generation	2.09E-05	BP
	GO:0031023	microtubule organizing center organization	2.64E-05	BP
	**GO:0090307**	**mitotic spindle assembly**	3.11E-05	BP
	GO:0051988	regulation of attachment of spindle microtubules to kinetochore	3.11E-05	BP
	GO:0034508	centromere complex assembly	3.15E-05	BP
	GO:1901989	positive regulation of cell cycle phase transition	3.61E-05	BP
	GO:0051383	kinetochore organization	3.93E-05	BP
	GO:0051984	positive regulation of chromosome segregation	5.47E-05	BP
	GO:0051347	positive regulation of transferase activity	6.85E-05	BP

	ID	Gene Ontology term	FDR-corrected q-value	Ontology
8 days	**GO:0007131**	**reciprocal meiotic recombination**	8.42E-05	BP
continue	GO:0090306	spindle assembly involved in meiosis	9.57E-05	BP
	GO:0007080	mitotic metaphase plate congression	1.03E-04	BP
	GO:0035825	homologous recombination	1.03E-04	BP
	GO:0006302	double-strand break repair	1.12E-04	BP
	**GO:0030261**	**chromosome condensation**	1.27E-04	BP
	GO:0009394	2'-deoxyribonucleotide metabolic process	1.56E-04	BP
	GO:0019692	deoxyribose phosphate metabolic process	1.56E-04	BP
	GO:0042770	signal transduction in response to DNA damage	1.69E-04	BP
	**GO:0032506**	**cytokinetic process**	1.89E-04	BP
	GO:0007276	gamete generation	2.21E-04	BP
	GO:0009262	deoxyribonucleotide metabolic process	2.28E-04	BP
	**GO:0007100**	**mitotic centrosome separation**	2.59E-04	BP
	GO:0071824	protein-DNA complex subunit organization	2.62E-04	BP
	GO:0031570	DNA integrity checkpoint	2.81E-04	BP
	**GO:1904666**	**regulation of ubiquitin protein ligase activity**	2.86E-04	BP
	GO:0051781	positive regulation of cell division	3.08E-04	BP
	GO:0051299	centrosome separation	3.94E-04	BP
	GO:1900262	regulation of DNA-directed DNA polymerase activity	5.90E-04	BP
	**GO:1900264**	**positive regulation of DNA-directed DNA polymerase activity**	5.90E-04	BP
	GO:0009162	deoxyribonucleoside monophosphate metabolic process	6.64E-04	BP
	**GO:0000076**	**DNA replication checkpoint**	8.50E-04	BP
	GO:0044282	small molecule catabolic process	9.71E-04	BP
	GO:0044818	mitotic G2/M transition checkpoint	1.01E-03	BP
	**GO:0000724**	**double-strand break repair via homologous recombination**	1.01E-03	BP
	**GO:0007095**	**mitotic G2 DNA damage checkpoint**	1.07E-03	BP
	GO:0044774	mitotic DNA integrity checkpoint	1.07E-03	BP
	GO:0000725	recombinational repair	1.15E-03	BP
	GO:0048609	multicellular organismal reproductive process	1.36E-03	BP
	GO:0032504	multicellular organism reproduction	1.50E-03	BP
	**GO:0051382**	**kinetochore assembly**	1.56E-03	BP
	GO:0019953	sexual reproduction	1.95E-03	BP
	**GO:0009219**	**pyrimidine deoxyribonucleotide metabolic process**	2.08E-03	BP
	**GO:0007144**	**female meiosis I**	2.17E-03	BP
	GO:0034440	lipid oxidation	2.35E-03	BP
	**GO:0051307**	**meiotic chromosome separation**	2.69E-03	BP
	GO:0000077	DNA damage checkpoint	2.82E-03	BP
	GO:0010972	negative regulation of G2/M transition of mitotic cell cycle	2.83E-03	BP
	GO:0046395	carboxylic acid catabolic process	3.12E-03	BP
	GO:0016054	organic acid catabolic process	3.32E-03	BP
	**GO:1901992**	**positive regulation of mitotic cell cycle phase transition**	3.32E-03	BP
	**GO:0007129**	**synapsis**	3.36E-03	BP
	GO:0044773	mitotic DNA damage checkpoint	3.38E-03	BP
	**GO:0006270**	**DNA replication initiation**	3.38E-03	BP
	GO:1902750	negative regulation of cell cycle G2/M phase transition	3.47E-03	BP
	**GO:0006635**	**fatty acid beta-oxidation**	3.89E-03	BP
	GO:0010639	negative regulation of organelle organization	4.54E-03	BP
	**GO:0051653**	**spindle localization**	4.61E-03	BP
	GO:0006220	pyrimidine nucleotide metabolic process	4.68E-03	BP
	GO:0009062	fatty acid catabolic process	5.37E-03	BP
	**GO:1905820**	**positive regulation of chromosome separation**	6.41E-03	BP
	GO:0051438	regulation of ubiquitin-protein transferase activity	6.71E-03	BP
	GO:0045931	positive regulation of mitotic cell cycle	7.14E-03	BP
	**GO:0022412**	**cellular process involved in reproduction in multicellular organism**	7.45E-03	BP
	GO:0051656	establishment of organelle localization	7.50E-03	BP
	GO:0019395	fatty acid oxidation	7.54E-03	BP
	GO:0007064	mitotic sister chromatid cohesion	7.61E-03	BP
	GO:0071897	DNA biosynthetic process	8.30E-03	BP
	GO:0051052	regulation of DNA metabolic process	8.30E-03	BP
	**GO:0006334**	**nucleosome assembly**	8.62E-03	BP
	**GO:0045859**	**regulation of protein kinase activity**	8.77E-03	BP
	**GO:0008017**	**microtubule binding**	5.69E-07	MF
	GO:0015631	tubulin binding	1.20E-05	MF
	GO:0003777	microtubule motor activity	1.20E-05	MF
	GO:0008094	DNA-dependent ATPase activity	1.20E-05	MF
	GO:0140097	catalytic activity, acting on DNA	1.70E-05	MF
	GO:0003774	motor activity	4.69E-04	MF
	**GO:0008574**	**ATP-dependent microtubule motor activity, plus-end-directed**	1.63E-03	MF
	**GO:0017116**	**single-stranded DNA helicase activity**	1.64E-03	MF
	GO:0003678	DNA helicase activity	4.08E-03	MF
	**GO:0017056**	**structural constituent of nuclear pore**	5.90E-03	MF

BP: biological process; MF: molecular function

Table S1 (continued). Gene Ontology terms for selected upregulated genes. These GO terms are enriched following PFOA treatment on Day 21. Shown in red are the GO terms at the bottom of the hierarchical tree output created using the Explore Biology feature in QuickGO.

	ID	Gene Ontology term	FDR-corrected q-value	Ontology
day 21	GO:0007059	chromosome segregation	4.39E-48	BP
	GO:0000280	nuclear division	9.08E-38	BP
	GO:0098813	nuclear chromosome segregation	3.98E-37	BP
	GO:0006261	DNA-dependent DNA replication	2.16E-36	BP
	GO:0006260	DNA replication	1.05E-35	BP
	GO:0048285	organelle fission	1.23E-35	BP
	GO:0000819	sister chromatid segregation	2.92E-33	BP
	GO:0140014	mitotic nuclear division	1.54E-30	BP
	GO:0000070	mitotic sister chromatid segregation	5.01E-30	BP
	GO:0006281	DNA repair	9.38E-25	BP
	GO:0051321	meiotic cell cycle	3.98E-22	BP
	GO:1903046	meiotic cell cycle process	4.81E-22	BP
	GO:0140013	meiotic nuclear division	1.78E-21	BP
	GO:0065004	protein-DNA complex assembly	4.09E-19	BP
	**GO:0006270**	**DNA replication initiation**	**1.91E-18**	**BP**
	GO:0051983	regulation of chromosome segregation	3.74E-18	BP
	GO:0006310	DNA recombination	1.18E-16	BP
	**GO:0006334**	**nucleosome assembly**	**2.39E-16**	**BP**
	GO:0071824	protein-DNA complex subunit organization	5.74E-16	BP
	GO:0044770	cell cycle phase transition	1.20E-14	BP
	GO:0007093	mitotic cell cycle checkpoint	1.20E-14	BP
	GO:0000075	cell cycle checkpoint	1.46E-14	BP
	GO:0051304	chromosome separation	1.46E-14	BP
	GO:0007127	meiosis I	1.71E-14	BP
	GO:0007051	spindle organization	4.28E-14	BP
	GO:0061982	meiosis I cell cycle process	5.27E-14	BP
	GO:1905818	regulation of chromosome separation	5.58E-14	BP
	GO:0033044	regulation of chromosome organization	1.13E-13	BP
	GO:0006302	double-strand break repair	1.26E-13	BP
	GO:0044772	mitotic cell cycle phase transition	1.42E-13	BP
	GO:0010948	negative regulation of cell cycle process	3.98E-13	BP
	GO:0044786	cell cycle DNA replication	6.42E-13	BP
	GO:0090068	positive regulation of cell cycle process	8.51E-13	BP
	GO:0034728	nucleosome organization	1.29E-12	BP
	GO:0000226	microtubule cytoskeleton organization	2.88E-12	BP
	GO:0045786	negative regulation of cell cycle	3.07E-12	BP
	GO:0007346	regulation of mitotic cell cycle	3.12E-12	BP
	GO:0032508	DNA duplex unwinding	4.96E-12	BP
	GO:0051052	regulation of DNA metabolic process	7.46E-12	BP
	GO:1901988	negative regulation of cell cycle phase transition	7.52E-12	BP
	GO:0000725	recombinational repair	8.49E-12	BP
	GO:0031497	chromatin assembly	2.33E-11	BP
	GO:1901987	regulation of cell cycle phase transition	2.51E-11	BP
	GO:0000724	double-strand break repair via homologous recombination	2.77E-11	BP
	**GO:0006268**	**DNA unwinding involved in DNA replication**	**3.37E-11**	**BP**
	GO:0045132	meiotic chromosome segregation	3.80E-11	BP
	GO:0045787	positive regulation of cell cycle	3.98E-11	BP
	GO:1902850	microtubule cytoskeleton organization involved in mitosis	5.19E-11	BP
	GO:0034502	protein localization to chromosome	7.77E-11	BP
	GO:0051383	kinetochore organization	7.90E-11	BP
	GO:0033260	nuclear DNA replication	8.43E-11	BP
	GO:0071459	protein localization to chromosome, centromeric region	8.99E-11	BP
	GO:1901991	negative regulation of mitotic cell cycle phase transition	1.03E-10	BP
	GO:0032392	DNA geometric change	1.16E-10	BP
	GO:0045930	negative regulation of mitotic cell cycle	1.18E-10	BP
	GO:0045005	DNA-dependent DNA replication maintenance of fidelity	1.26E-10	BP
	GO:0051783	regulation of nuclear division	2.11E-10	BP
	**GO:0034501**	**protein localization to kinetochore**	**2.96E-10**	**BP**
	GO:1903083	protein localization to condensed chromosome	2.96E-10	BP
	GO:1901990	regulation of mitotic cell cycle phase transition	3.28E-10	BP
	**GO:0070192**	**chromosome organization involved in meiotic cell cycle**	**9.12E-10**	**BP**
	GO:0071103	DNA conformation change	1.19E-09	BP
	GO:0033045	regulation of sister chromatid segregation	1.19E-09	BP
	GO:0007088	regulation of mitotic nuclear division	1.19E-09	BP
	GO:0007052	mitotic spindle organization	1.35E-09	BP
	GO:0010965	regulation of mitotic sister chromatid separation	1.64E-09	BP
	GO:0071897	DNA biosynthetic process	1.86E-09	BP
	GO:0051303	establishment of chromosome localization	2.00E-09	BP
	GO:0031577	spindle checkpoint	2.11E-09	BP
	GO:0051985	negative regulation of chromosome segregation	2.11E-09	BP
	GO:0030261	chromosome condensation	2.18E-09	BP
	**GO:0051984**	**positive regulation of chromosome segregation**	**3.03E-09**	**BP**
	GO:0050000	chromosome localization	3.47E-09	BP
	GO:0051306	mitotic sister chromatid separation	4.22E-09	BP
	GO:0051310	metaphase plate congression	4.22E-09	BP
	**GO:0031297**	**replication fork processing**	**5.46E-09**	**BP**
	GO:0071174	mitotic spindle checkpoint	5.46E-09	BP
	GO:0045839	negative regulation of mitotic nuclear division	7.26E-09	BP
	GO:0031570	DNA integrity checkpoint	7.78E-09	BP
	GO:0007091	metaphase/anaphase transition of mitotic cell cycle	8.13E-09	BP
	GO:0033046	negative regulation of sister chromatid segregation	8.13E-09	BP
	GO:0033048	negative regulation of mitotic sister chromatid segregation	8.13E-09	BP
	GO:2000816	negative regulation of mitotic sister chromatid separation	8.13E-09	BP
	GO:0044784	metaphase/anaphase transition of cell cycle	1.12E-08	BP
	GO:1905819	negative regulation of chromosome separation	1.25E-08	BP
	GO:0044774	mitotic DNA integrity checkpoint	1.26E-08	BP
	GO:0034508	centromere complex assembly	1.32E-08	BP
	GO:0008608	attachment of spindle microtubules to kinetochore	1.35E-08	BP
	GO:0090329	regulation of DNA-dependent DNA replication	1.45E-08	BP
	**GO:1902969**	**mitotic DNA replication**	**1.50E-08**	**BP**
	GO:0044839	cell cycle G2/M phase transition	1.61E-08	BP
	GO:0051784	negative regulation of nuclear division	2.08E-08	BP
	GO:0033047	regulation of mitotic sister chromatid segregation	2.71E-08	BP
	GO:0045841	negative regulation of mitotic metaphase/anaphase transition	5.11E-08	BP
	GO:2001251	negative regulation of chromosome organization	7.10E-08	BP
	GO:0051225	spindle assembly	7.53E-08	BP
	GO:1902100	negative regulation of metaphase/anaphase transition of cell cycle	7.60E-08	BP
	GO:0006275	regulation of DNA replication	1.07E-07	BP
	GO:0000086	G2/M transition of mitotic cell cycle	1.10E-07	BP
	GO:0007080	mitotic metaphase plate congression	1.20E-07	BP
day 21	GO:0044773	mitotic DNA damage checkpoint	1.55E-07	BP
continue	GO:0030071	regulation of mitotic metaphase/anaphase transition	1.58E-07	BP
	**GO:0006271**	**DNA strand elongation involved in DNA replication**	**1.61E-07**	**BP**
	**GO:0007094**	**mitotic spindle assembly checkpoint**	**2.05E-07**	**BP**
	GO:0071173	spindle assembly checkpoint	2.05E-07	BP
	GO:1902099	regulation of metaphase/anaphase transition of cell cycle	2.10E-07	BP
	GO:0009394	2'-deoxyribonucleotide metabolic process	3.04E-07	BP
	GO:0019692	deoxyribose phosphate metabolic process	3.04E-07	BP
	GO:1900262	regulation of DNA-directed DNA polymerase activity	4.00E-07	BP
	**GO:1900264**	**positive regulation of DNA-directed DNA polymerase activity**	**4.00E-07**	**BP**
	GO:0009263	deoxyribonucleotide biosynthetic process	4.21E-07	BP
	GO:0009265	2'-deoxyribonucleotide biosynthetic process	4.21E-07	BP
	GO:0046385	deoxyribose phosphate biosynthetic process	4.21E-07	BP
	GO:0006338	chromatin remodeling	4.97E-07	BP
	**GO:0000727**	**double-strand break repair via break-induced replication**	**6.02E-07**	**BP**
	GO:0009262	deoxyribonucleotide metabolic process	6.26E-07	BP
	GO:0010389	regulation of G2/M transition of mitotic cell cycle	1.00E-06	BP
	GO:1902749	regulation of cell cycle G2/M phase transition	1.50E-06	BP
	GO:0007143	female meiotic nuclear division	1.54E-06	BP
	**GO:0006335**	**DNA replication-dependent nucleosome assembly**	**1.63E-06**	**BP**
	GO:0034723	DNA replication-dependent nucleosome organization	1.63E-06	BP
	GO:0045143	homologous chromosome segregation	2.32E-06	BP
	GO:0007098	centrosome cycle	3.61E-06	BP
	GO:0000077	DNA damage checkpoint	3.64E-06	BP
	**GO:0051382**	**kinetochore assembly**	**3.64E-06**	**BP**
	GO:0009069	serine family amino acid metabolic process	4.50E-06	BP
	**GO:0007062**	**sister chromatid cohesion**	**4.54E-06**	**BP**
	GO:0051053	negative regulation of DNA metabolic process	5.68E-06	BP
	GO:0009219	pyrimidine deoxyribonucleotide metabolic process	6.58E-06	BP
	**GO:0009157**	**deoxyribonucleoside monophosphate biosynthetic process**	**6.58E-06**	**BP**
	**GO:0046073**	**dTMP metabolic process**	**6.58E-06**	**BP**
	**GO:0007131**	**reciprocal meiotic recombination**	**7.07E-06**	**BP**
	GO:0051054	positive regulation of DNA metabolic process	7.31E-06	BP
	GO:0022613	ribonucleoprotein complex biogenesis	8.78E-06	BP
	GO:0035825	homologous recombination	9.96E-06	BP
	GO:0000018	regulation of DNA recombination	1.10E-05	BP
	GO:2000278	regulation of DNA biosynthetic process	1.10E-05	BP
	GO:0022616	DNA strand elongation	1.38E-05	BP
	**GO:0000076**	**DNA replication checkpoint**	**1.56E-05**	**BP**
	**GO:0007076**	**mitotic chromosome condensation**	**1.56E-05**	**BP**
	**GO:0009221**	**pyrimidine deoxyribonucleotide biosynthetic process**	**1.60E-05**	**BP**
	**GO:2001252**	**positive regulation of chromosome organization**	**1.82E-05**	**BP**
	GO:0031023	microtubule organizing center organization	2.23E-05	BP
	GO:0000212	meiotic spindle organization	2.86E-05	BP
	GO:0009123	nucleoside monophosphate metabolic process	2.86E-05	BP
	GO:0042770	signal transduction in response to DNA damage	3.25E-05	BP
	**GO:0006284**	**base-excision repair**	**3.44E-05**	**BP**
	**GO:0007100**	**mitotic centrosome separation**	**3.44E-05**	**BP**
	**GO:0009070**	**serine family amino acid biosynthetic process**	**3.44E-05**	**BP**
	GO:0006220	pyrimidine nucleotide metabolic process	3.80E-05	BP
	GO:0032465	regulation of cytokinesis	4.27E-05	BP
	GO:0009124	nucleoside monophosphate biosynthetic process	4.50E-05	BP
	GO:0009165	nucleotide biosynthetic process	5.07E-05	BP
	GO:2000573	positive regulation of DNA biosynthetic process	5.14E-05	BP
	GO:1901293	nucleoside phosphate biosynthetic process	5.64E-05	BP
	GO:0044818	mitotic G2/M transition checkpoint	5.95E-05	BP
	**GO:0000281**	**mitotic cytokinesis**	**6.03E-05**	**BP**
	GO:0009176	pyrimidine deoxyribonucleoside monophosphate metabolic process	6.63E-05	BP
	GO:0051299	centrosome separation	6.63E-05	BP
	GO:0009162	deoxyribonucleoside monophosphate metabolic process	6.88E-05	BP
	**GO:1905820**	**positive regulation of chromosome separation**	**6.88E-05**	**BP**
	**GO:0007095**	**mitotic G2 DNA damage checkpoint**	**9.28E-05**	**BP**
	GO:0006221	pyrimidine nucleotide biosynthetic process	1.03E-04	BP
	GO:0010639	negative regulation of organelle organization	1.21E-04	BP
	**GO:0051988**	**regulation of attachment of spindle microtubules to kinetochore**	**1.21E-04**	**BP**
	**GO:0006364**	**rRNA processing**	**1.35E-04**	**BP**
	GO:0051302	regulation of cell division	1.42E-04	BP
	**GO:0000387**	**spliceosomal snRNP assembly**	**1.42E-04**	**BP**
	GO:0007144	female meiosis I	1.44E-04	BP
	GO:0090306	spindle assembly involved in meiosis	1.44E-04	BP
	**GO:0032467**	**positive regulation of cytokinesis**	**1.46E-04**	**BP**
	GO:0009314	response to radiation	1.46E-04	BP
	GO:0042273	ribosomal large subunit biogenesis	1.51E-04	BP
	GO:0000910	cytokinesis	1.59E-04	BP
	GO:0009117	nucleotide metabolic process	1.81E-04	BP
	GO:0006325	chromatin organization	1.82E-04	BP
	GO:0072527	pyrimidine-containing compound metabolic process	1.94E-04	BP
	**GO:0009130**	**pyrimidine nucleoside monophosphate biosynthetic process**	**2.00E-04**	**BP**
	GO:0072528	pyrimidine-containing compound biosynthetic process	2.02E-04	BP
	GO:0016072	rRNA metabolic process	2.03E-04	BP
	GO:0006753	nucleoside phosphate metabolic process	2.38E-04	BP
	**GO:0035999**	**tetrahydrofolate interconversion**	**2.82E-04**	**BP**
	GO:0010972	negative regulation of G2/M transition of mitotic cell cycle	3.20E-04	BP
	**GO:0045910**	**negative regulation of DNA recombination**	**3.28E-04**	**BP**
	GO:0061640	cytoskeleton-dependent cytokinesis	3.70E-04	BP
	GO:1902750	negative regulation of cell cycle G2/M phase transition	4.71E-04	BP
	GO:0010212	response to ionizing radiation	6.75E-04	BP
	GO:0042254	ribosome biogenesis	6.91E-04	BP
	**GO:0000723**	**telomere maintenance**	**7.49E-04**	**BP**
	**GO:0051315**	**attachment of mitotic spindle microtubules to kinetochore**	**9.05E-04**	**BP**
	GO:0055086	nucleobase-containing small molecule metabolic process	9.15E-04	BP
	GO:0019953	sexual reproduction	1.02E-03	BP
	GO:0009129	pyrimidine nucleoside monophosphate metabolic process	1.17E-03	BP
	GO:0051307	meiotic chromosome separation	1.17E-03	BP
	GO:0032200	telomere organization	1.25E-03	BP
	GO:0022618	ribonucleoprotein complex assembly	1.32E-03	BP
	**GO:0010569**	**regulation of double-strand break repair via homologous recombination**	**1.33E-03**	**BP**
	GO:0007276	gamete generation	1.40E-03	BP
	GO:2000779	regulation of double-strand break repair	1.58E-03	BP
	GO:0006336	DNA replication-independent nucleosome assembly	1.64E-03	BP

BP: biological process

Table S1 (continued). Gene Ontology terms for selected upregulated genes. These GO terms are enriched following PFOA treatment on Day 21. Shown in red are the GO terms at the bottom of the hierarchical tree output created using the Explore Biology feature in QuickGO.

	ID	Gene Ontology term	FDR-corrected q-value	Ontology
day 21	**GO:1904666**	**regulation of ubiquitin protein ligase activity**	1.64E-03	BP
continue	GO:0007292	female gamete generation	1.99E-03	BP
	GO:0034724	DNA replication-independent nucleosome organization	2.32E-03	BP
	**GO:1904851**	**positive regulation of establishment of protein localization to telomere**	2.35E-03	BP
	**GO:0000079**	**regulation of cyclin-dependent protein serine/threonine kinase activity**	2.38E-03	BP
	GO:0071478	cellular response to radiation	2.42E-03	BP
	GO:0051347	positive regulation of transferase activity	2.59E-03	BP
	GO:0071826	ribonucleoprotein complex subunit organization	2.71E-03	BP
	GO:1904029	regulation of cyclin-dependent protein kinase activity	2.76E-03	BP
	**GO:0071479**	**cellular response to ionizing radiation**	3.26E-03	BP
	**GO:0009147**	**pyrimidine nucleoside triphosphate metabolic process**	3.26E-03	BP
	**GO:0006301**	**postreplication repair**	3.28E-03	BP
	**GO:1901992**	**positive regulation of mitotic cell cycle phase transition**	3.31E-03	BP
	**GO:0006606**	**protein import into nucleus**	3.33E-03	BP
	**GO:0031055**	**chromatin remodeling at centromere**	3.88E-03	BP
	GO:0070203	regulation of establishment of protein localization to telomere	3.88E-03	BP
	GO:0006282	regulation of DNA repair	4.06E-03	BP
	**GO:0000731**	**DNA synthesis involved in DNA repair**	4.12E-03	BP
	**GO:0090307**	**mitotic spindle assembly**	4.27E-03	BP
	GO:0051170	import into nucleus	4.33E-03	BP
	GO:0046653	tetrahydrofolate metabolic process	4.57E-03	BP
	**GO:0000027**	**ribosomal large subunit assembly**	5.40E-03	BP
	GO:0006913	nucleocytoplasmic transport	5.43E-03	BP
	GO:0051169	nuclear transport	5.43E-03	BP
	**GO:0044843**	**cell cycle G1/S phase transition**	5.47E-03	BP
	**GO:0060623**	**regulation of chromosome condensation**	6.01E-03	BP
	GO:0070202	regulation of establishment of protein localization to chromosome	6.01E-03	BP
	**GO:0036297**	**interstrand cross-link repair**	6.36E-03	BP
	**GO:0072522**	**purine-containing compound biosynthetic process**	6.36E-03	BP
	GO:0048609	multicellular organismal reproductive process	6.38E-03	BP
	GO:0070199	establishment of protein localization to chromosome	6.84E-03	BP
	GO:1901989	positive regulation of cell cycle phase transition	6.94E-03	BP
	GO:0032504	multicellular organism reproduction	7.19E-03	BP
	GO:0071214	cellular response to abiotic stimulus	7.19E-03	BP
	GO:0104004	cellular response to environmental stimulus	7.19E-03	BP
	**GO:0022412**	**cellular process involved in reproduction in multicellular organism**	7.72E-03	BP
	GO:2001020	regulation of response to DNA damage stimulus	7.72E-03	BP
	GO:1904816	positive regulation of protein localization to chromosome, telomeric region	8.87E-03	BP
	GO:0051298	centrosome duplication	9.33E-03	BP
	GO:0009141	nucleoside triphosphate metabolic process	9.79E-03	BP
	GO:0098687	chromosomal region	1.16E-42	CC
	GO:0000775	chromosome, centromeric region	1.64E-41	CC
	GO:0000793	condensed chromosome	2.29E-35	CC
	GO:0000779	condensed chromosome, centromeric region	2.11E-27	CC
	GO:0000776	kinetochore	6.77E-27	CC
	GO:0000228	nuclear chromosome	2.04E-25	CC
	GO:0032993	protein-DNA complex	2.88E-22	CC
	GO:0044815	DNA packaging complex	2.38E-19	CC
	GO:0005819	spindle	1.39E-17	CC
	GO:0000786	nucleosome	3.47E-16	CC
	GO:0072686	mitotic spindle	3.74E-14	CC
	GO:0005657	replication fork	3.86E-14	CC
	GO:0034506	chromosome, centromeric core domain	4.66E-14	CC
	**GO:0043505**	**CENP-A containing nucleosome**	6.93E-13	CC
	GO:0061638	CENP-A containing chromatin	6.93E-13	CC
	**GO:0071162**	**CMG complex**	1.16E-10	CC
	**GO:0000794**	**condensed nuclear chromosome**	1.57E-10	CC
	GO:0031261	DNA replication preinitiation complex	5.87E-10	CC
	GO:0051233	spindle midzone	3.06E-08	CC
	GO:0043596	nuclear replication fork	4.68E-08	CC
	GO:0099513	polymeric cytoskeletal fiber	9.81E-08	CC
	GO:0005874	microtubule	1.47E-07	CC
	**GO:0000940**	**condensed chromosome outer kinetochore**	2.03E-07	CC
	GO:0042555	MCM complex	3.06E-07	CC
	**GO:0000922**	**spindle pole**	3.99E-07	CC
	GO:0005876	spindle microtubule	6.28E-07	CC
	**GO:0043073**	**germ cell nucleus**	4.22E-06	CC
	GO:0000792	heterochromatin	8.75E-06	CC
	GO:0030687	preribosome, large subunit precursor	8.91E-06	CC
	GO:0030684	preribosome	2.74E-05	CC
	GO:0005875	microtubule associated complex	3.27E-05	CC
	**GO:1990023**	**mitotic spindle midzone**	3.50E-05	CC
	**GO:0042575**	**DNA polymerase complex**	4.07E-05	CC
	GO:0030496	midbody	4.24E-05	CC
	GO:0005635	nuclear envelope	5.11E-05	CC
	**GO:0005643**	**nuclear pore**	6.33E-05	CC
	GO:0120114	Sm-like protein family complex	8.47E-05	CC
	**GO:0043601**	**nuclear replisome**	1.40E-04	CC
	GO:0030894	replisome	2.04E-04	CC
	**GO:0071005**	**U2-type precatalytic spliceosome**	2.29E-04	CC
	**GO:0005871**	**kinesin complex**	2.55E-04	CC
	GO:0071011	precatalytic spliceosome	3.32E-04	CC
	**GO:0090734**	**site of DNA damage**	3.63E-04	CC
	**GO:0045120**	**pronucleus**	3.63E-04	CC
	**GO:0000307**	**cyclin-dependent protein kinase holoenzyme complex**	3.68E-04	CC
	**GO:0046540**	**U4/U6 x U5 tri-snRNP complex**	4.03E-04	CC
	GO:0097526	spliceosomal tri-snRNP complex	4.03E-04	CC
	**GO:0000781**	**chromosome, telomeric region**	4.34E-04	CC
	GO:0030532	small nuclear ribonucleoprotein complex	6.74E-04	CC
	GO:0097525	spliceosomal snRNP complex	7.28E-04	CC
	**GO:0005721**	**pericentric heterochromatin**	8.31E-04	CC
	**GO:0072687**	**meiotic spindle**	9.80E-04	CC
	**GO:0031965**	**nuclear membrane**	1.65E-03	CC
	**GO:0097449**	**astrocyte projection**	1.65E-03	CC
	**GO:0034709**	**methylosome**	2.64E-03	CC
	**GO:1990498**	**mitotic spindle microtubule**	4.00E-03	CC
	GO:0061695	transferase complex, transferring phosphorus-containing groups	6.13E-03	CC
	GO:0005681	spliceosomal complex	9.73E-03	CC

BP: biological process; CC: cellular component

Table S2

Gene Ontology terms for selected downregulated genes. These GO terms are enriched due to PFOA treatment for 1 or 24 hours. Shown in red are the GO terms at the bottom of the hierarchical tree output created using the Explore Biology feature in QuickGO.

	ID	Gene Ontology term	FDR-corrected q-value	Ontology
1 hour	GO:1902041	regulation of extrinsic apoptotic signaling pathway via death domain receptors	5.08E-04	BP
	GO:0008625	extrinsic apoptotic signaling pathway via death domain receptors	1.40E-03	BP
	GO:0070423	nucleotide-binding oligomerization domain containing signaling pathway	2.75E-03	BP
	**GO:0070431**	**nucleotide-binding oligomerization domain containing 2 signaling pathway**	2.75E-03	BP
	GO:0035872	nucleotide-binding domain, leucine rich repeat containing receptor signaling pathway	2.75E-03	BP
	**GO:0032495**	**response to muramyl dipeptide**	2.75E-03	BP
	**GO:2000352**	**negative regulation of endothelial cell apoptotic process**	2.75E-03	BP
	GO:2001236	regulation of extrinsic apoptotic signaling pathway	2.75E-03	BP
	**GO:1902042**	**negative regulation of extrinsic apoptotic signaling pathway via death domain receptors**	4.23E-03	BP
	**GO:0030155**	**regulation of cell adhesion**	4.64E-03	BP
	**GO:0050730**	**regulation of peptidyl-tyrosine phosphorylation**	4.64E-03	BP
	GO:0097191	extrinsic apoptotic signaling pathway	5.14E-03	BP
	GO:2000351	regulation of endothelial cell apoptotic process	5.14E-03	BP
	GO:1904036	negative regulation of epithelial cell apoptotic process	5.14E-03	BP
	GO:0072577	endothelial cell apoptotic process	5.14E-03	BP
	GO:0018108	peptidyl-tyrosine phosphorylation	5.45E-03	BP
	GO:0018212	peptidyl-tyrosine modification	5.45E-03	BP
	GO:0002753	cytoplasmic pattern recognition receptor signaling pathway	5.91E-03	BP
	**GO:0031663**	**lipopolysaccharide-mediated signaling pathway**	5.91E-03	BP
	**GO:0033627**	**cell adhesion mediated by integrin**	7.08E-03	BP
	**GO:0002260**	**lymphocyte homeostasis**	8.95E-03	BP
	**GO:0000079**	**regulation of cyclin-dependent protein serine/threonine kinase activity**	9.89E-03	BP
	**GO:0032088**	**negative regulation of NF-kappaB transcription factor activity**	9.89E-03	BP
	GO:1904029	regulation of cyclin-dependent protein kinase activity	9.89E-03	BP
	**GO:1901652**	**response to peptide**	9.89E-03	BP
	GO:1904035	regulation of epithelial cell apoptotic process	9.89E-03	BP
	**GO:0070062**	**extracellular exosome**	6.34E-03	CC
	GO:1903561	extracellular vesicle	6.34E-03	CC
	GO:0043230	extracellular organelle	6.34E-03	CC
	GO:0065010	extracellular membrane-bounded organelle	6.34E-03	CC
6 hours	None			

BP: biological process; CC: cellular component

	ID	Gene Ontology term	FDR-corrected q-value	Ontology
24 hours	**GO:0035458**	**cellular response to interferon-beta**	2.47E-30	BP
	GO:0035456	response to interferon-beta	4.27E-29	BP
	GO:0009615	response to virus	5.19E-27	BP
	**GO:0051607**	**defense response to virus**	4.94E-26	BP
	GO:0045087	innate immune response	9.79E-18	BP
	GO:0009617	response to bacterium	1.21E-14	BP
	GO:0048525	negative regulation of viral process	7.00E-14	BP
	**GO:0045071**	**negative regulation of viral genome replication**	1.15E-13	BP
	GO:0035455	response to interferon-alpha	2.66E-13	BP
	GO:0060700	regulation of ribonuclease activity	5.69E-12	BP
	GO:0045069	regulation of viral genome replication	1.26E-11	BP
	GO:0034340	response to type I interferon	1.42E-11	BP
	GO:0045824	negative regulation of innate immune response	1.42E-11	BP
	**GO:0035457**	**cellular response to interferon-alpha**	2.09E-11	BP
	GO:0032481	positive regulation of type I interferon production	2.27E-11	BP
	GO:0050792	regulation of viral process	2.27E-11	BP
	**GO:0032728**	**positive regulation of interferon-beta production**	4.28E-11	BP
	GO:0060337	type I interferon signaling pathway	2.01E-10	BP
	GO:0071357	cellular response to type I interferon	2.01E-10	BP
	GO:0019079	viral genome replication	6.53E-10	BP
	GO:0002832	negative regulation of response to biotic stimulus	7.84E-10	BP
	GO:0032608	interferon-beta production	8.01E-10	BP
	GO:0032648	regulation of interferon-beta production	8.01E-10	BP
	GO:1903900	regulation of viral life cycle	8.41E-10	BP
	GO:0032069	regulation of nuclease activity	1.08E-09	BP
	GO:0032479	regulation of type I interferon production	1.08E-09	BP
	GO:0032606	type I interferon production	1.72E-09	BP
	GO:0042742	defense response to bacterium	4.17E-09	BP
	GO:0050777	negative regulation of immune response	1.16E-08	BP
	**GO:0060339**	**negative regulation of type I interferon-mediated signaling pathway**	1.86E-08	BP
	GO:0002831	regulation of response to biotic stimulus	3.00E-08	BP
	GO:0045088	regulation of innate immune response	3.13E-08	BP
	GO:0060338	regulation of type I interferon-mediated signaling pathway	3.13E-08	BP
	GO:0019221	cytokine-mediated signaling pathway	5.57E-08	BP
	GO:0001960	negative regulation of cytokine-mediated signaling pathway	1.15E-07	BP
	GO:0016032	viral process	1.29E-07	BP
	GO:0031348	negative regulation of defense response	1.71E-07	BP
	GO:0060761	negative regulation of response to cytokine stimulus	2.02E-07	BP
	GO:0001819	positive regulation of cytokine production	2.02E-07	BP
	GO:0019058	viral life cycle	2.93E-07	BP
	GO:0002221	pattern recognition receptor signaling pathway	4.91E-07	BP
	GO:0034341	response to interferon-gamma	1.09E-06	BP
	GO:0001959	regulation of cytokine-mediated signaling pathway	1.78E-06	BP
	**GO:0042832**	**defense response to protozoan**	1.86E-06	BP
	**GO:0032760**	**positive regulation of tumor necrosis factor production**	3.13E-06	BP
	GO:0060759	regulation of response to cytokine stimulus	3.32E-06	BP
	GO:1903557	positive regulation of tumor necrosis factor superfamily cytokine production	3.32E-06	BP
	GO:0001562	response to protozoan	3.65E-06	BP
	GO:0031347	regulation of defense response	4.02E-06	BP
	**GO:0044406**	**adhesion of symbiont to host**	4.06E-06	BP
	GO:0071605	monocyte chemotactic protein-1 production	9.11E-06	BP
	**GO:0071637**	**regulation of monocyte chemotactic protein-1 production**	9.11E-06	BP
	GO:0050776	regulation of immune response	9.44E-06	BP
	GO:0032102	negative regulation of response to external stimulus	1.01E-05	BP
	**GO:0034138**	**toll-like receptor 3 signaling pathway**	1.73E-05	BP
	GO:0002683	negative regulation of immune system process	1.79E-05	BP
	GO:0001817	regulation of cytokine production	2.31E-05	BP
	**GO:0032682**	**negative regulation of chemokine production**	3.84E-05	BP
	GO:0002764	immune response-regulating signaling pathway	4.45E-05	BP
	GO:0032680	regulation of tumor necrosis factor production	4.45E-05	BP
	GO:1903555	regulation of tumor necrosis factor superfamily cytokine production	4.96E-05	BP
	GO:0032640	tumor necrosis factor production	5.52E-05	BP
	GO:0072567	chemokine (C-X-C motif) ligand 2 production	5.62E-05	BP
	**GO:2000341**	**regulation of chemokine (C-X-C motif) ligand 2 production**	5.62E-05	BP
	**GO:0001816**	**cytokine production**	5.62E-05	BP
	GO:0071706	tumor necrosis factor superfamily cytokine production	5.84E-05	BP
	**GO:0032722**	**positive regulation of chemokine production**	6.04E-05	BP
	**GO:0002224**	**toll-like receptor signaling pathway**	6.27E-05	BP
	**GO:0071346**	**cellular response to interferon-gamma**	9.05E-05	BP
	**GO:0098586**	**cellular response to virus**	2.03E-04	BP
	GO:0032642	regulation of chemokine production	2.85E-04	BP
	GO:0032602	chemokine production	3.60E-04	BP
	**GO:0062208**	**positive regulation of pattern recognition receptor signaling pathway**	3.78E-04	BP
	GO:0062207	regulation of pattern recognition receptor signaling pathway	5.94E-04	BP
	**GO:0043129**	**surfactant homeostasis**	6.27E-04	BP
	GO:0048875	chemical homeostasis within a tissue	7.84E-04	BP
	**GO:0032727**	**positive regulation of interferon-alpha production**	1.18E-03	BP
	**GO:0050830**	**defense response to Gram-positive bacterium**	1.32E-03	BP
	**GO:1901857**	**positive regulation of cellular respiration**	1.66E-03	BP
	GO:0032647	regulation of interferon-alpha production	1.94E-03	BP
	**GO:0039529**	**RIG-I signaling pathway**	3.00E-03	BP
	GO:0032607	interferon-alpha production	3.40E-03	BP
	GO:0039528	cytoplasmic pattern recognition receptor signaling pathway in response to virus	4.32E-03	BP
	**GO:0034142**	**toll-like receptor 4 signaling pathway**	6.01E-03	BP
	**GO:0003725**	**double-stranded RNA binding**	3.97E-11	MF
	**GO:0003924**	**GTPase activity**	2.19E-10	MF
	GO:0017111	nucleoside-triphosphatase activity	2.95E-09	MF
	GO:0016462	pyrophosphatase activity	8.58E-09	MF
	GO:0016818	hydrolase activity, acting on acid anhydrides, in phosphorus-containing anhydrides	8.58E-09	MF
	GO:0016817	hydrolase activity, acting on acid anhydrides	8.58E-09	MF
	**GO:0070566**	**adenylyltransferase activity**	2.93E-07	MF
	GO:0016779	nucleotidyltransferase activity	9.26E-05	MF
	GO:0043230	extracellular organelle	2.59E-04	CC
	**GO:0065010**	**extracellular membrane-bounded organelle**	2.59E-04	CC
	**GO:0005789**	**endoplasmic reticulum membrane**	9.65E-03	CC
	GO:0098827	endoplasmic reticulum subcompartment	9.65E-03	CC
	GO:0042175	nuclear outer membrane-endoplasmic reticulum membrane network	9.65E-03	CC

BP: biological process; MF: molecular function; CC: cellular component

Table S2 (continued). Gene Ontology terms for selected downregulated genes. These GO terms are enriched due to PFOA treatment for 8 days. Shown in red are the GO terms at the bottom of the hierarchical tree output created using the Explore Biology feature in QuickGO.

	ID	Gene Ontology term	FDR-corrected q-value	Ontology
8 days	GO:0035456	response to interferon-beta	4.81E-21	BP
	**GO:0035458**	**cellular response to interferon-beta**	4.05E-18	BP
	GO:0045087	innate immune response	9.16E-18	BP
	GO:0009615	response to virus	2.00E-16	BP
	**GO:0051607**	**defense response to virus**	4.27E-16	BP
	GO:0009617	response to bacterium	2.04E-14	BP
	GO:0048525	negative regulation of viral process	7.18E-10	BP
	GO:0031347	regulation of defense response	1.22E-08	BP
	GO:0050776	regulation of immune response	2.71E-08	BP
	**GO:0045071**	**negative regulation of viral genome replication**	6.89E-08	BP
	GO:0002684	positive regulation of immune system process	1.09E-07	BP
	GO:0042742	defense response to bacterium	1.47E-07	BP
	GO:0035455	response to interferon-alpha	1.58E-07	BP
	GO:0050792	regulation of viral process	1.64E-07	BP
	GO:0002831	regulation of response to biotic stimulus	2.48E-07	BP
	GO:0045088	regulation of innate immune response	3.46E-07	BP
	GO:0001816	cytokine production	3.59E-07	BP
	GO:1903900	regulation of viral life cycle	3.75E-07	BP
	GO:0050778	positive regulation of immune response	2.36E-06	BP
	GO:0034341	response to interferon-gamma	2.96E-06	BP
	GO:0032481	positive regulation of type I interferon production	3.43E-06	BP
	GO:0002711	positive regulation of T cell mediated immunity	4.25E-06	BP
	GO:0001817	regulation of cytokine production	4.67E-06	BP
	GO:0045069	regulation of viral genome replication	5.03E-06	BP
	GO:0001819	positive regulation of cytokine production	5.48E-06	BP
	GO:0019221	cytokine-mediated signaling pathway	9.98E-06	BP
	GO:0034340	response to type I interferon	1.66E-05	BP
	GO:0002237	response to molecule of bacterial origin	1.66E-05	BP
	**GO:0032728**	**positive regulation of interferon-beta production**	1.67E-05	BP
	GO:0033002	muscle cell proliferation	2.63E-05	BP
	GO:0001503	ossification	3.04E-05	BP
	GO:0002709	regulation of T cell mediated immunity	3.59E-05	BP
	GO:0019058	viral life cycle	3.59E-05	BP
	GO:0032606	type I interferon production	4.09E-05	BP
	GO:0019079	viral genome replication	4.69E-05	BP
	GO:0002697	regulation of immune effector process	5.47E-05	BP
	GO:0060337	type I interferon signaling pathway	5.99E-05	BP
	GO:0071357	cellular response to type I interferon	5.99E-05	BP
	GO:0002367	cytokine production involved in immune response	7.00E-05	BP
	GO:0031341	regulation of cell killing	7.00E-05	BP
	GO:0002705	positive regulation of leukocyte mediated immunity	7.34E-05	BP
	GO:0002703	regulation of leukocyte mediated immunity	7.42E-05	BP
	GO:0001916	positive regulation of T cell mediated cytotoxicity	7.94E-05	BP
	GO:0016032	viral process	8.03E-05	BP
	GO:0032496	response to lipopolysaccharide	8.44E-05	BP
	GO:0045824	negative regulation of innate immune response	1.06E-04	BP
	GO:0001914	regulation of T cell mediated cytotoxicity	1.28E-04	BP
	GO:0032479	regulation of type I interferon production	1.28E-04	BP
	GO:0002708	positive regulation of lymphocyte mediated immunity	1.42E-04	BP
	GO:0002683	negative regulation of immune system process	1.42E-04	BP
	**GO:0060700**	**regulation of ribonuclease activity**	1.54E-04	BP
	GO:0001910	regulation of leukocyte mediated cytotoxicity	1.57E-04	BP
	GO:0071222	cellular response to lipopolysaccharide	1.67E-04	BP
	GO:0001909	leukocyte mediated cytotoxicity	1.69E-04	BP
	GO:0001912	positive regulation of leukocyte mediated cytotoxicity	1.69E-04	BP
	GO:0032608	interferon-beta production	1.69E-04	BP
	GO:0032648	regulation of interferon-beta production	1.69E-04	BP
	GO:0002833	positive regulation of response to biotic stimulus	1.72E-04	BP
	GO:0030155	regulation of cell adhesion	1.91E-04	BP
	GO:0050777	negative regulation of immune response	1.94E-04	BP
	GO:0045785	positive regulation of cell adhesion	1.94E-04	BP
	GO:0071219	cellular response to molecule of bacterial origin	2.60E-04	BP
	GO:0071216	cellular response to biotic stimulus	2.60E-04	BP
	GO:0031343	positive regulation of cell killing	2.60E-04	BP
	GO:0002456	T cell mediated immunity	3.18E-04	BP
	**GO:0035457**	**cellular response to interferon-alpha**	3.18E-04	BP
	GO:0031349	positive regulation of defense response	3.85E-04	BP
	GO:0002443	leukocyte mediated immunity	3.91E-04	BP
	GO:0002252	immune effector process	3.91E-04	BP
	GO:0001906	cell killing	3.97E-04	BP
	GO:0002706	regulation of lymphocyte mediated immunity	4.04E-04	BP
	GO:0002824	positive regulation of adaptive immune response based on somatic recombination of immune receptors built from immunoglobulin superfamily domains	4.15E-04	BP
	GO:0045089	positive regulation of innate immune response	4.22E-04	BP
	GO:0032103	positive regulation of response to external stimulus	4.22E-04	BP
	GO:0001913	T cell mediated cytotoxicity	4.25E-04	BP
	GO:0002699	positive regulation of immune effector process	5.45E-04	BP
	GO:0032102	negative regulation of response to external stimulus	5.64E-04	BP
	GO:0071346	cellular response to interferon-gamma	6.08E-04	BP
	GO:0031348	negative regulation of defense response	6.62E-04	BP
	GO:0002821	positive regulation of adaptive immune response	6.62E-04	BP
	GO:0002718	regulation of cytokine production involved in immune response	7.67E-04	BP
	GO:0008285	negative regulation of cell population proliferation	7.93E-04	BP
	GO:0031214	biomineral tissue development	8.20E-04	BP
	GO:0060759	regulation of response to cytokine stimulus	1.07E-03	BP
	GO:0002449	lymphocyte mediated immunity	1.07E-03	BP
	GO:0002475	antigen processing and presentation via MHC class Ib	1.09E-03	BP
	GO:0031663	lipopolysaccharide-mediated signaling pathway	1.12E-03	BP
	GO:0110148	biomineralization	1.12E-03	BP
	GO:0002819	regulation of adaptive immune response	1.28E-03	BP
	**GO:0060339**	**negative regulation of type I interferon-mediated signaling pathway**	1.41E-03	BP
	GO:0006954	inflammatory response	1.64E-03	BP
	**GO:0050830**	**defense response to Gram-positive bacterium**	1.73E-03	BP
	**GO:0048660**	**regulation of smooth muscle cell proliferation**	1.79E-03	BP
	**GO:0019884**	**antigen processing and presentation of exogenous antigen**	1.80E-03	BP
	GO:0002832	negative regulation of response to biotic stimulus	1.83E-03	BP
	GO:0042330	taxis	1.86E-03	BP
	**GO:0070167**	**regulation of biomineral tissue development**	2.12E-03	BP
	GO:0048659	smooth muscle cell proliferation	2.12E-03	BP
	GO:0002221	pattern recognition receptor signaling pathway	2.12E-03	BP
	GO:0001959	regulation of cytokine-mediated signaling pathway	2.12E-03	BP
8 days continue	**GO:0030282**	**bone mineralization**	2.12E-03	BP
	**GO:0042832**	**defense response to protozoan**	2.17E-03	BP
	GO:0002822	regulation of adaptive immune response based on somatic recombination of immune receptors built from immunoglobulin superfamily domains	2.21E-03	BP
	GO:0110149	regulation of biomineralization	2.25E-03	BP
	GO:0002764	immune response-regulating signaling pathway	2.28E-03	BP
	GO:0001568	blood vessel development	2.39E-03	BP
	**GO:0019883**	**antigen processing and presentation of endogenous antigen**	2.66E-03	BP
	**GO:0060044**	**negative regulation of cardiac muscle cell proliferation**	3.23E-03	BP
	GO:0032069	regulation of nuclease activity	3.31E-03	BP
	GO:0048514	blood vessel morphogenesis	3.57E-03	BP
	GO:0060338	regulation of type I interferon-mediated signaling pathway	3.63E-03	BP
	GO:0001562	response to protozoan	4.04E-03	BP
	GO:0006935	chemotaxis	4.15E-03	BP
	GO:0001525	angiogenesis	4.22E-03	BP
	**GO:0032680**	**regulation of tumor necrosis factor production**	4.65E-03	BP
	GO:1903706	regulation of hemopoiesis	4.71E-03	BP
	**GO:1903901**	**negative regulation of viral life cycle**	4.79E-03	BP
	GO:1903555	regulation of tumor necrosis factor superfamily cytokine production	5.29E-03	BP
	**GO:0002428**	**antigen processing and presentation of peptide antigen via MHC class Ib**	5.66E-03	BP
	**GO:0060333**	**interferon-gamma-mediated signaling pathway**	5.66E-03	BP
	**GO:0002474**	**antigen processing and presentation of peptide antigen via MHC class I**	5.66E-03	BP
	**GO:0045621**	**positive regulation of lymphocyte differentiation**	5.66E-03	BP
	GO:0032640	tumor necrosis factor production	5.90E-03	BP
	GO:0060537	muscle tissue development	5.97E-03	BP
	**GO:0022409**	**positive regulation of cell-cell adhesion**	6.61E-03	BP
	GO:0071706	tumor necrosis factor superfamily cytokine production	6.67E-03	BP
	GO:0014706	striated muscle tissue development	6.89E-03	BP
	**GO:0002726**	**positive regulation of T cell cytokine production**	7.10E-03	BP
	**GO:0002224**	**toll-like receptor signaling pathway**	7.10E-03	BP
	**GO:0051091**	**positive regulation of DNA-binding transcription factor activity**	7.10E-03	BP
	GO:0001960	negative regulation of cytokine-mediated signaling pathway	8.65E-03	BP
	GO:0002460	adaptive immune response based on somatic recombination of immune receptors built from immunoglobulin superfamily domains	8.65E-03	BP
	GO:0031664	regulation of lipopolysaccharide-mediated signaling pathway	8.86E-03	BP
	**GO:0032727**	**positive regulation of interferon-alpha production**	8.86E-03	BP
	**GO:0045765**	**regulation of angiogenesis**	8.86E-03	BP
	GO:0055017	cardiac muscle tissue growth	8.86E-03	BP
	GO:0035265	organ growth	8.86E-03	BP
	**GO:0050727**	**regulation of inflammatory response**	9.66E-03	BP
	**GO:0046889**	**positive regulation of lipid biosynthetic process**	9.74E-03	BP
	**GO:0042608**	**T cell receptor binding**	1.84E-03	MF
	GO:0030545	receptor regulator activity	1.84E-03	MF
	**GO:0048018**	**receptor ligand activity**	1.84E-03	MF
	**GO:0050839**	**cell adhesion molecule binding**	1.84E-03	MF
	GO:0030546	signaling receptor activator activity	1.84E-03	MF
	**GO:0003725**	**double-stranded RNA binding**	2.68E-03	MF
	**GO:0070566**	**adenylyltransferase activity**	2.68E-03	MF
	**GO:0003924**	**GTPase activity**	4.32E-03	MF
	**GO:0030881**	**beta-2-microglobulin binding**	4.40E-03	MF
	GO:0030312	external encapsulating structure	2.89E-04	CC
	**GO:0031012**	**extracellular matrix**	2.89E-04	CC
	**GO:0042612**	**MHC class I protein complex**	2.89E-04	CC
	GO:0009986	cell surface	2.93E-04	CC
	GO:0042611	MHC protein complex	1.04E-03	CC
	**GO:0009897**	**external side of plasma membrane**	1.40E-03	CC

BP: biological process; MF: molecular function; CC: cellular component

Table S2 (continued). Gene Ontology terms for selected downregulated genes. These GO terms are enriched due to PFOA treatment on Day 21. Shown in red are the GO terms at the bottom of the hierarchical tree output created using the Explore Biology feature in QuickGO.

	ID	Gene Ontology term	FDR-corrected q-value	Ontology
day 21	GO:0001503	ossification	1.22E-03	BP
	**GO:0007169**	**transmembrane receptor protein tyrosine kinase signaling pathway**	1.22E-03	BP
	GO:0060350	endochondral bone morphogenesis	2.82E-03	BP
	GO:0001568	blood vessel development	2.82E-03	BP
	**GO:0001935**	**endothelial cell proliferation**	6.86E-03	BP
	**GO:0061061**	**muscle structure development**	6.86E-03	BP
	GO:0060348	bone development	6.86E-03	BP
	GO:0048705	skeletal system morphogenesis	6.86E-03	BP
	GO:0006941	striated muscle contraction	7.98E-03	BP
	GO:0048514	blood vessel morphogenesis	7.98E-03	BP
	**GO:0003009**	**skeletal muscle contraction**	7.98E-03	BP
	**GO:0001525**	**angiogenesis**	9.41E-03	BP
	**GO:1903522**	**regulation of blood circulation**	9.41E-03	BP
	**GO:0001649**	**osteoblast differentiation**	9.41E-03	BP
	**GO:0061448**	**connective tissue development**	9.41E-03	BP
	GO:0014065	phosphatidylinositol 3-kinase signaling	9.41E-03	BP
	**GO:0001958**	**endochondral ossification**	9.41E-03	BP
	GO:0036075	replacement ossification	9.41E-03	BP
	**GO:0044344**	**cellular response to fibroblast growth factor stimulus**	9.41E-03	BP
	**GO:0005201**	**extracellular matrix structural constituent**	3.83E-07	MF
	GO:0038023	signaling receptor activity	2.36E-04	MF
	GO:0060089	molecular transducer activity	2.36E-04	MF
	GO:0004888	transmembrane signaling receptor activity	5.58E-04	MF
	**GO:0046935**	**1-phosphatidylinositol-3-kinase regulator activity**	5.61E-04	MF
	GO:0019838	growth factor binding	9.02E-04	MF
	**GO:0035014**	**phosphatidylinositol 3-kinase regulator activity**	1.42E-03	MF
	**GO:0004896**	**cytokine receptor activity**	2.84E-03	MF
	GO:0140375	immune receptor activity	3.28E-03	MF
	**GO:0043425**	**bHLH transcription factor binding**	3.78E-03	MF
	GO:0005198	structural molecule activity	3.99E-03	MF
	**GO:0048407**	**platelet-derived growth factor binding**	6.68E-03	MF
	GO:0140297	DNA-binding transcription factor binding	7.57E-03	MF
	**GO:0005159**	**insulin-like growth factor receptor binding**	7.57E-03	MF
	**GO:0008201**	**heparin binding**	8.50E-03	MF
	GO:0062023	collagen-containing extracellular matrix	6.14E-07	CC
	GO:0030312	external encapsulating structure	3.52E-06	CC
	GO:0031012	extracellular matrix	3.52E-06	CC
	GO:0009986	cell surface	1.21E-03	CC
	**GO:0005942**	**phosphatidylinositol 3-kinase complex**	5.34E-03	CC
	**GO:0005583**	**fibrillar collagen trimer**	5.34E-03	CC
	GO:0098643	banded collagen fibril	5.34E-03	CC

BP: biological process; MF: molecular function; CC: cellular component

Figure S1

Phase 1- Functionalization of compounds

24 hours
CAGE analysis

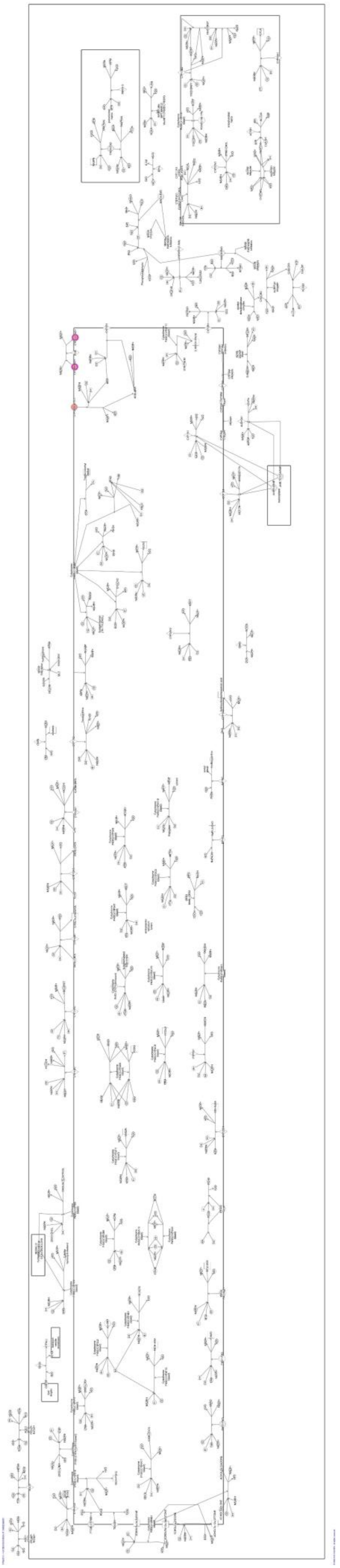

Figure S2

Phase 1-
Functionalization of
compounds

Day 21
CAGE analysis

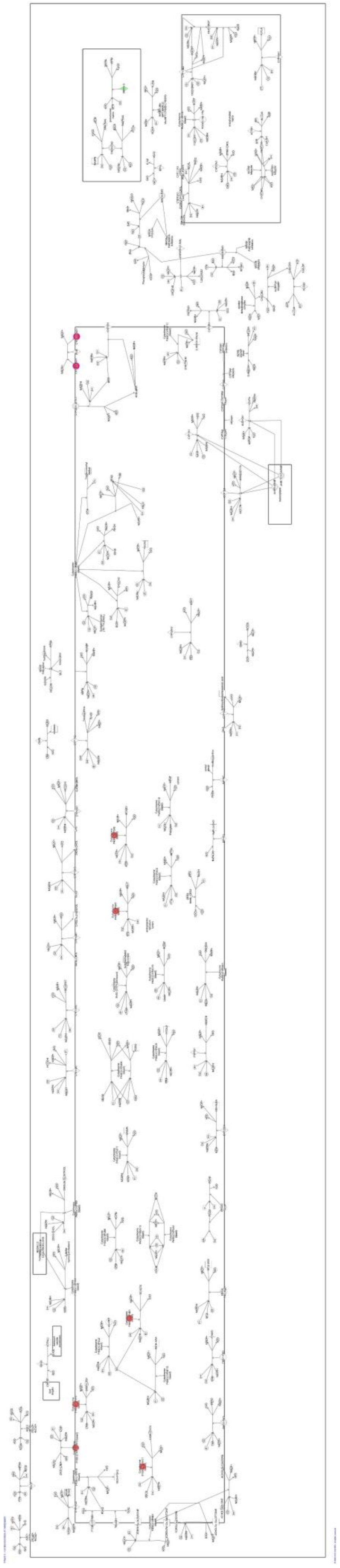

Figure S3

Aryl Hydrocarbon Receptor Signaling

24 hours
CAGE analysis

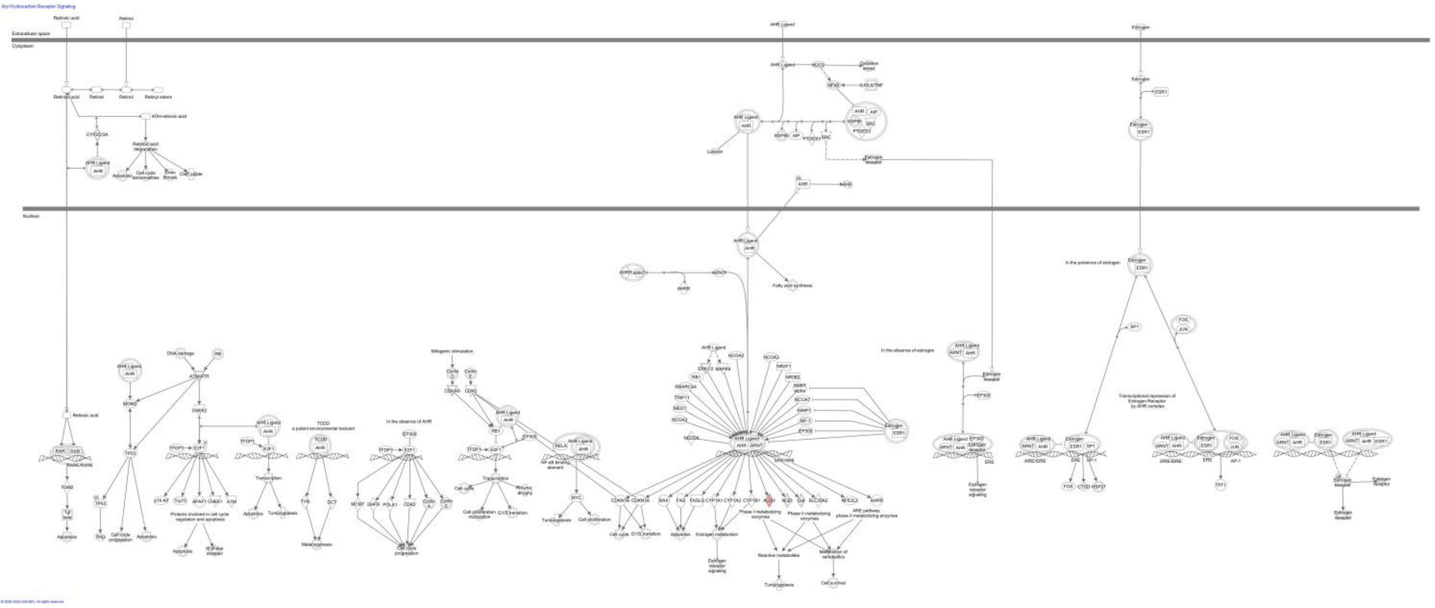

Figure S4

Aryl Hydrocarbon Receptor Signaling

8 days
CAGE analysis

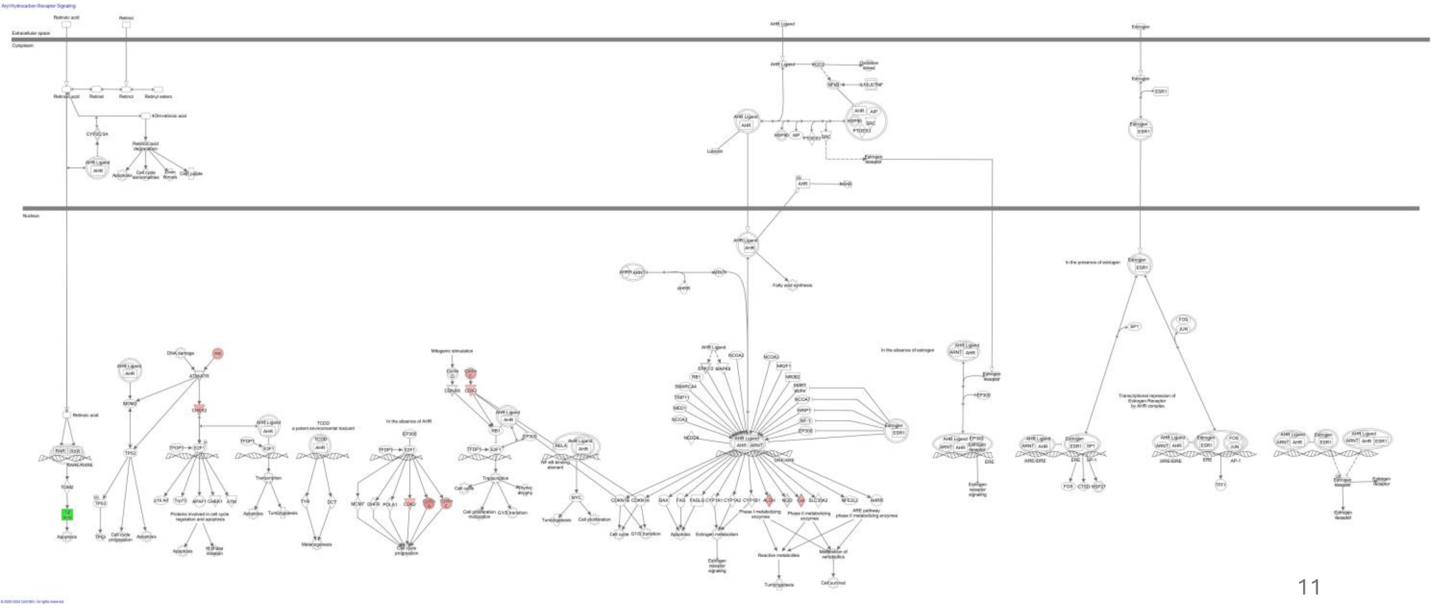

Figure S5

Aryl Hydrocarbon Receptor Signaling

Day 21
CAGE analysis

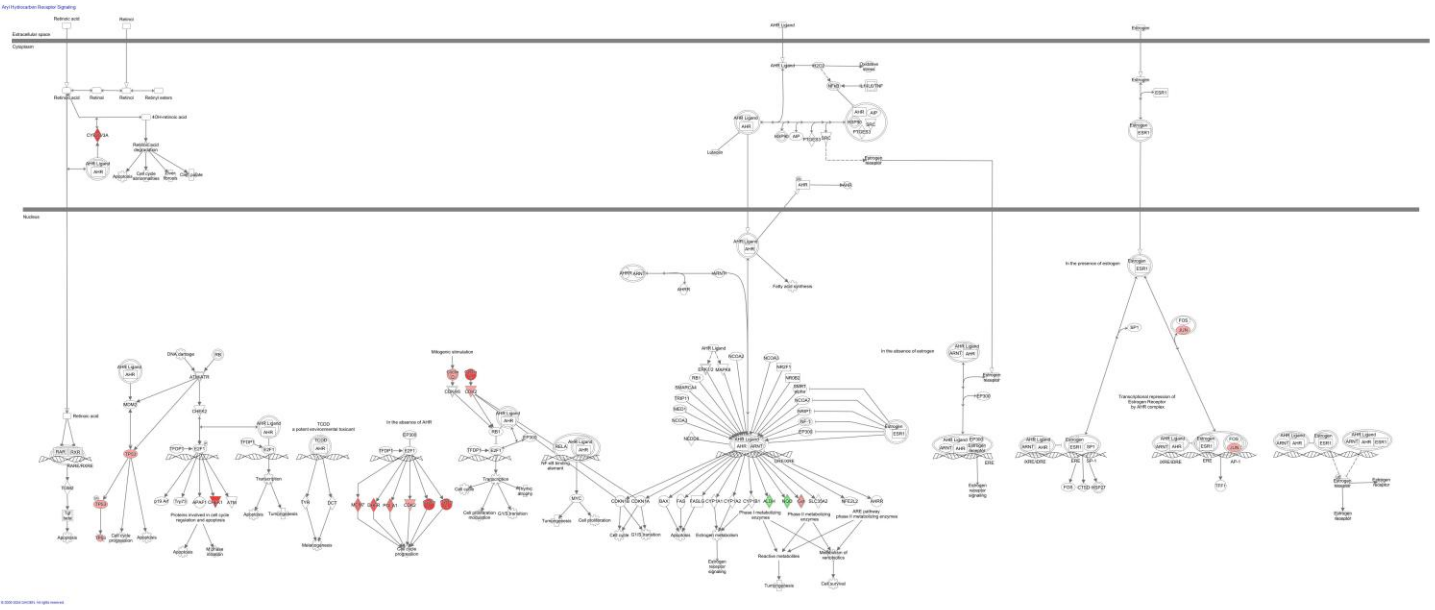

Figure S6

Xenobiotic Metabolism Signaling

6 hour
CAGE analysis

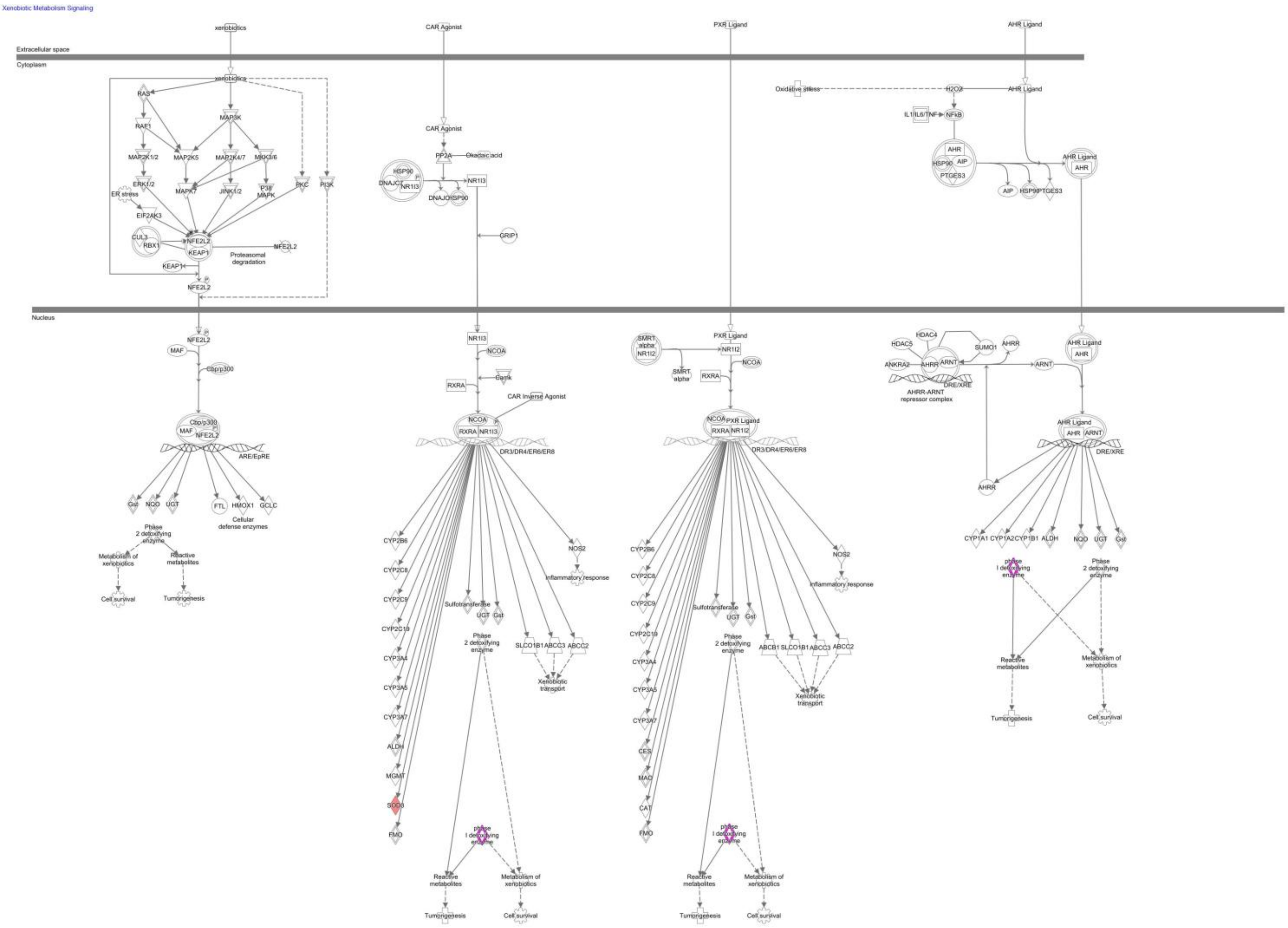

Figure S7

Xenobiotic Metabolism Signaling

24 hour
CAGE analysis

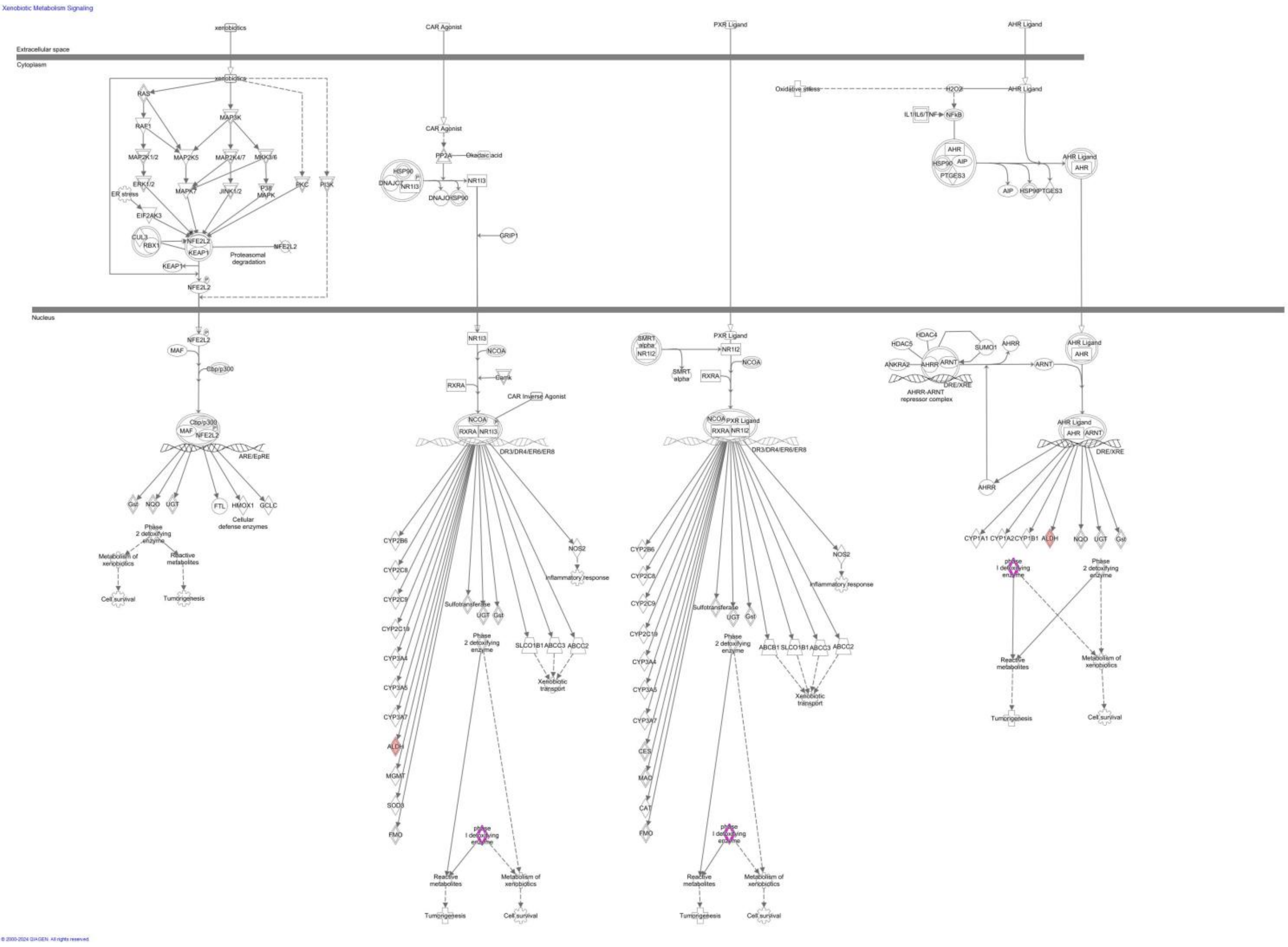

Figure S8

Xenobiotic Metabolism Signaling

8 days
CAGE analysis

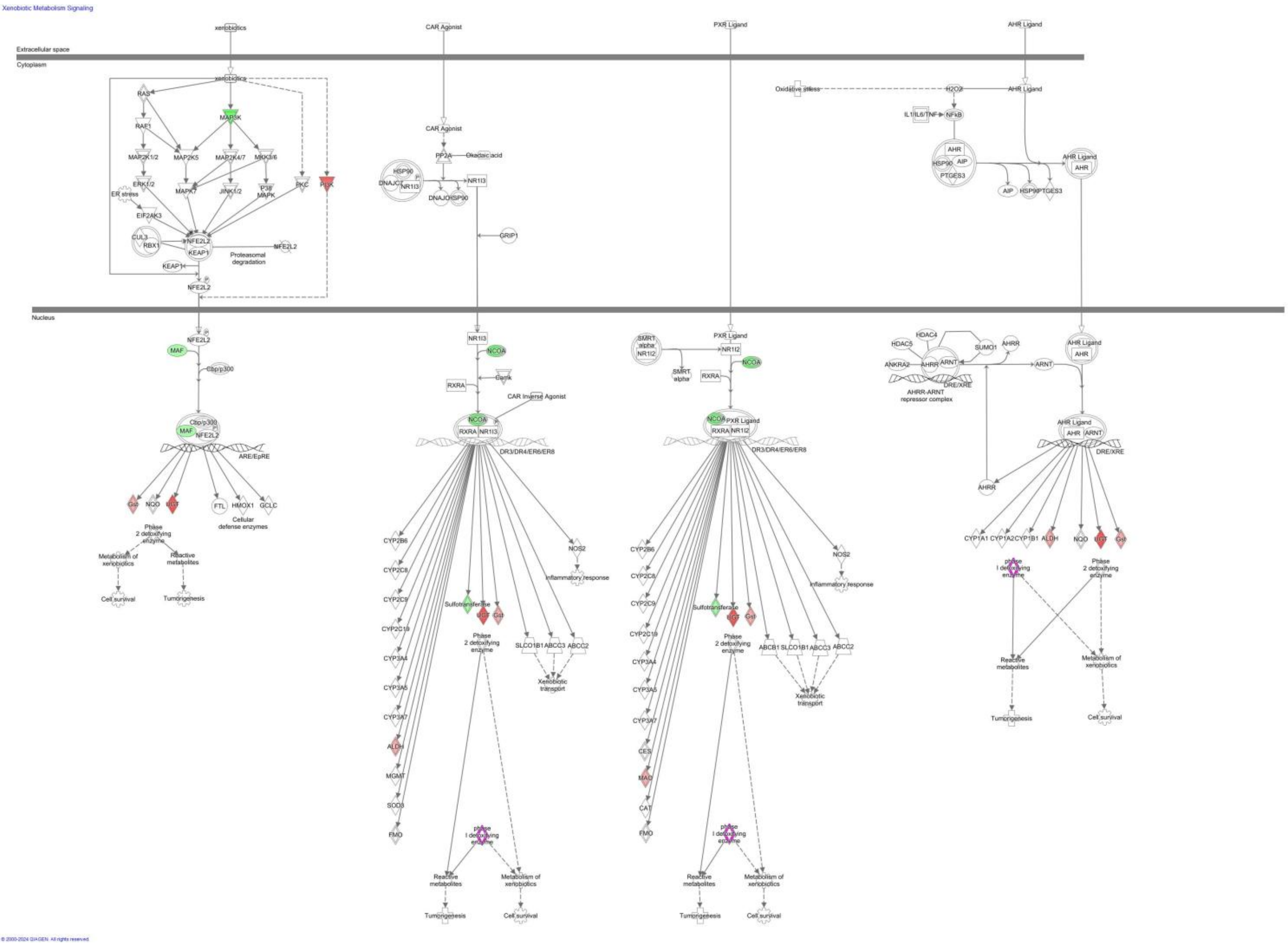

© 2000-2024 QIAGEN. All rights reserved.

Figure S9

Xenobiotic Metabolism Signaling

Day 21
CAGE analysis

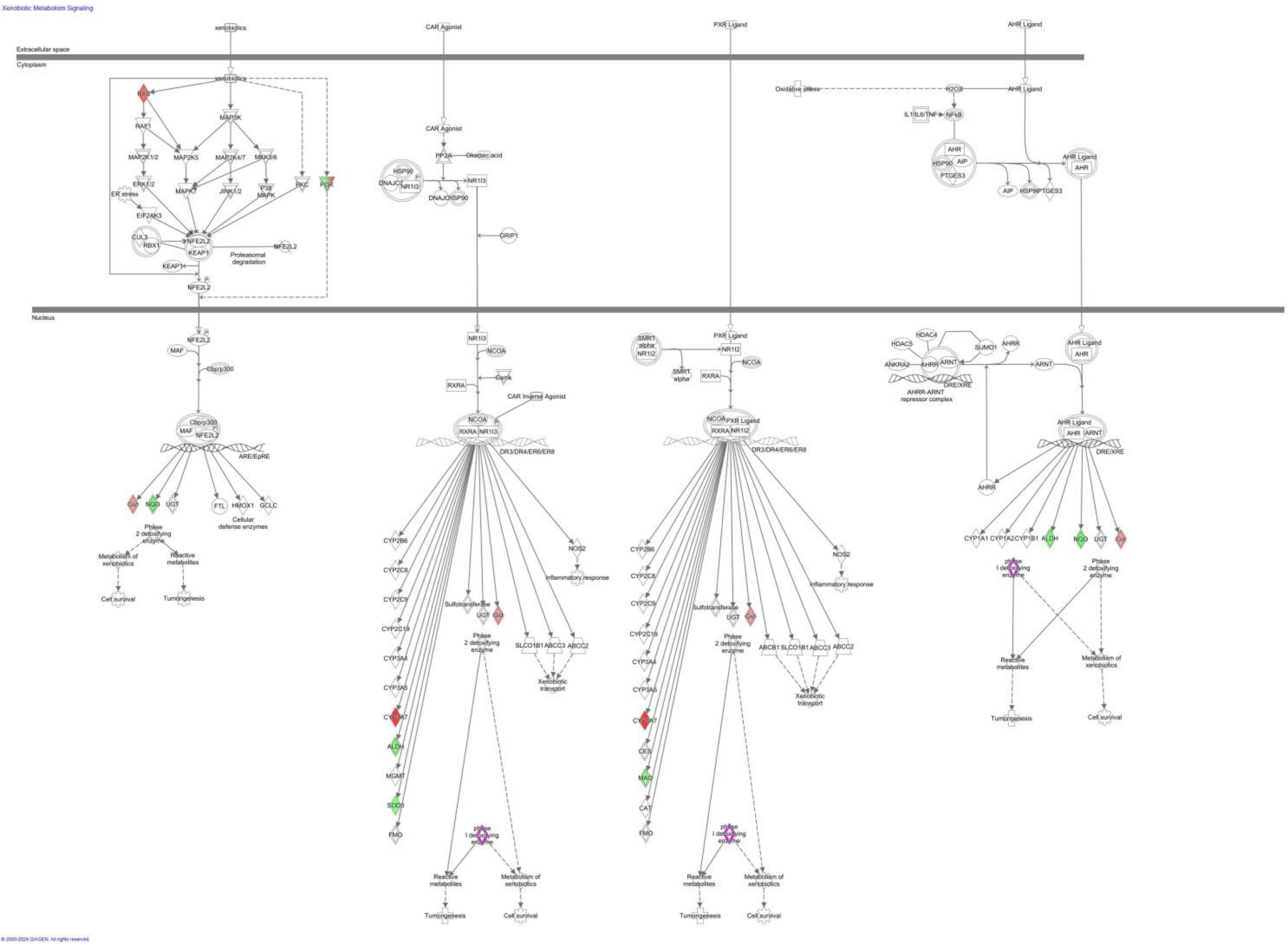

Figure S10

HIPPO Signaling

8 days
CAGE analysis

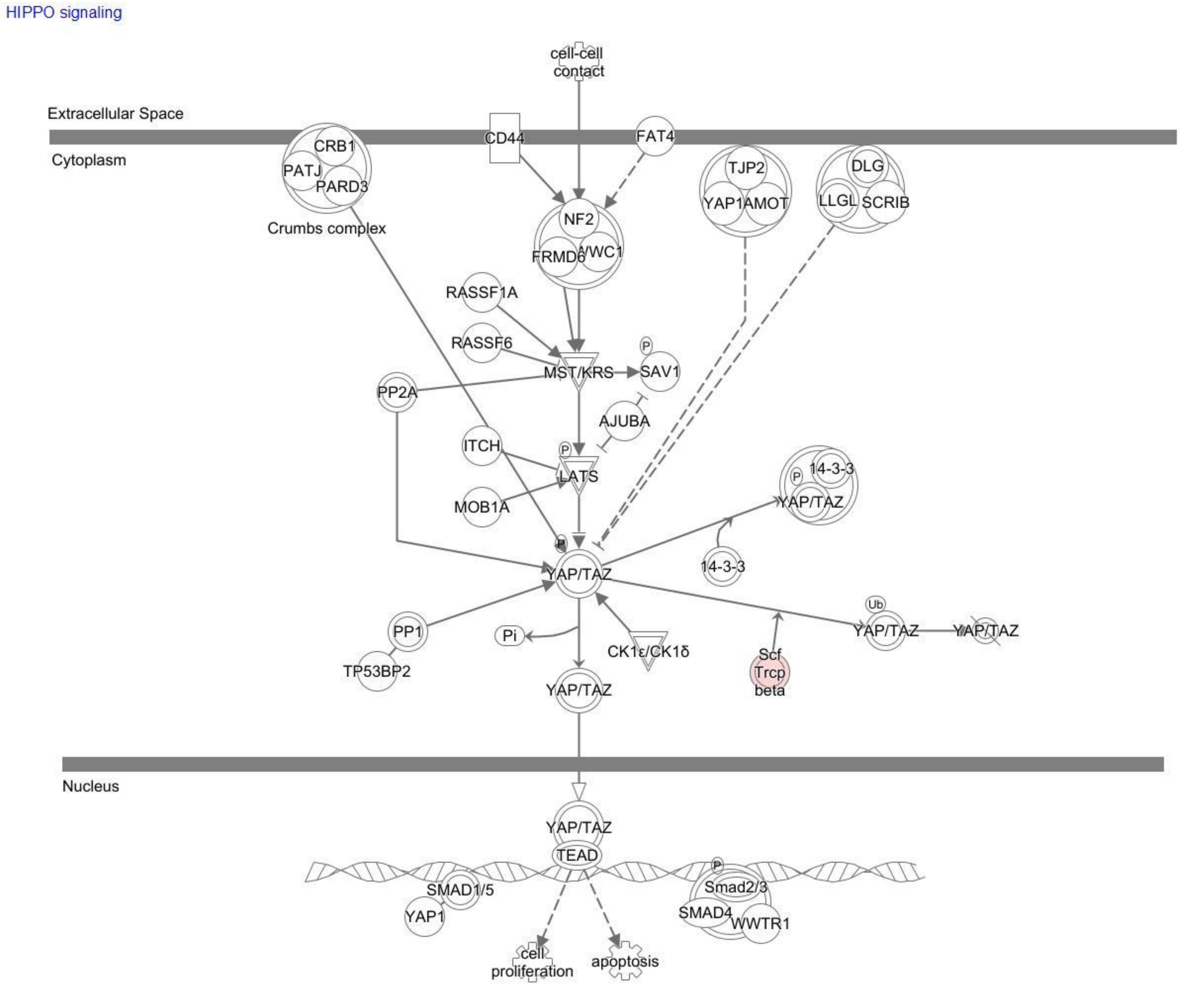

© 2000-2024 QIAGEN. All rights reserved.

Figure S11

HIPPO Signaling

Day 21
CAGE analysis

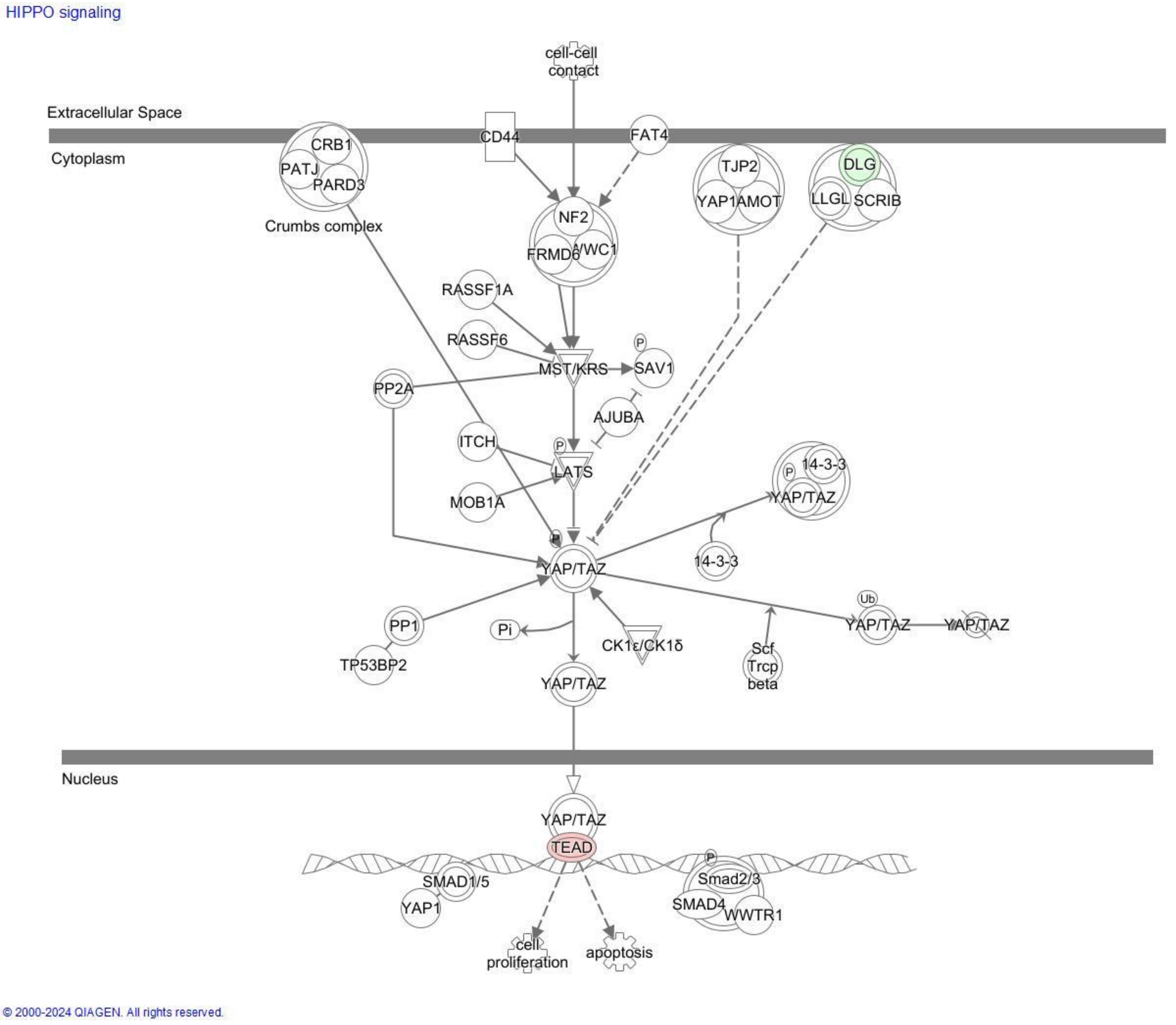

© 2000-2024 QIAGEN. All rights reserved.

Figure S12

Gap Junction Signaling

6 hours
CAGE analysis

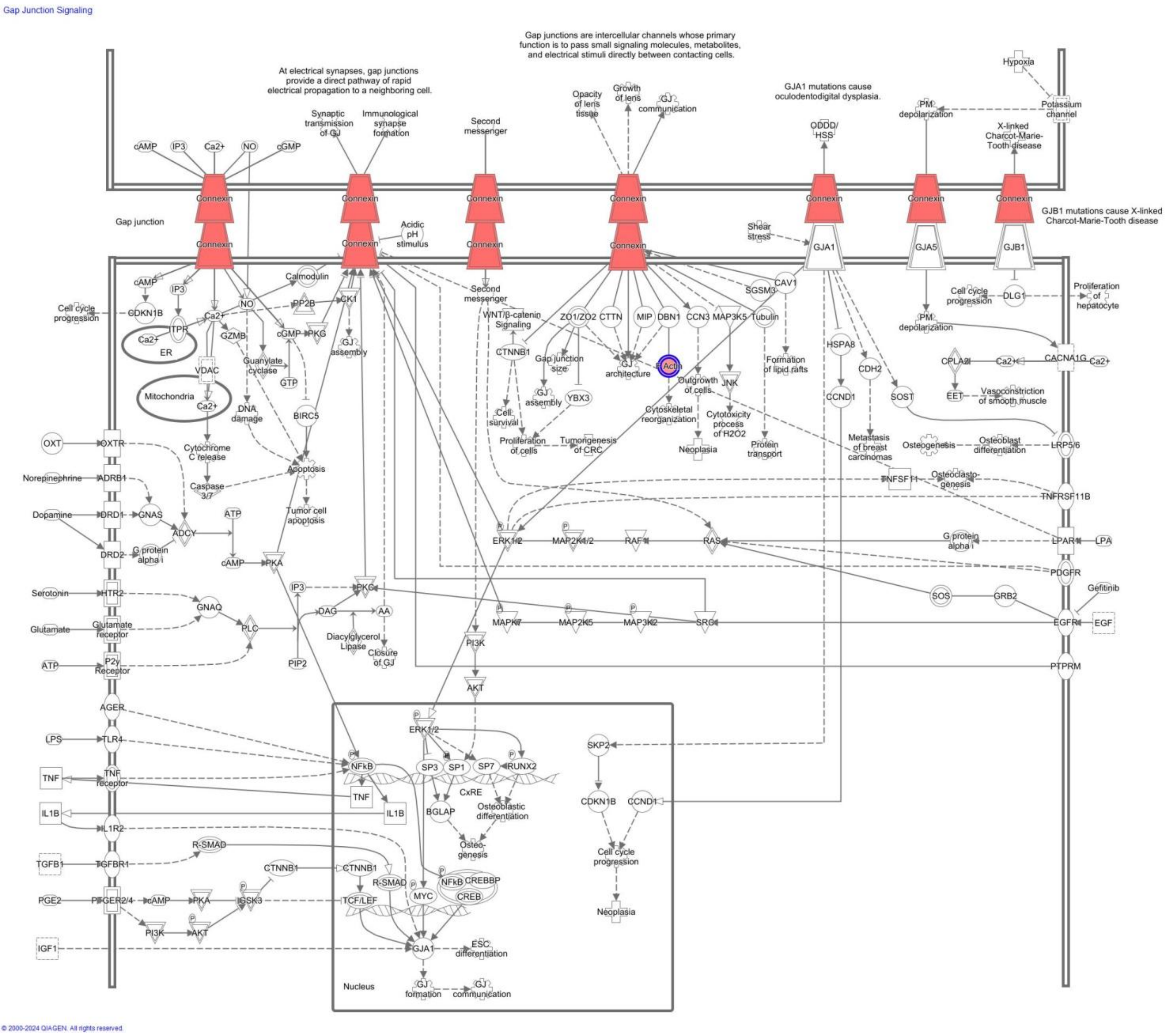

Figure S13

Gap Junction Signaling

8 days
CAGE analysis

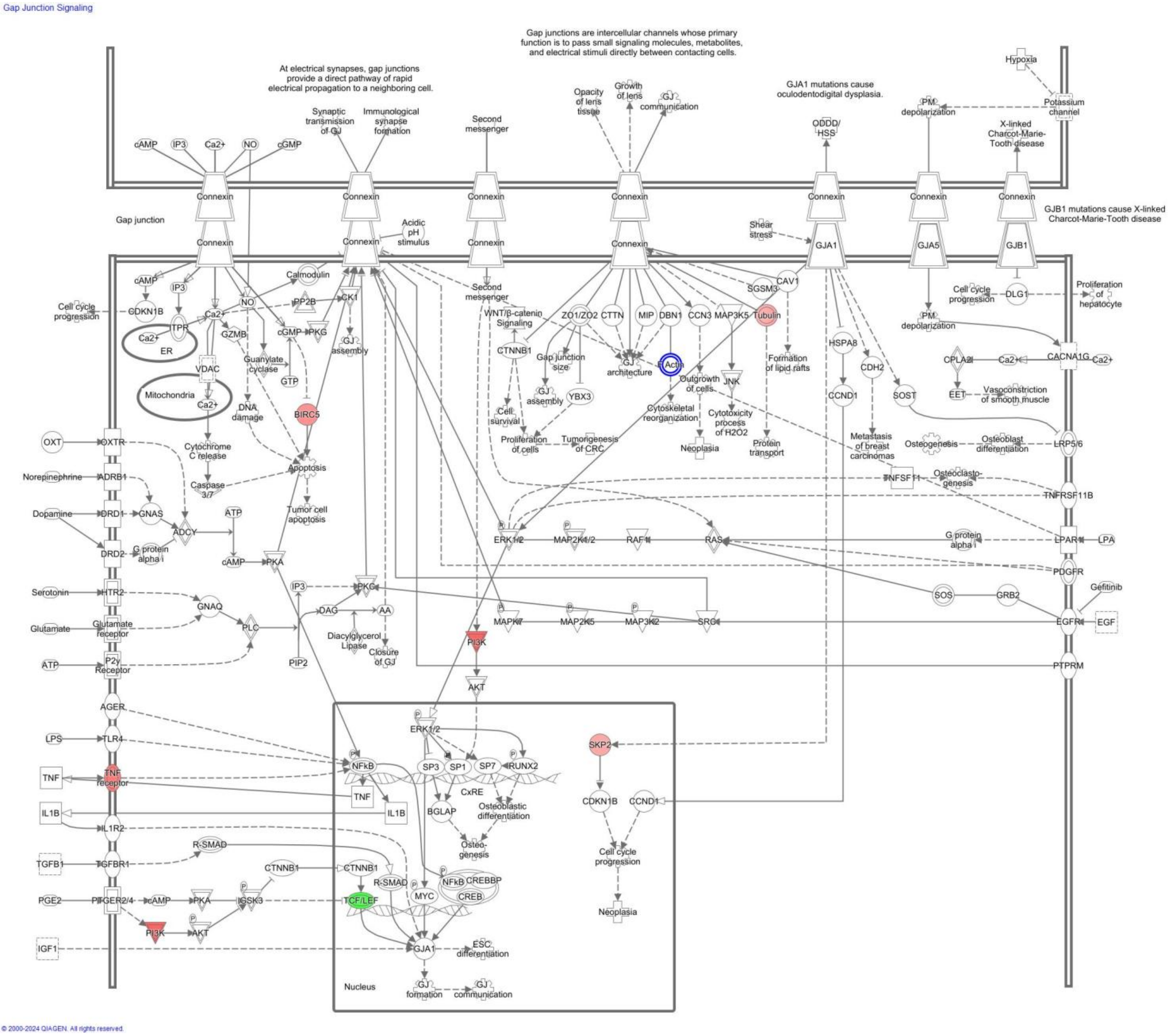

Figure S14

Gap Junction Signaling

Day 21
CAGE analysis

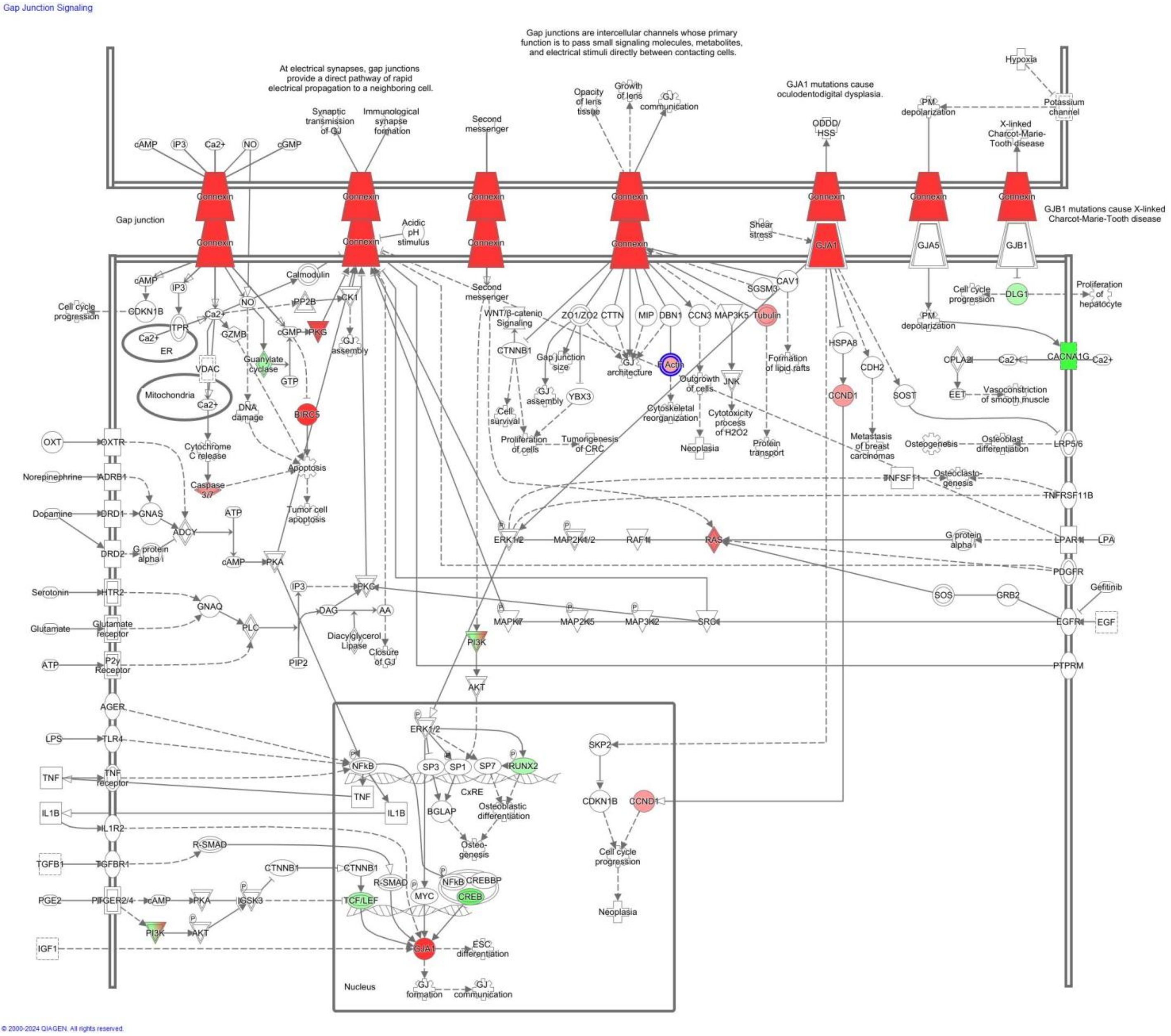

Figure S15

Apoptosis Signaling

1 hour
CAGE analysis

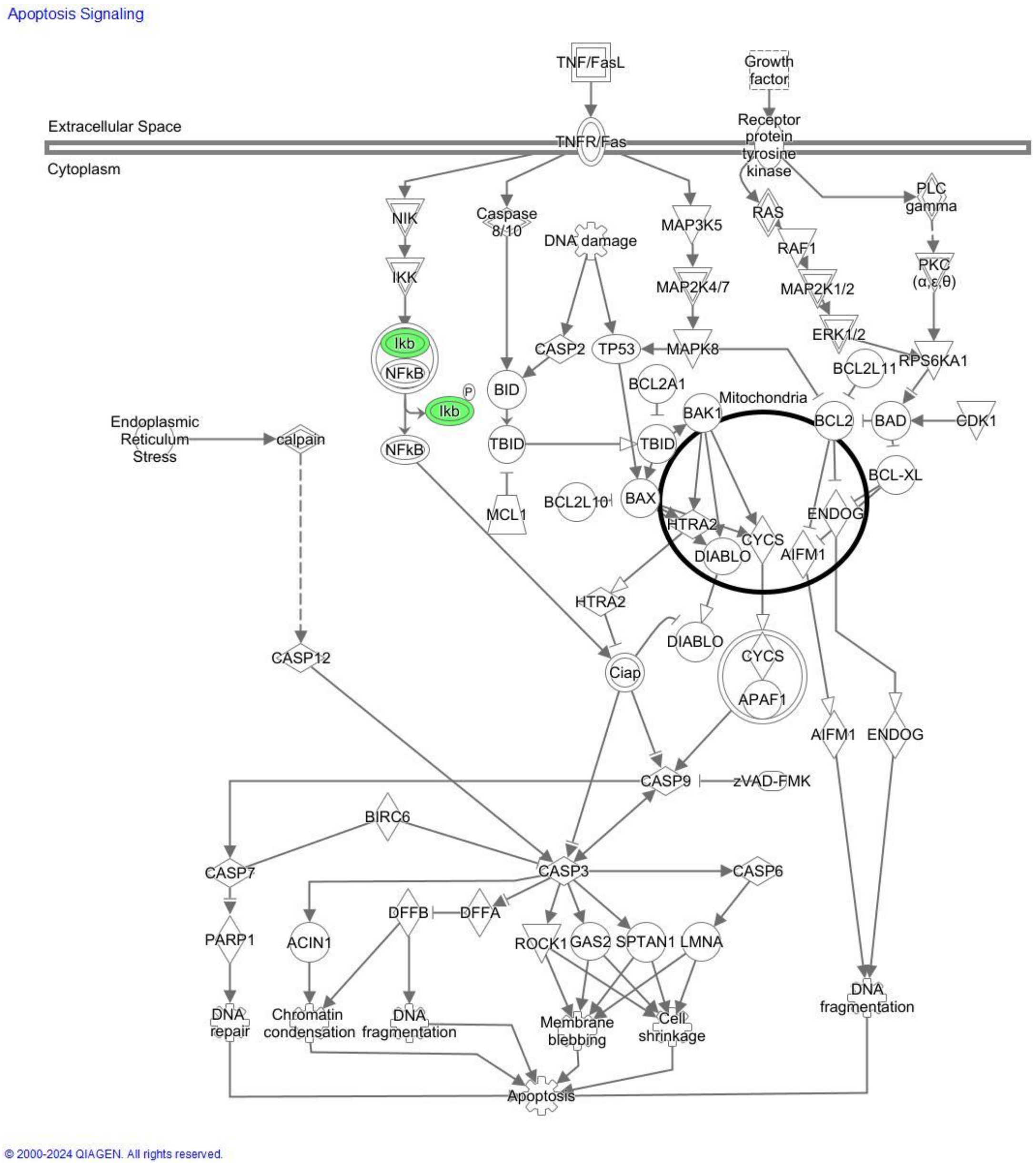

Figure S16

Apoptosis Signaling

6 hours
CAGE analysis

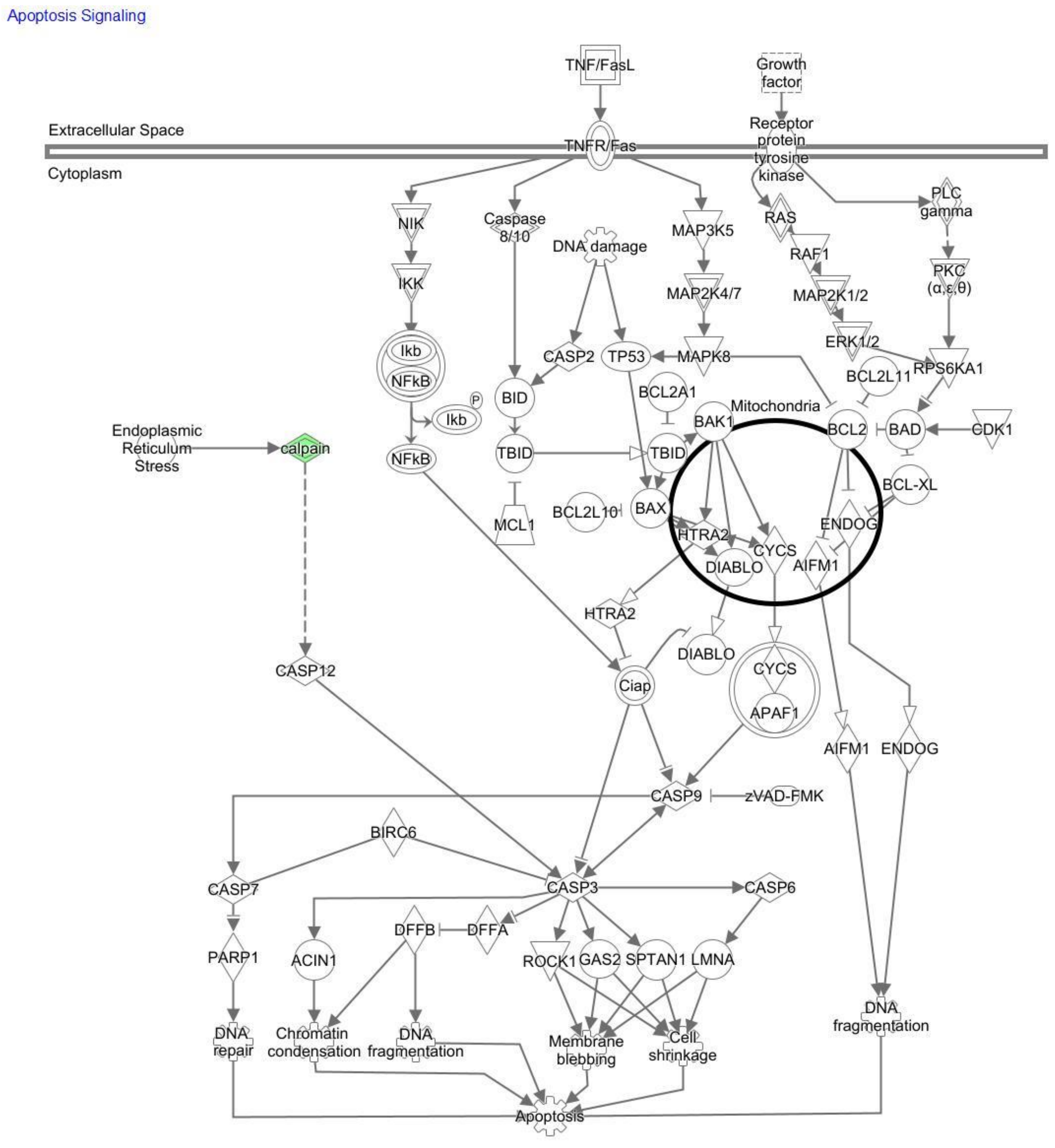

© 2000-2024 QIAGEN. All rights reserved.

Figure S17

Apoptosis Signaling

8 days
CAGE analysis

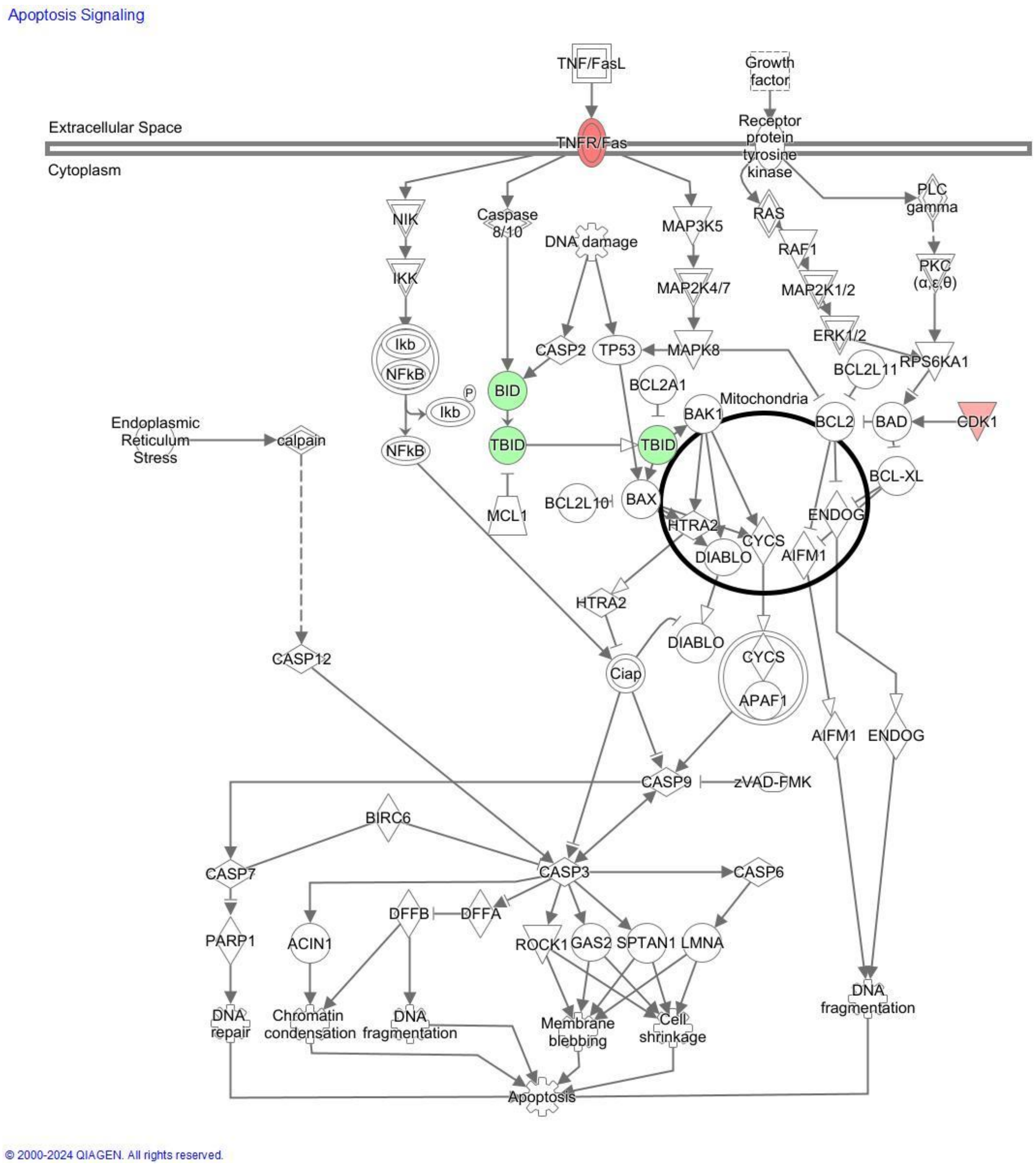

© 2000-2024 QIAGEN. All rights reserved.

Figure S18

Apoptosis Signaling

Day 21
CAGE analysis

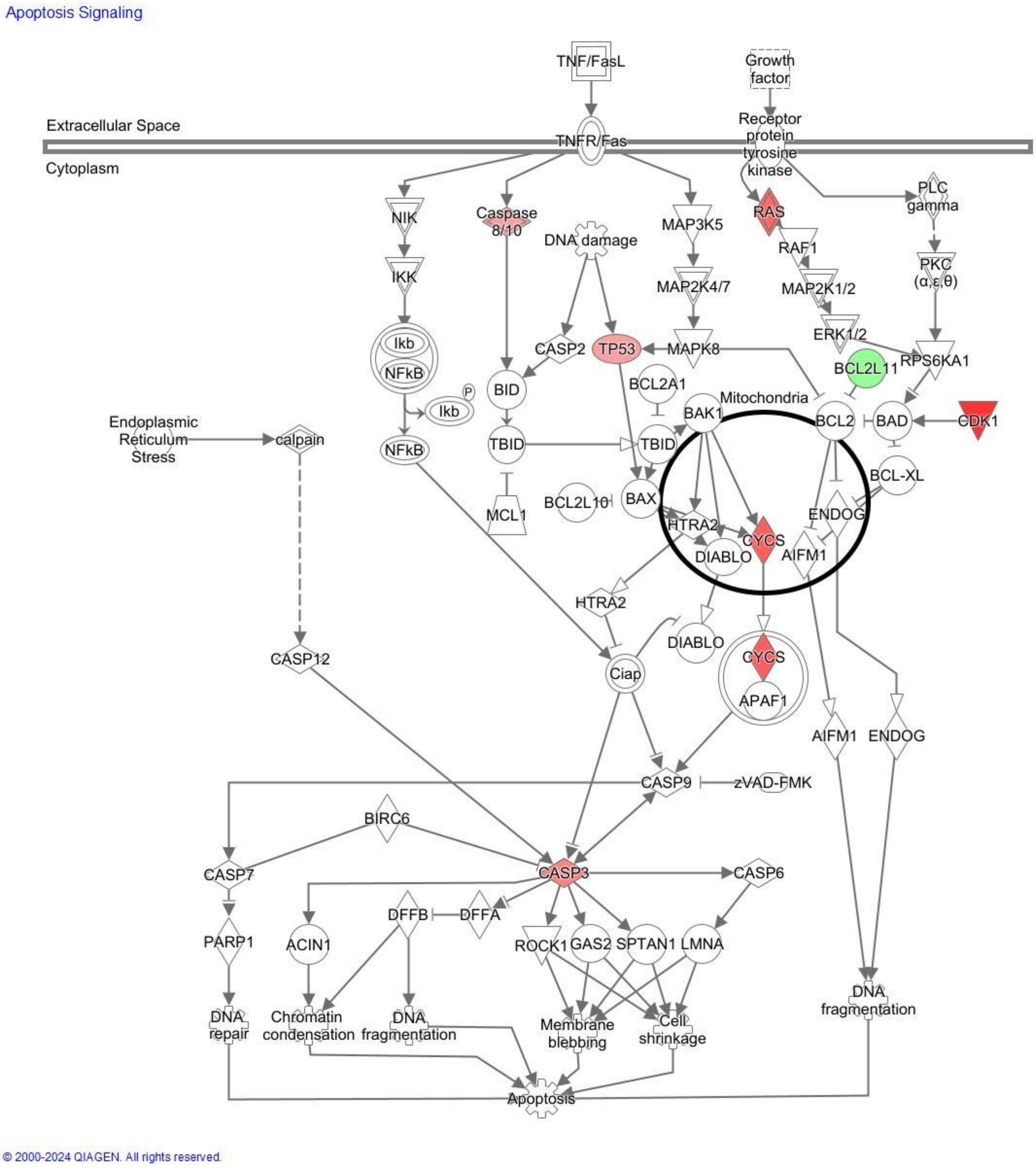

© 2000-2024 QIAGEN. All rights reserved.

Figure S19

Autophagy

8 days
CAGE analysis

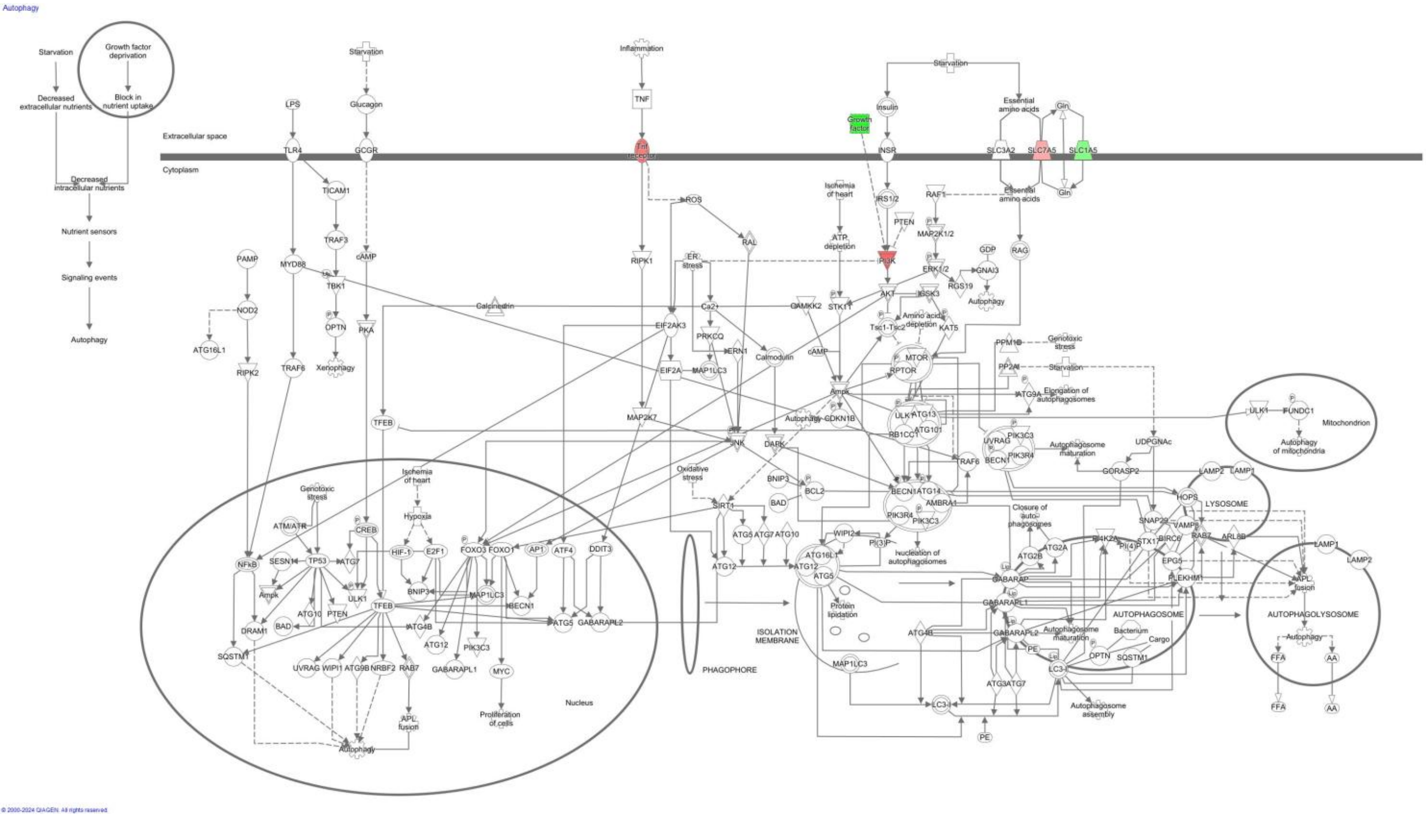

Figure S20

Autophagy

Day 21
CAGE analysis

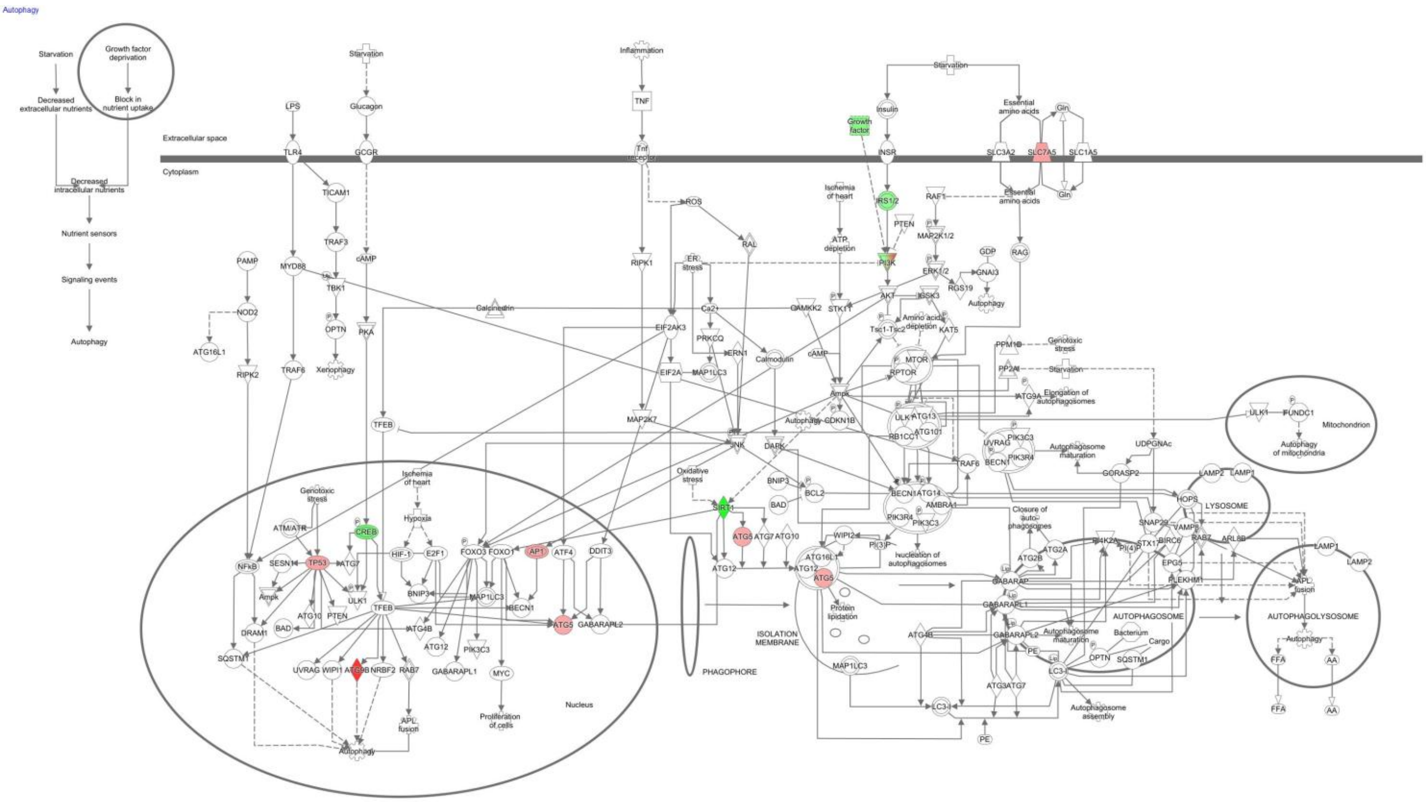

© 2000-2024 QIAGEN. All rights reserved.

Figure S21

Microautophagy Signaling Pathway

24 hours
CAGE analysis

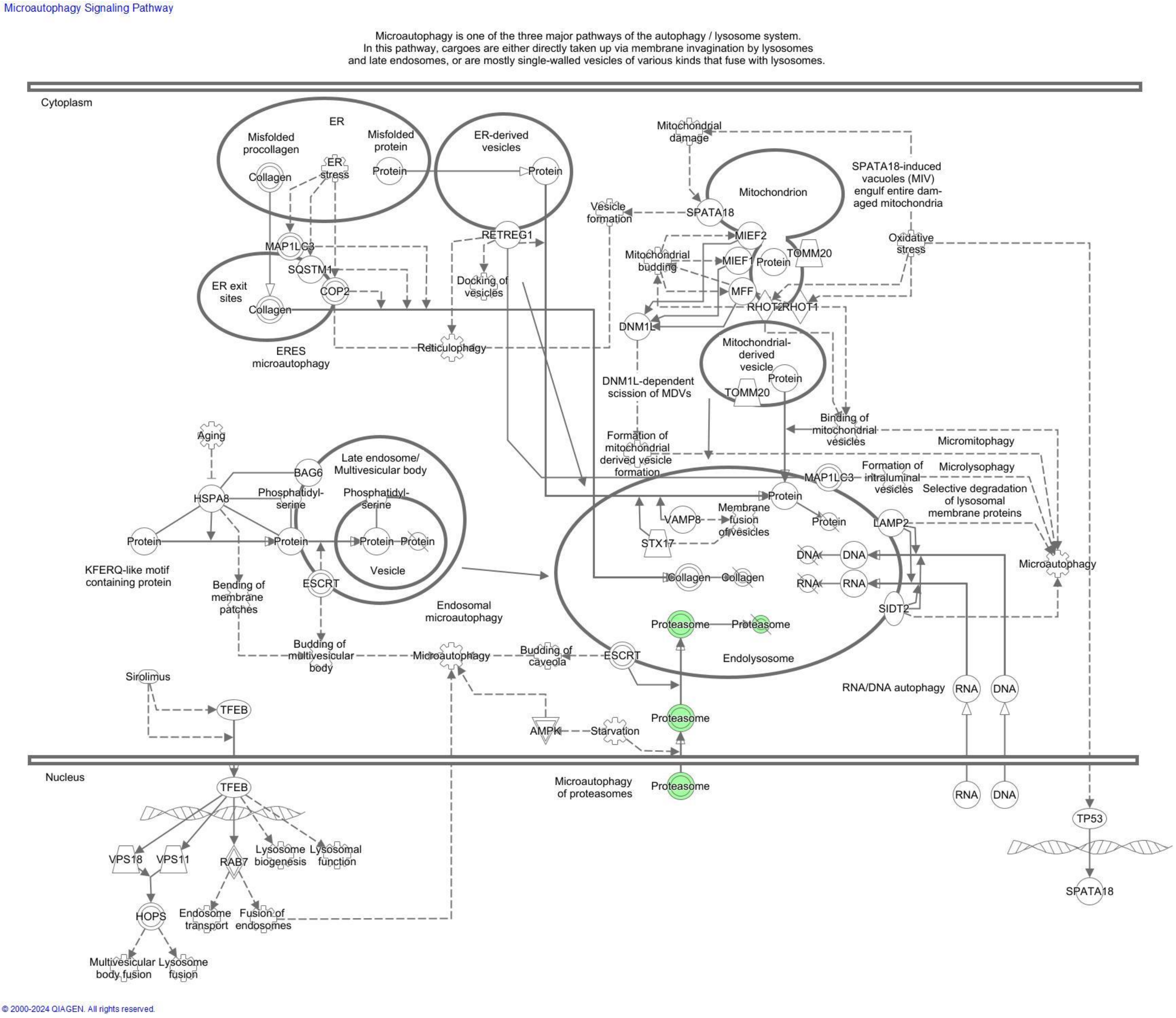

© 2000-2024 QIAGEN. All rights reserved.

Figure S22

Microautophagy Signaling Pathway

Day 21
CAGE analysis

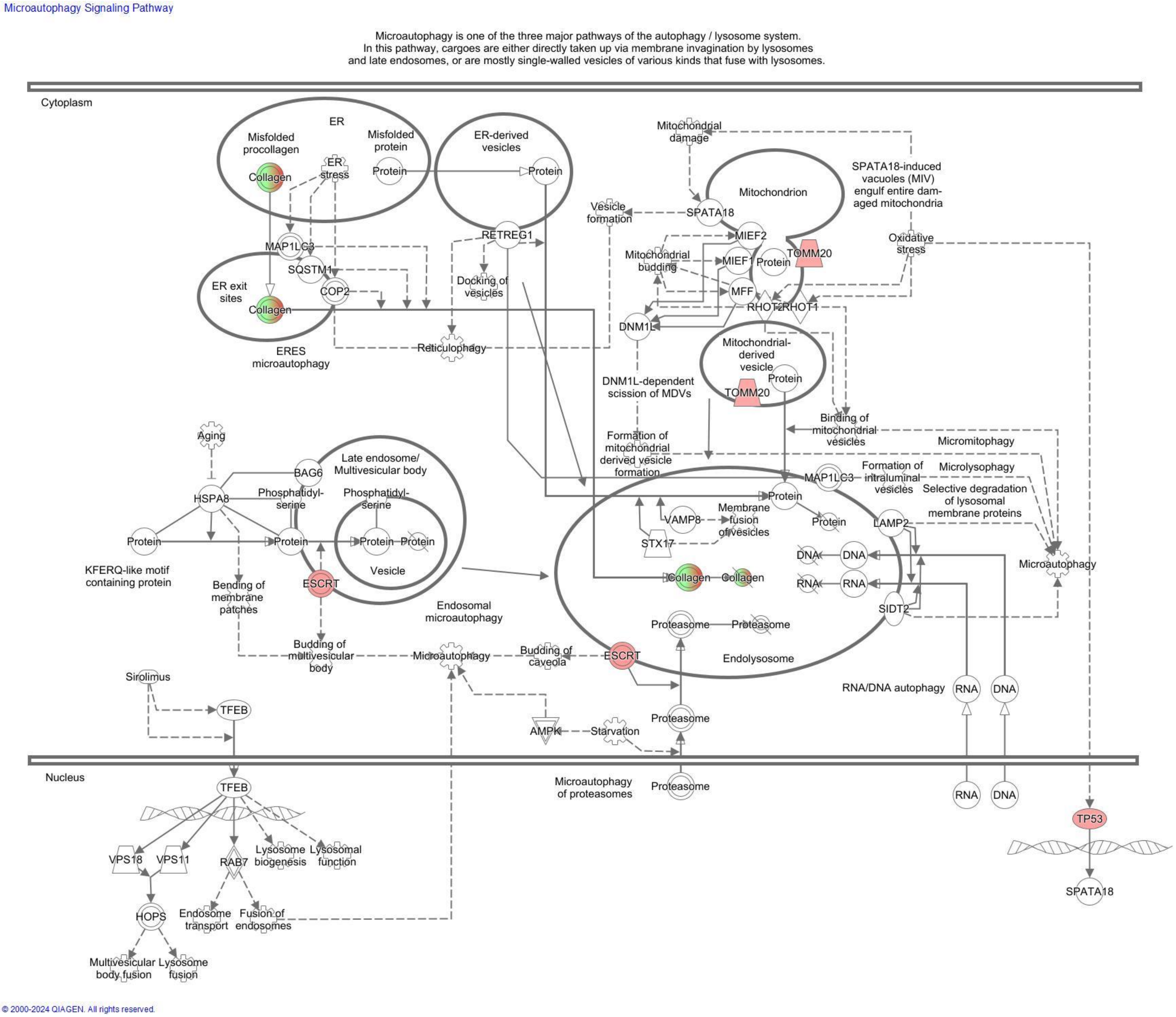

© 2000-2024 QIAGEN. All rights reserved.

Figure S23

Immunogenic Cell Death Signaling

24 hours
CAGE analysis

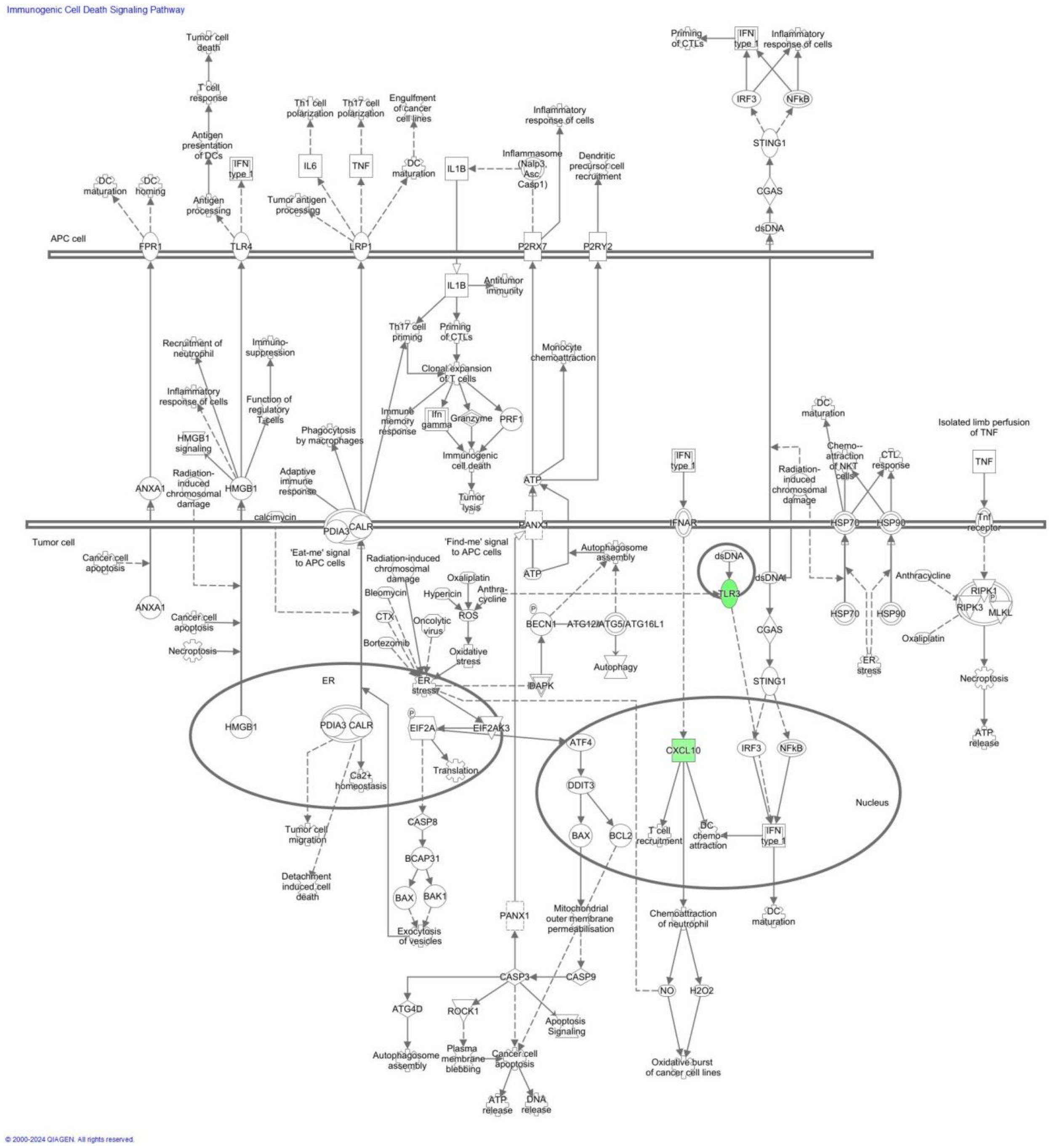

Figure S24

Immunogenic Cell Death Signaling

8 days
CAGE analysis

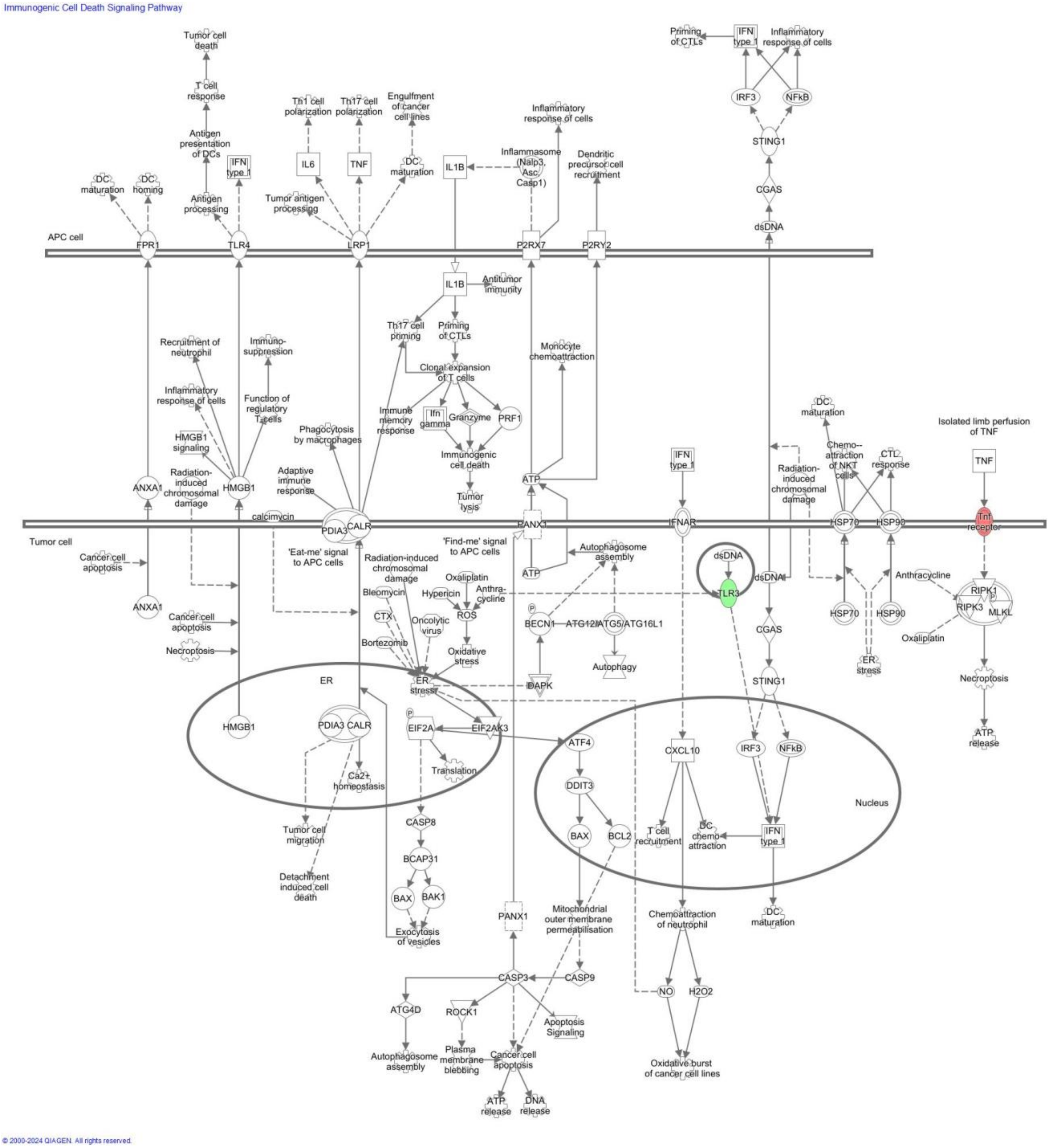

Figure S25

Immunogenic Cell Death Signaling

Day 21
CAGE analysis

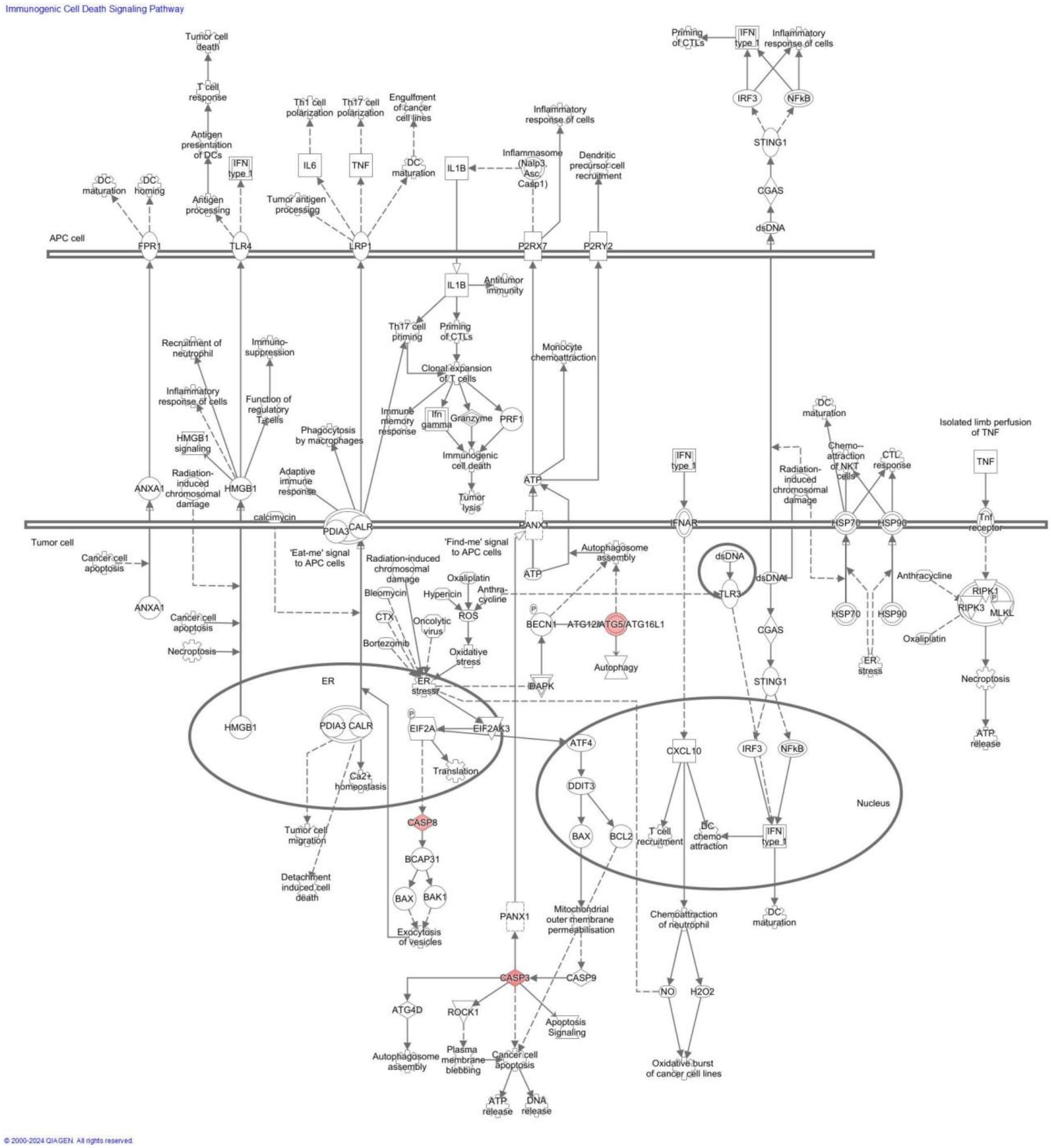

Figure S26

Interferon Signaling

24 hours
CAGE analysis

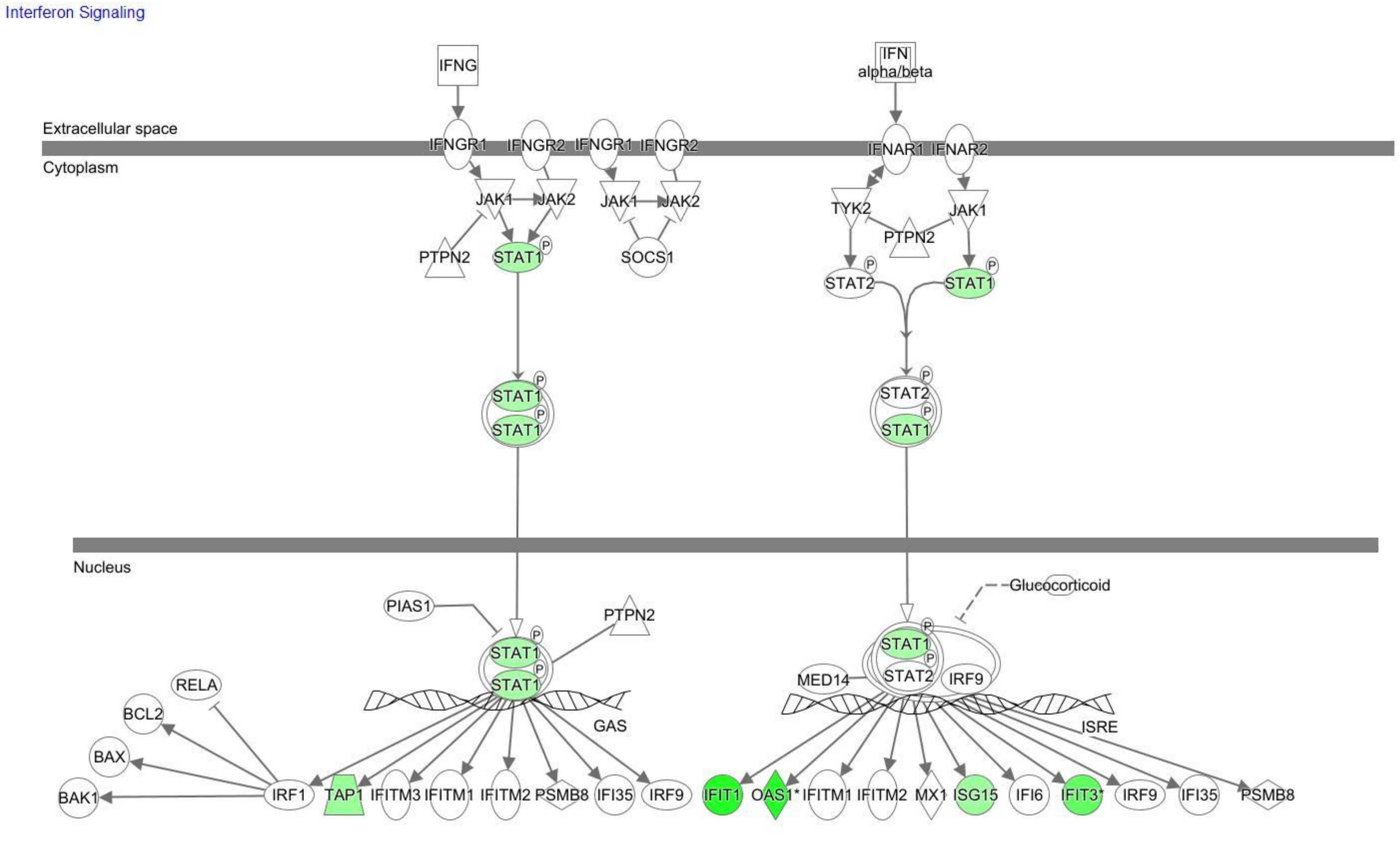

© 2000-2024 QIAGEN. All rights reserved.

Figure S27

Interferon Signaling

8 days
CAGE analysis

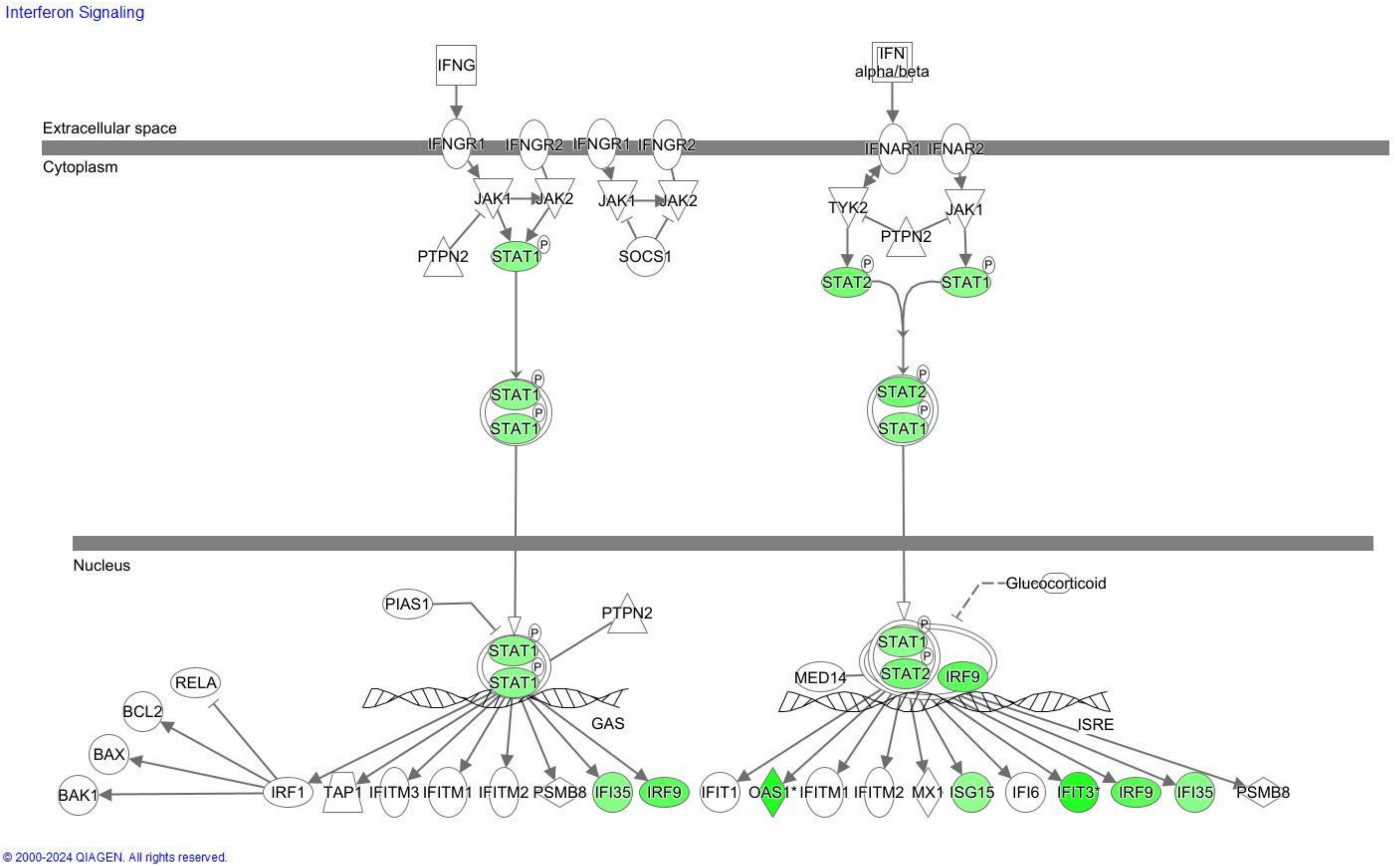

© 2000-2024 QIAGEN. All rights reserved.

Figure S28

PD-1, PDL-1 cancer immunotherapy pathway

8 days
CAGE analysis

PD-1, PD-L1 cancer immunotherapy pathway

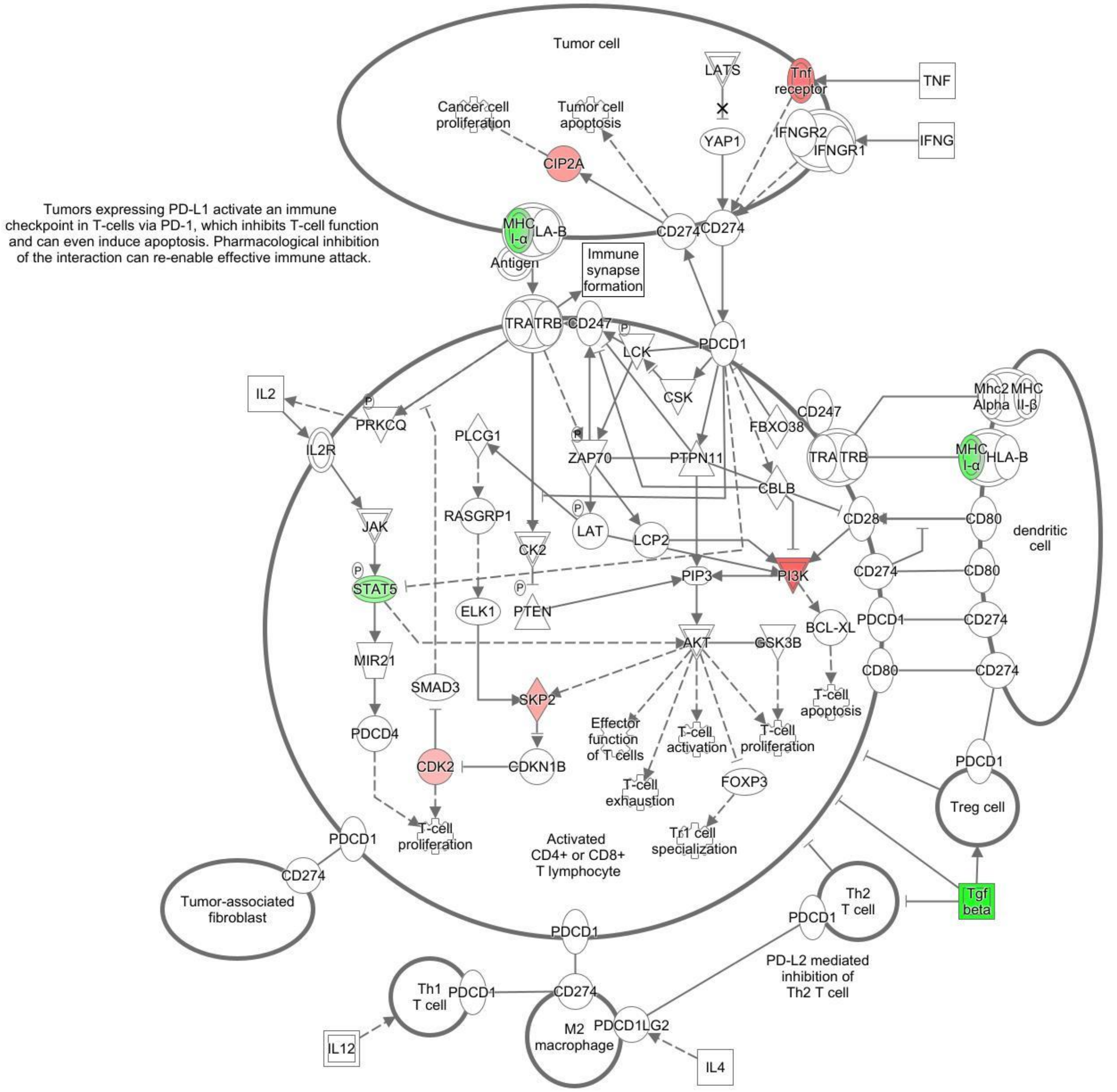

© 2000-2024 QIAGEN. All rights reserved.

Figure S29

PD-1, PDL-1 cancer immunotherapy pathway

Day 21
CAGE analysis

PD-1, PD-L1 cancer immunotherapy pathway

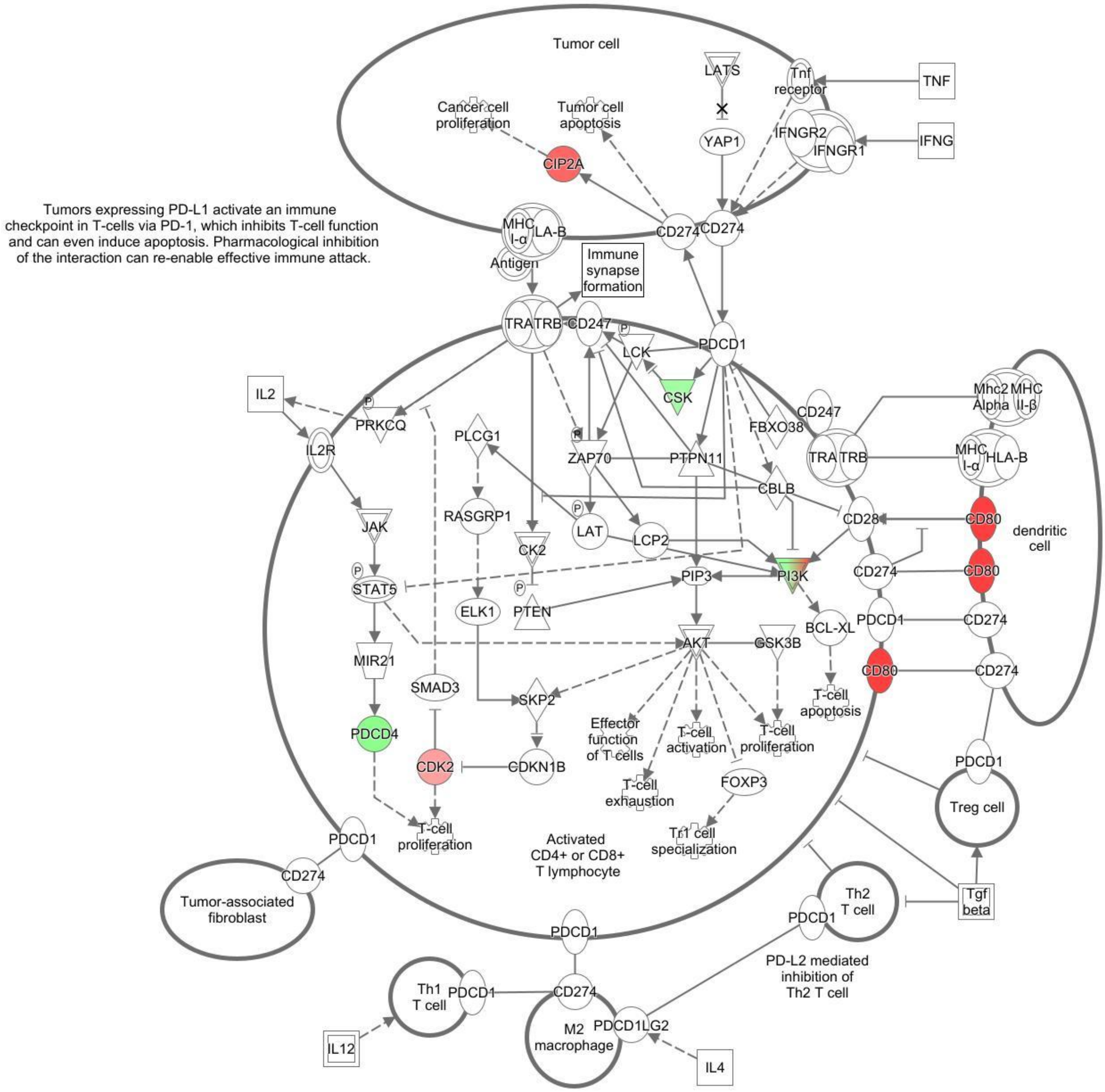

© 2000-2024 QIAGEN. All rights reserved.

Figure S30

IL-1 Signaling

1 hour
CAGE analysis

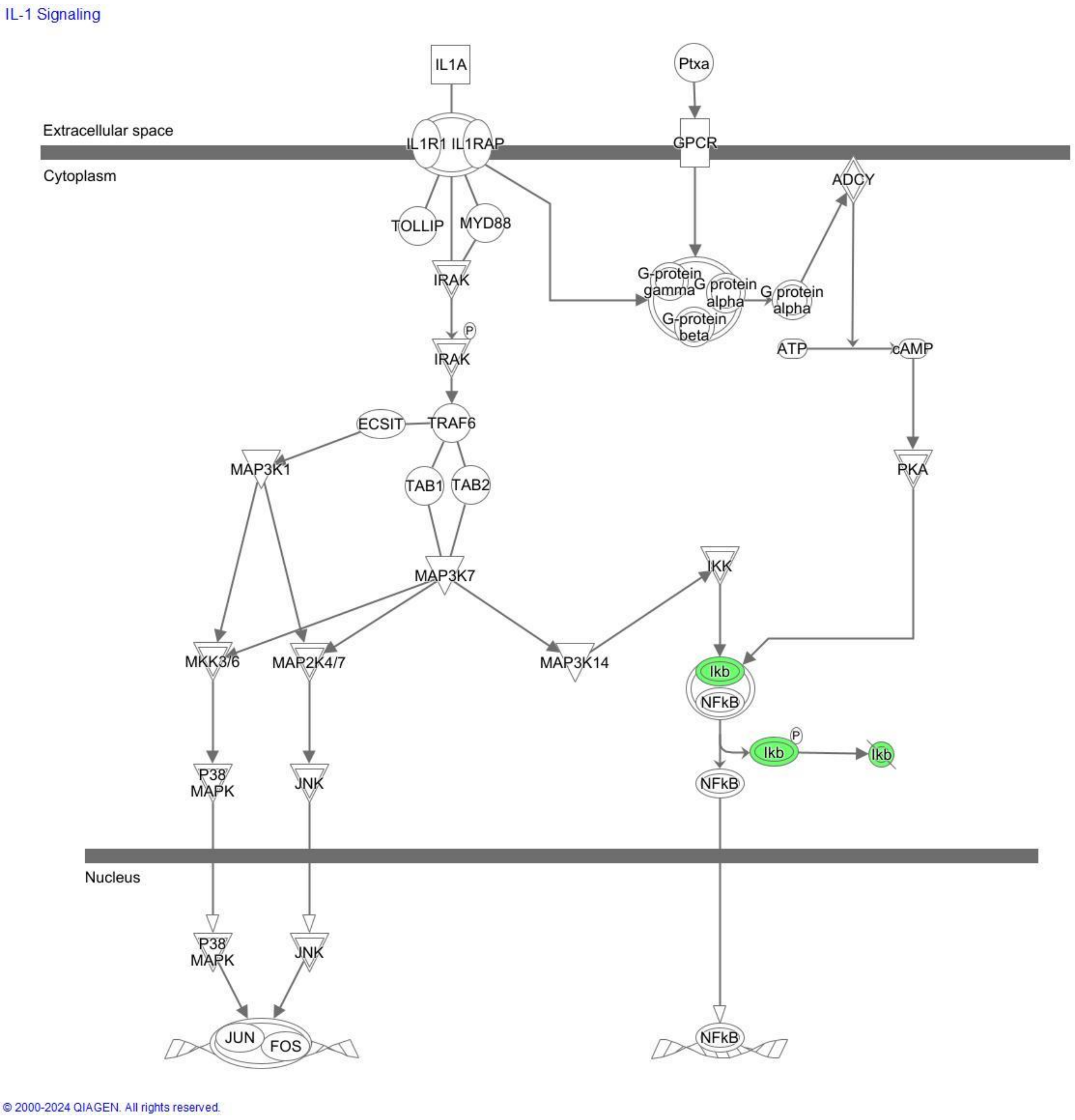

Figure S31

IL-1 Signaling

Day 21
CAGE analysis

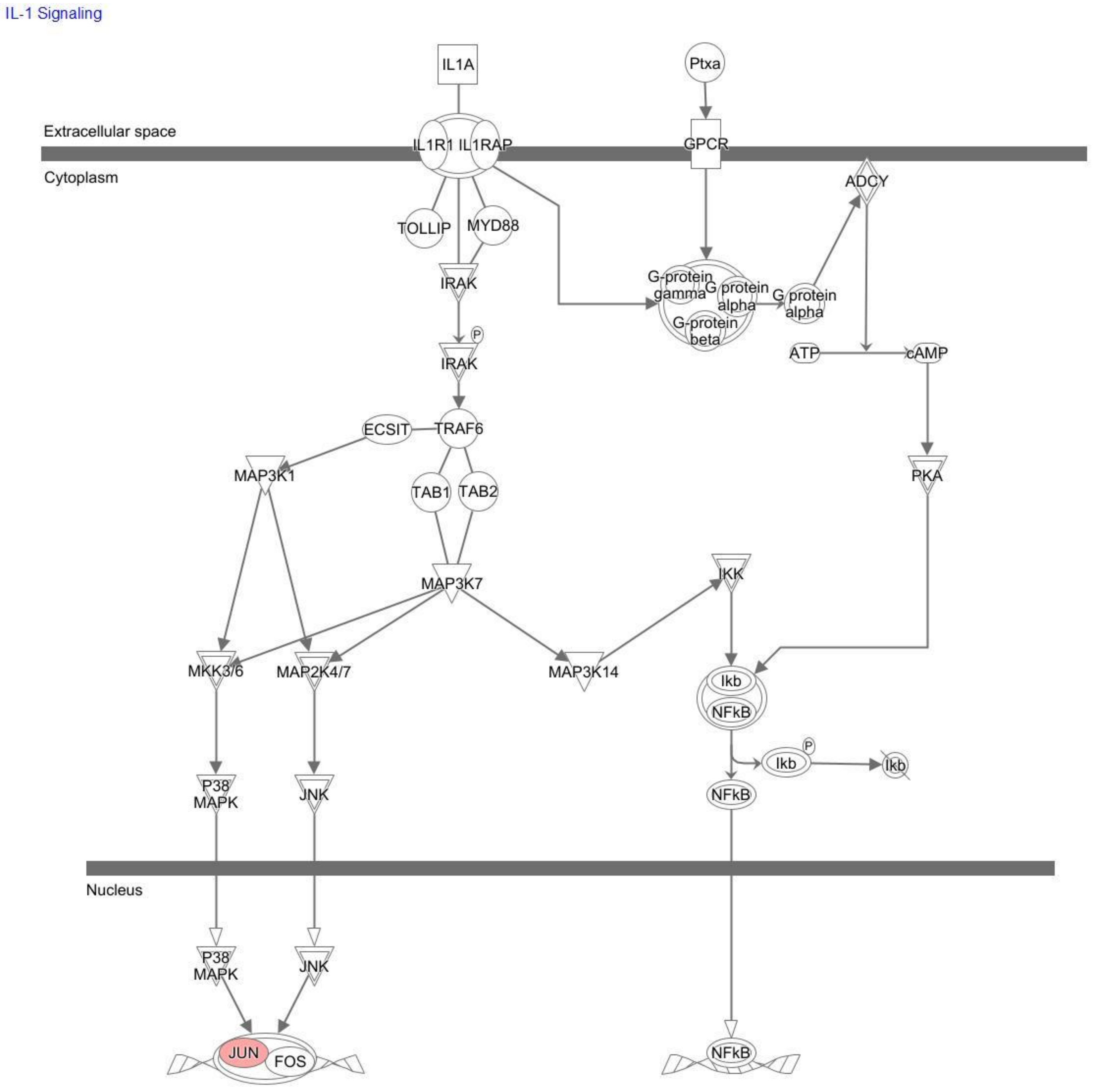

© 2000-2024 QIAGEN. All rights reserved.

Figure S32

Activin Inhibin Signaling Pathway

1 hour
CAGE analysis

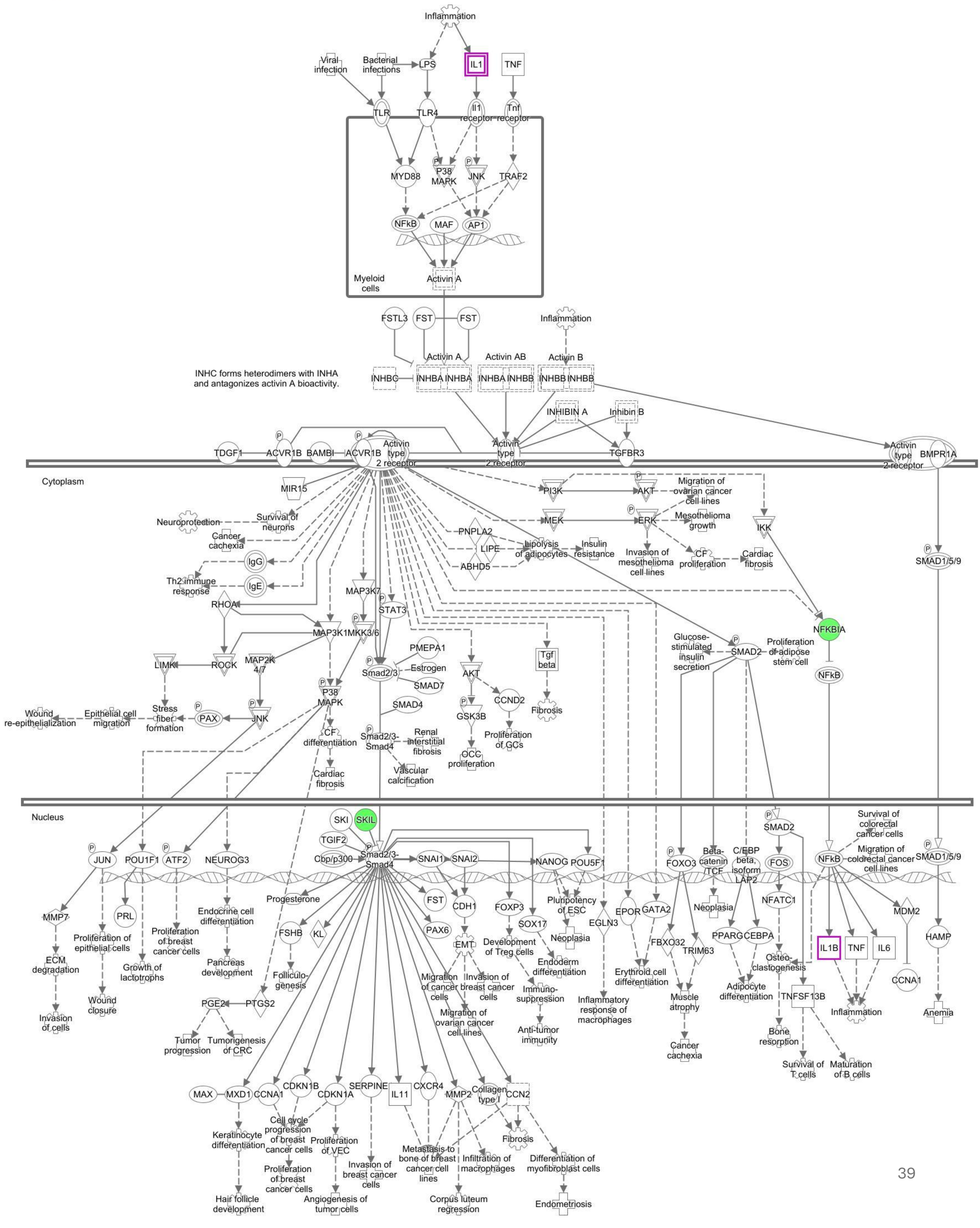

© 2000-2024 QIAGEN. All rights reserved.

Figure S33

Activin Inhibin Signaling Pathway

6 hour
CAGE analysis

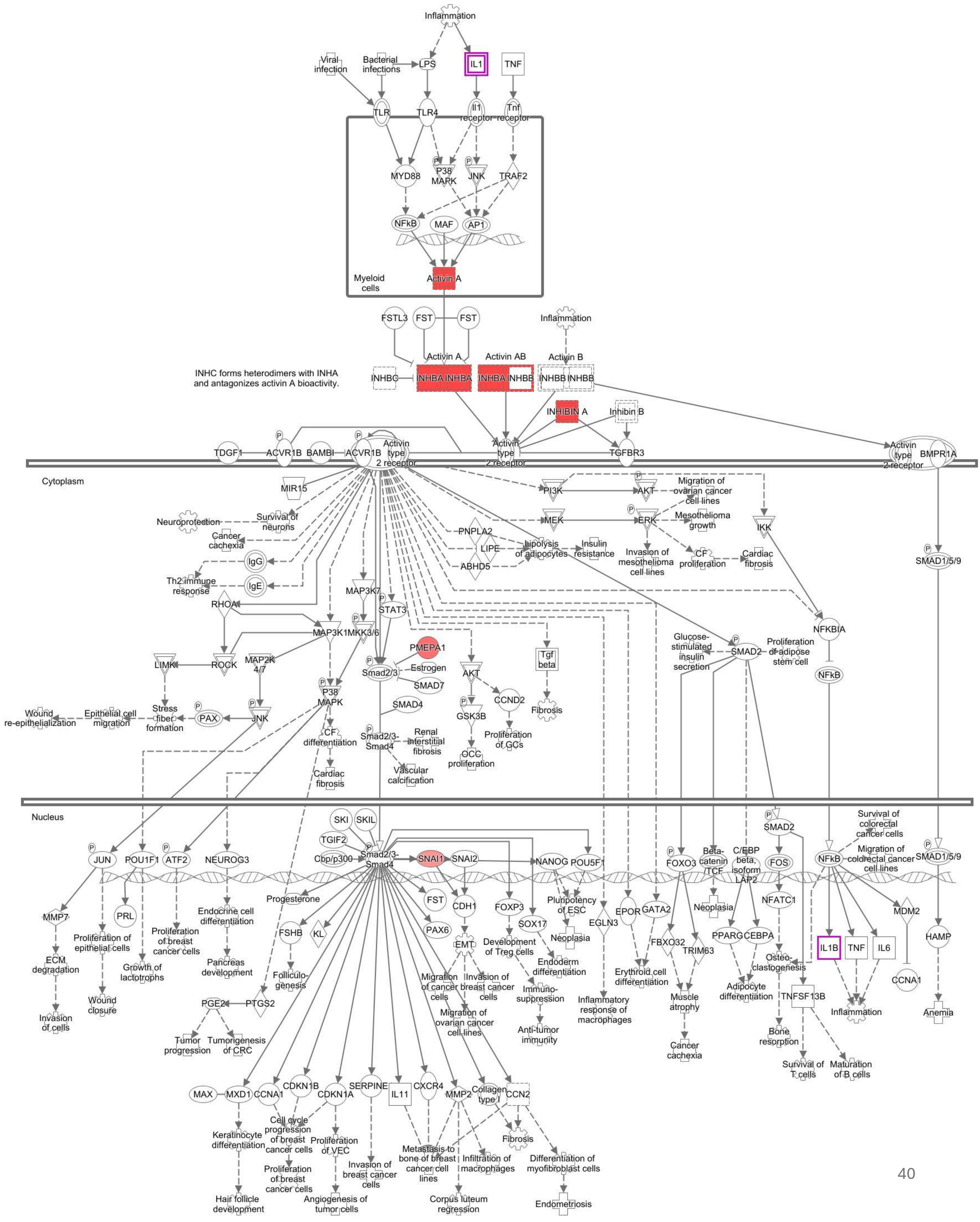

© 2000-2024 QIAGEN. All rights reserved.

Figure S34

Activin Inhibin Signaling Pathway

24 hour
CAGE analysis

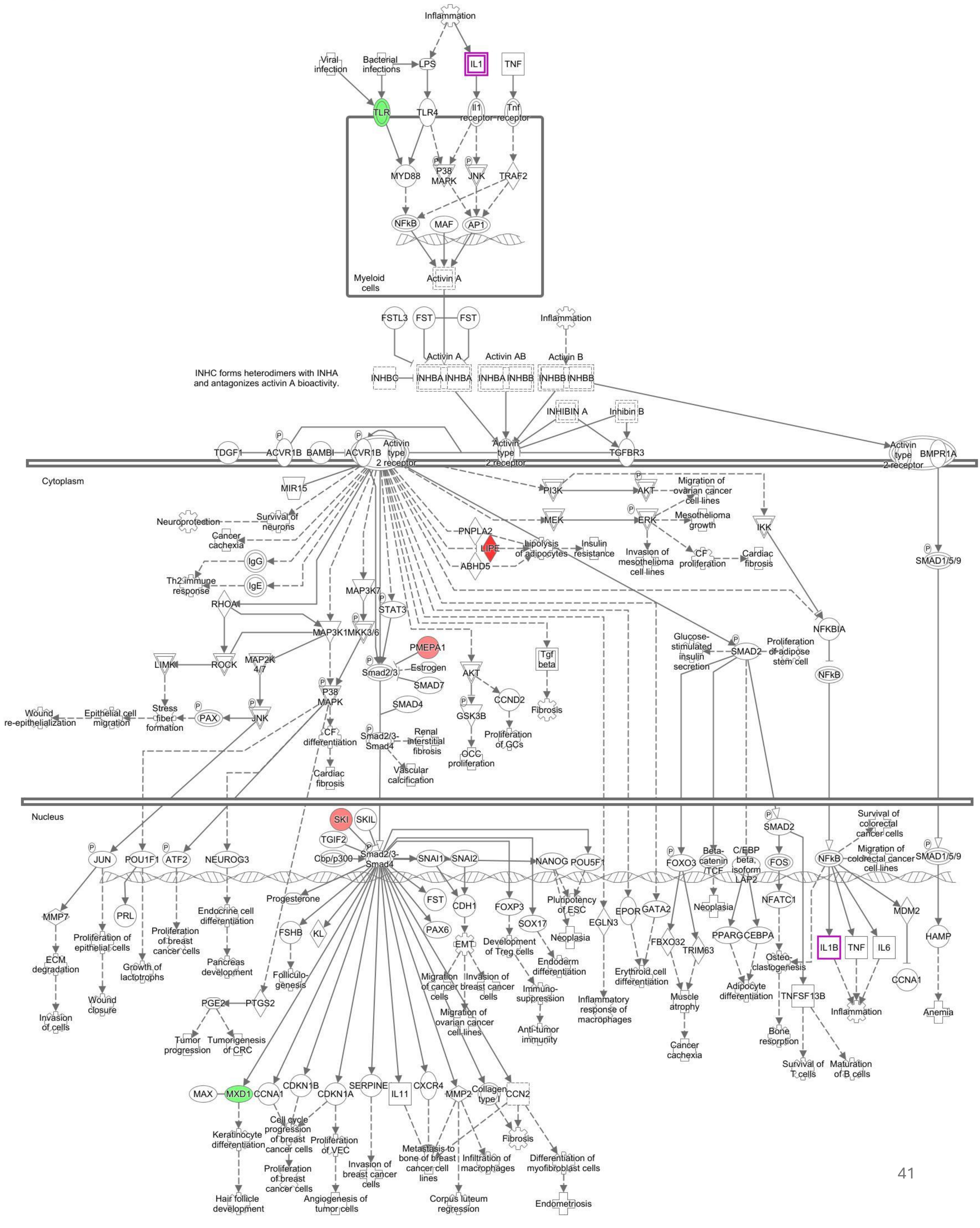

© 2000-2024 QIAGEN. All rights reserved.

Figure S35

Activin Inhibin Signaling Pathway

8 daysr
CAGE analysis

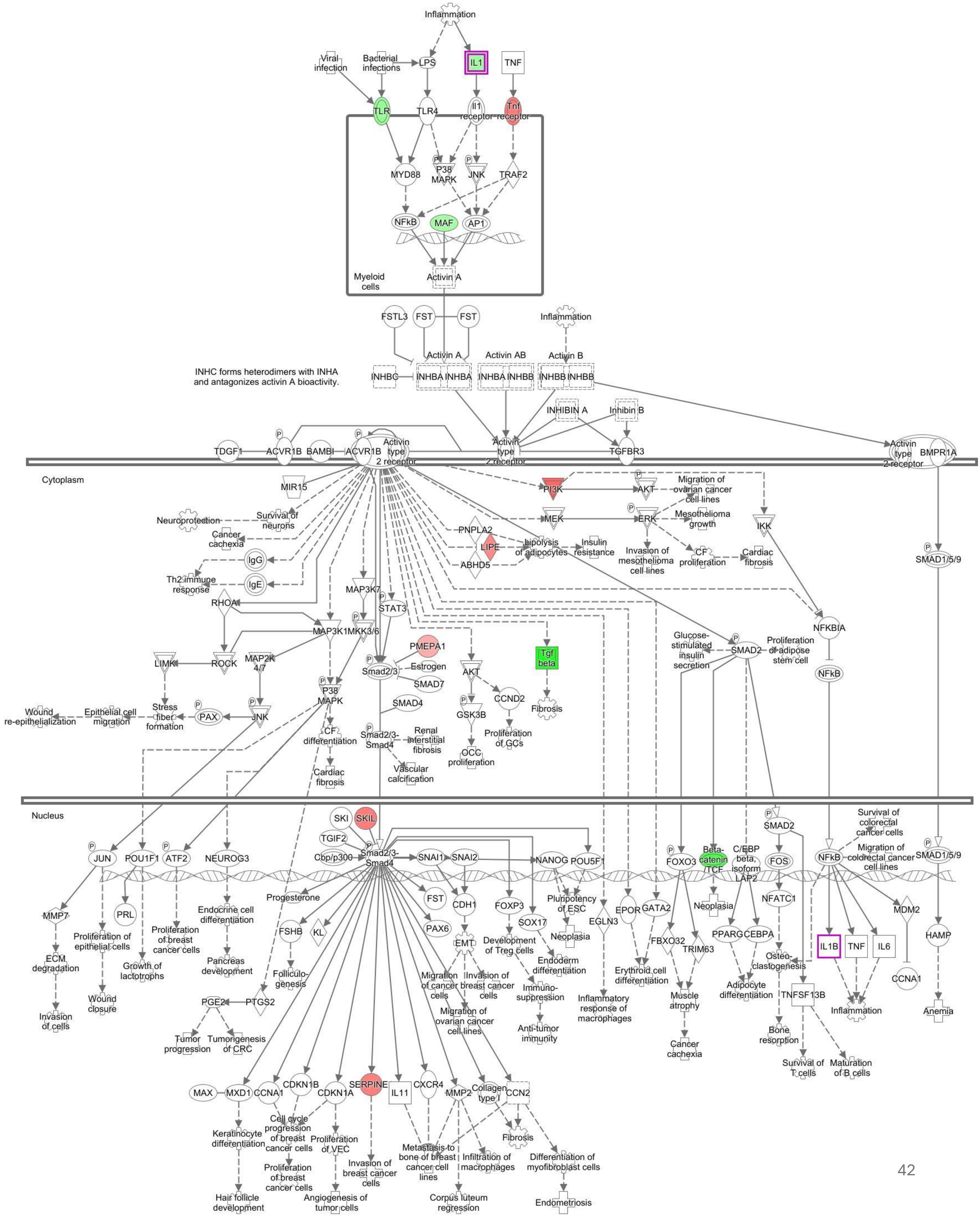

© 2000-2024 QIAGEN. All rights reserved.

Figure S36

Activin Inhibin Signaling Pathway

Day 21
CAGE analysis

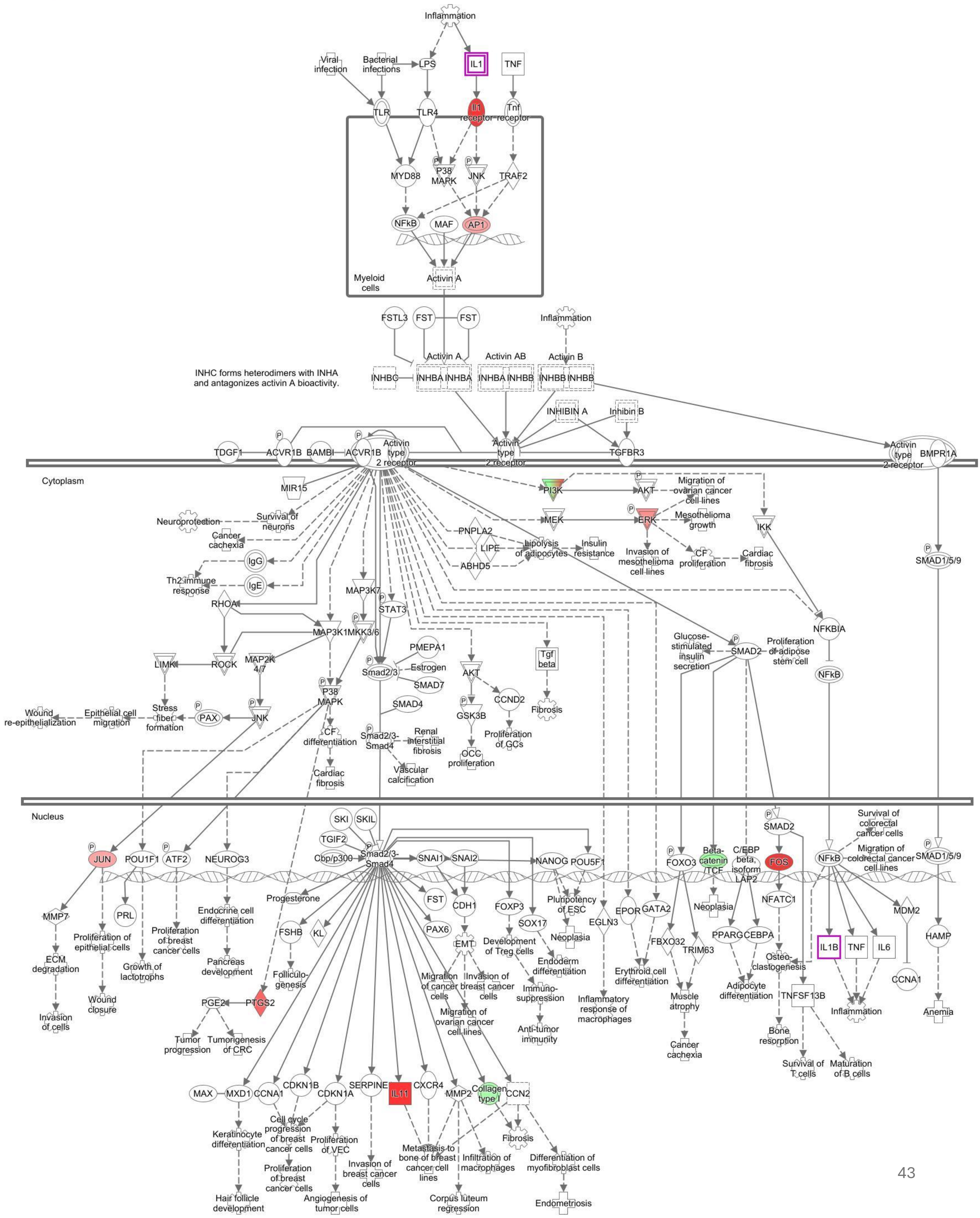

© 2000-2024 QIAGEN. All rights reserved.

Figure S37

IL-4 Signaling

24 hours
CAGE analysis

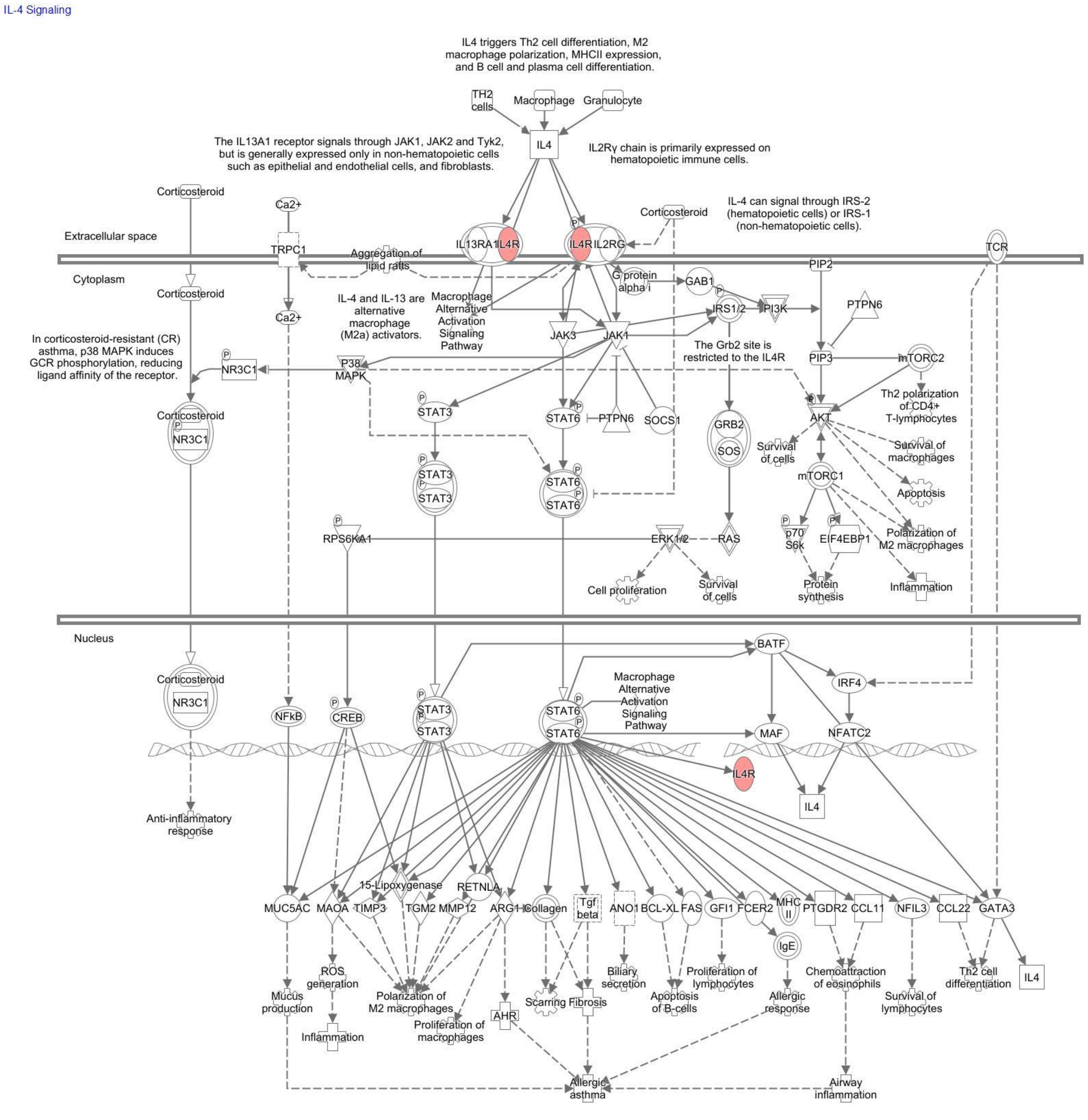

© 2000-2024 QIAGEN. All rights reserved.

Figure S38

IL-4 Signaling

8 days
CAGE analysis

IL-4 Signaling

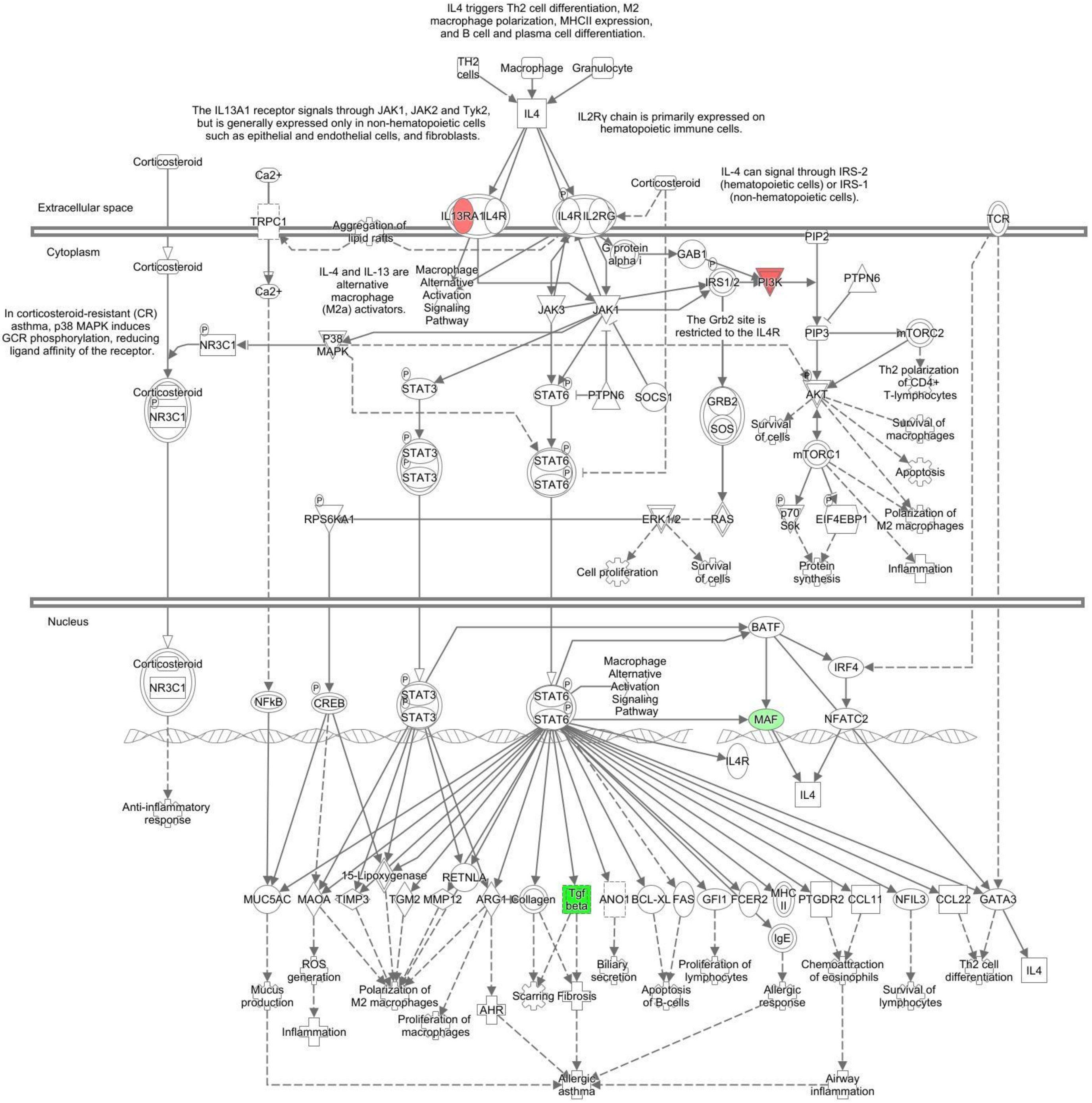

© 2000-2024 QIAGEN. All rights reserved.

Figure S39

IL-4 Signaling

Day 21
CAGE analysis

IL-4 Signaling

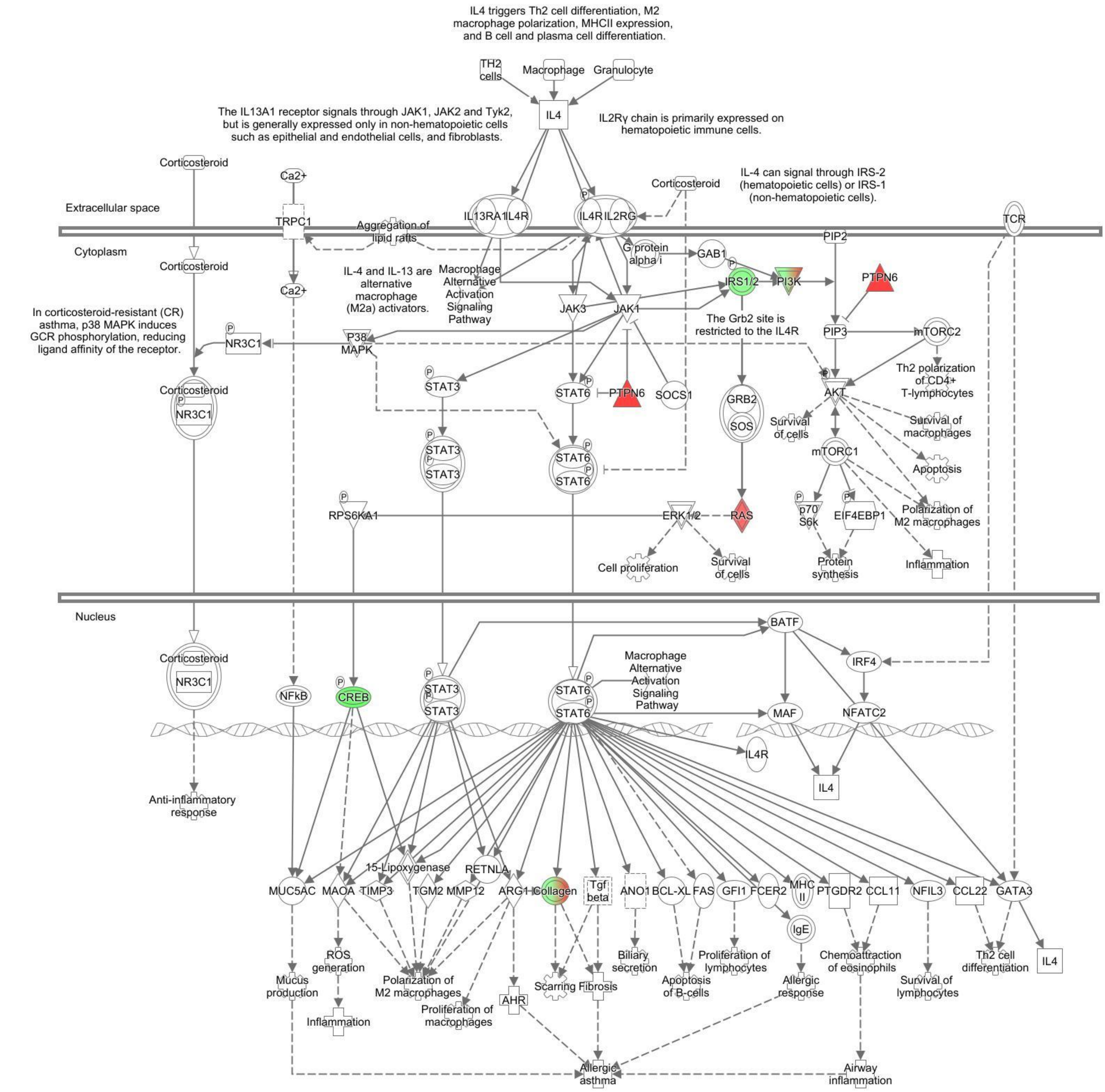

© 2000-2024 QIAGEN. All rights reserved.

Figure S40

IL-6 Signaling

1 hour
CAGE analysis

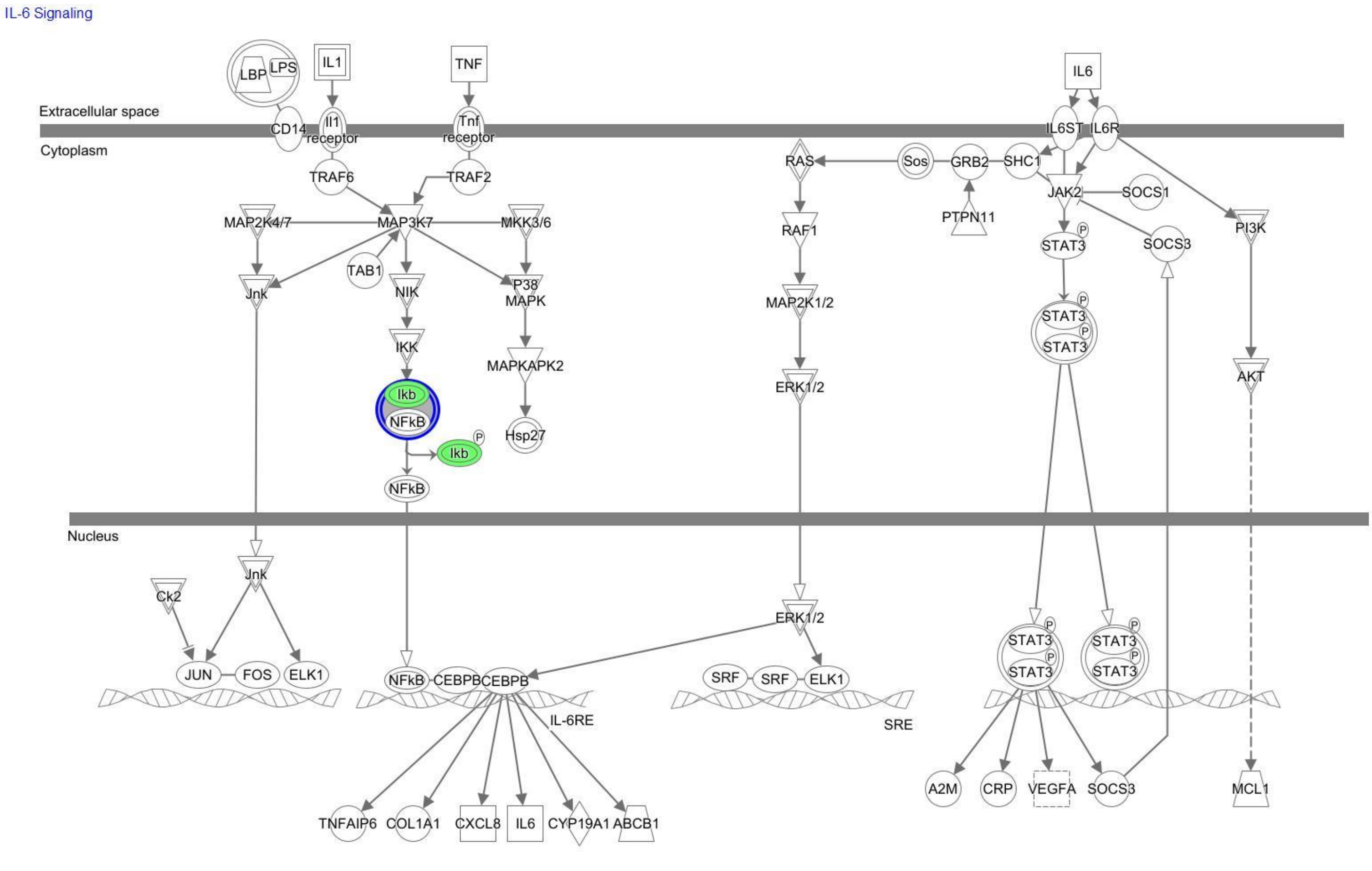

© 2000-2024 QIAGEN. All rights reserved.

Figure S41

IL-6 Signaling

6 hours
CAGE analysis

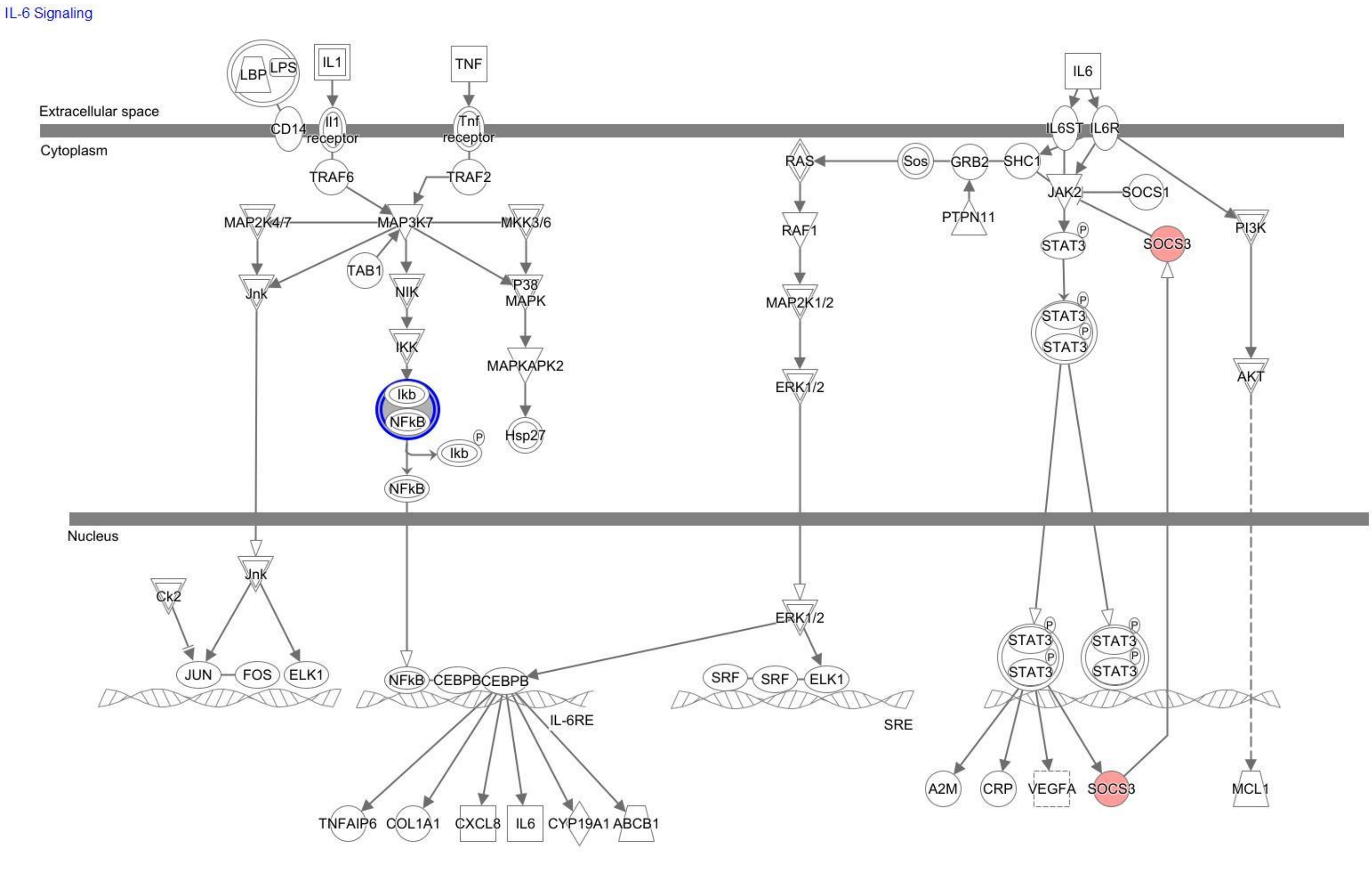

© 2000-2024 QIAGEN. All rights reserved.

Figure S42

IL-6 Signaling

8 days
CAGE analysis

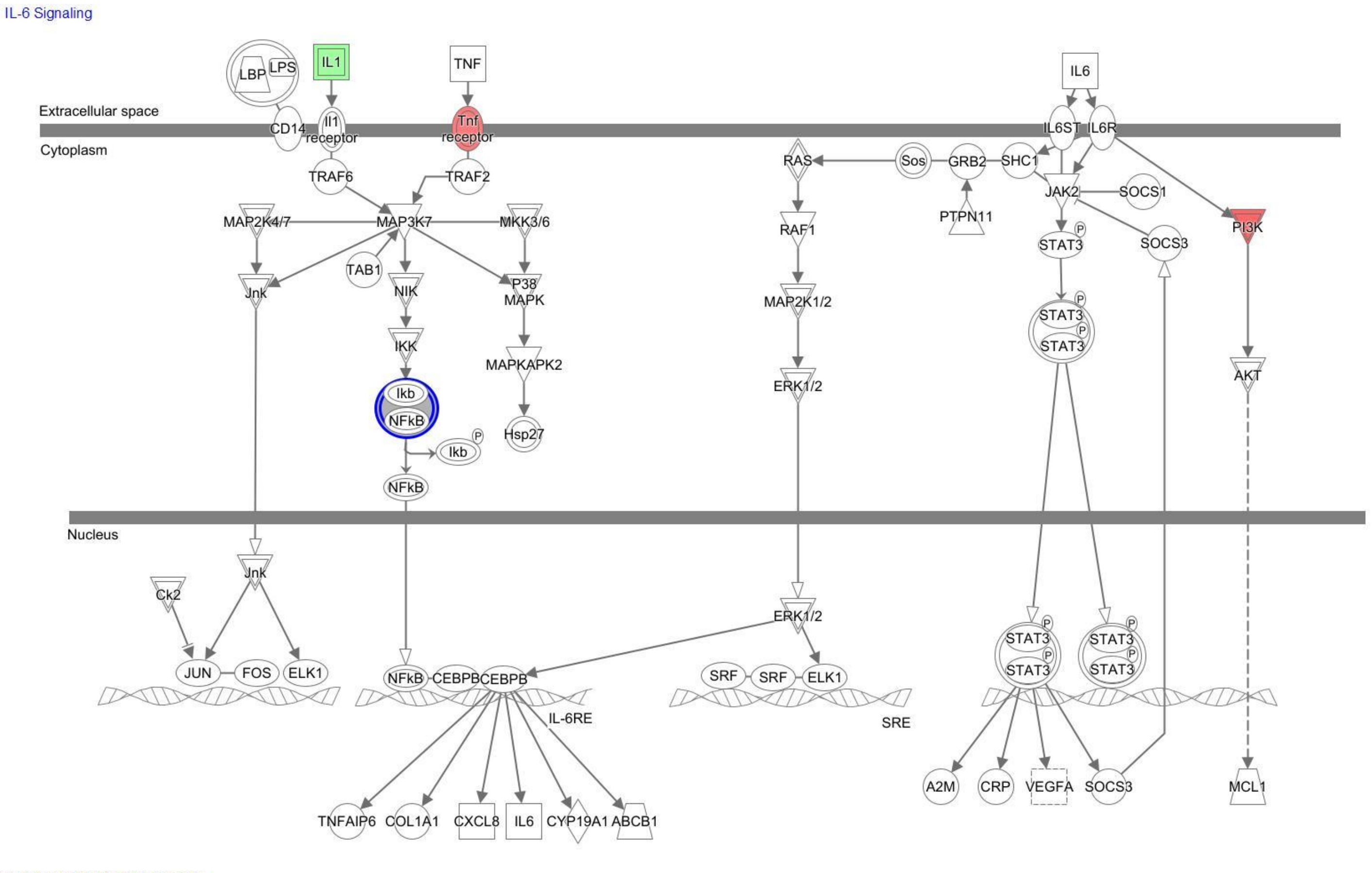

© 2000-2024 QIAGEN. All rights reserved.

Figure S43

IL-6 Signaling

Day 21
CAGE analysis

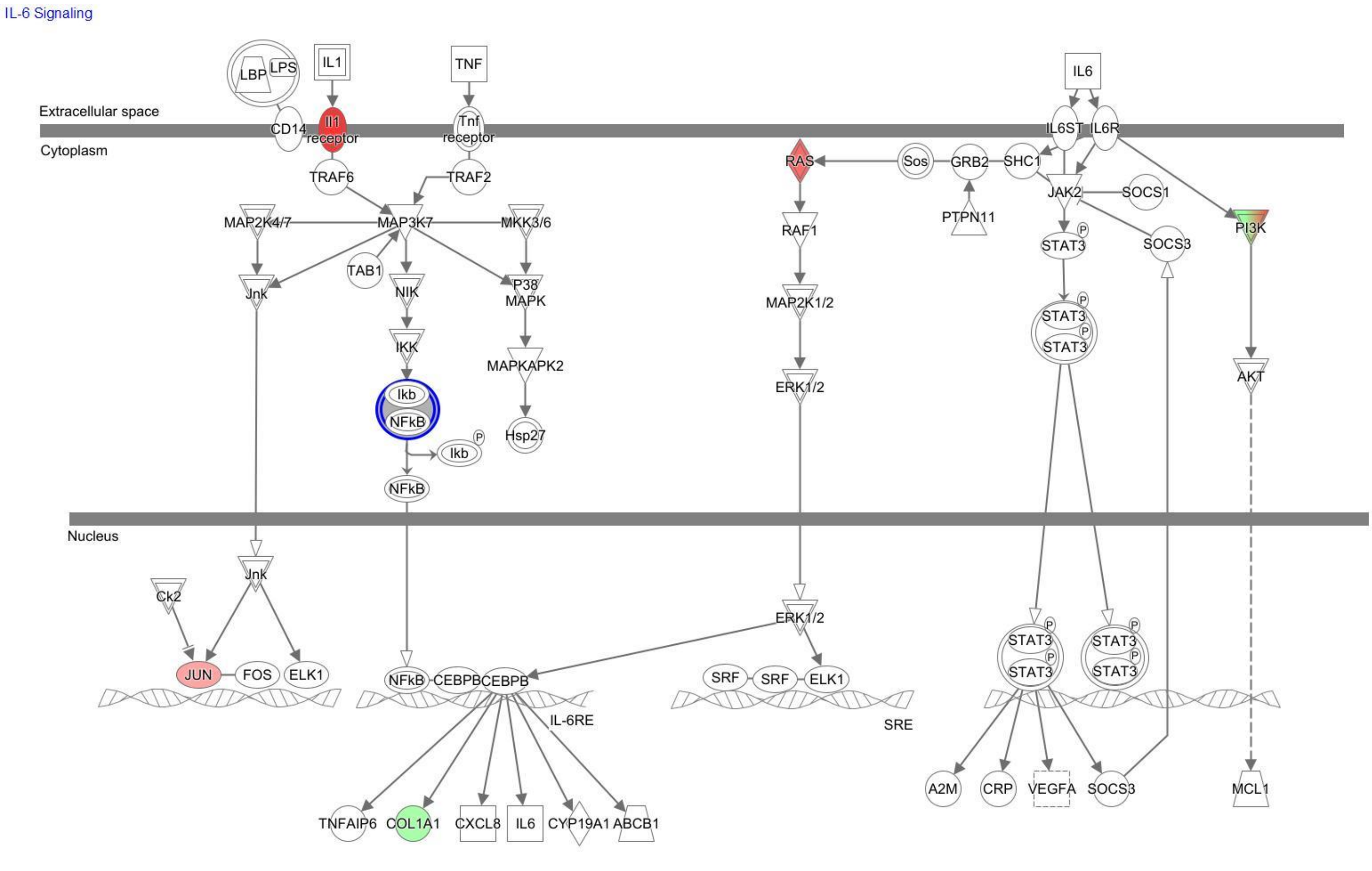

© 2000-2024 QIAGEN. All rights reserved.

Figure S44

Role of JAK family kinases in IL-6-type Cytokine Signaling

1 hour
CAGE analysis

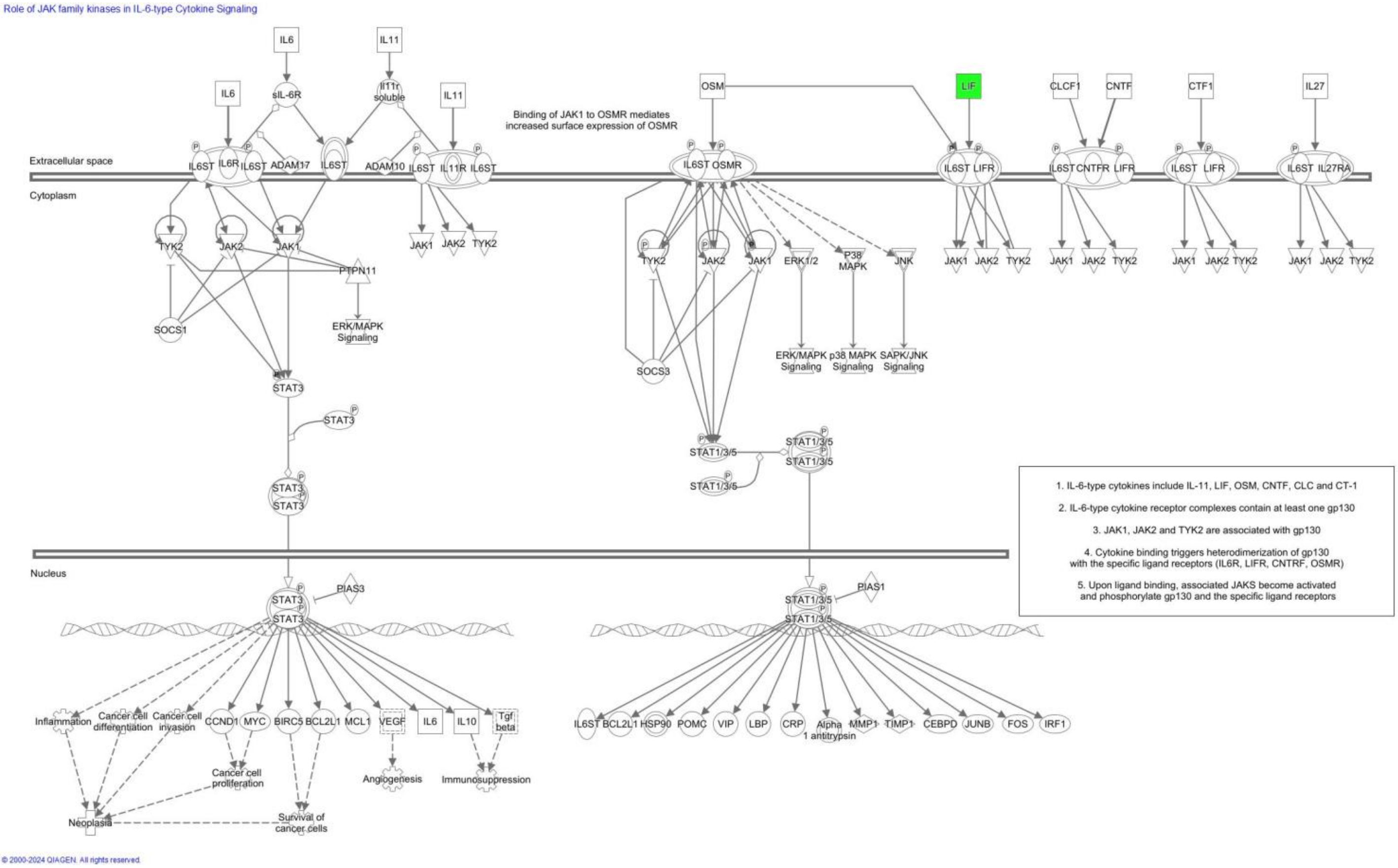

Figure S45

Role of JAK family kinases in IL-6-type Cytokine Signaling

6 hours
CAGE analysis

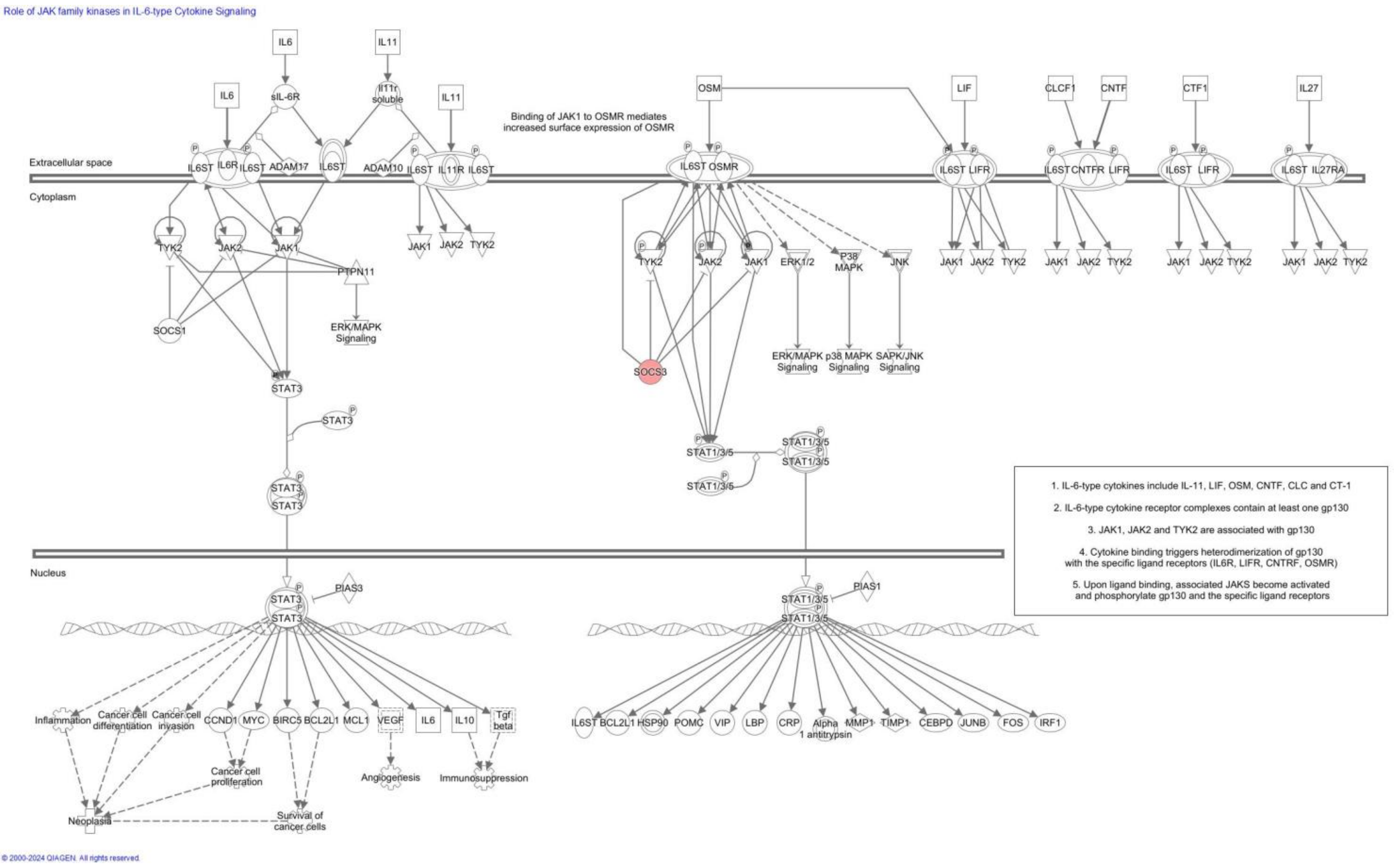

Figure S46

Role of JAK family kinases in IL-6-type Cytokine Signaling

24 hours
CAGE analysis

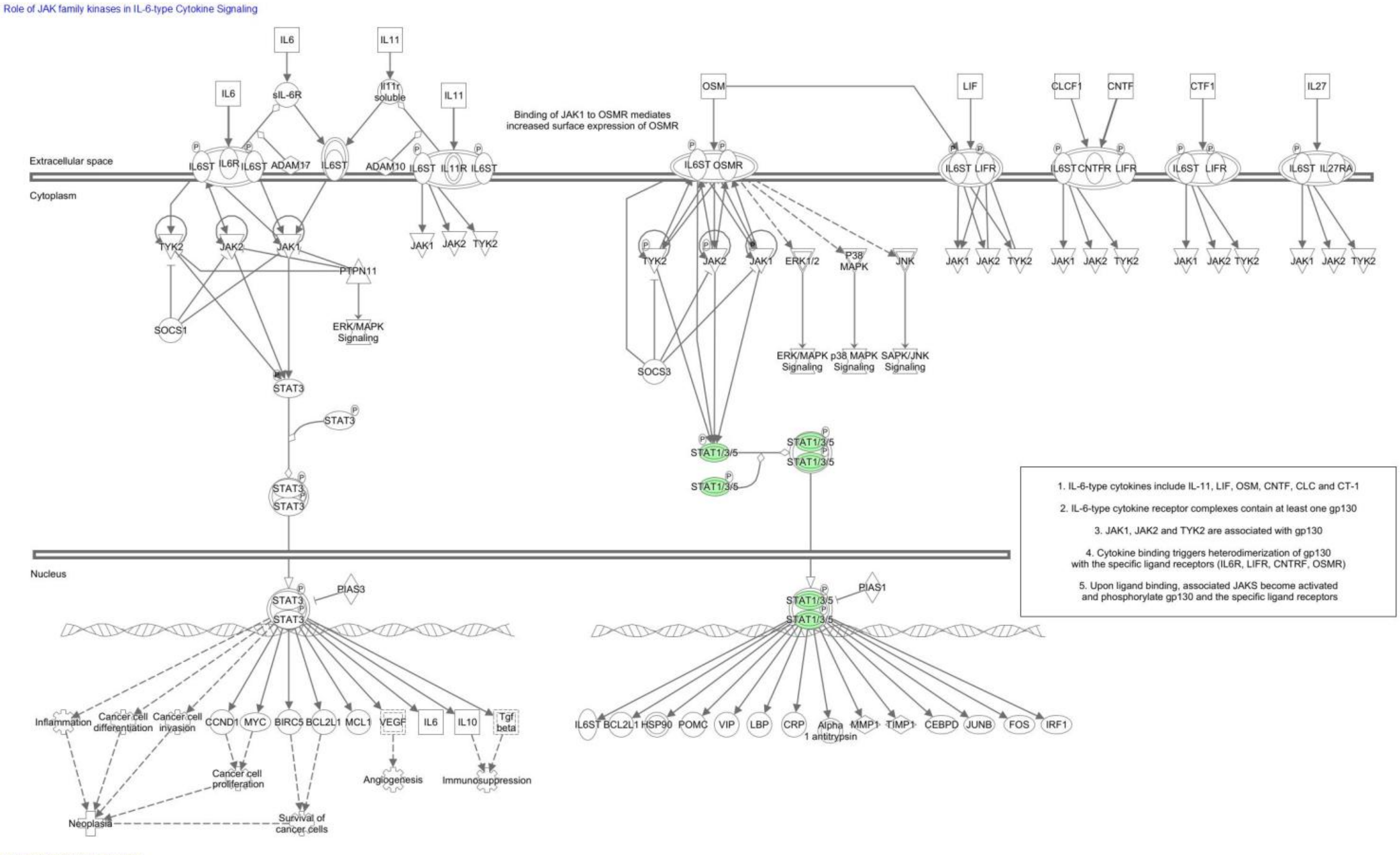

© 2000-2024 QIAGEN. All rights reserved.

Figure S47

Role of JAK family kinases in IL-6-type Cytokine Signaling

8 days
CAGE analysis

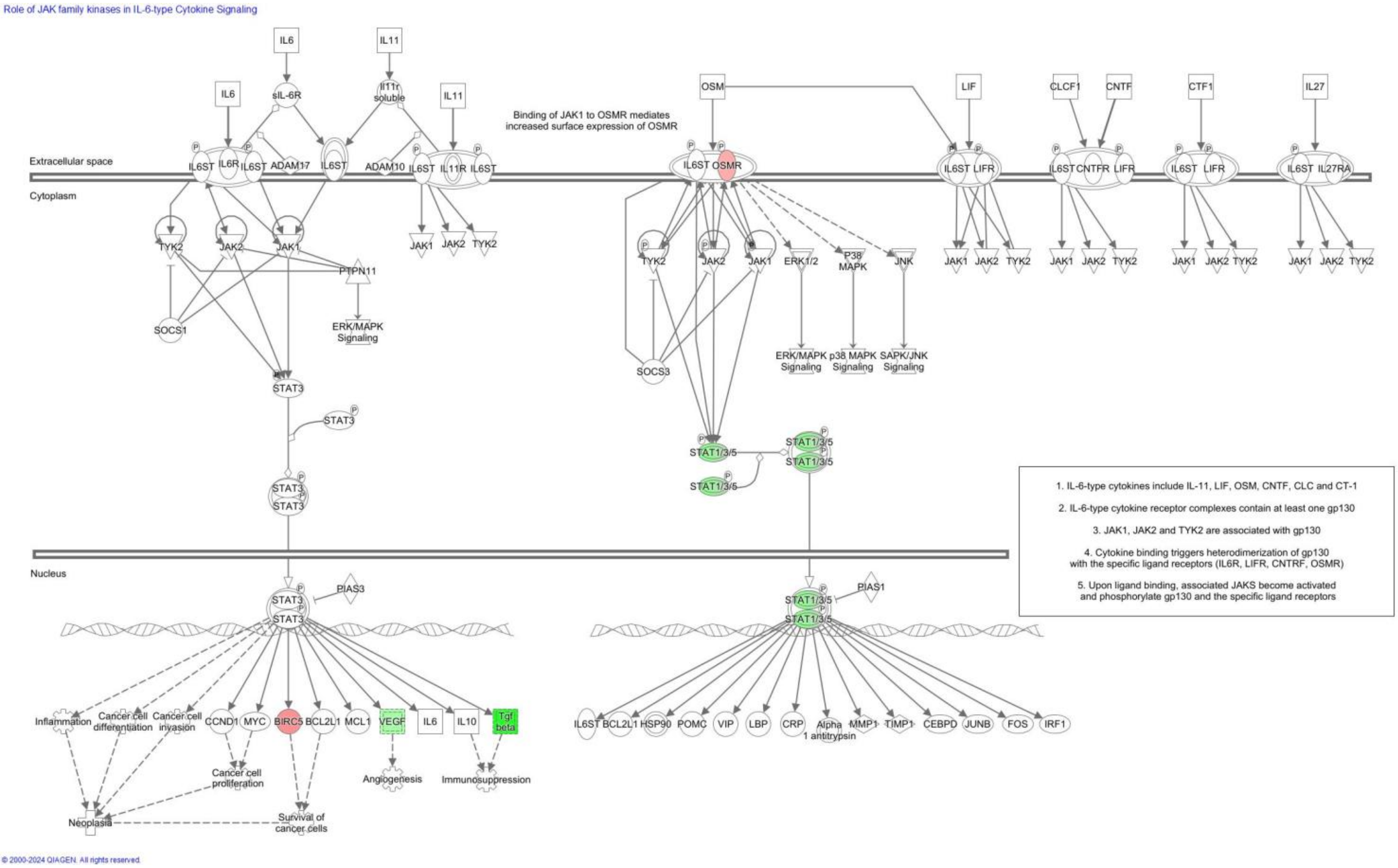

Figure S48

Role of JAK family kinases in IL-6-type Cytokine Signaling

Day 21
CAGE analysis

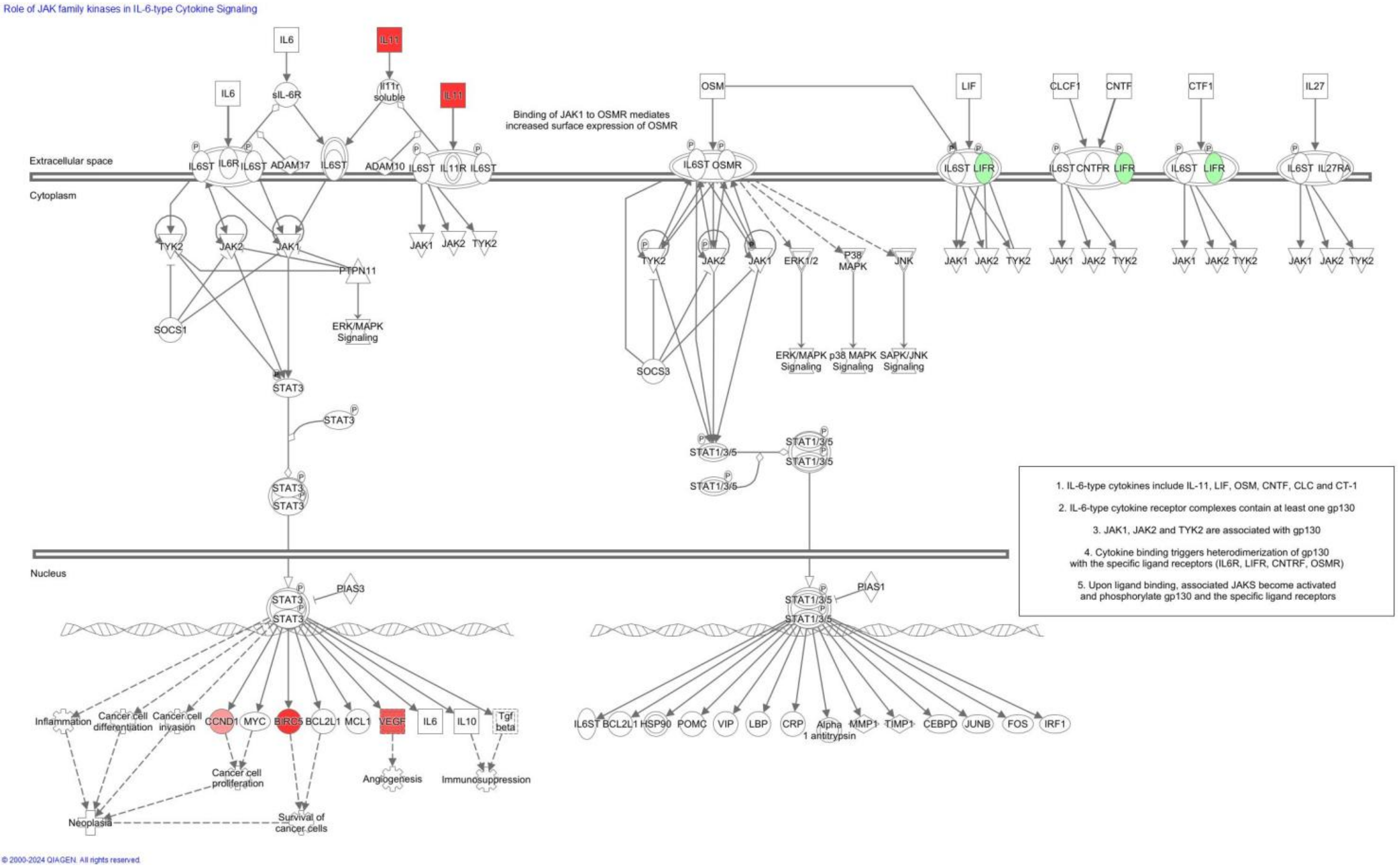

Figure S49

IL-8

1 hour
CAGE analysis

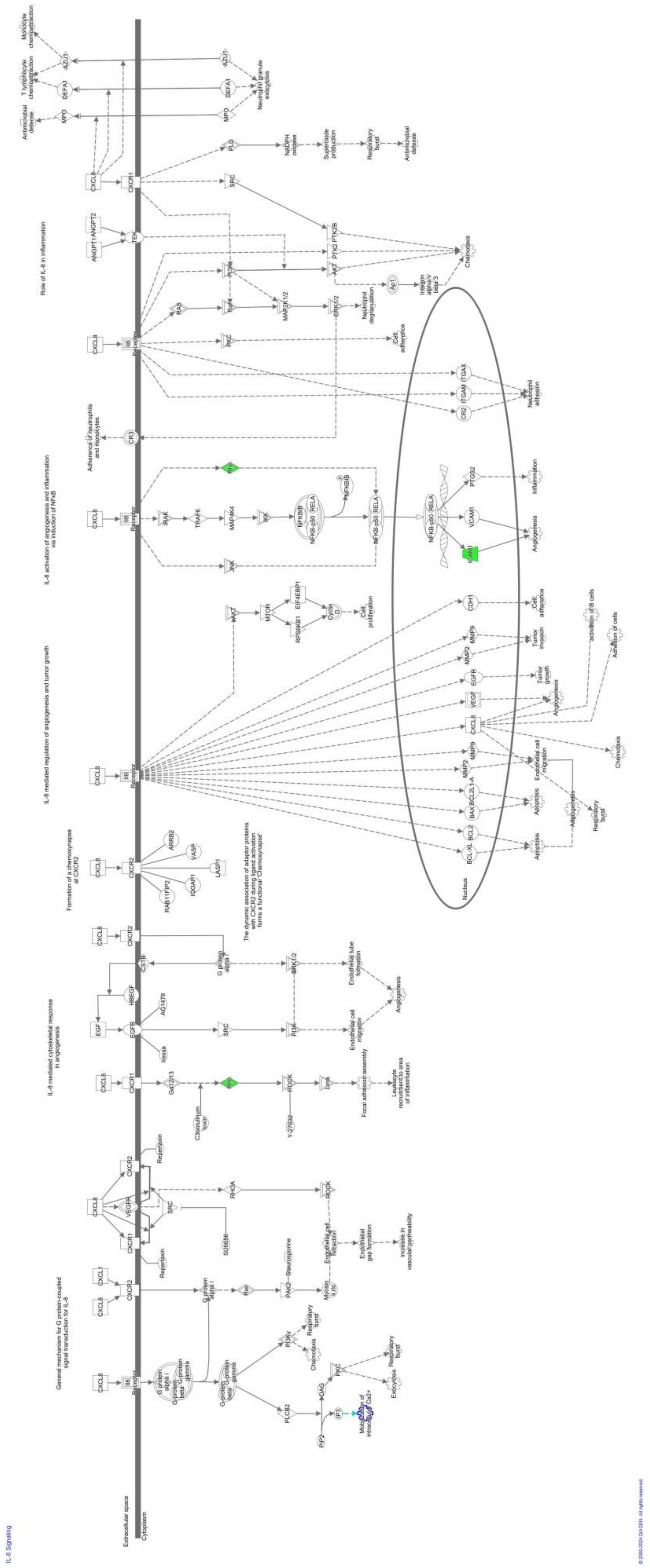

Figure S50

IL-8

8 days
CAGE analysis

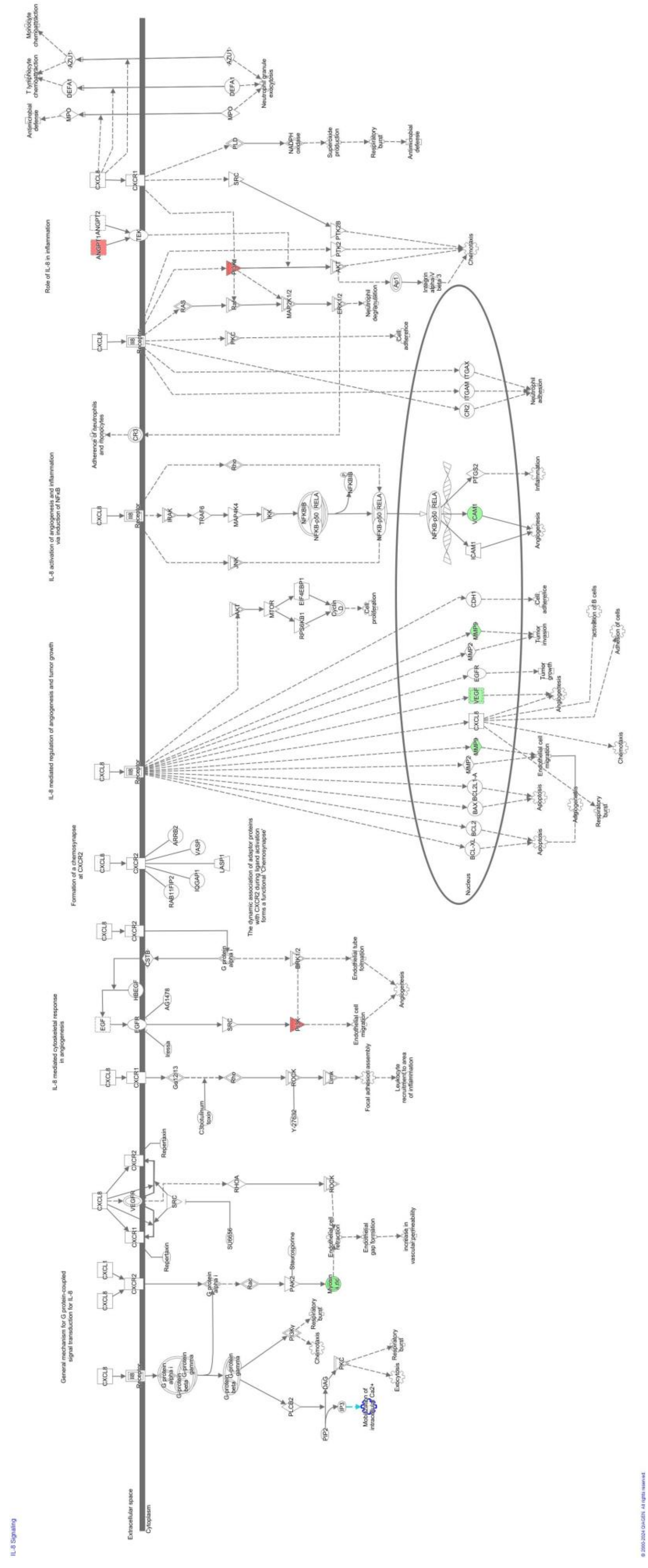

Figure S51

IL-8

Day 21
CAGE analysis

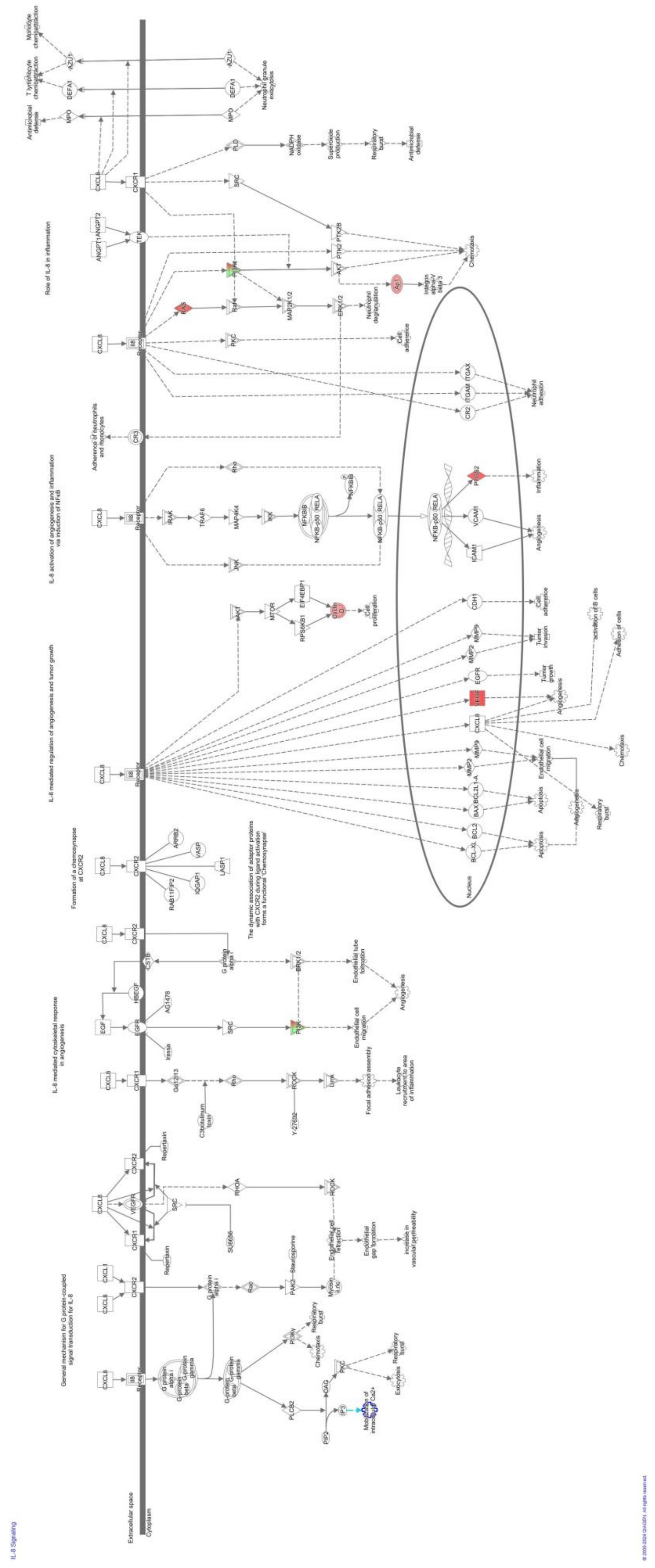

Figure S52

TNFR2 Signaling

1 hour
CAGE analysis

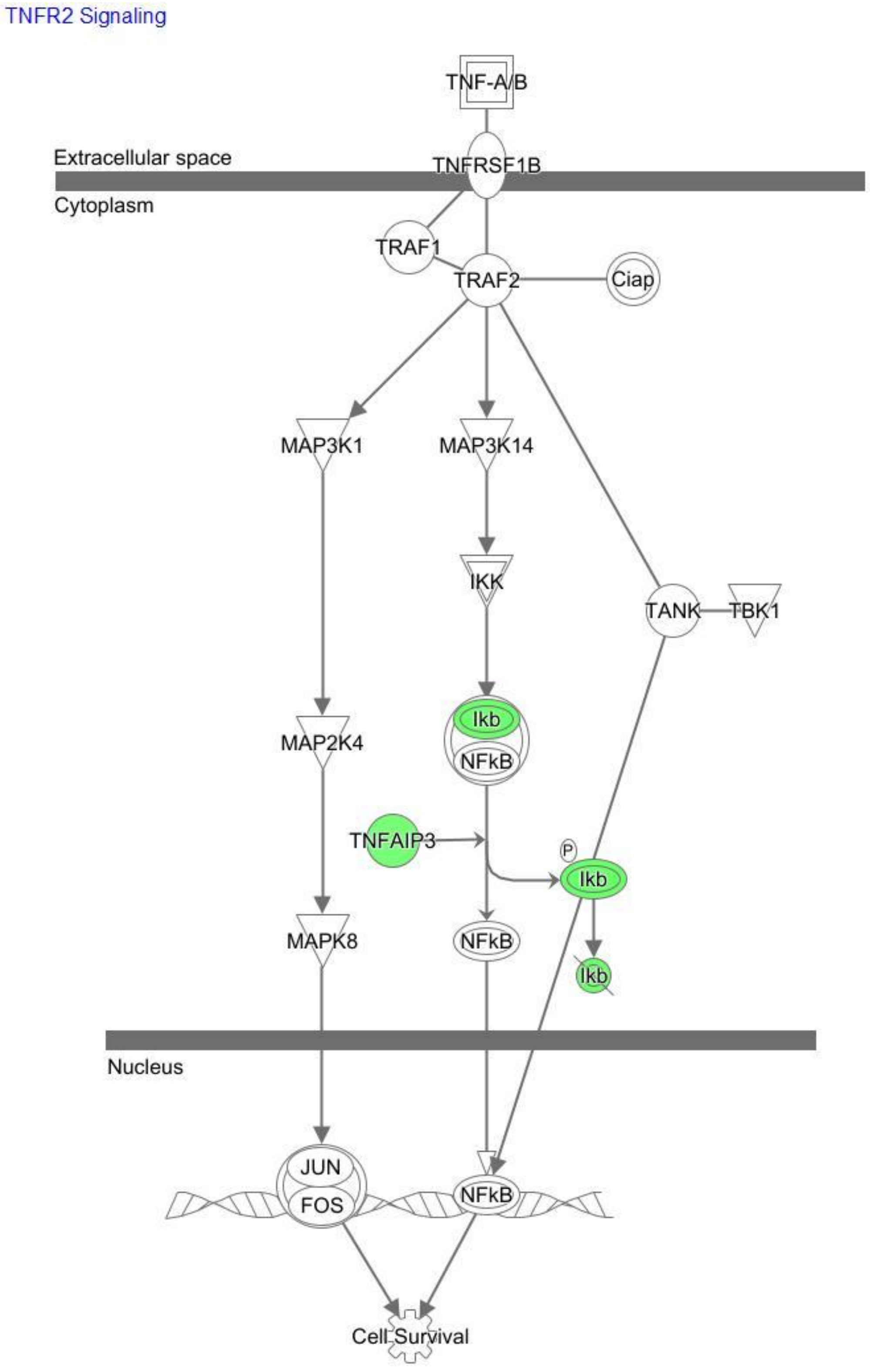

© 2000-2024 QIAGEN. All rights reserved.

Figure S53

TNFR2 Signaling

8 days
CAGE analysis

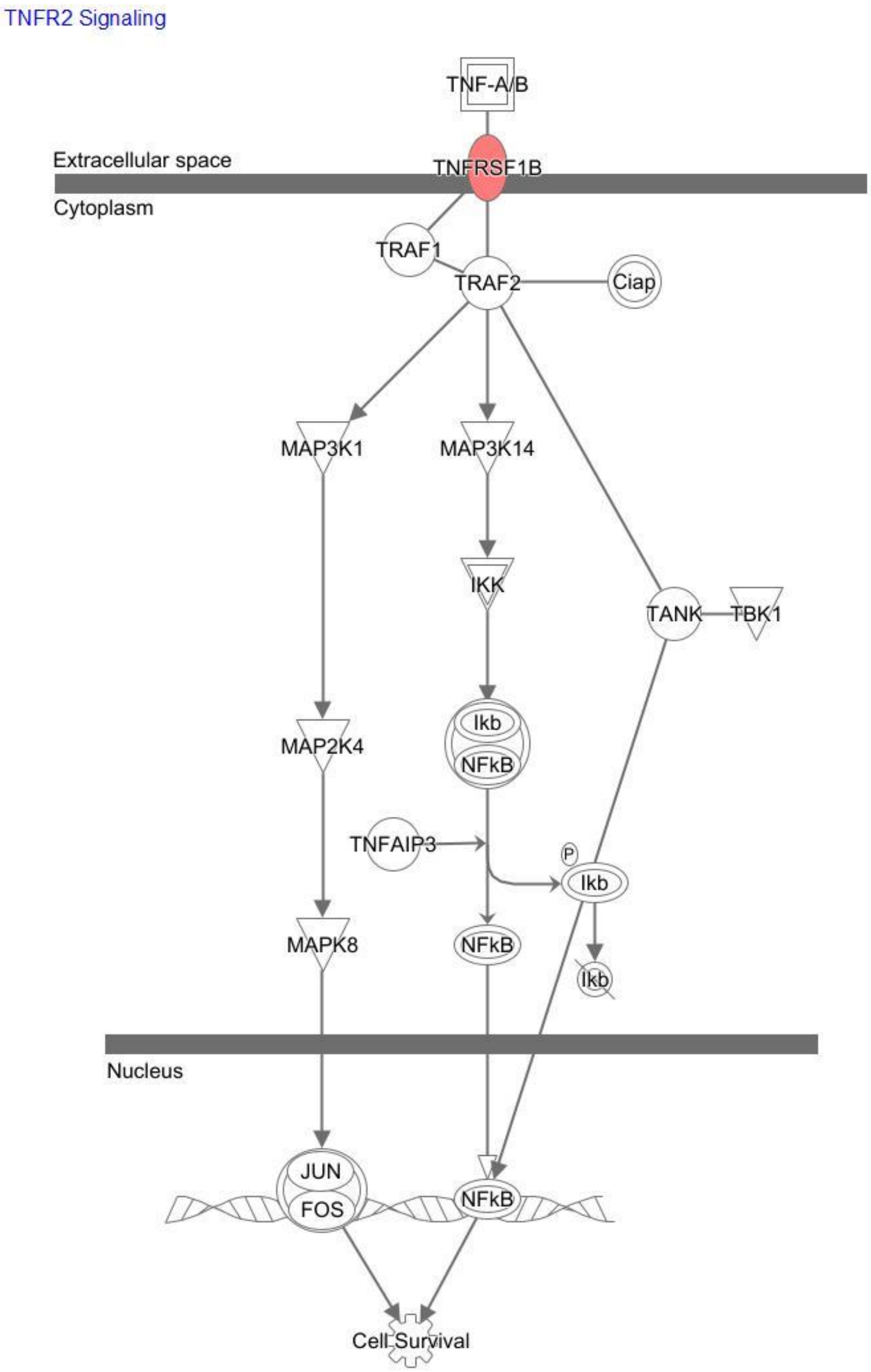

© 2000-2024 QIAGEN. All rights reserved.

Figure S54

TNFR2 Signaling

Day 21
CAGE analysis

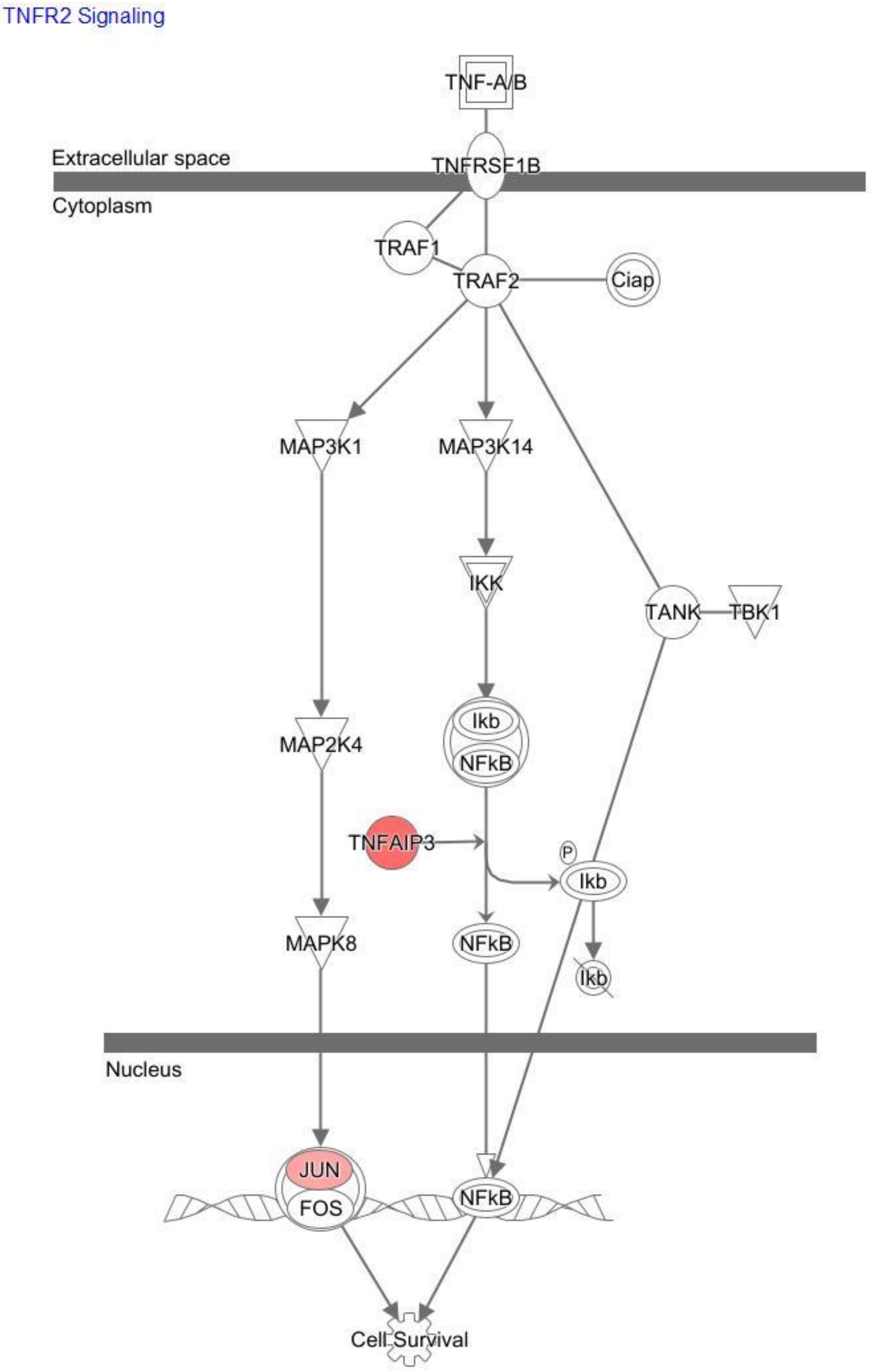

© 2000-2024 QIAGEN. All rights reserved.

Figure S55

Tumor Microenvironment

1 hour
CAGE analysis

Tumor Microenvironment Pathway

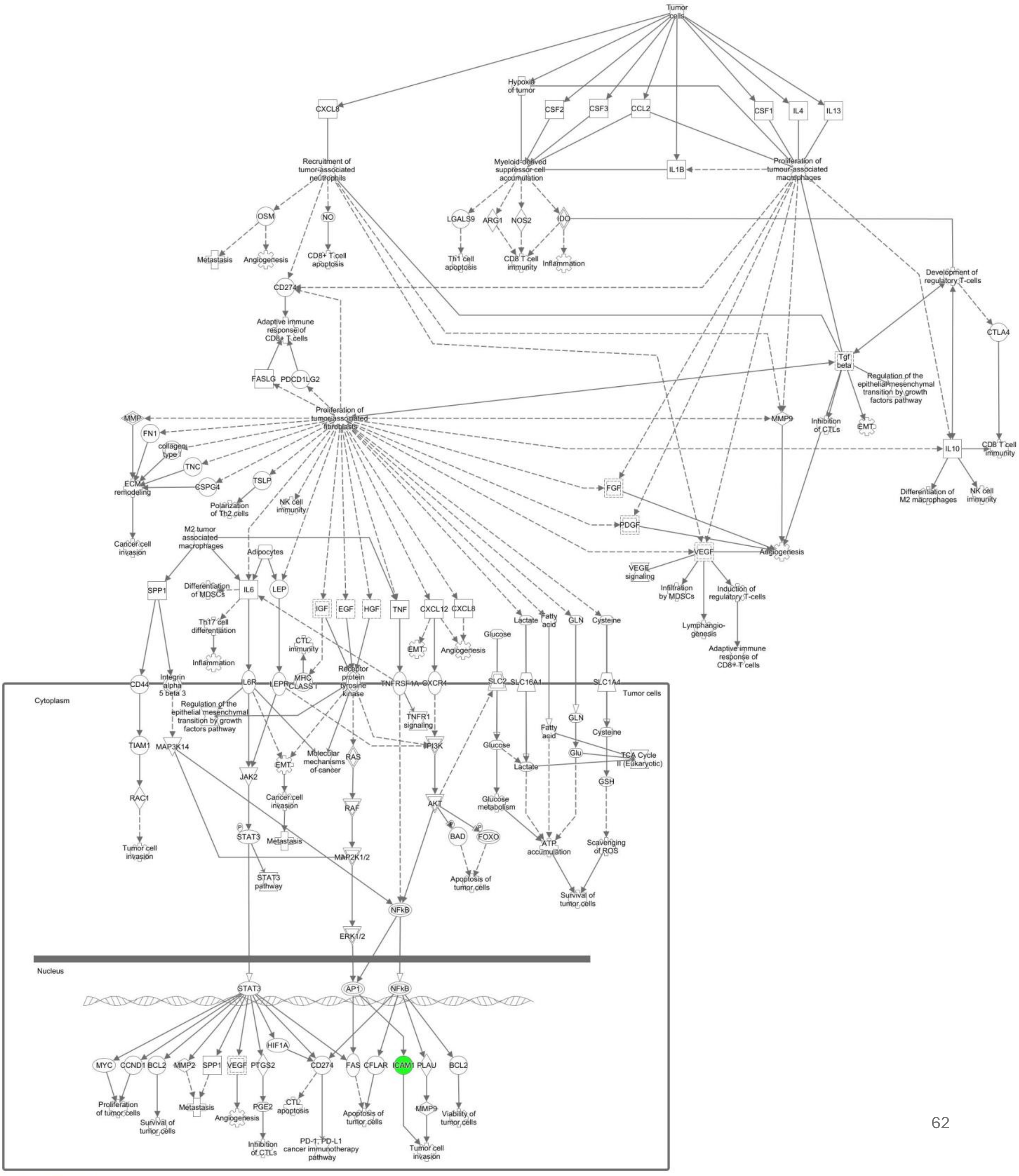

© 2000-2024 QIAGEN. All rights reserved.

Figure S56

Tumor Microenvironment

24 hours
CAGE analysis

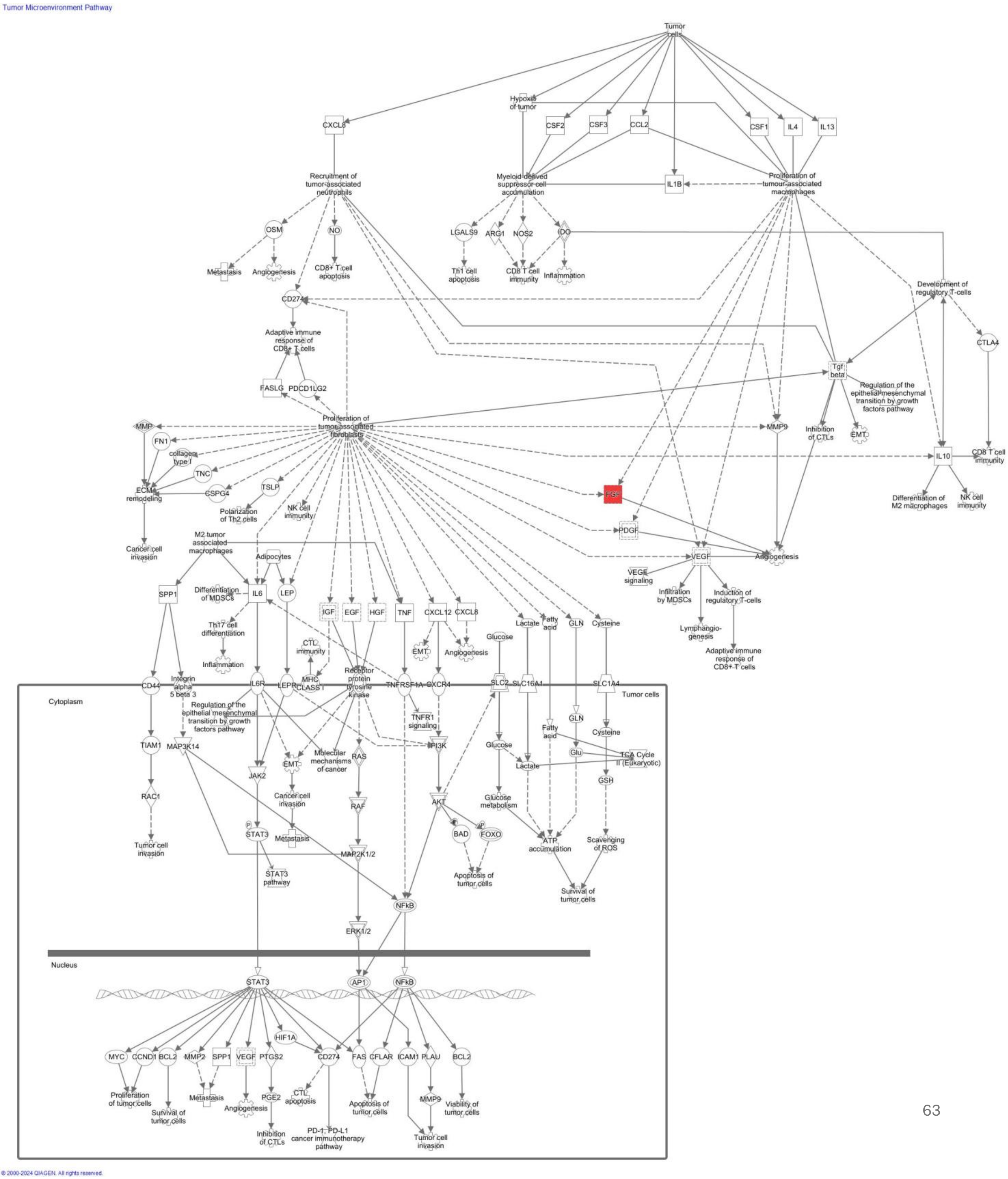

Figure S57

Tumor Microenvironment

8 days
CAGE analysis

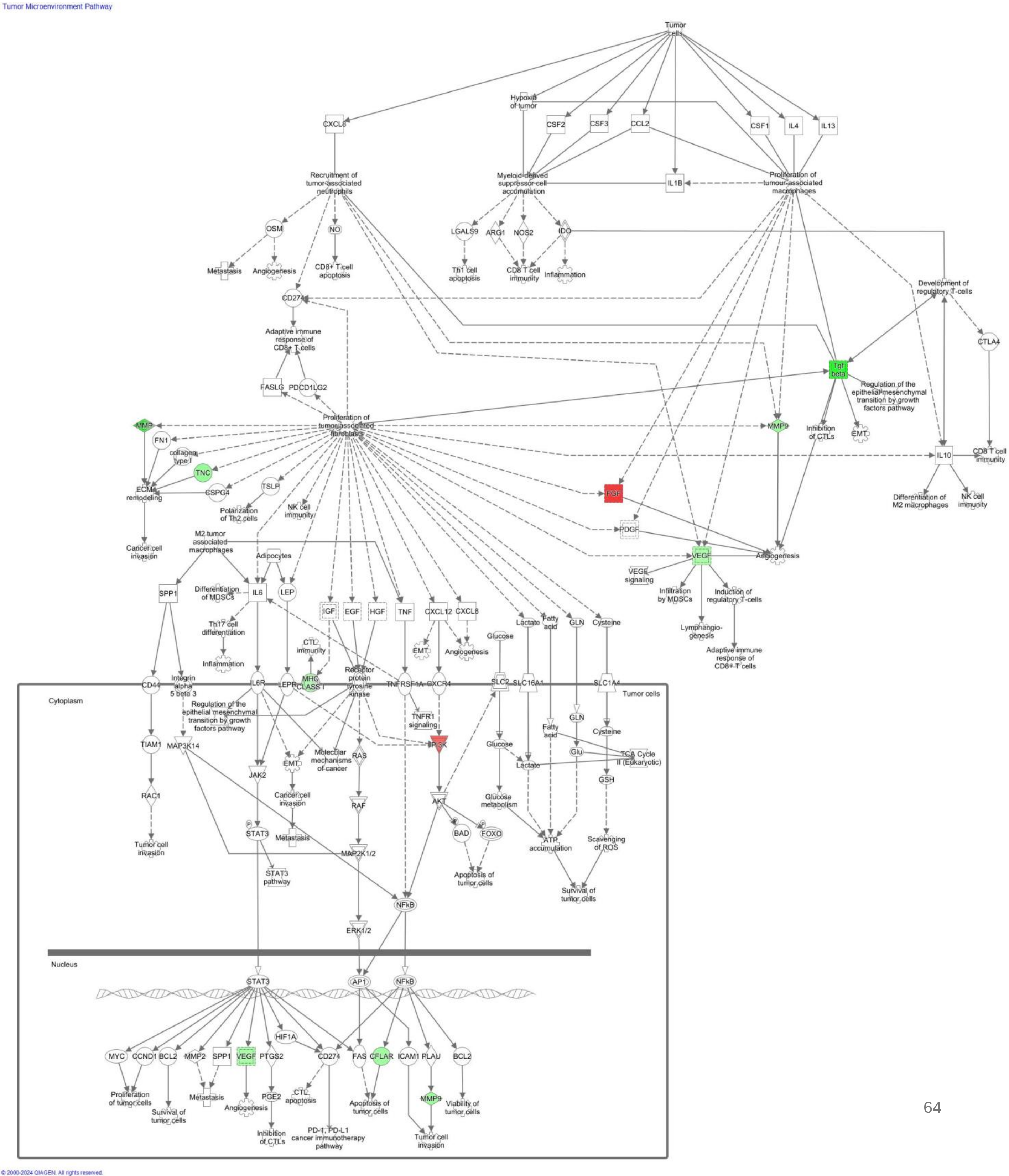

© 2000-2024 QIAGEN. All rights reserved.

Figure S58

Tumor Microenvironment

Day 21
CAGE analysis

Tumor Microenvironment Pathway

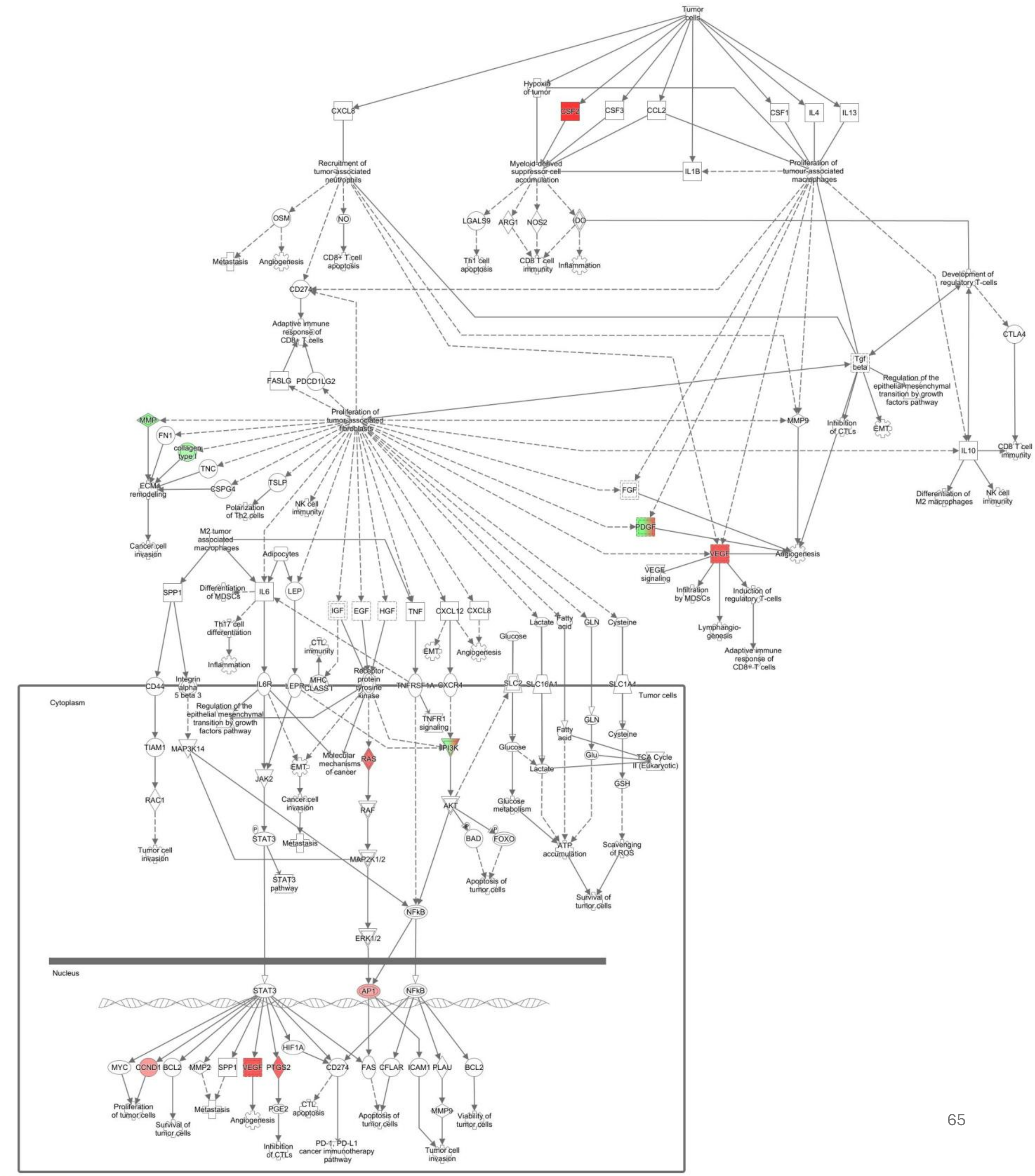

© 2000-2024 QIAGEN. All rights reserved.

Figure S59

JAK/STAT Signaling

6 hours
CAGE analysis

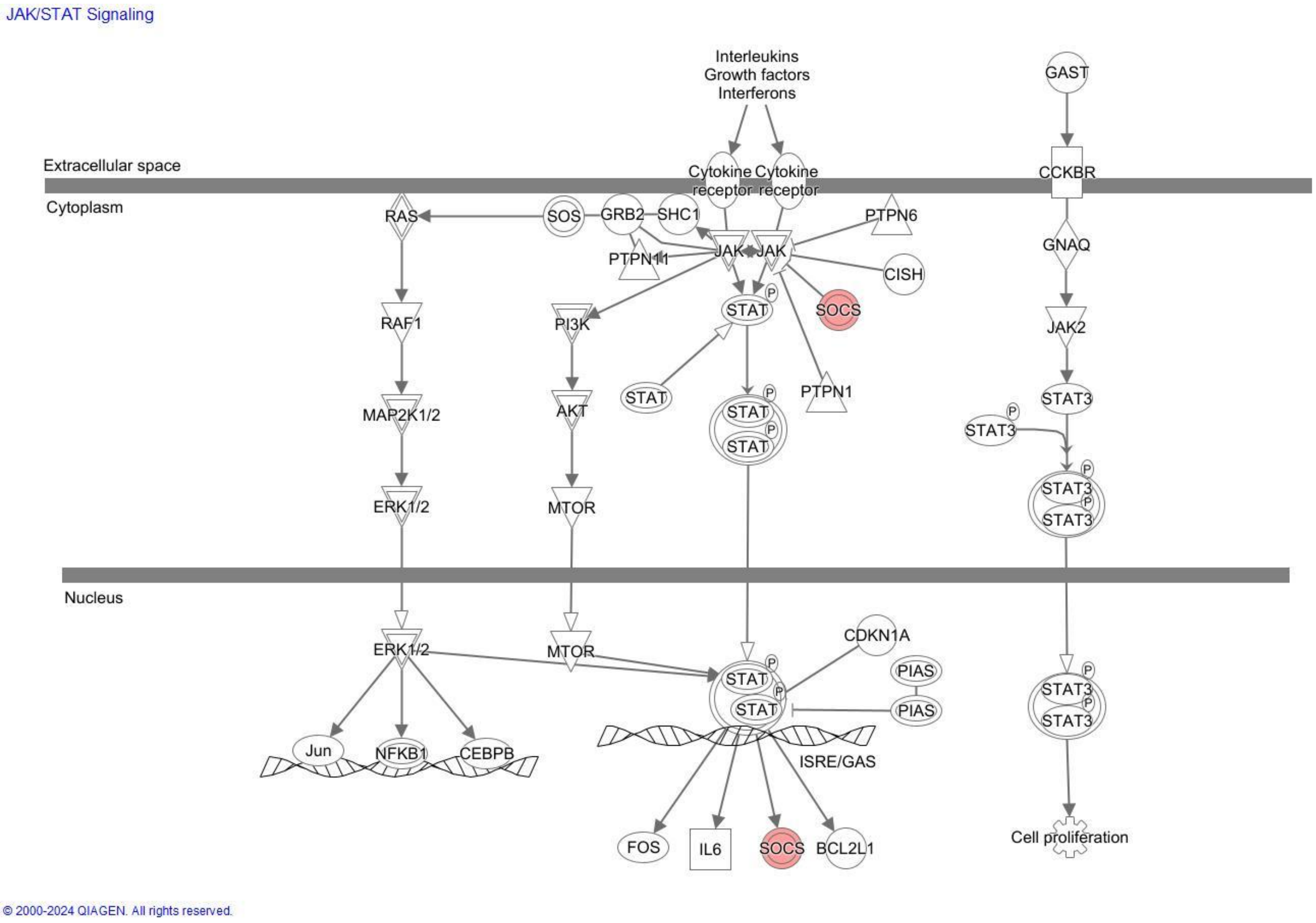

© 2000-2024 QIAGEN. All rights reserved.

Figure S60

JAK/STAT Signaling

24 hours
CAGE analysis

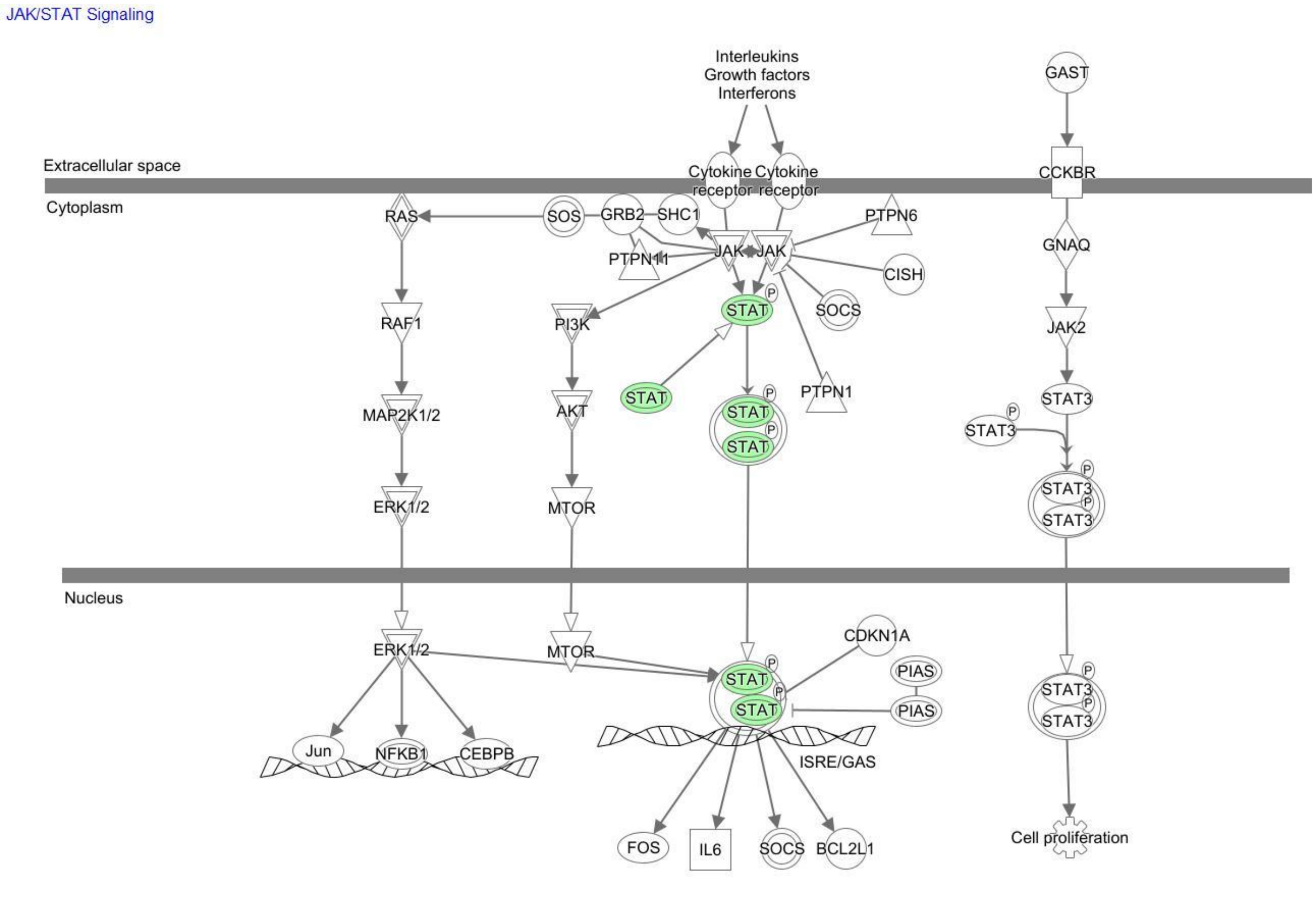

© 2000-2024 QIAGEN. All rights reserved.

Figure S61

JAK/STAT Signaling

8 days
CAGE analysis

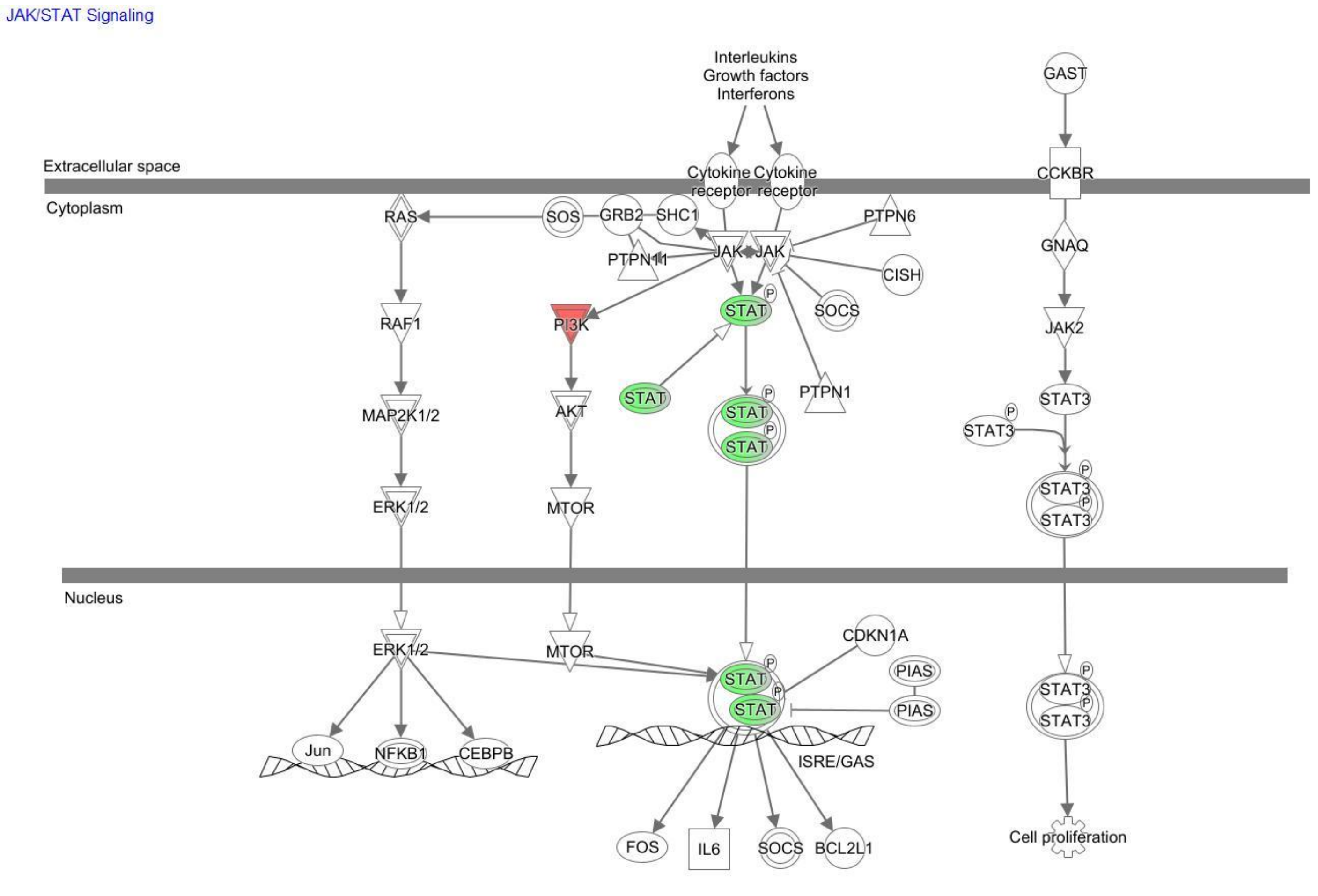

© 2000-2024 QIAGEN. All rights reserved.

Figure S62

JAK/STAT Signaling

day 21
CAGE analysis

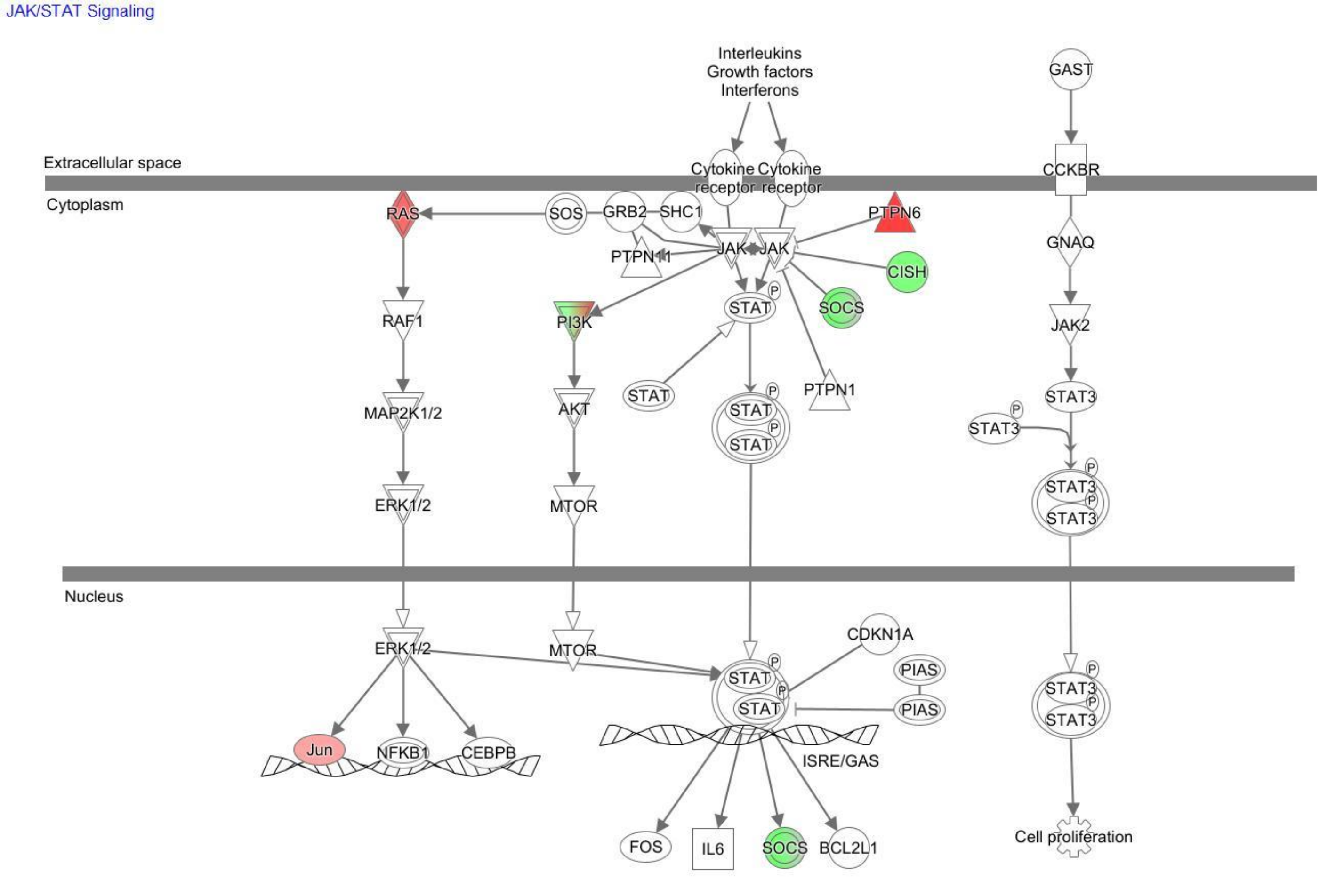

© 2000-2024 QIAGEN. All rights reserved.

Figure S63

Glioma Invasiveness Signaling

1 hour
CAGE analysis

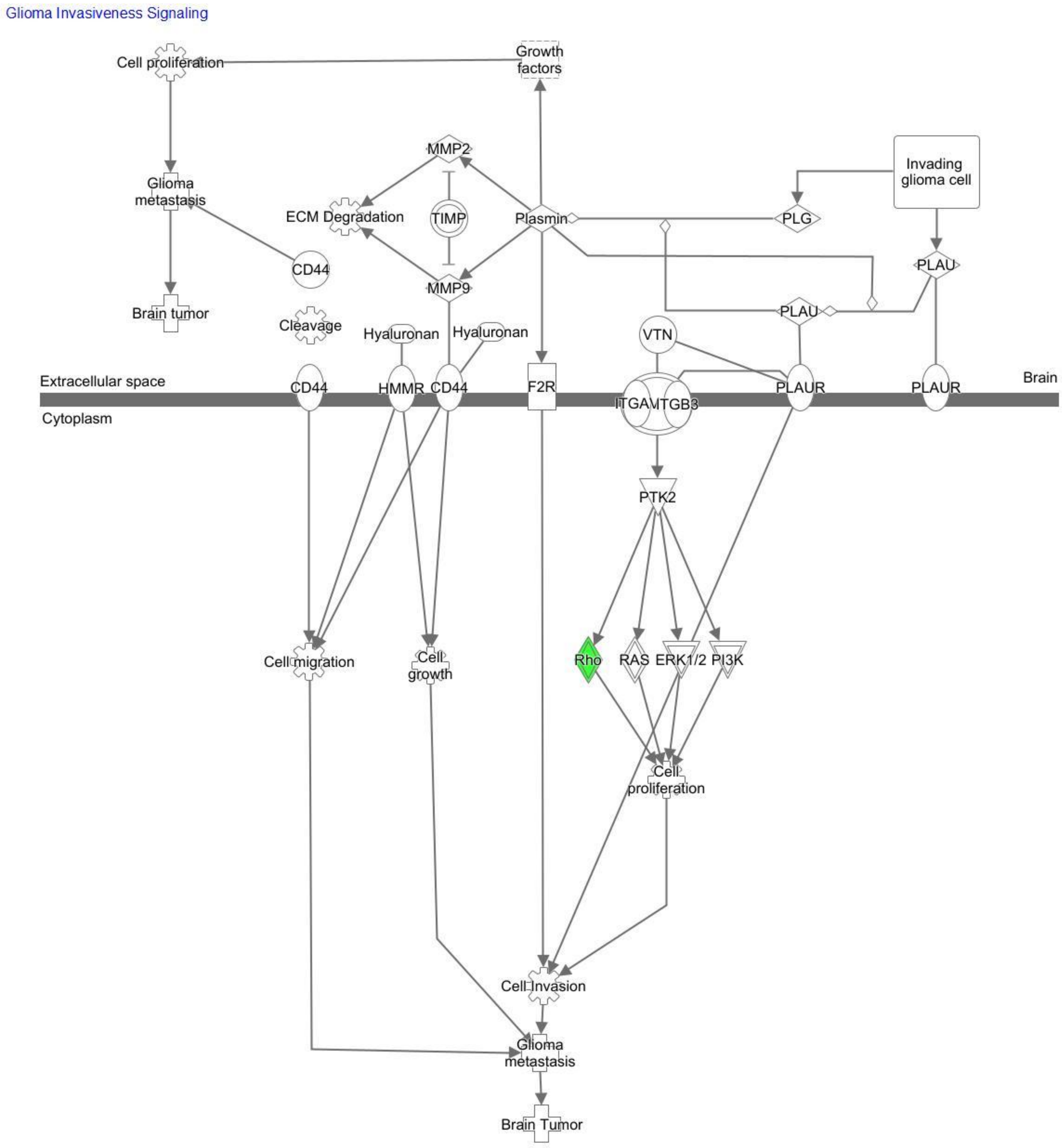

© 2000-2024 QIAGEN. All rights reserved.

Figure S64

Glioma Invasiveness Signaling

24 hours
CAGE analysis

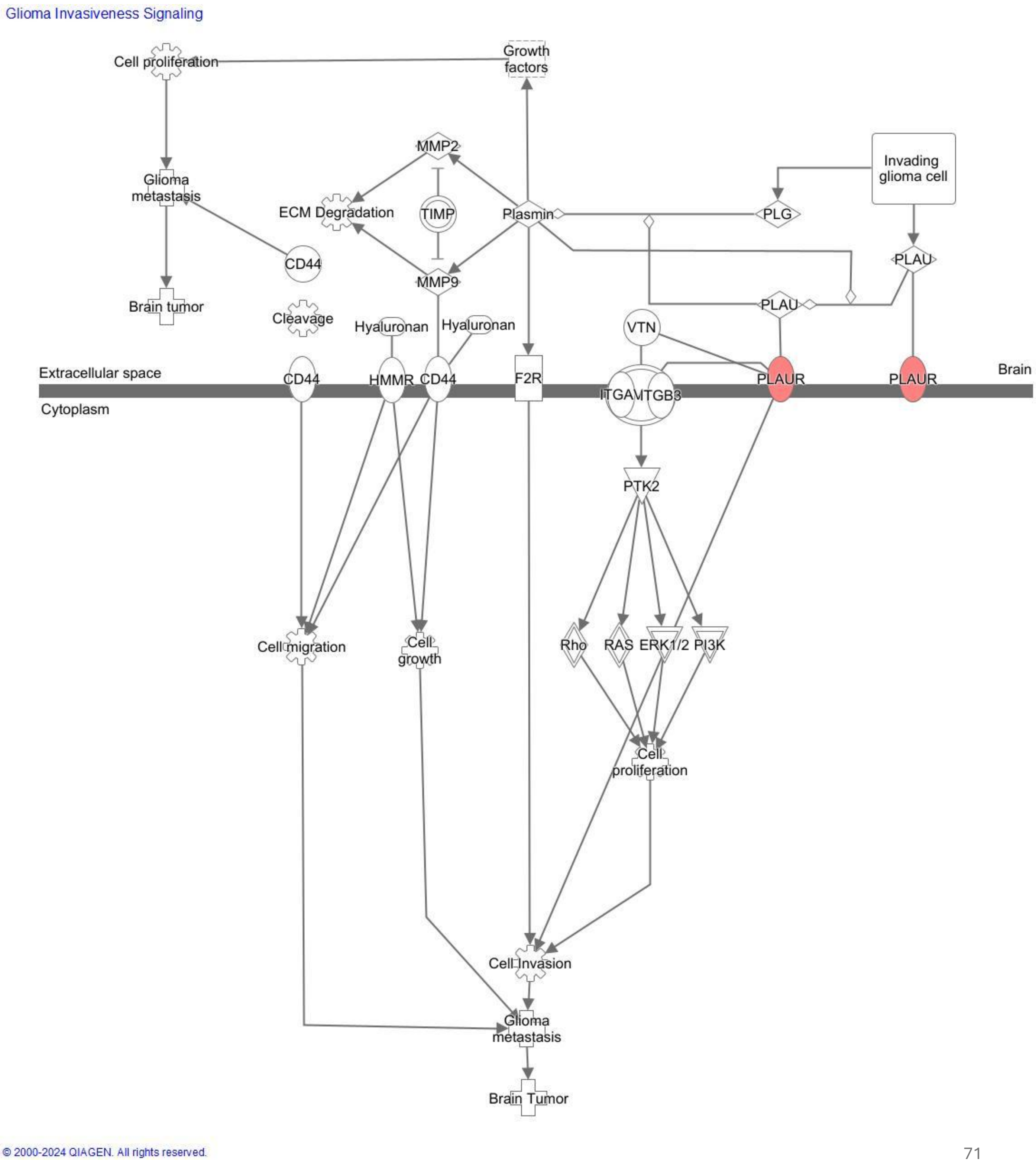

© 2000-2024 QIAGEN. All rights reserved.

Figure S65

Glioma Invasiveness Signaling

8 days
CAGE analysis

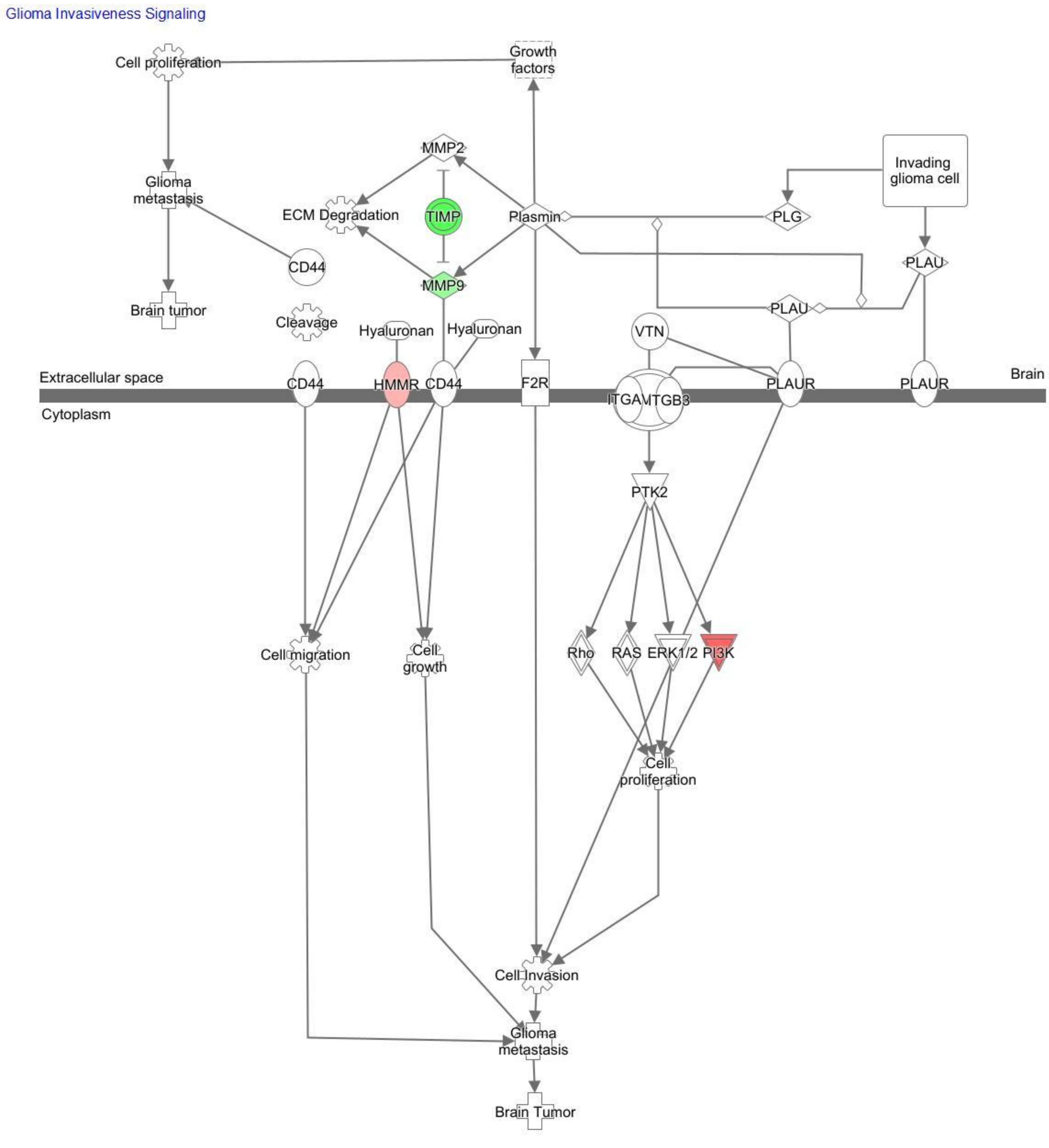

© 2000-2024 QIAGEN. All rights reserved.

Figure S66

Glioma Invasiveness Signaling

Day 21
CAGE analysis

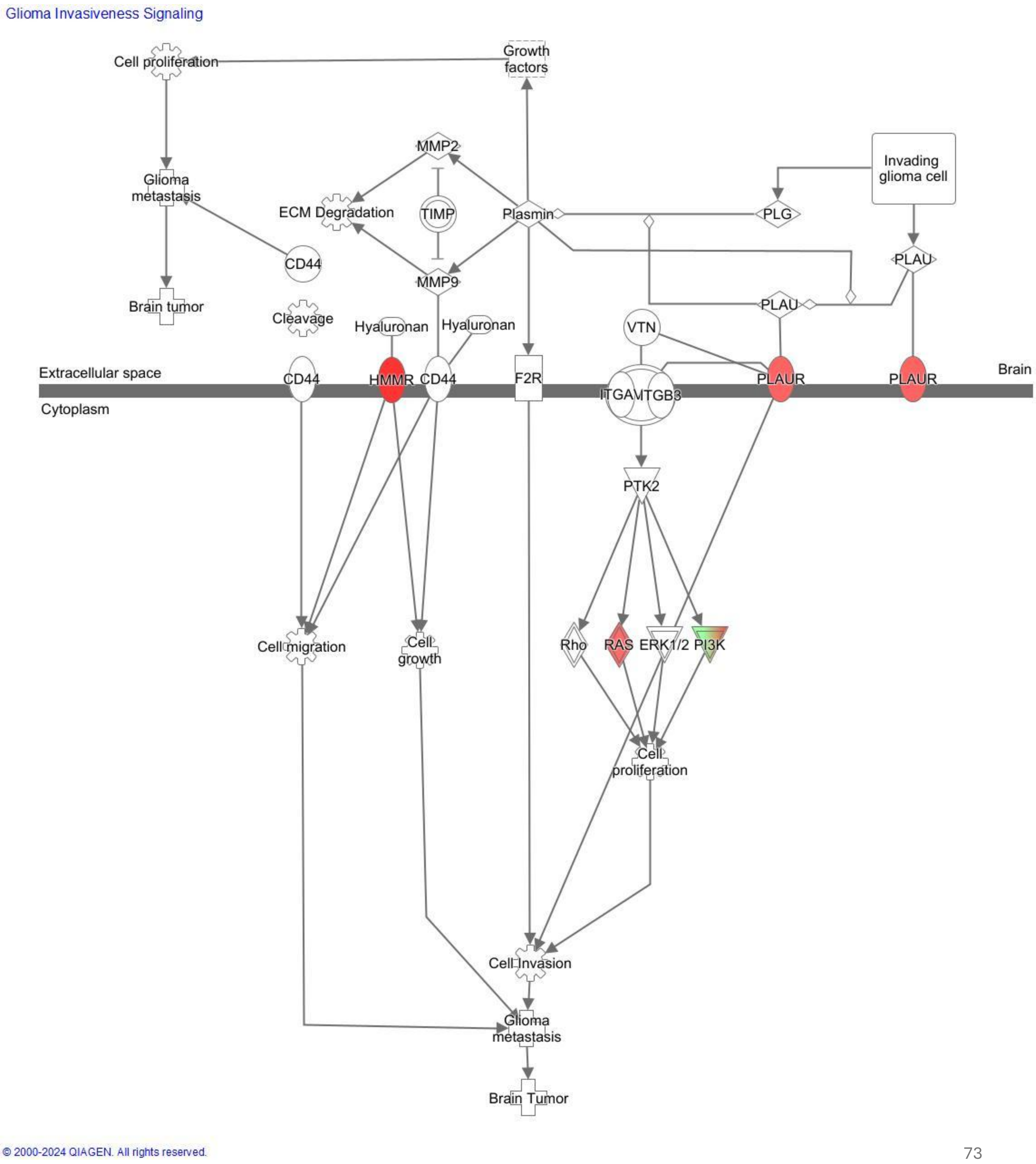

© 2000-2024 QIAGEN. All rights reserved.

Figure S67

FAK Signaling

1 hour
CAGE analysis

FAK Signaling

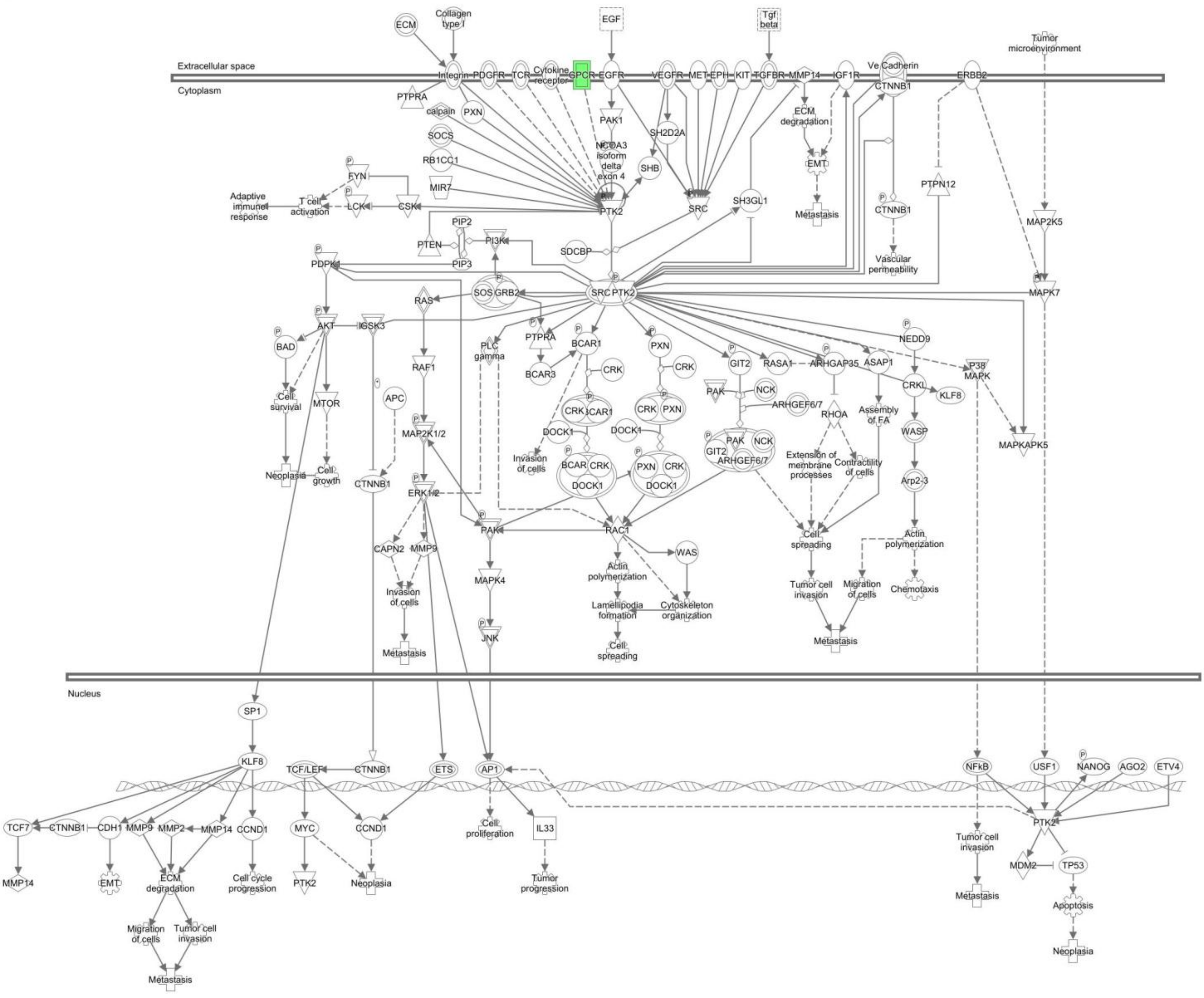

© 2000-2024 QIAGEN. All rights reserved.

Figure S68

FAK Signaling

6 hours
CAGE analysis

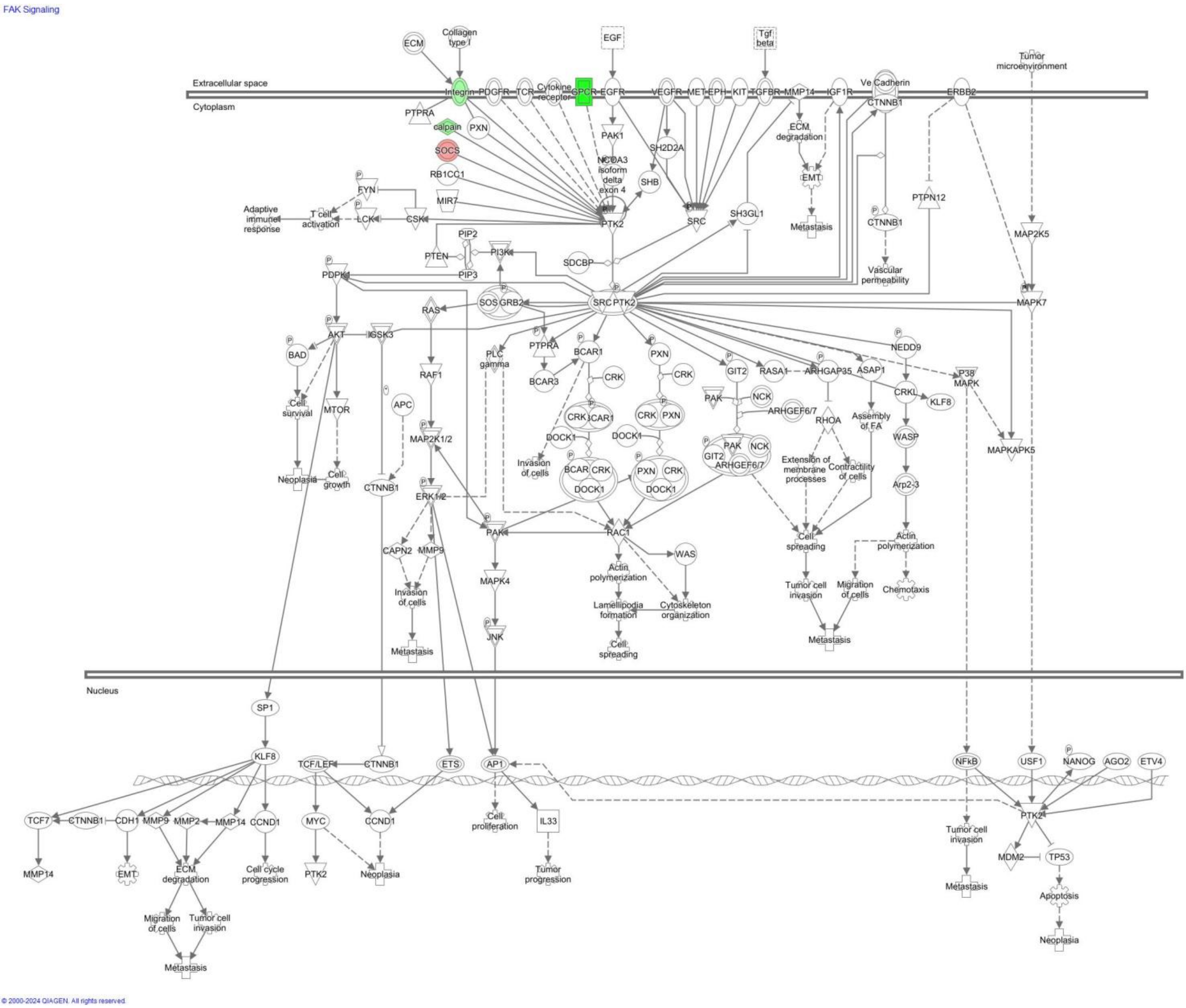

Figure S69

FAK Signaling

24 hours
CAGE analysis

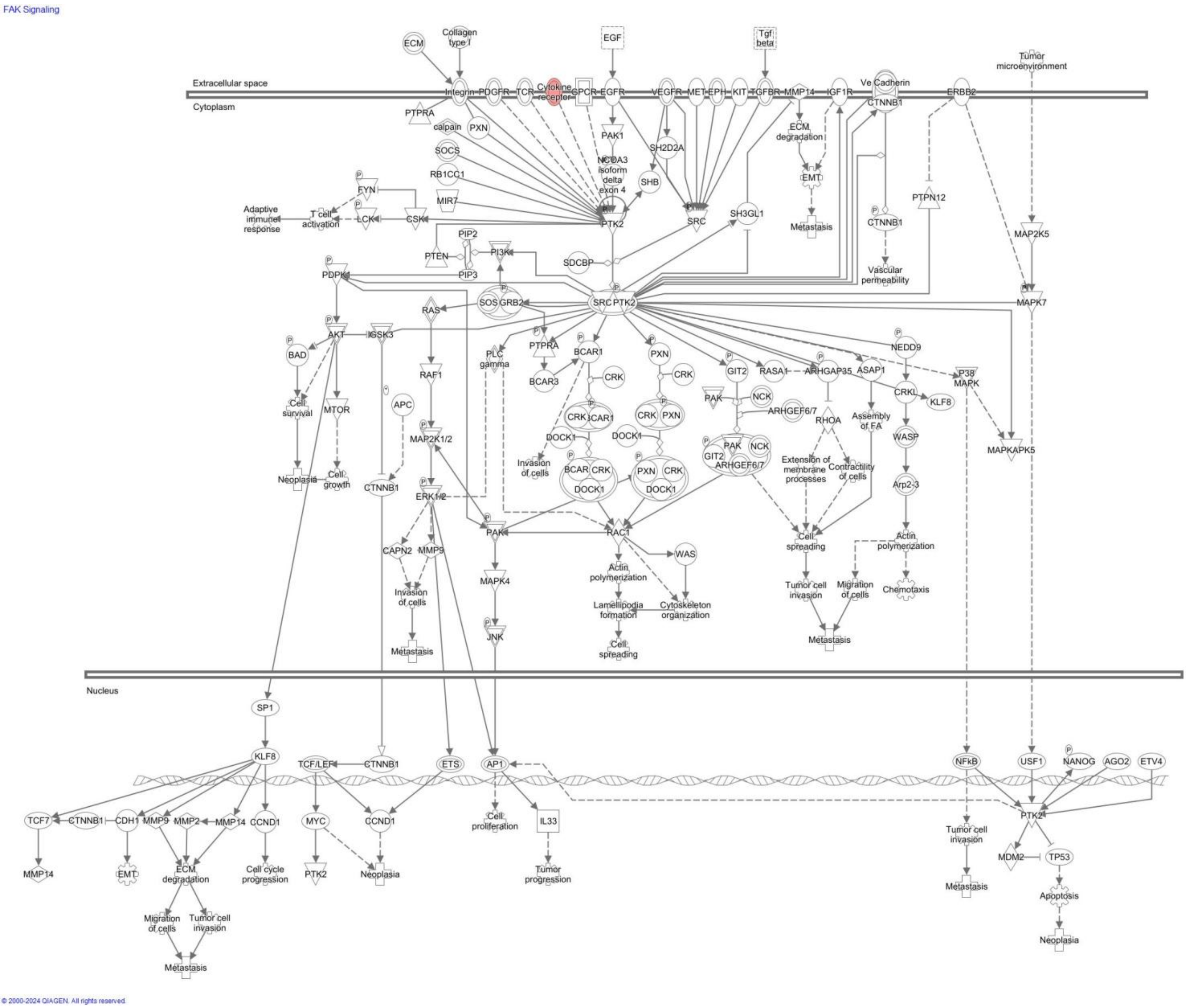

© 2000-2024 QIAGEN. All rights reserved.

Figure S70

FAK Signaling

8 days
CAGE analysis

FAK Signaling

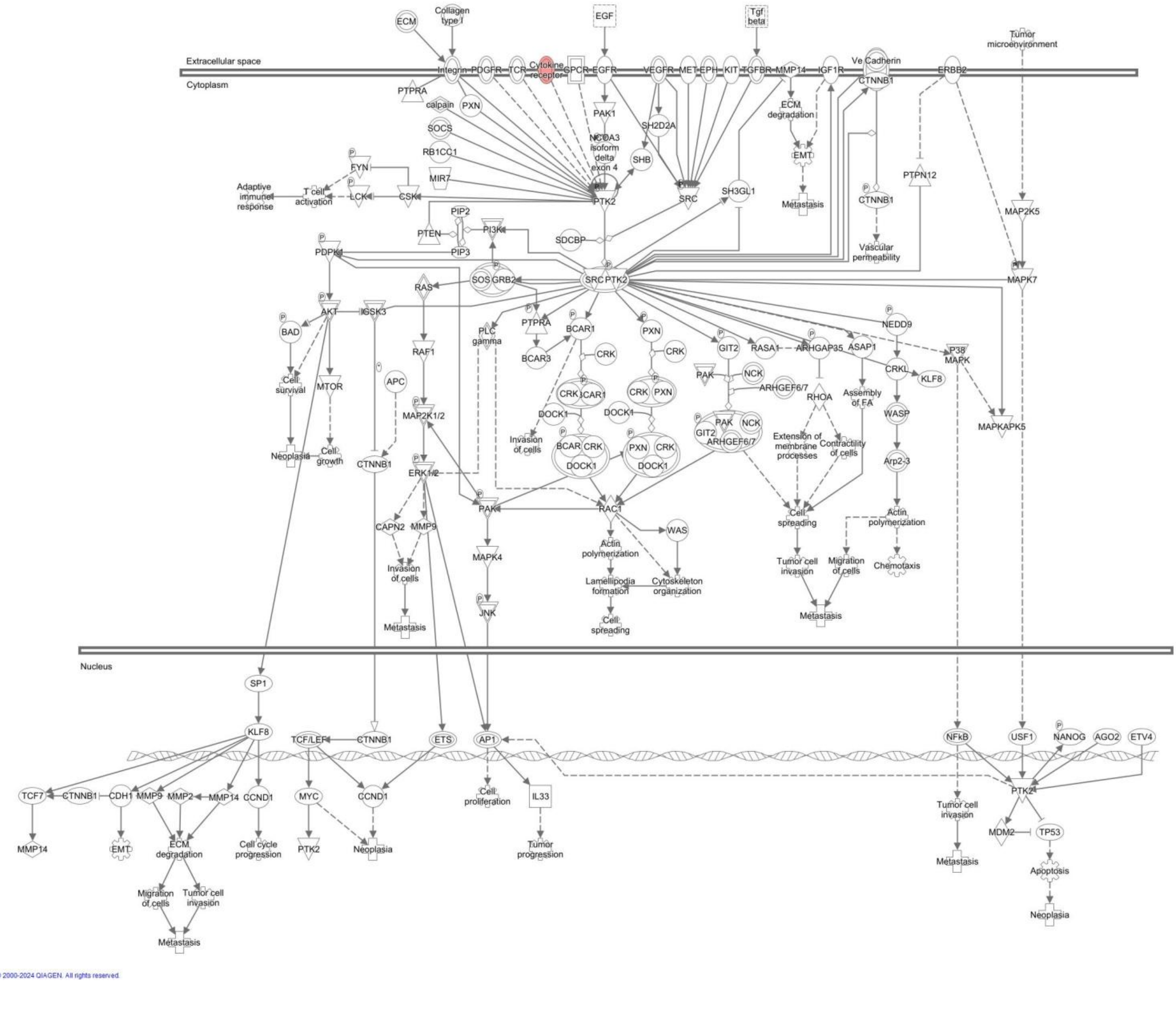

© 2000-2024 QIAGEN. All rights reserved.

Figure S71

FAK Signaling

Day 21
CAGE analysis

FAK Signaling

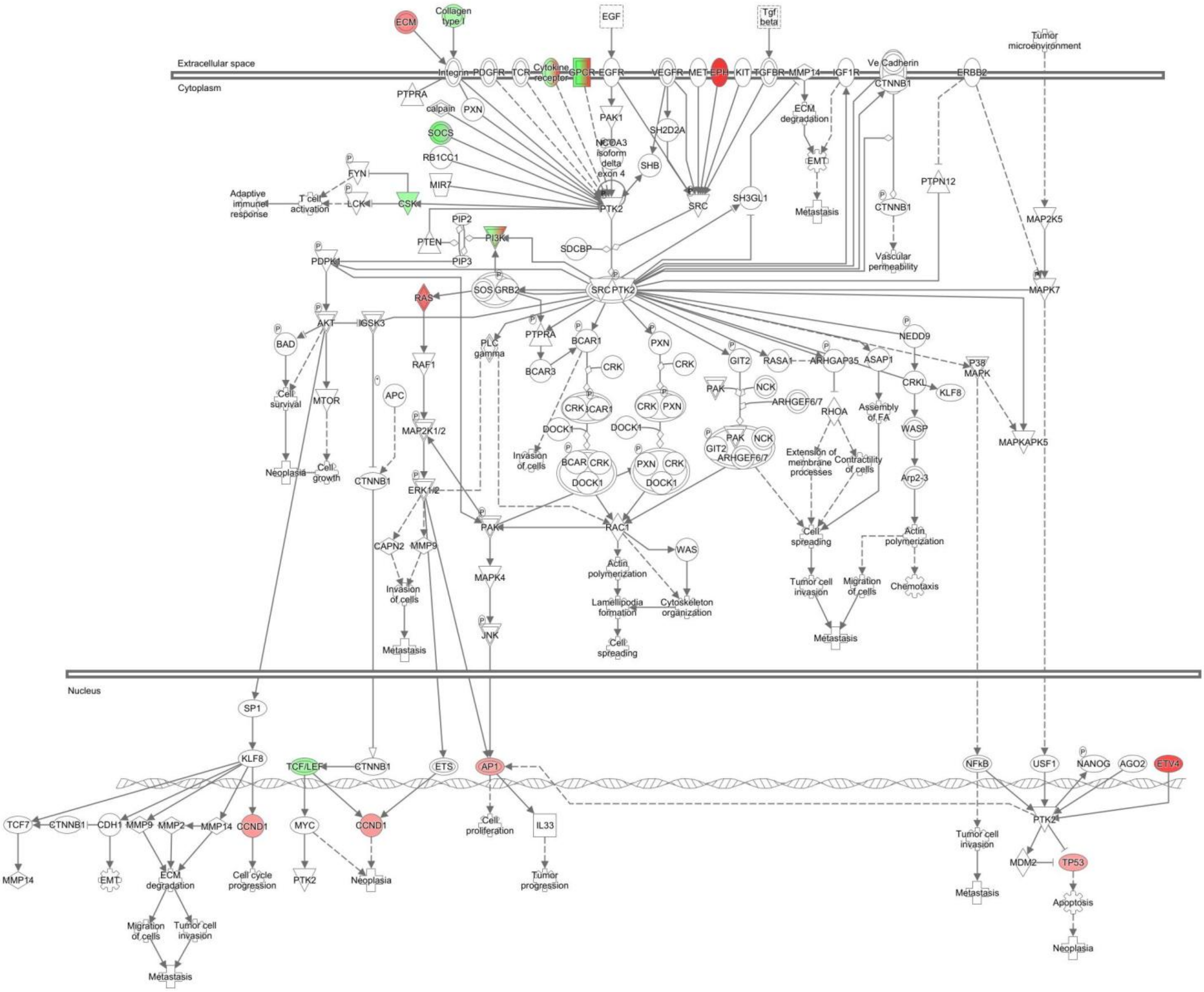

© 2000-2024 QIAGEN. All rights reserved.

Figure S72

CSDE1 Signaling Pathway

6 hours
CAGE analysis

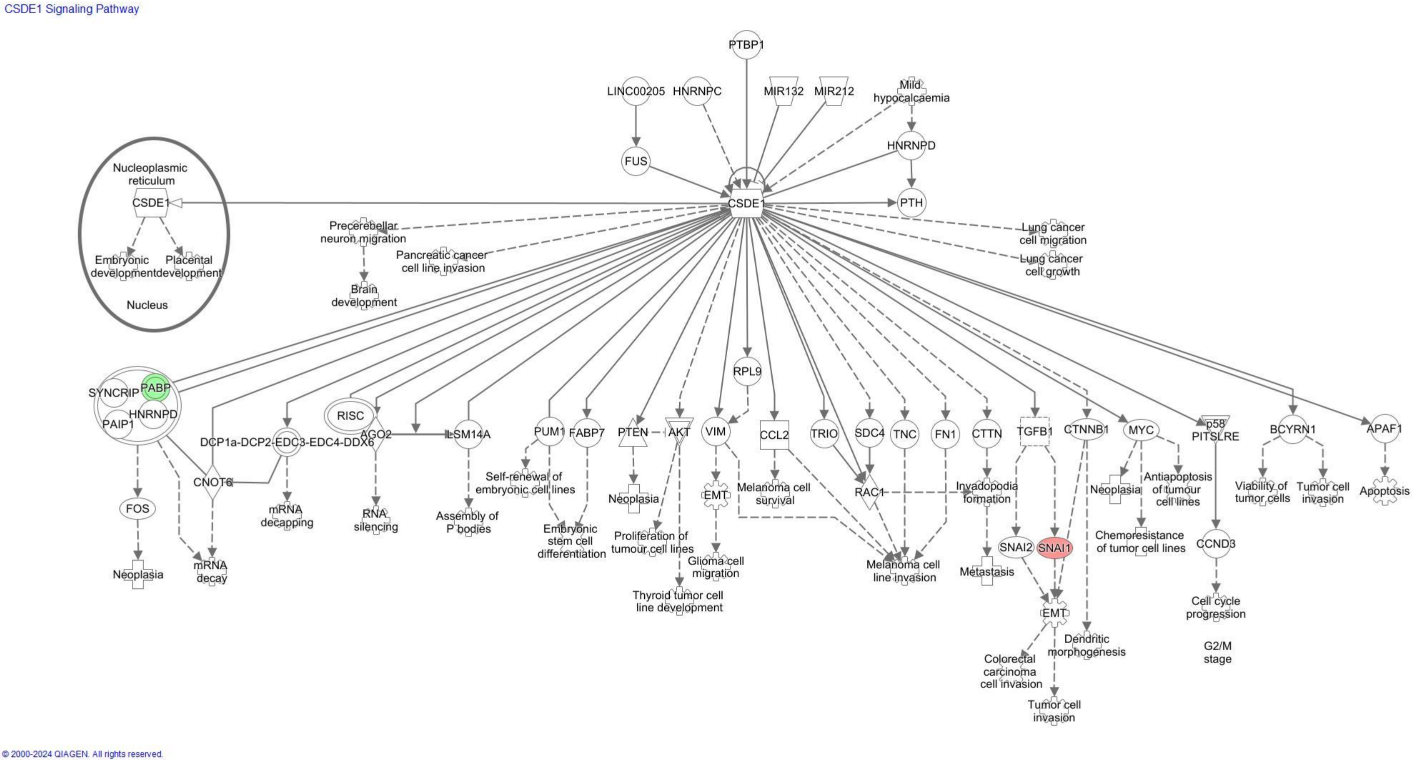

© 2000-2024 QIAGEN. All rights reserved.

Figure S73

CSDE1 Signaling Pathway

8 days
CAGE analysis

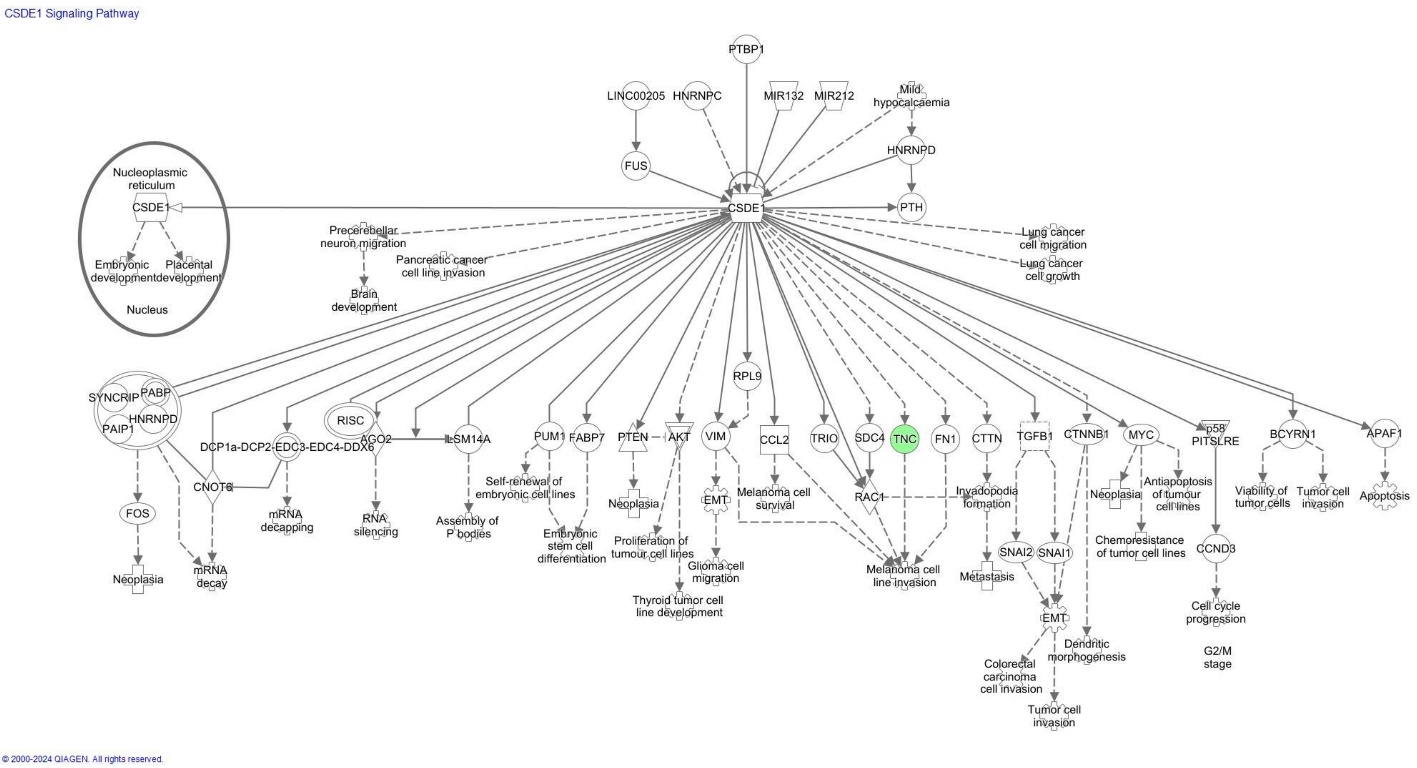

Figure S74

CSDE1 Signaling Pathway

Day 21
CAGE analysis

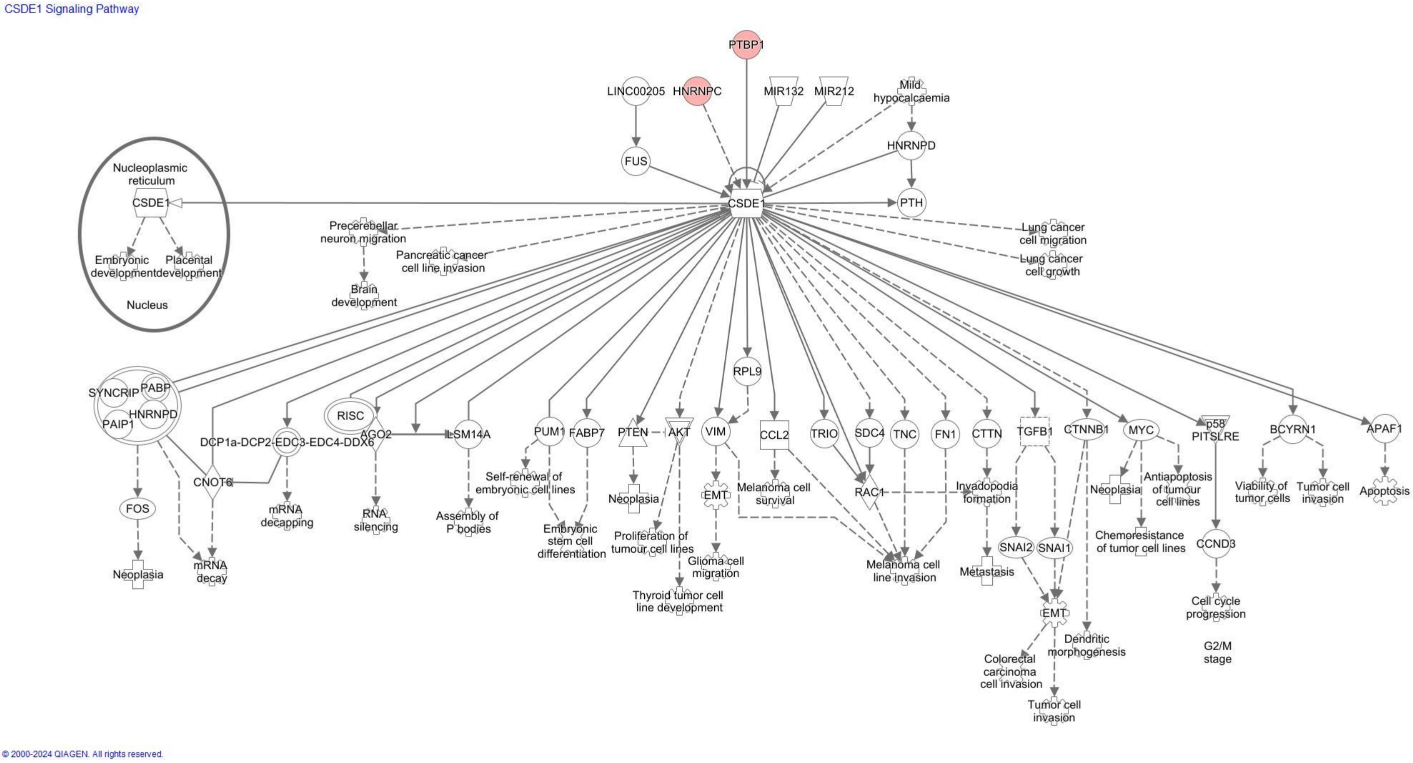

Figure S85

Cyclins and Cell Cycle Regulation

8 days
CAGE analysis

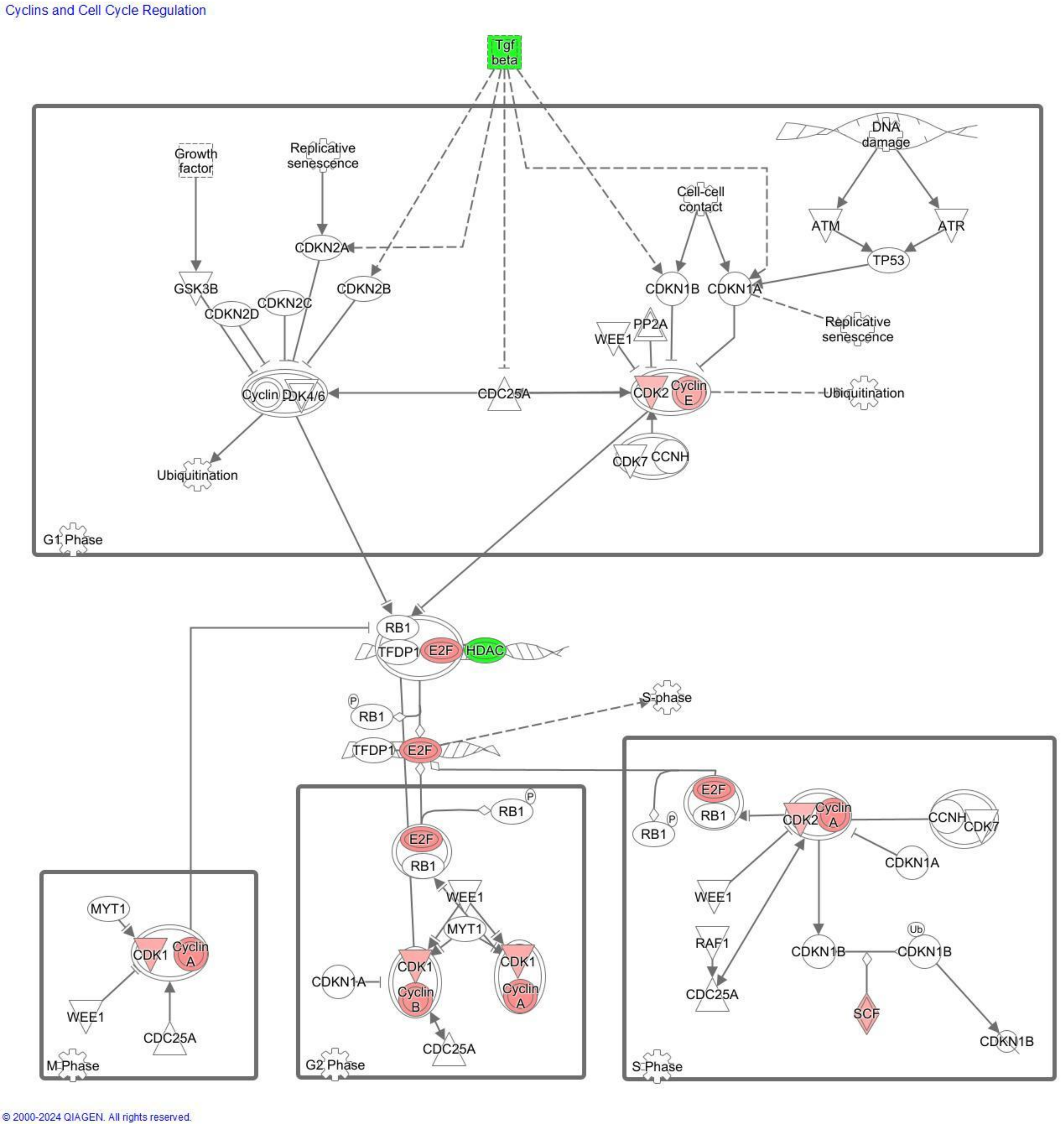

© 2000-2024 QIAGEN. All rights reserved.

Figure S76

Cyclins and Cell Cycle Regulation

Day 21
CAGE analysis

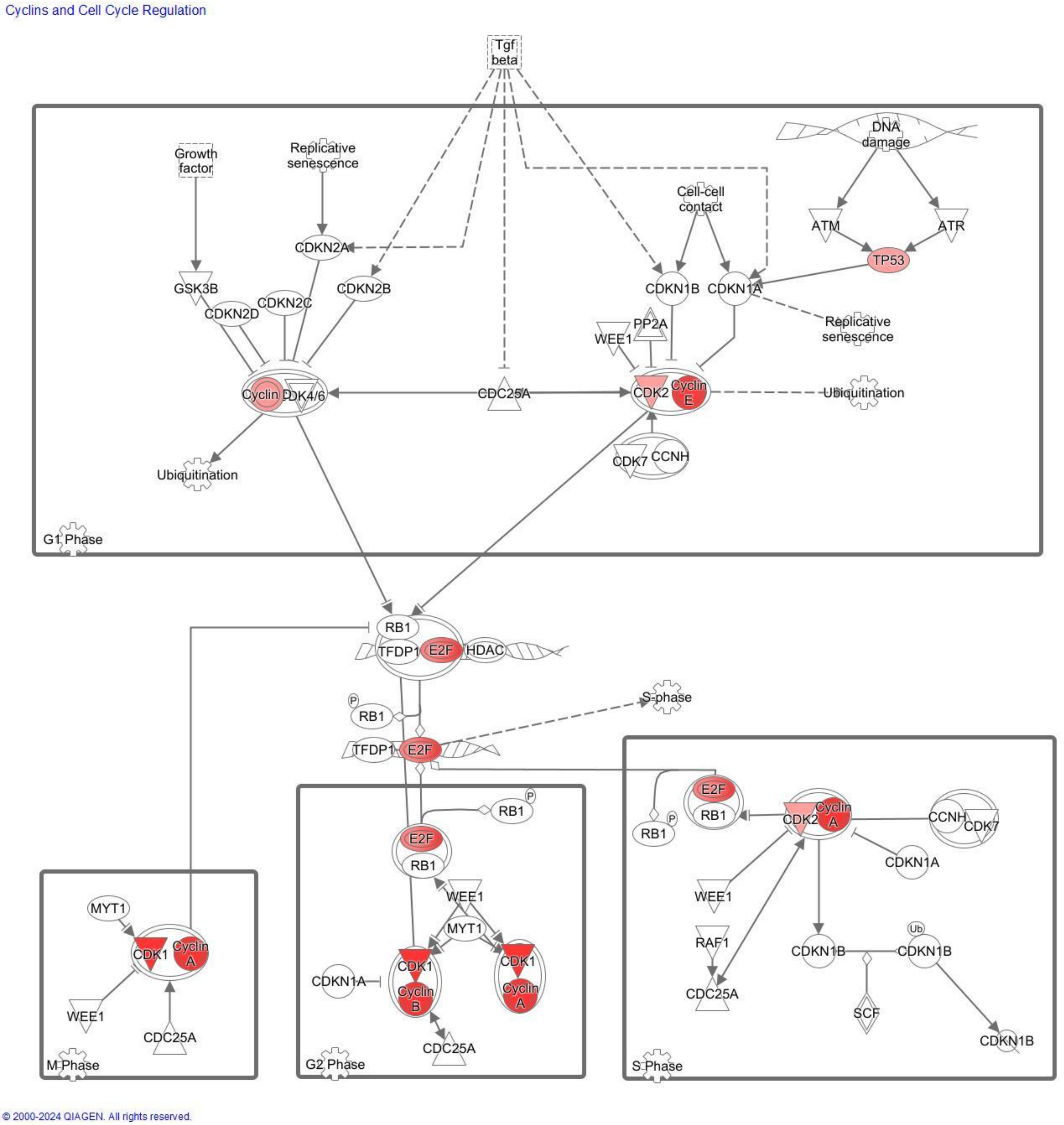

© 2000-2024 QIAGEN. All rights reserved.

Figure S77

PAK Signaling

6 hours
CAGE analysis

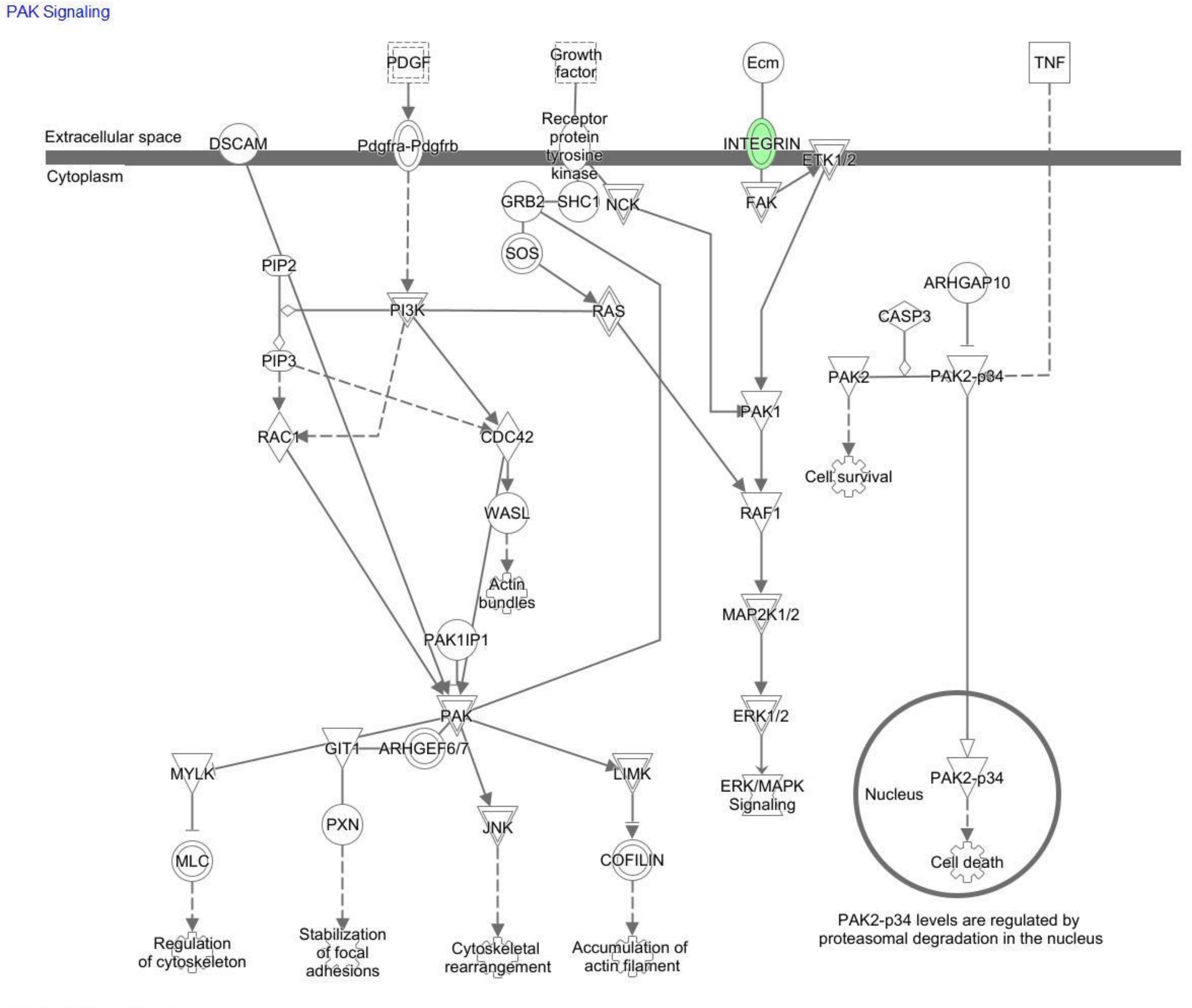

© 2000-2024 QIAGEN. All rights reserved.

Figure S78

PAK Signaling

8 days
CAGE analysis

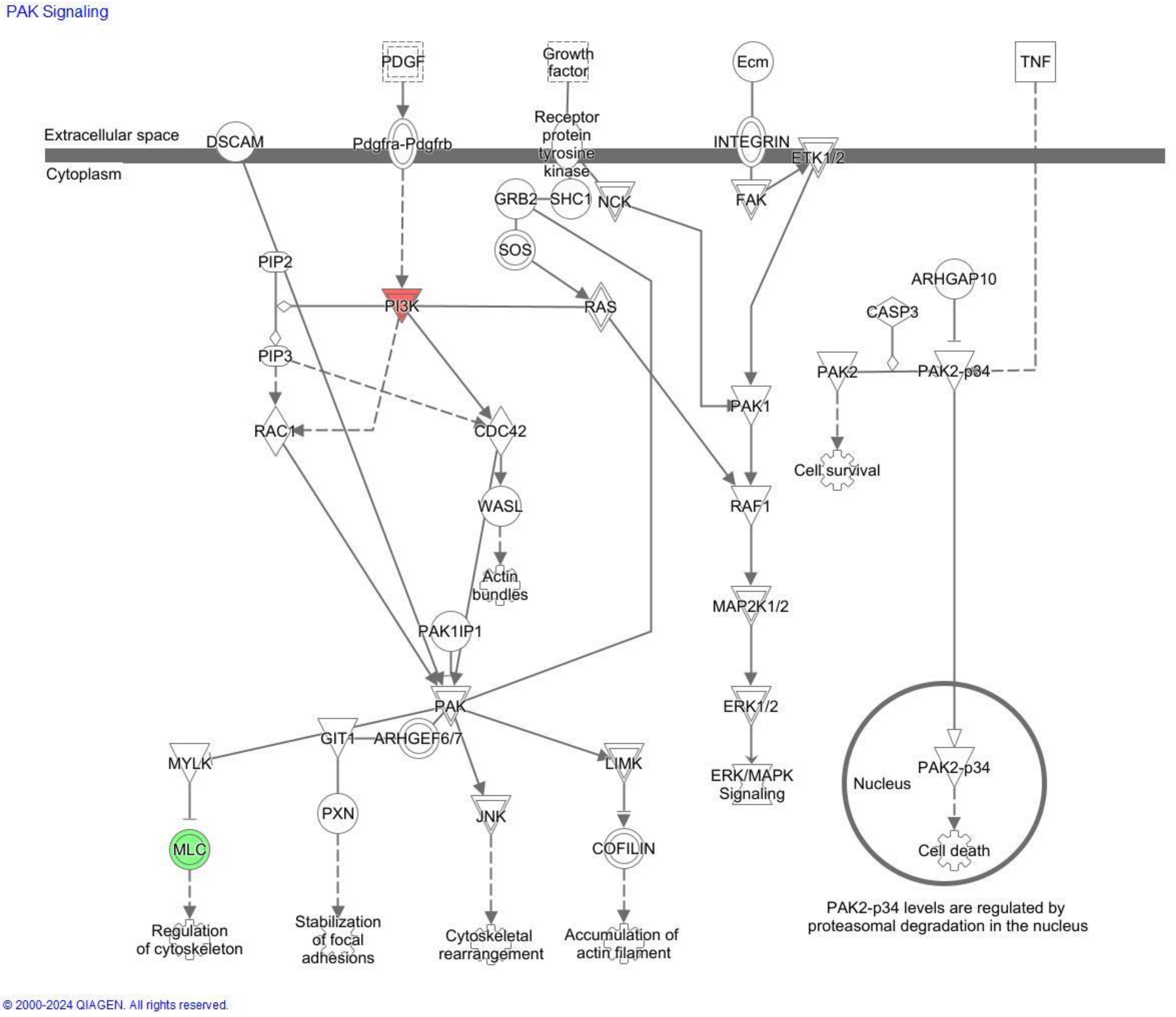

© 2000-2024 QIAGEN. All rights reserved.

Figure S79

PAK Signaling

Day 21
CAGE analysis

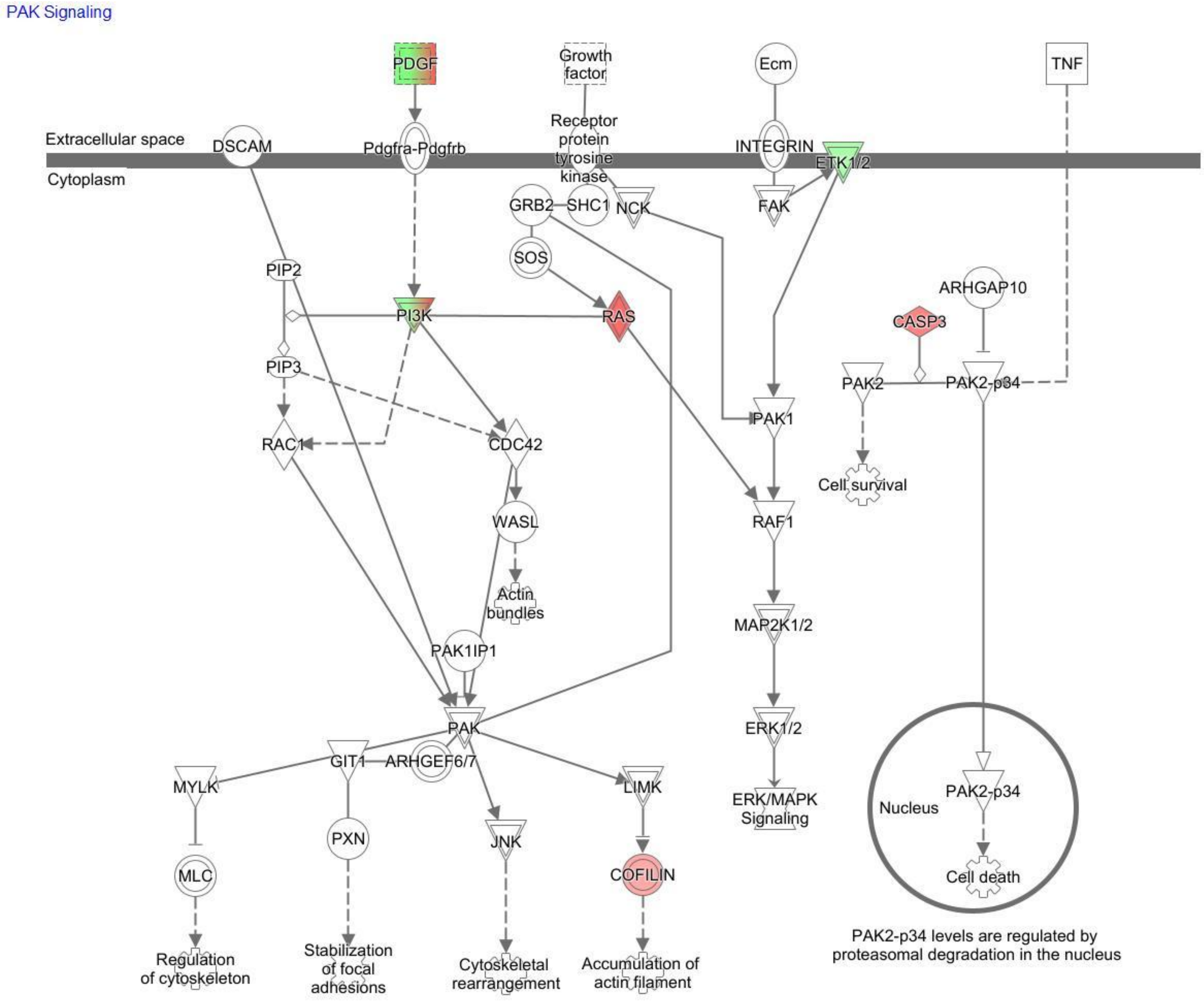

© 2000-2024 QIAGEN. All rights reserved.

Figure S80

HOTAIR Regulatory Signaling

1 hour
CAGE analysis

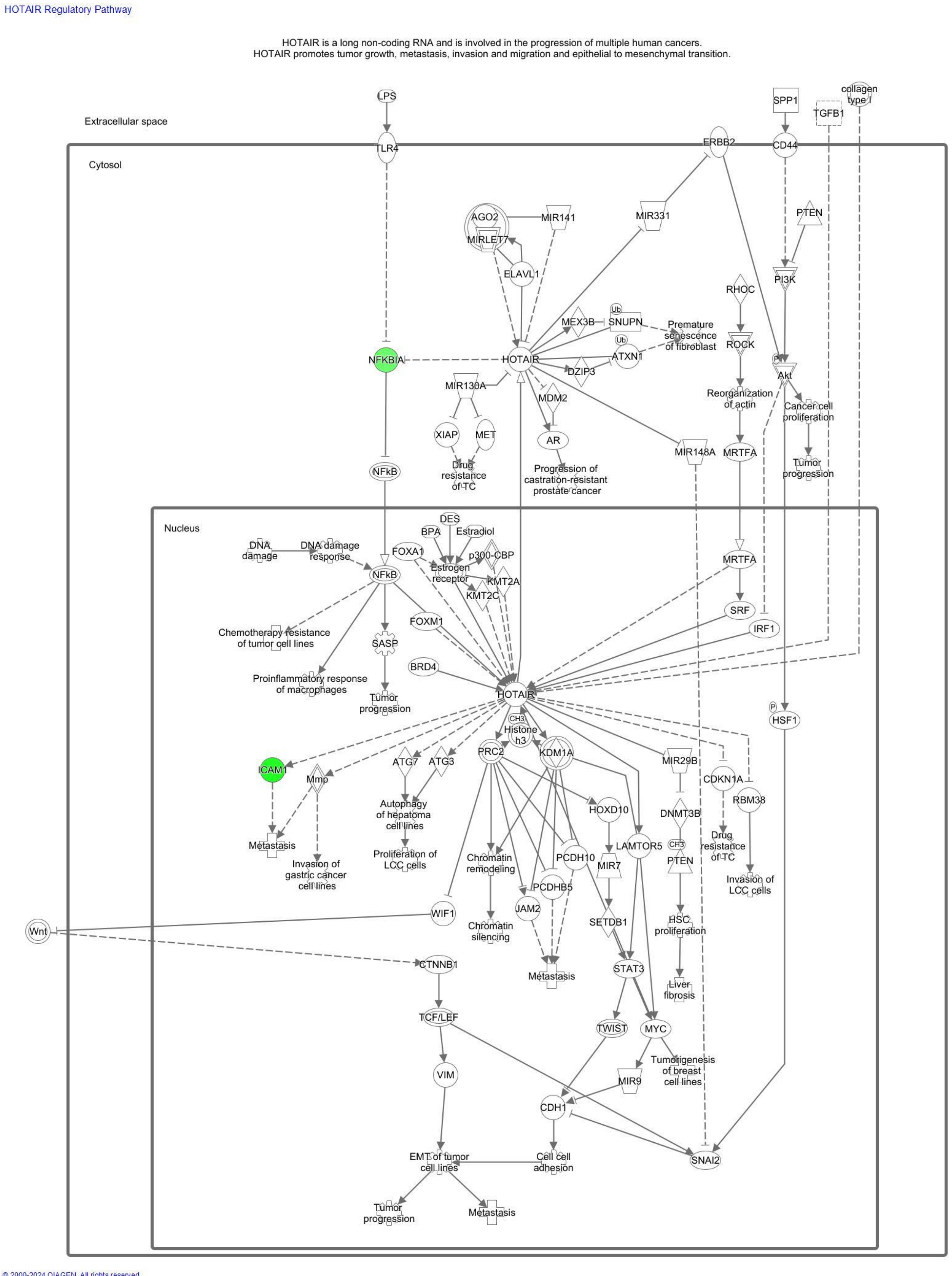

© 2000-2024 QIAGEN. All rights reserved.

Figure S81

HOTAIR Regulatory Signaling

8 days
CAGE analysis

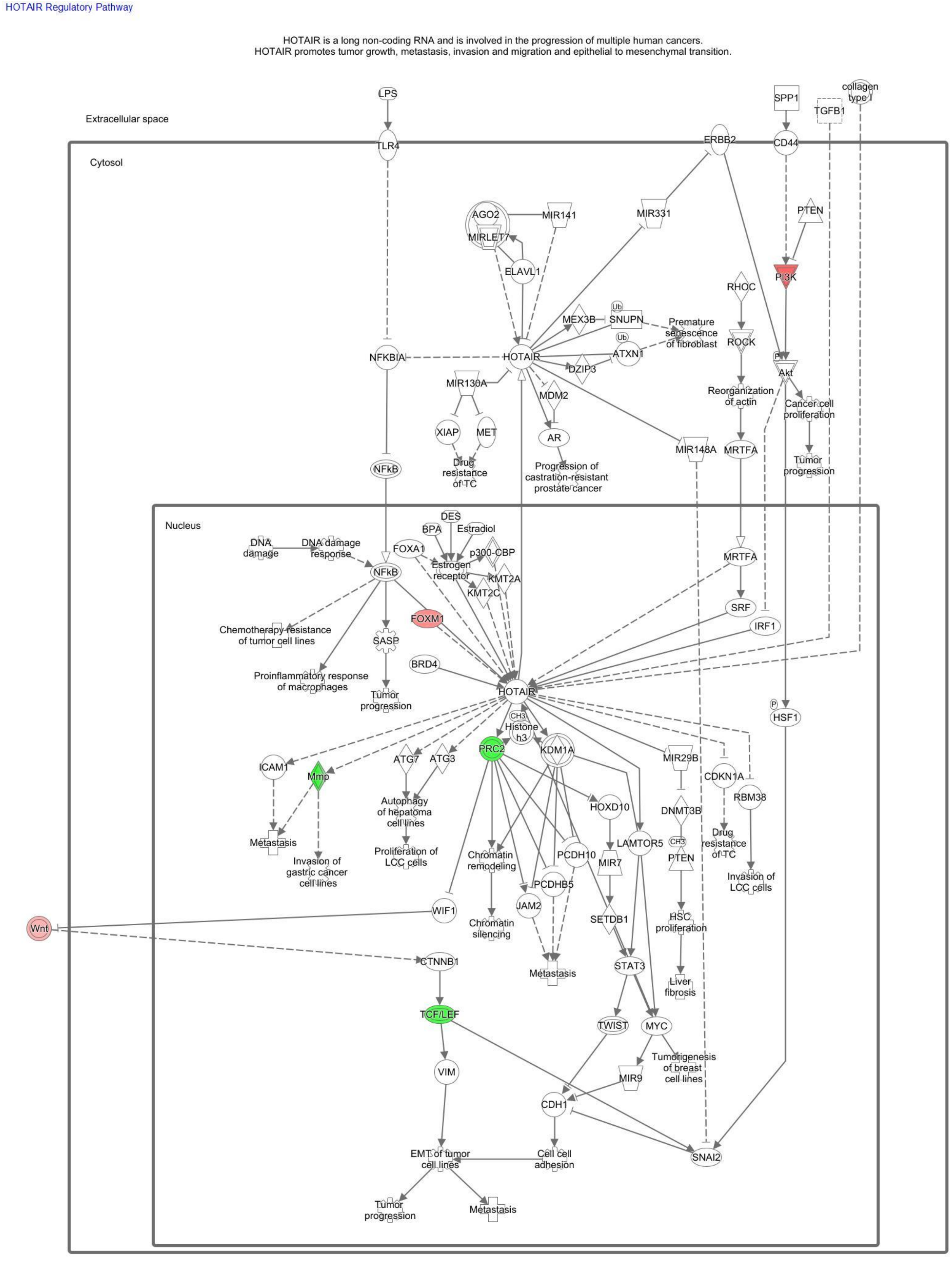

© 2000-2024 QIAGEN. All rights reserved.

Figure S82

HOTAIR Regulatory Signaling

Day 21
CAGE analysis

HOTAIR Regulatory Pathway

HOTAIR is a long non-coding RNA and is involved in the progression of multiple human cancers.
HOTAIR promotes tumor growth, metastasis, invasion and migration and epithelial to mesenchymal transition.

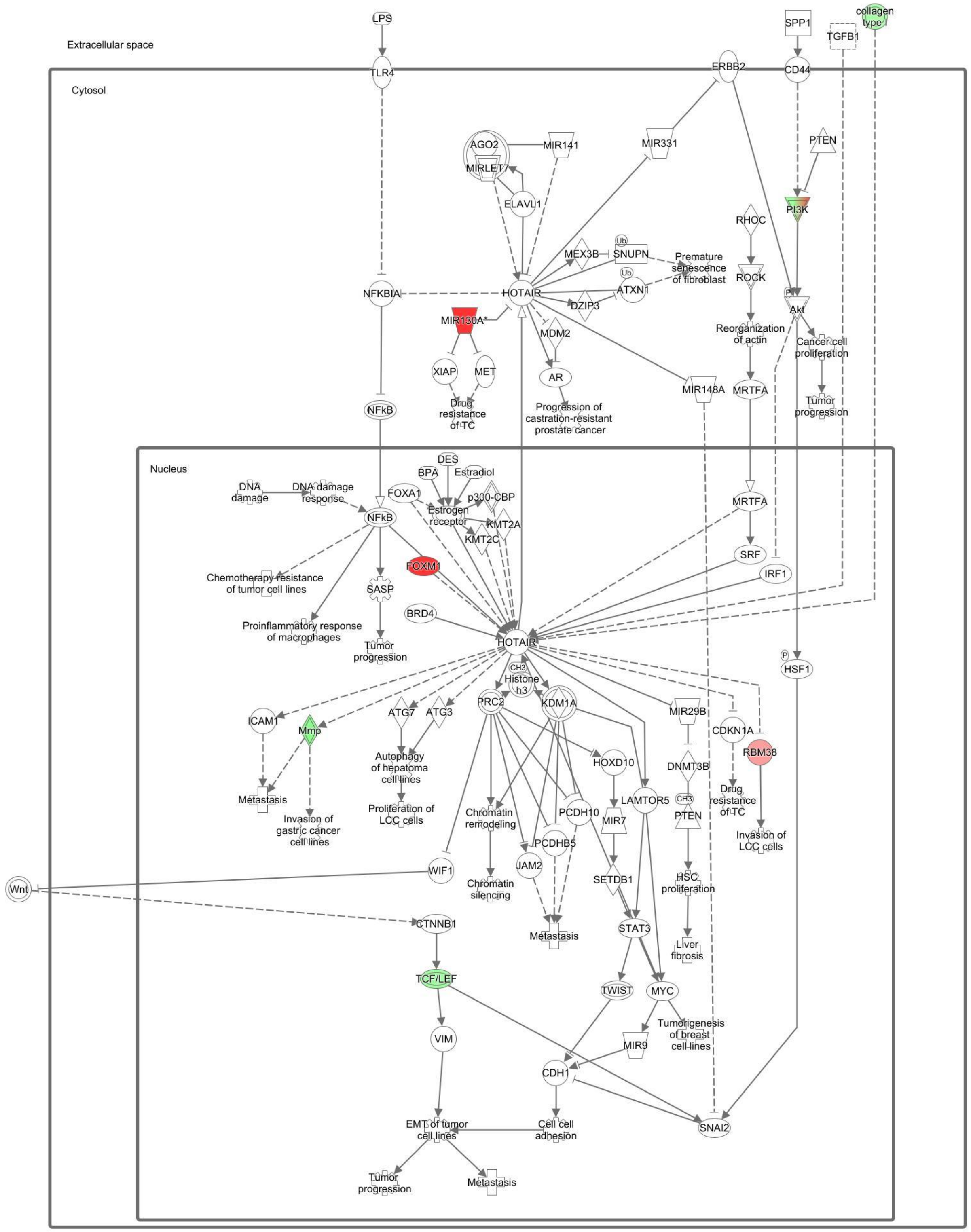

© 2000-2024 QIAGEN. All rights reserved.

Figure S83

EGF Signaling

24 hours
CAGE analysis

EGF Signaling : NC24h-PA24h_FDR0.05_log2(PFOAvsNC)_Gene : Expr Log Ratio

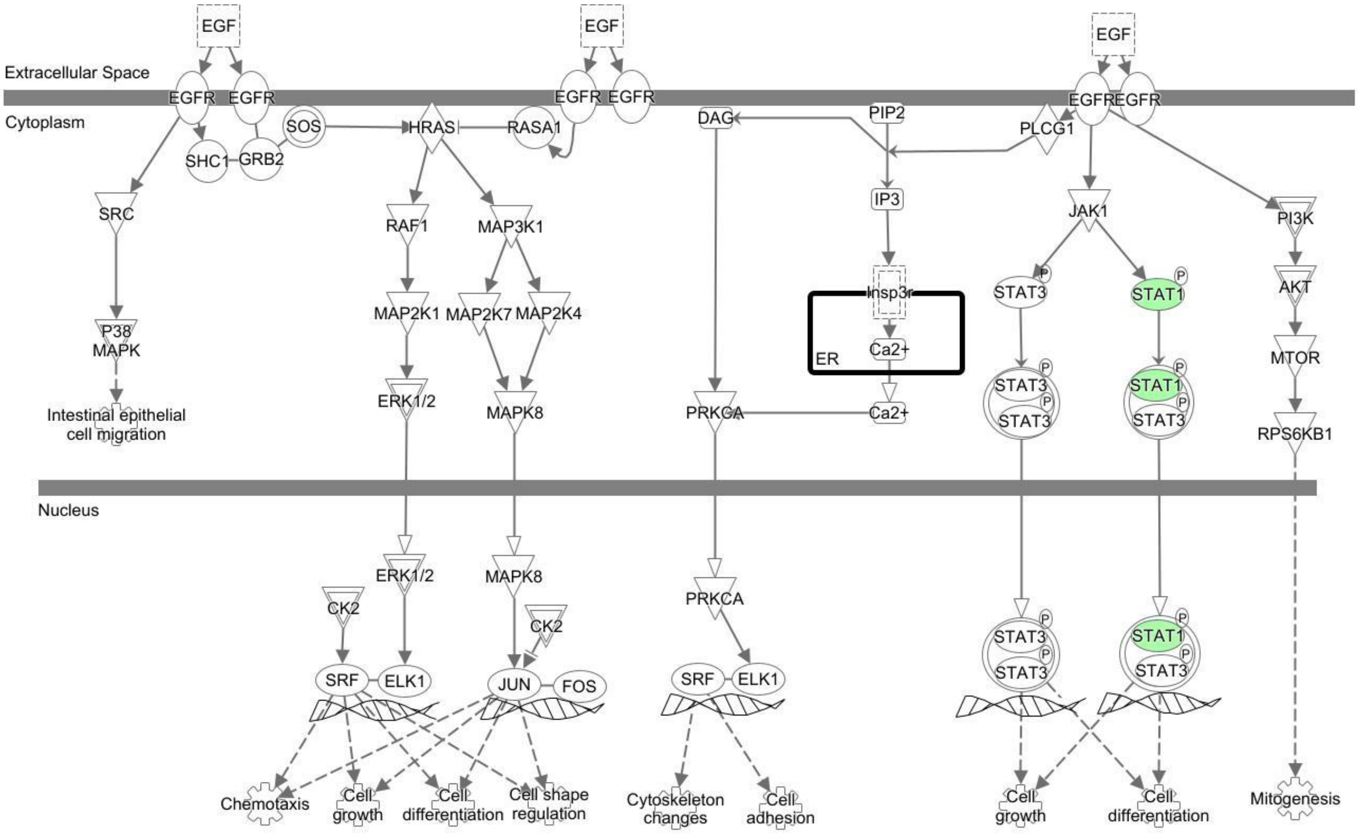

© 2000-2024 QIAGEN. All rights reserved.

Figure S84

EGF Signaling

8 days
CAGE analysis

EGF Signaling : NC12d-PA12d_FDR0.05_log2(PFOAvsNC)_Gene : Expr Log Ratio

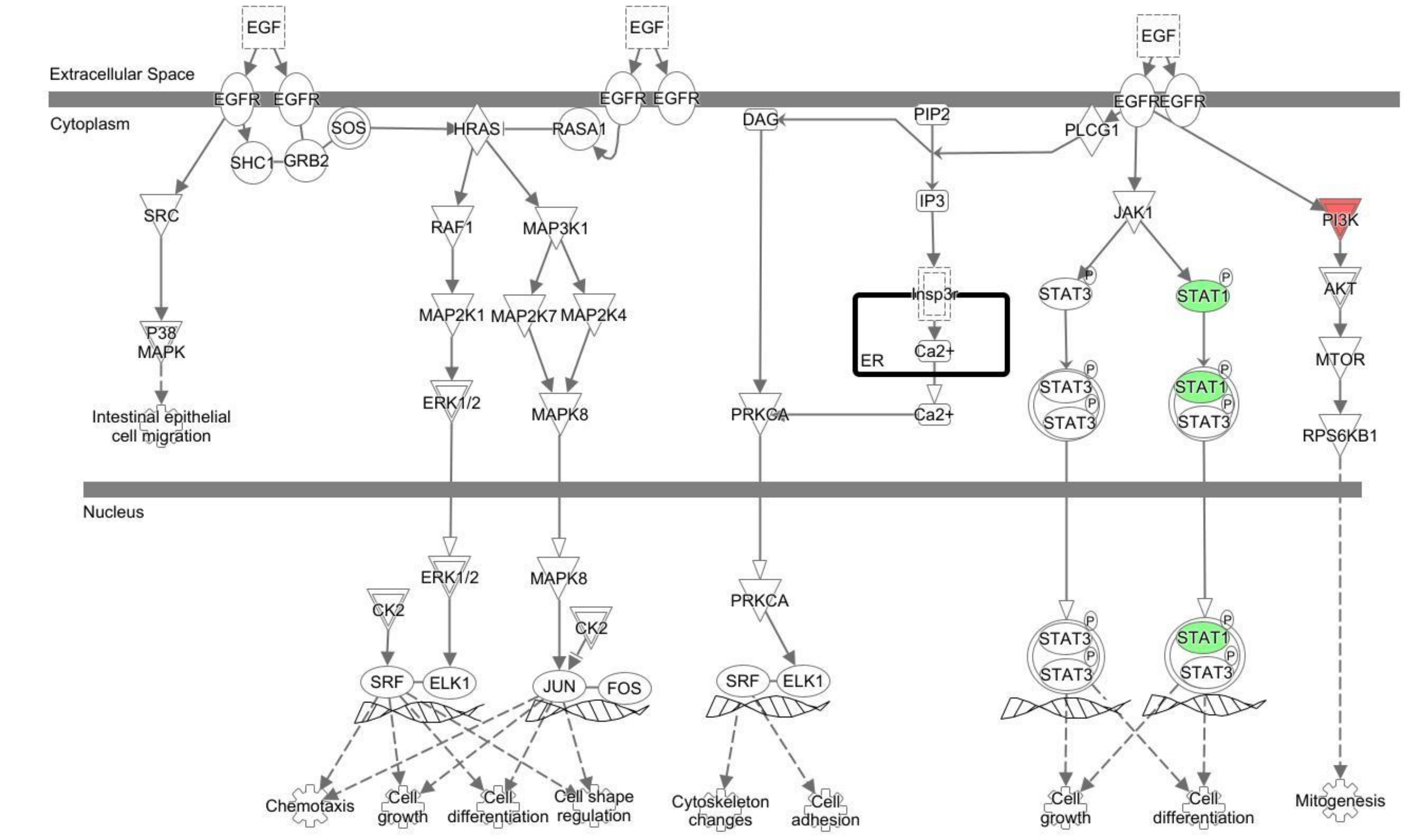

© 2000-2024 QIAGEN. All rights reserved.

Figure S85

EGF Signaling

Day 21
CAGE analysis

EGF Signaling : NC21d-PA21d_FDR0.05_log2(PFOAvsNC)_Gene : Expr Log Ratio

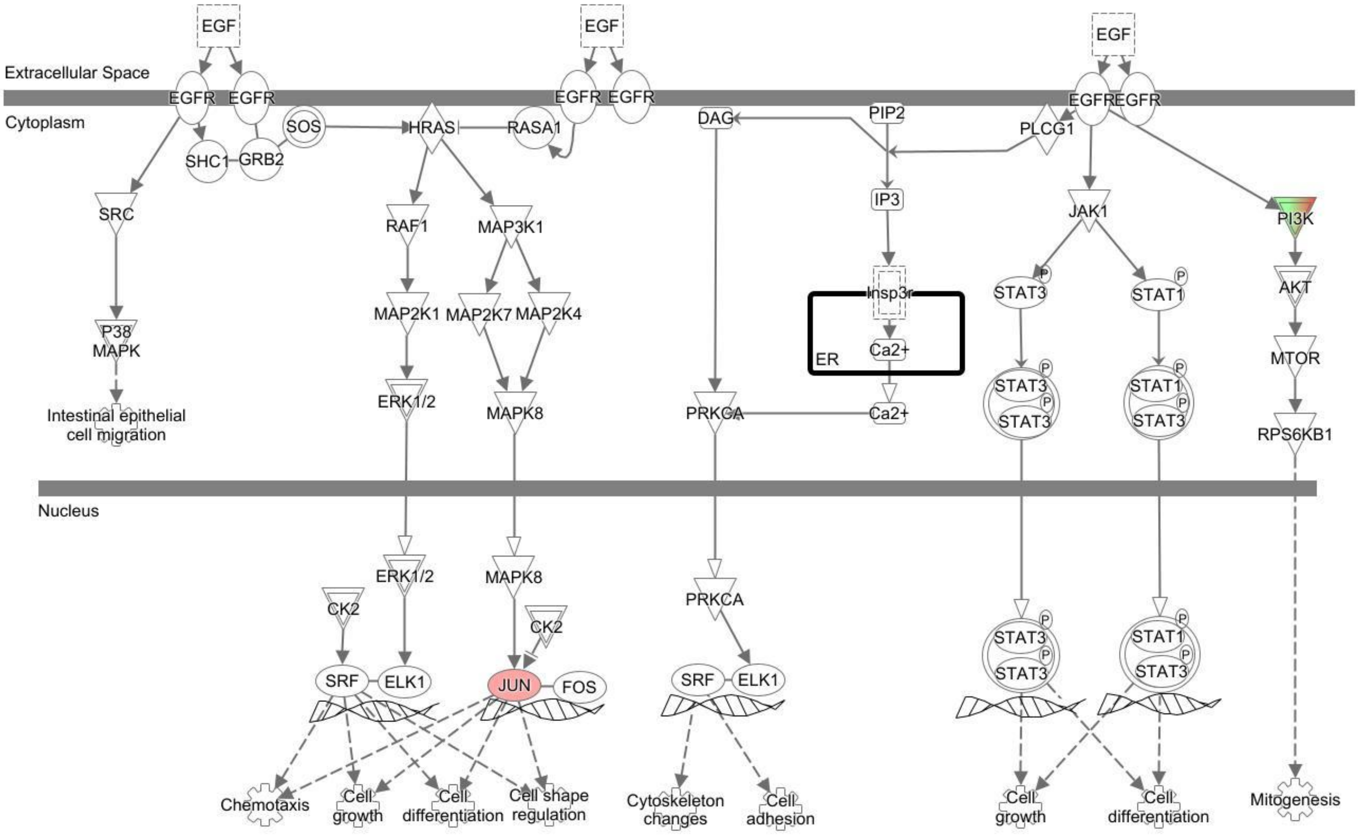

© 2000-2024 QIAGEN. All rights reserved.

Figure S86

FGF Signaling

24 hours
CAGE analysis

FGF Signaling : NC24h-PA24h_FDR0.05_log2(PFOAvsNC)_Gene : Expr Log Ratio

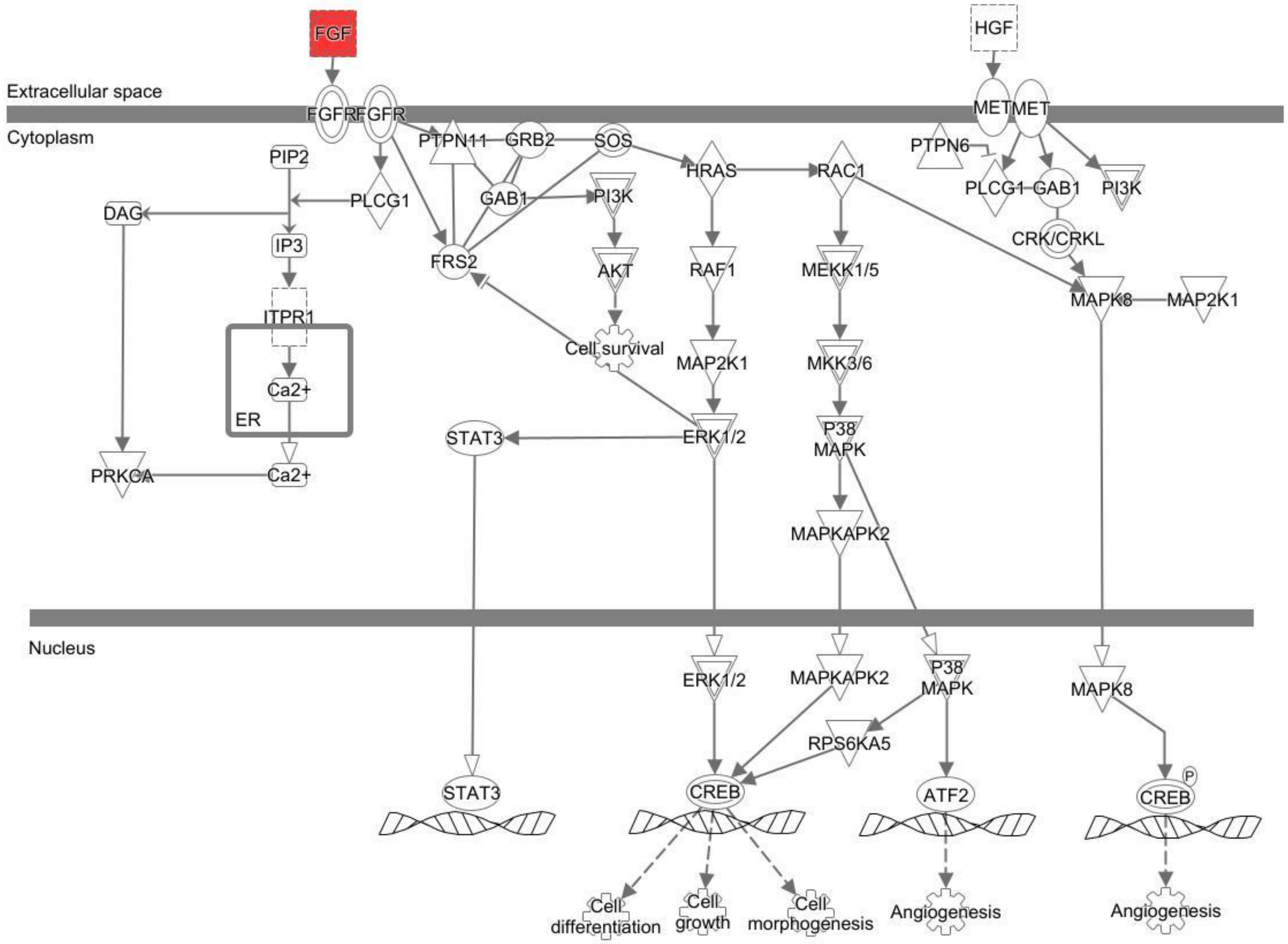

© 2000-2024 QIAGEN. All rights reserved.

Figure S87

FGF Signaling

8 days
CAGE analysis

FGF Signaling : NC12d-PA12d_FDR0.05_log2(PFOAvsNC)_Gene : Expr Log Ratio

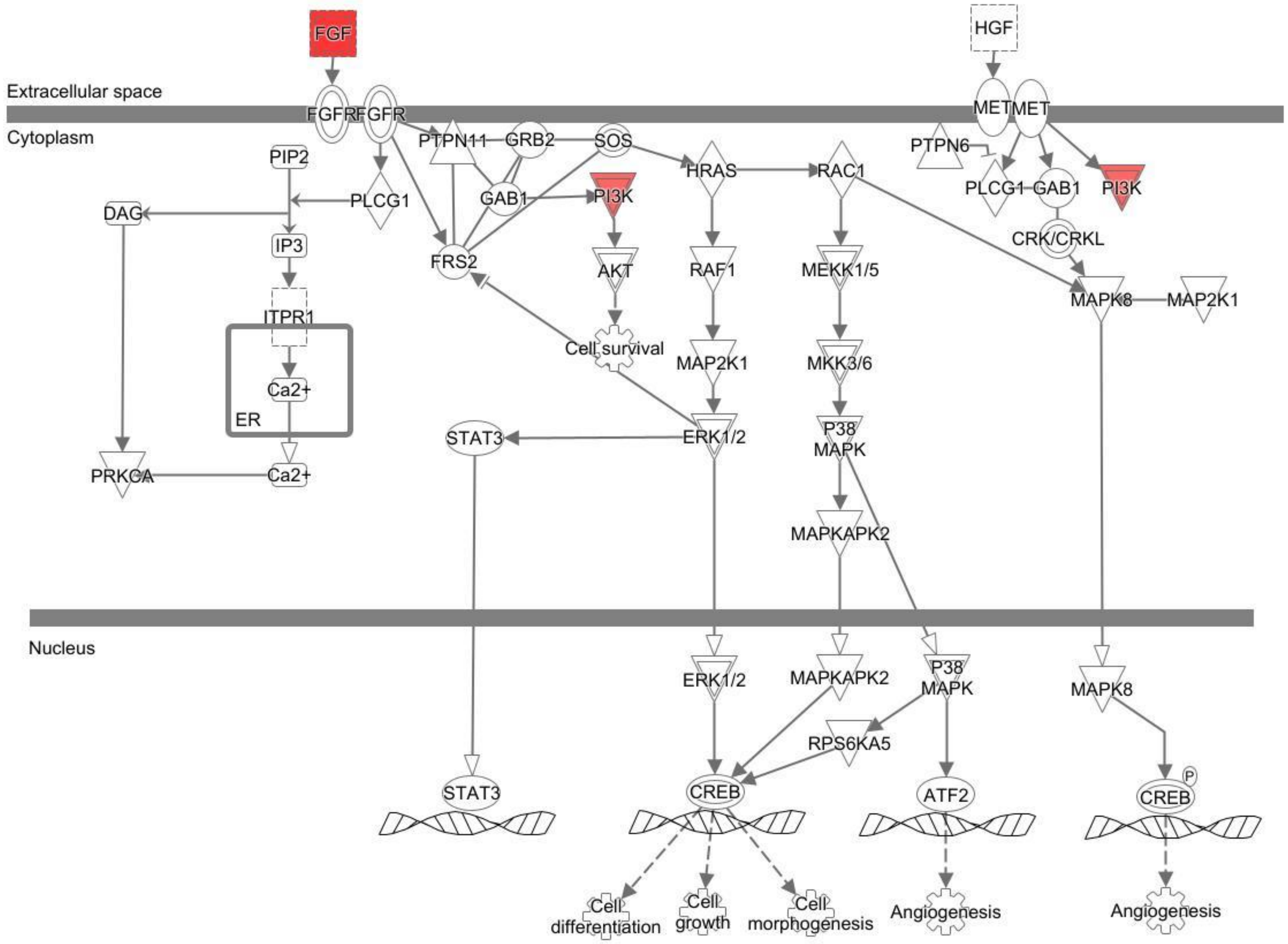

© 2000-2024 QIAGEN. All rights reserved.

Figure S88

FGF Signaling

Day 21
CAGE analysis

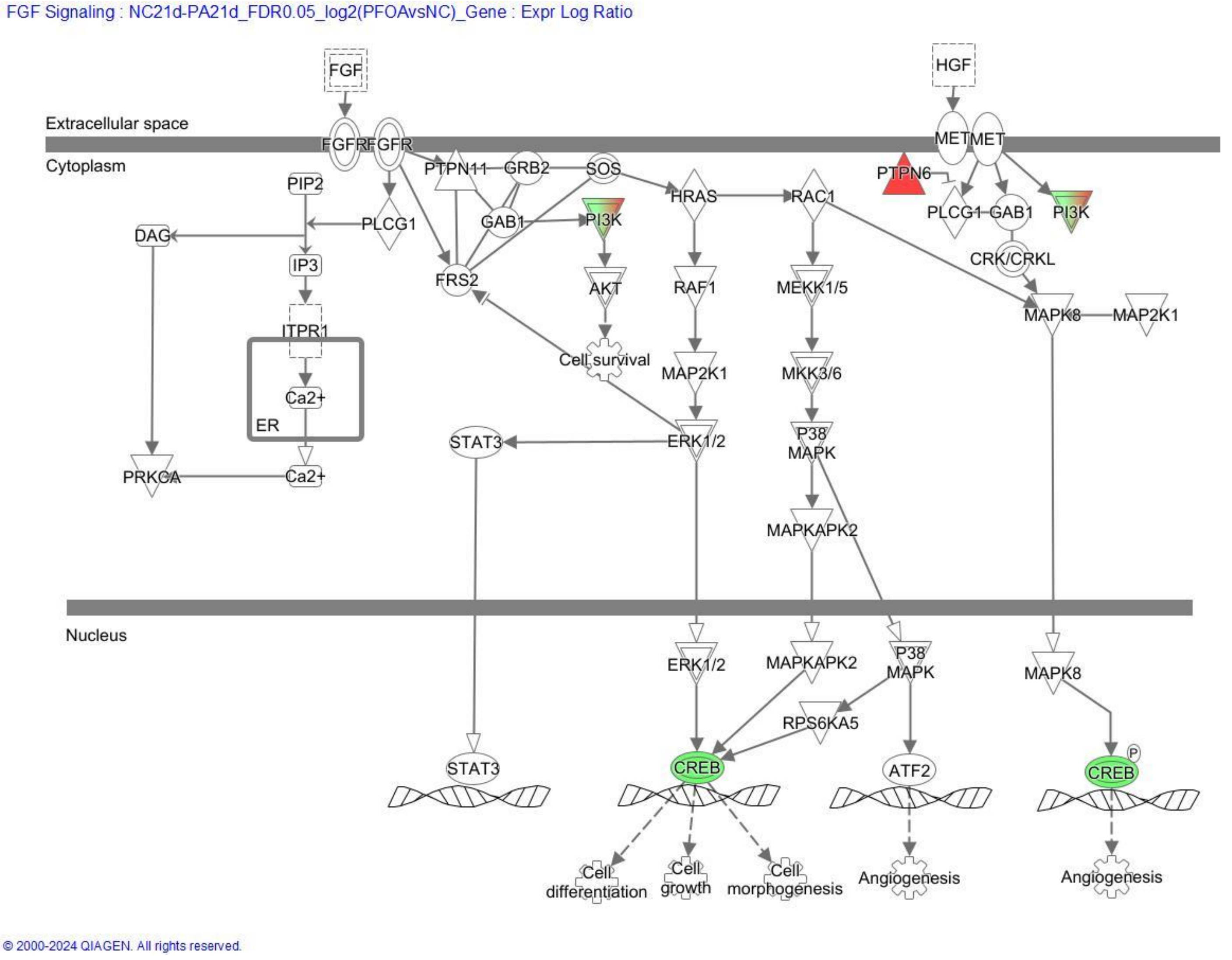

© 2000-2024 QIAGEN. All rights reserved.

Figure S89

IGF Signaling

6 hours
CAGE analysis

IGF-1 Signaling : NC6h-PA6h_FDR0.05_log2(PFOAvsNC)_Gene : Expr Log Ratio

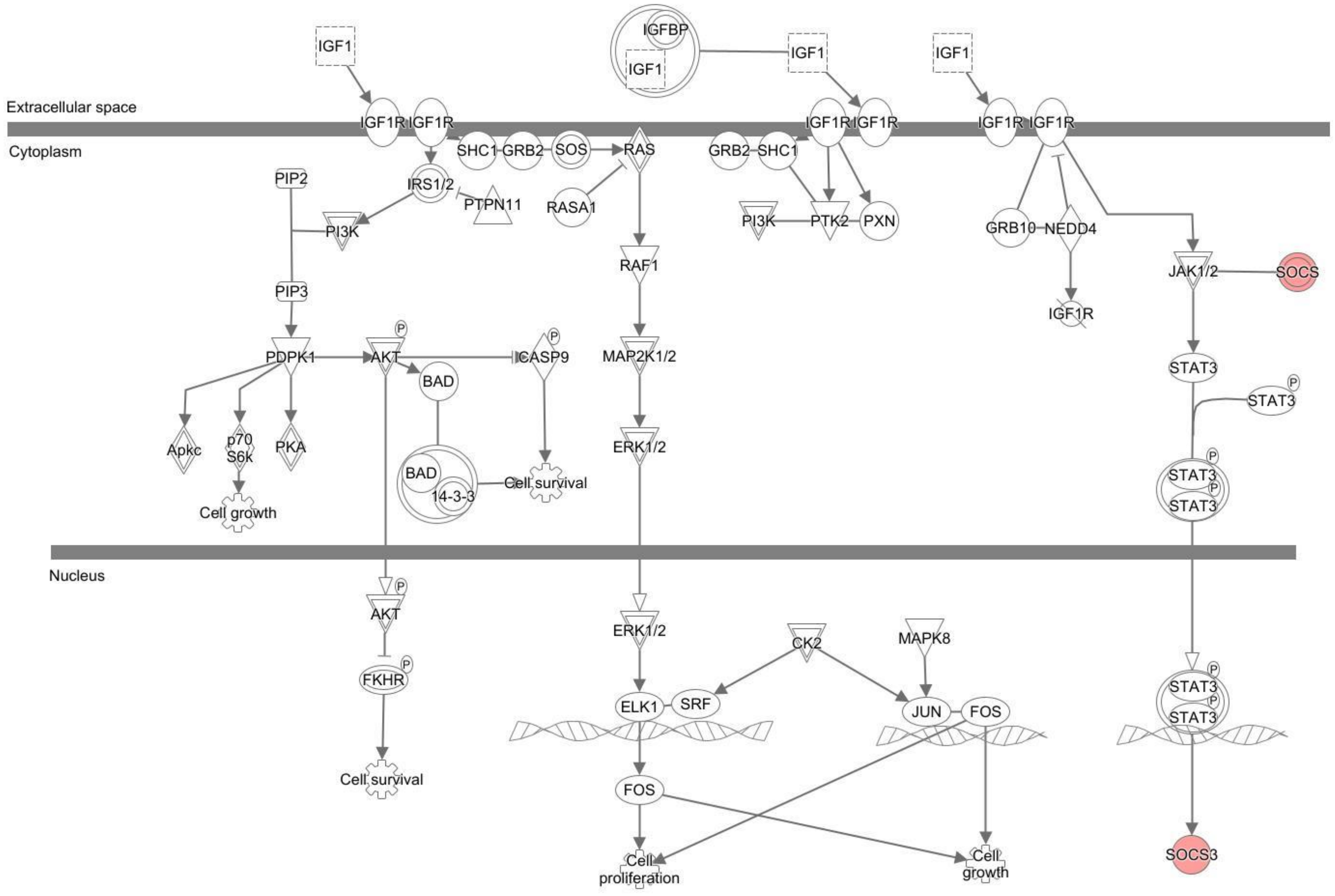

© 2000-2024 QIAGEN. All rights reserved.

Figure S90

IGF Signaling

8 days
CAGE analysis

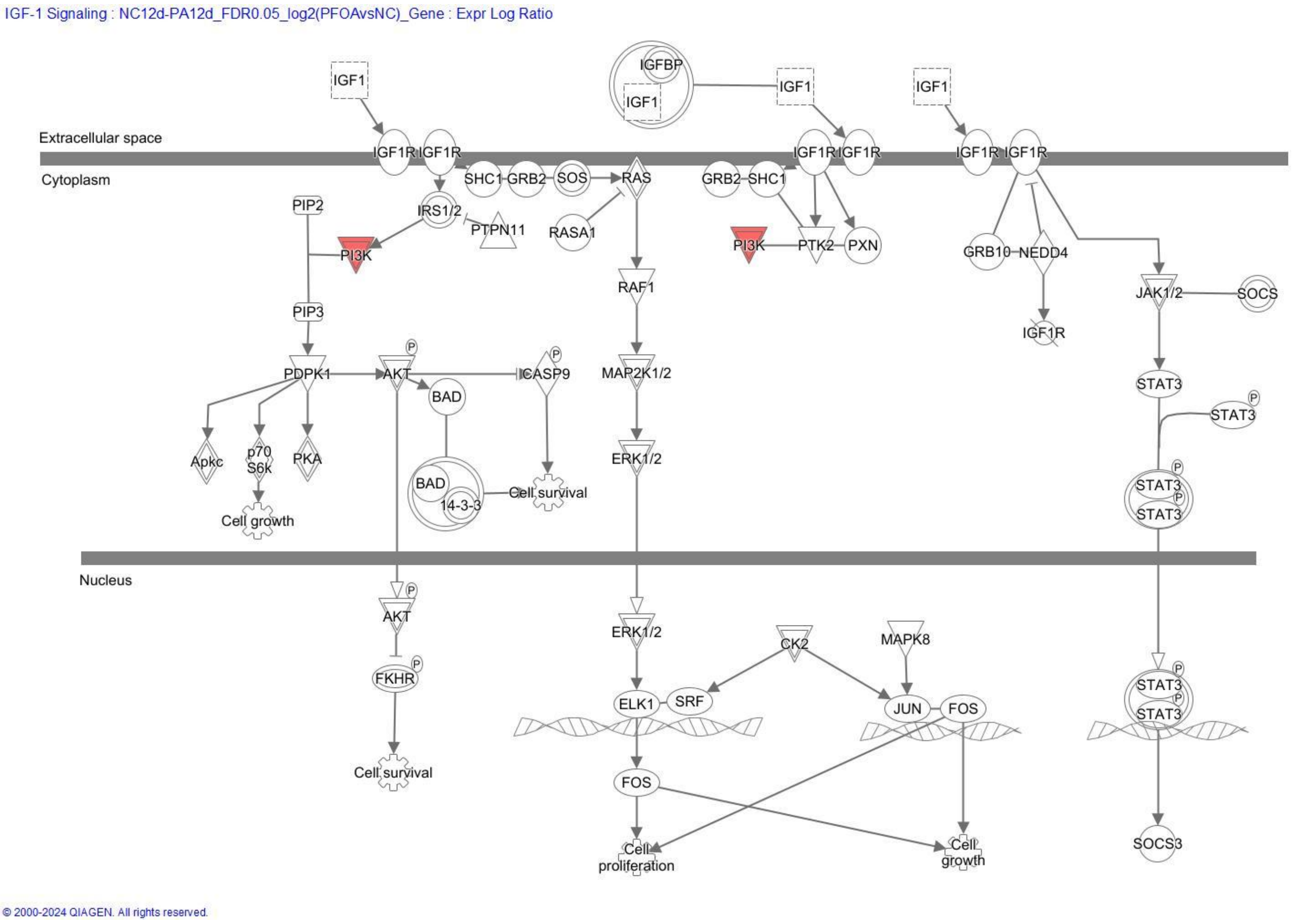

© 2000-2024 QIAGEN. All rights reserved.

Figure S91

IGF Signaling

Day 21
CAGE analysis

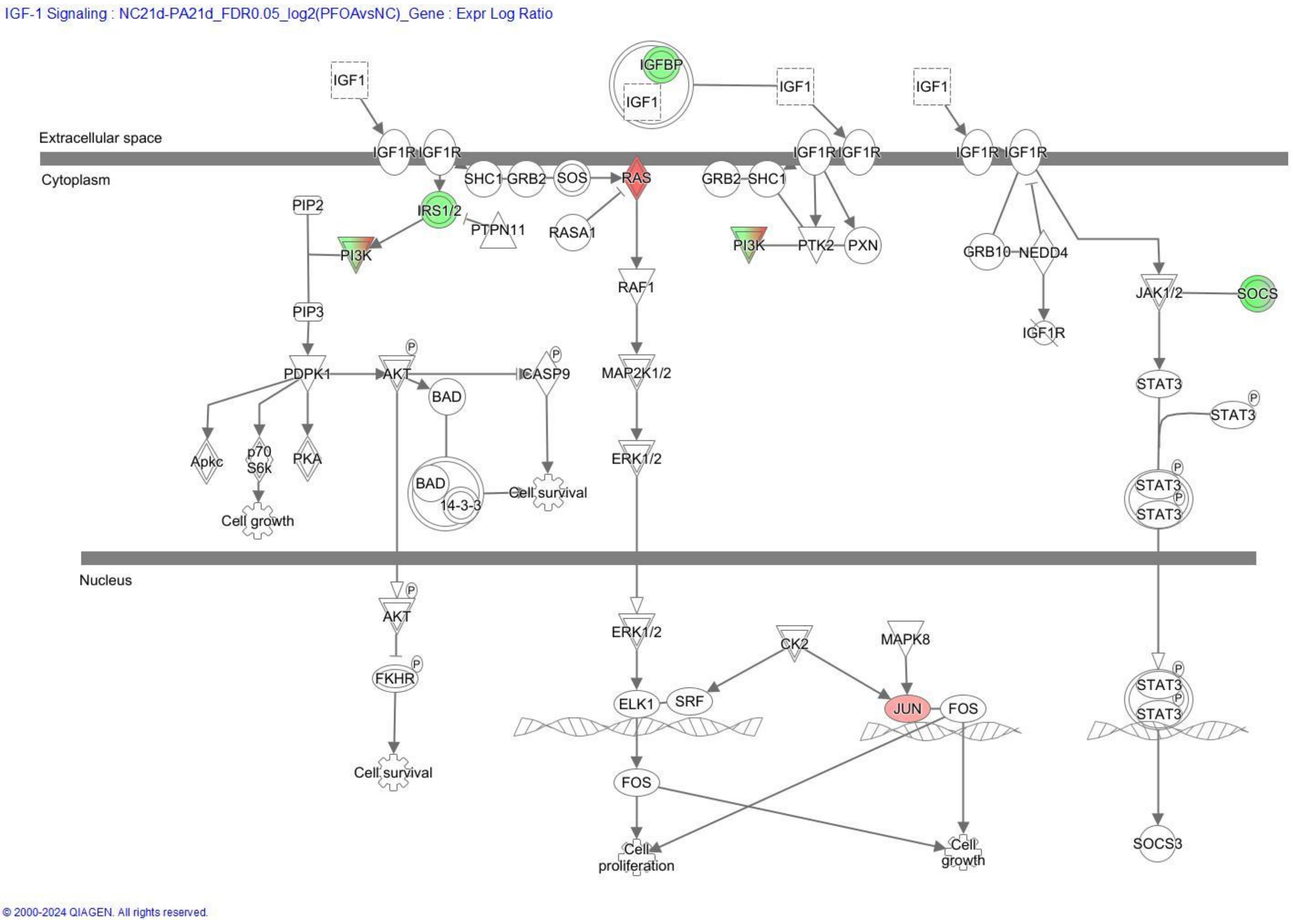

© 2000-2024 QIAGEN. All rights reserved.

Figure S92

TGF-β Signaling

6 hours
CAGE analysis

TGF-β Signaling

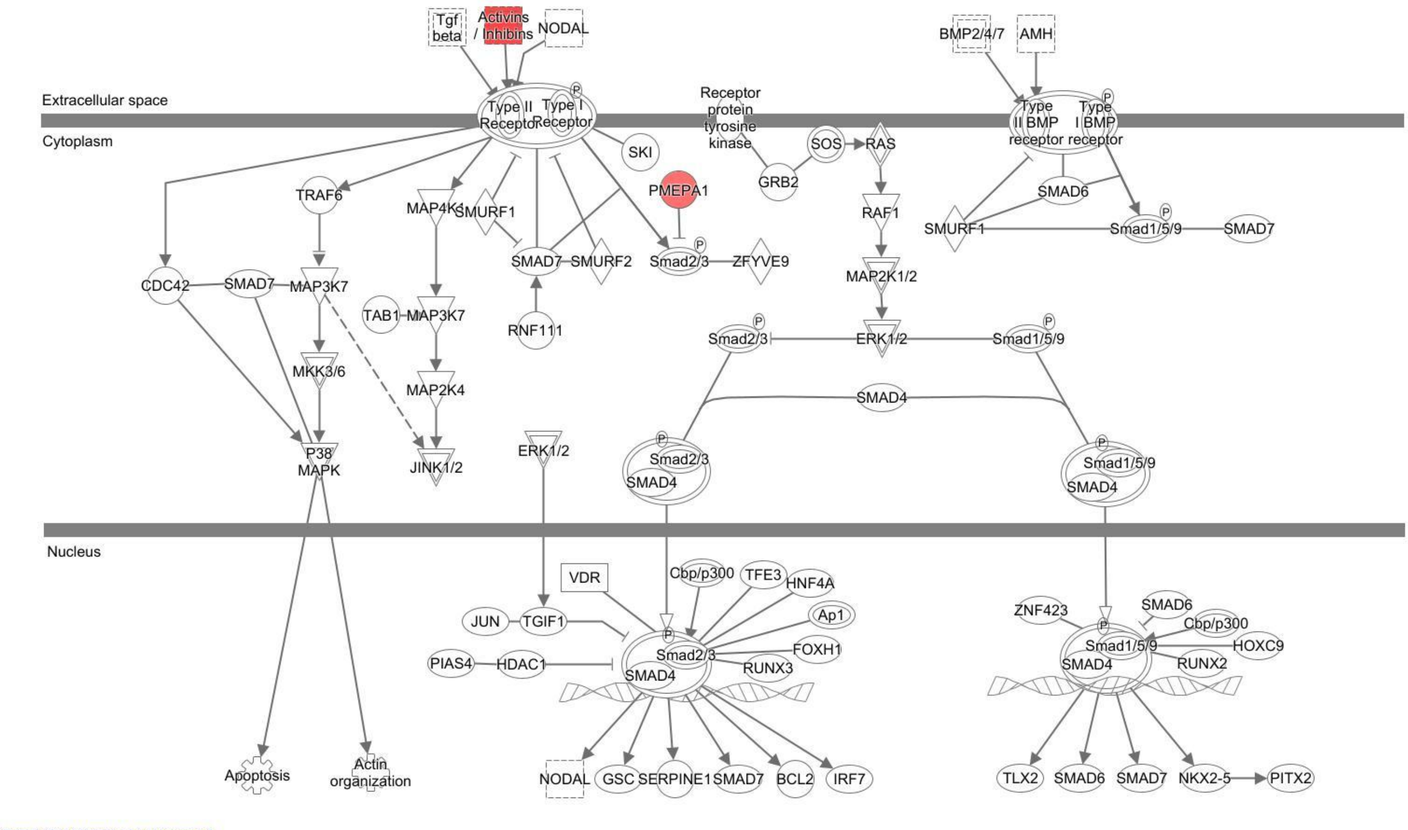

© 2000-2024 QIAGEN. All rights reserved.

Figure S93

TGF-β Signaling

24 hours
CAGE analysis

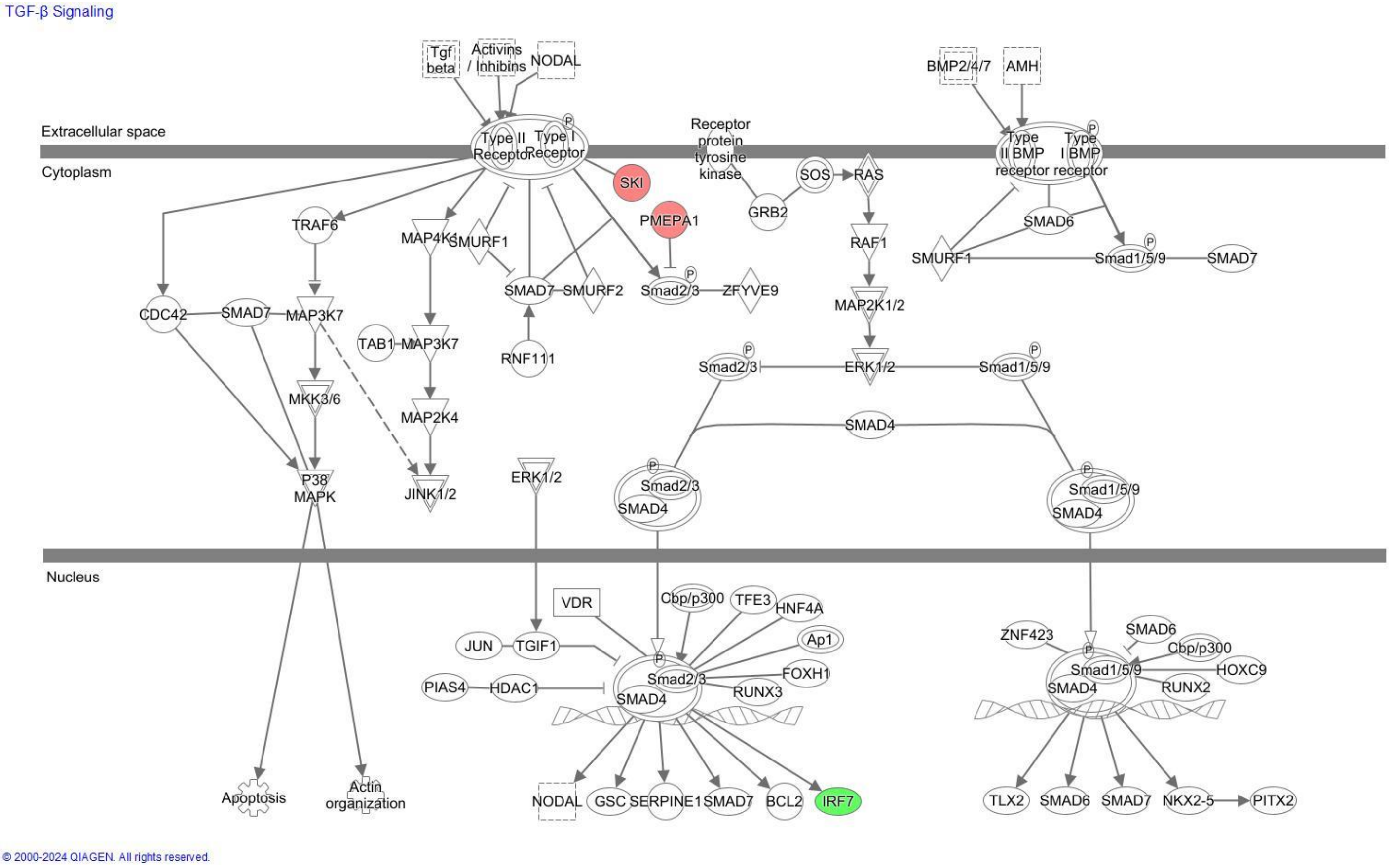

Figure S94

TGF-β Signaling

8 days
CAGE analysis

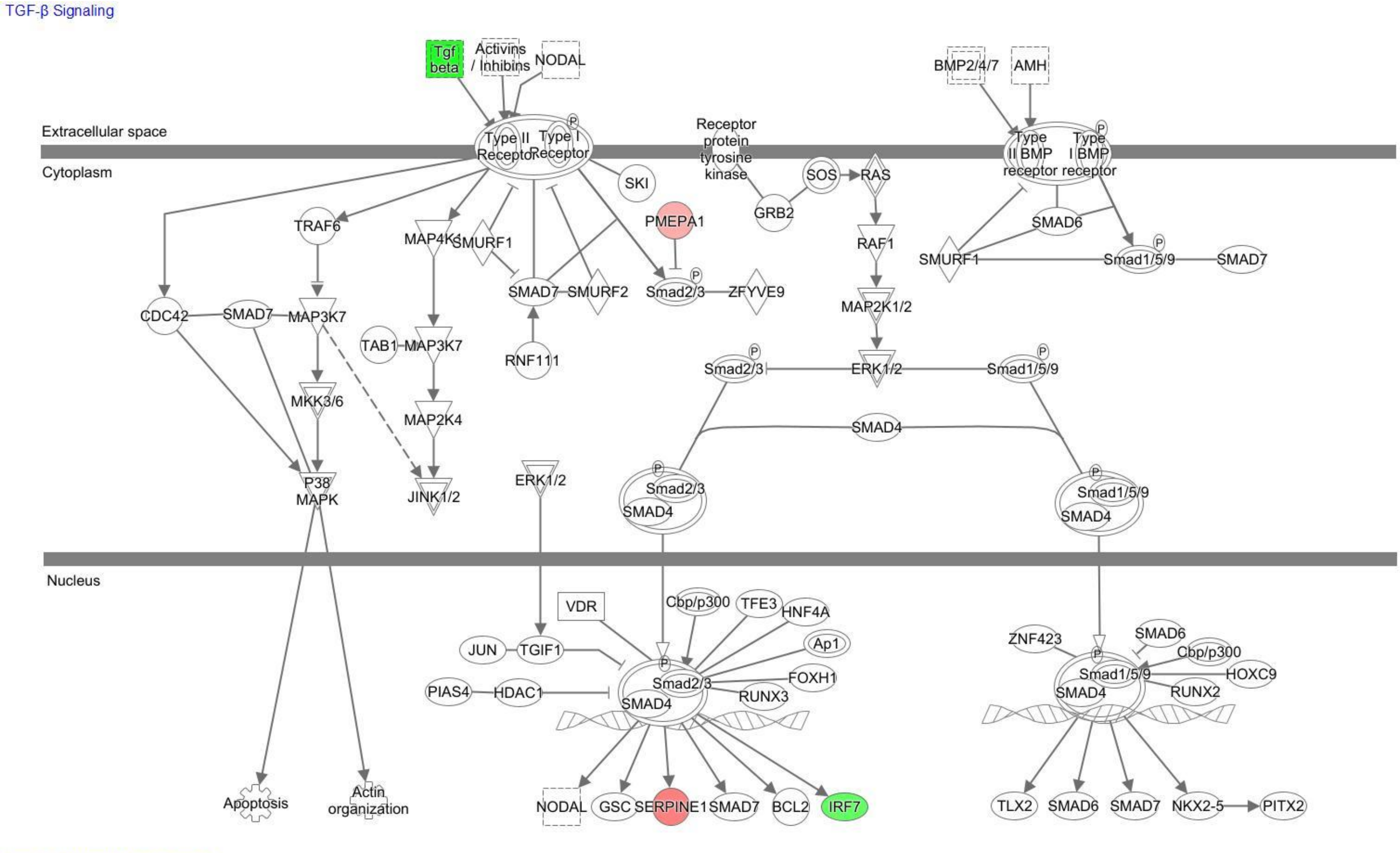

© 2000-2024 QIAGEN. All rights reserved.

Figure S95

TGF-β Signaling

Day 21
CAGE analysis

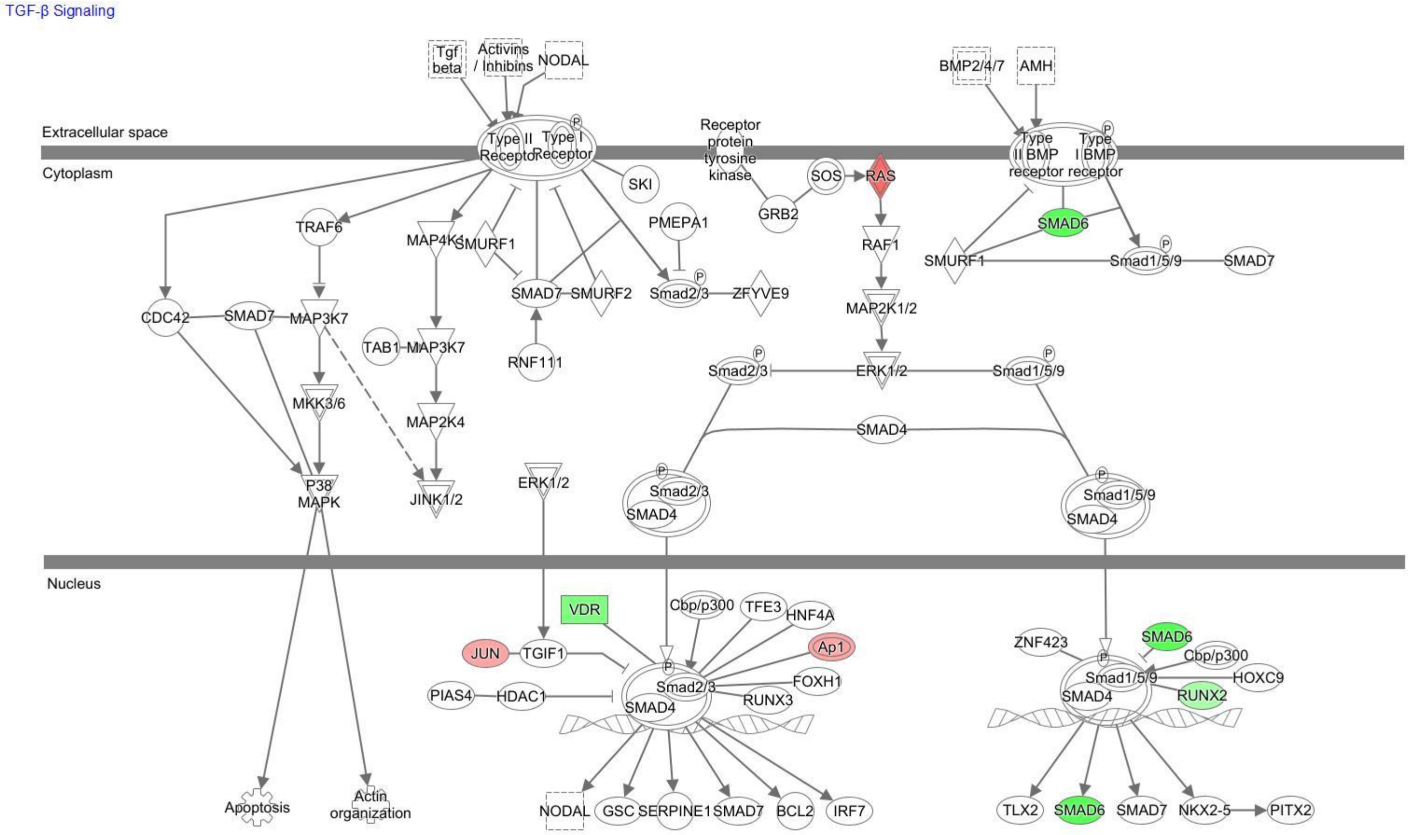

© 2000-2024 QIAGEN. All rights reserved.

Figure S96

VEGF Signaling

6 hours
CAGE analysis

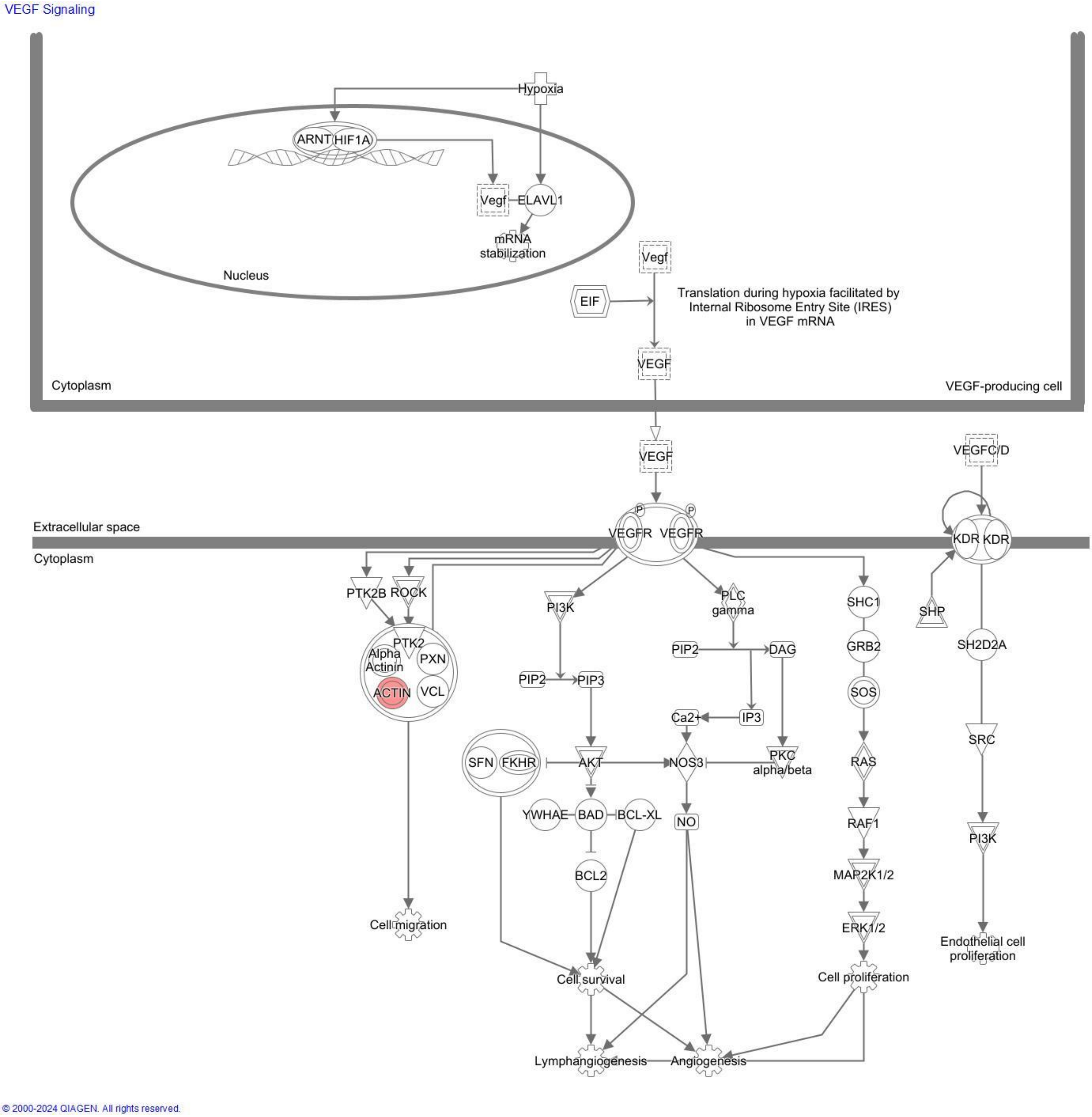

© 2000-2024 QIAGEN. All rights reserved.

Figure S97

VEGF Signaling

8 days
CAGE analysis

VEGF Signaling

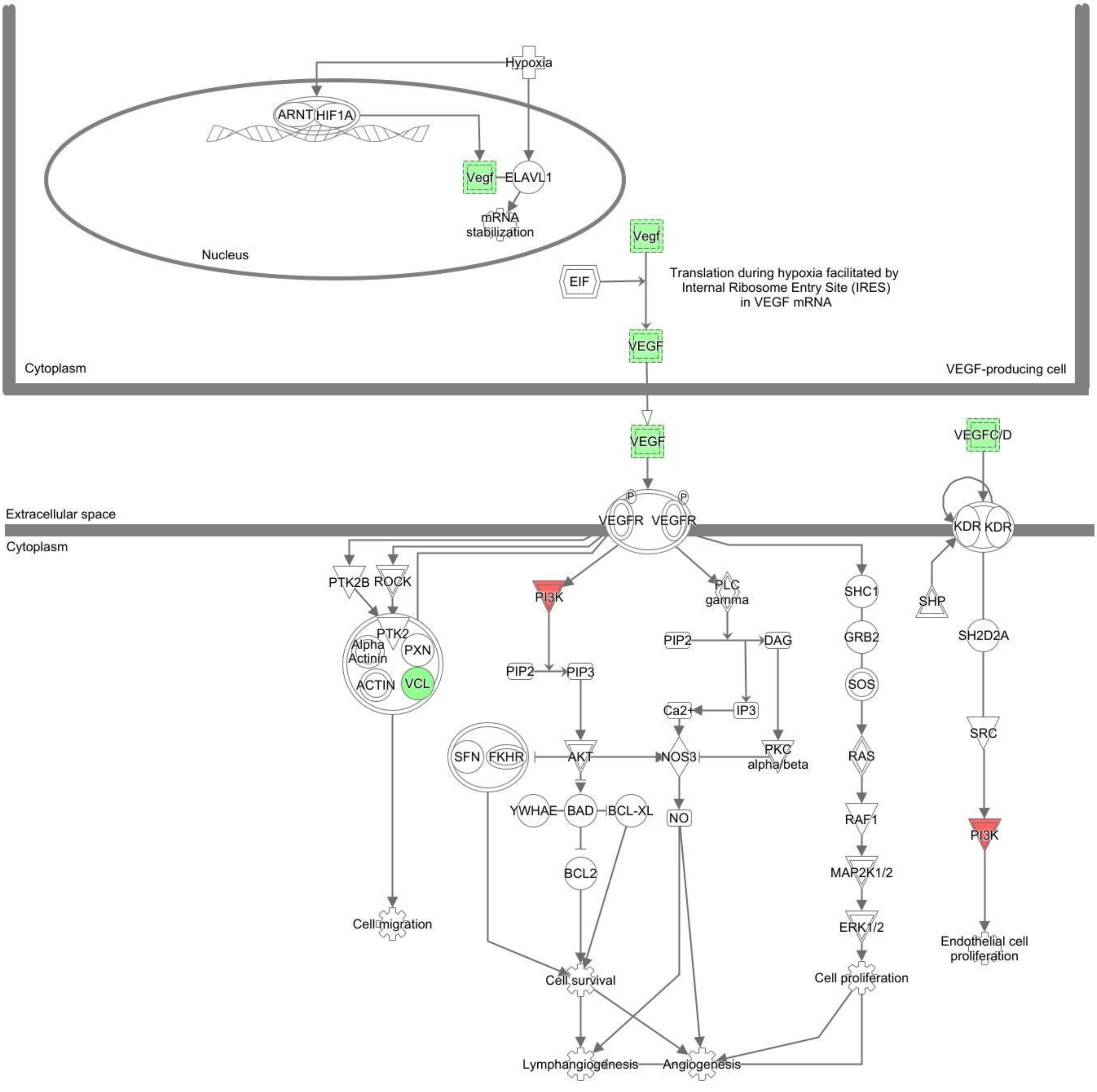

© 2000-2024 QIAGEN. All rights reserved.

Figure S98

VEGF Signaling

Day 21
CAGE analysis

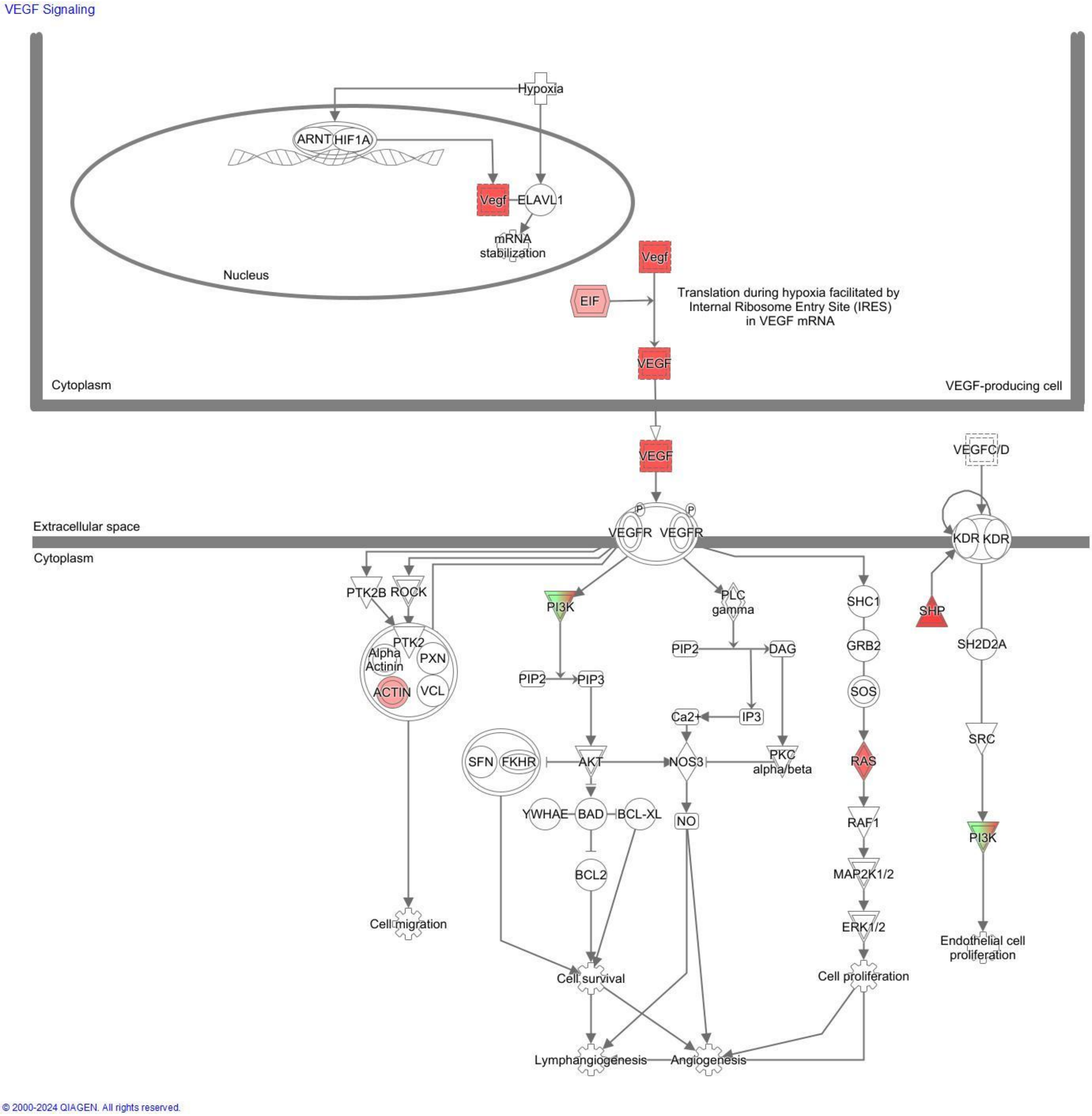

Figure S99

mTOR Signaling

1 hour
CAGE analysis

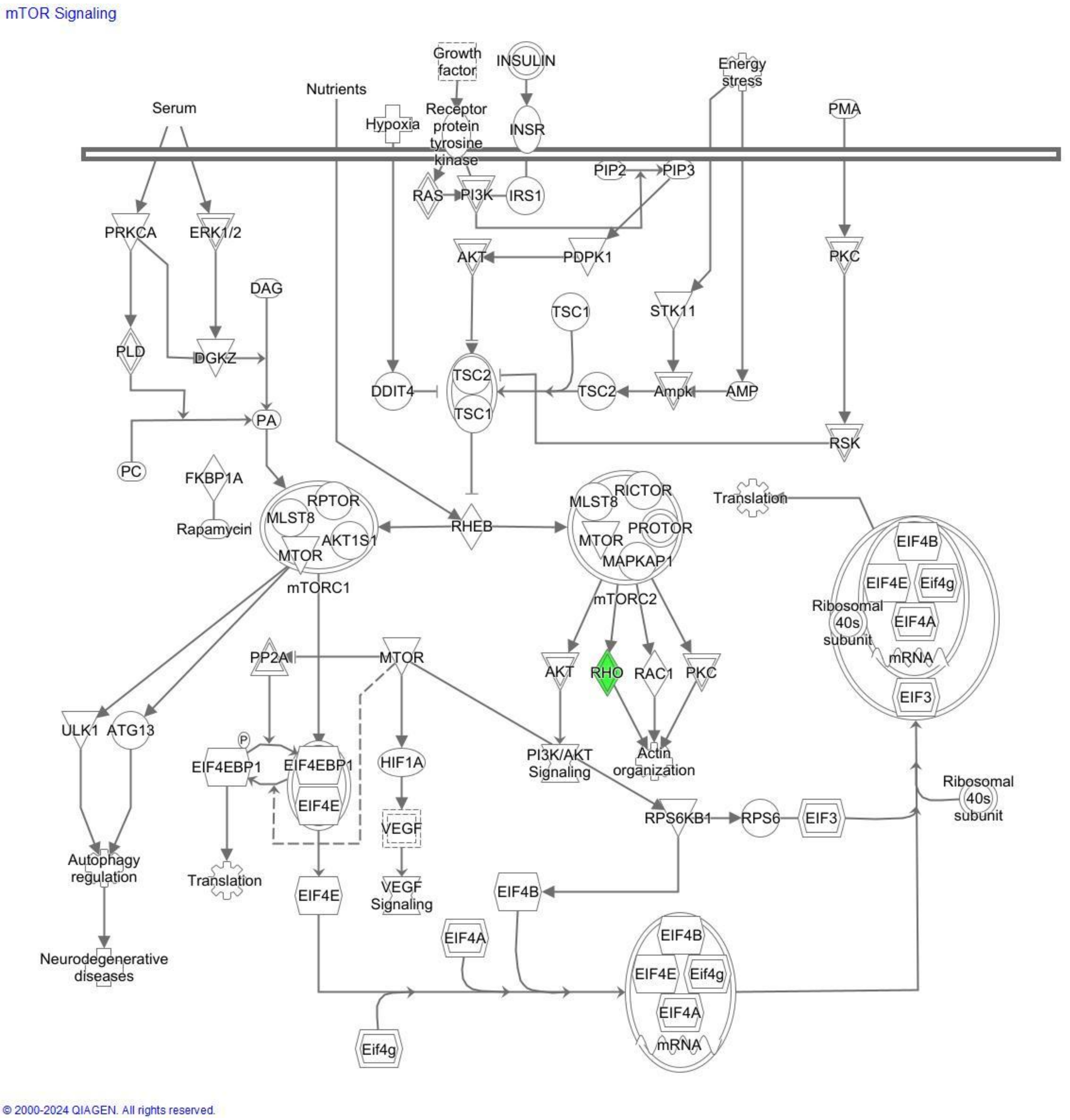

© 2000-2024 QIAGEN. All rights reserved.

Figure S100

mTOR Signaling

8 days
CAGE analysis

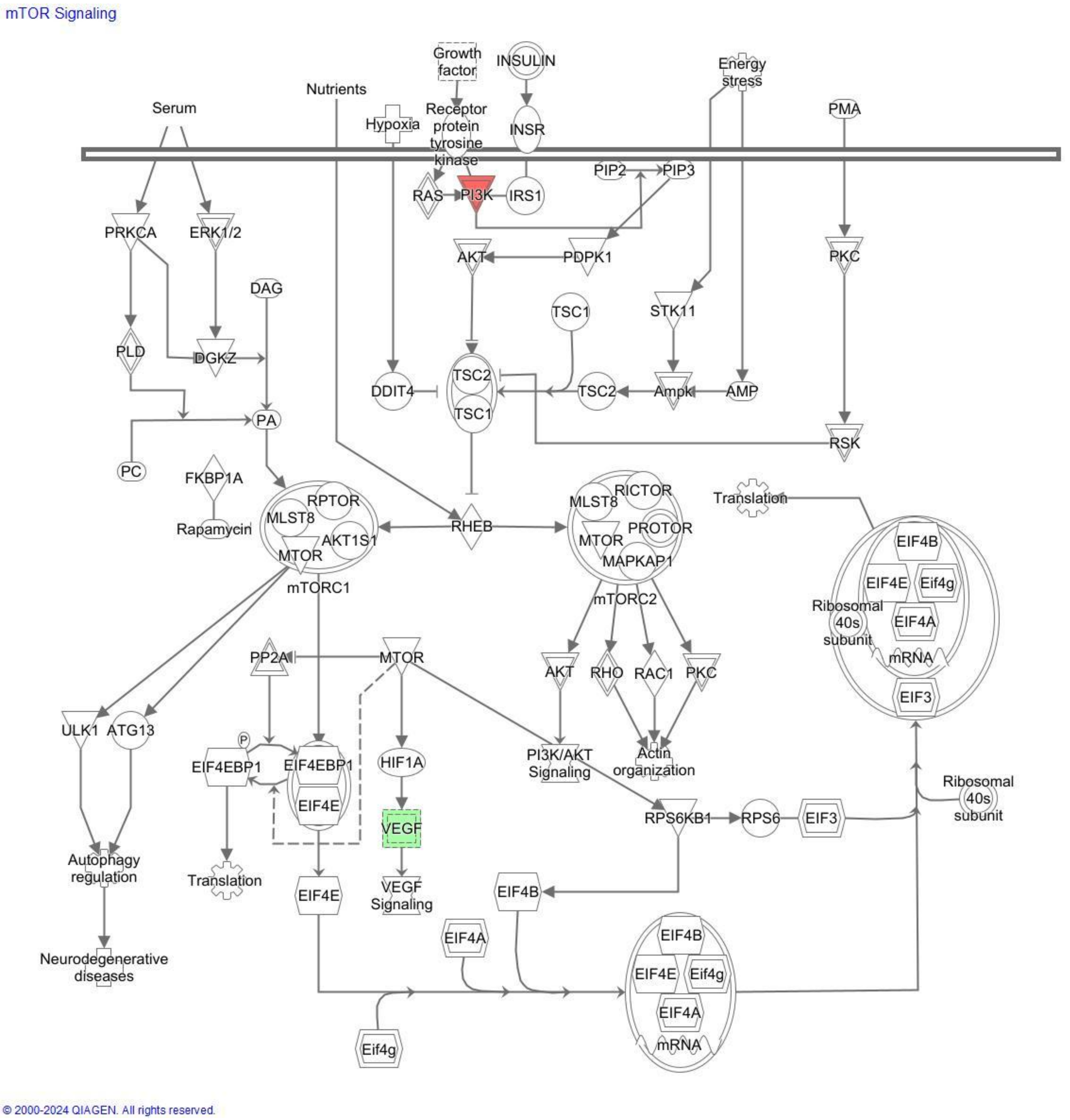

© 2000-2024 QIAGEN. All rights reserved.

Figure S101

mTOR Signaling

Day 21
CAGE analysis

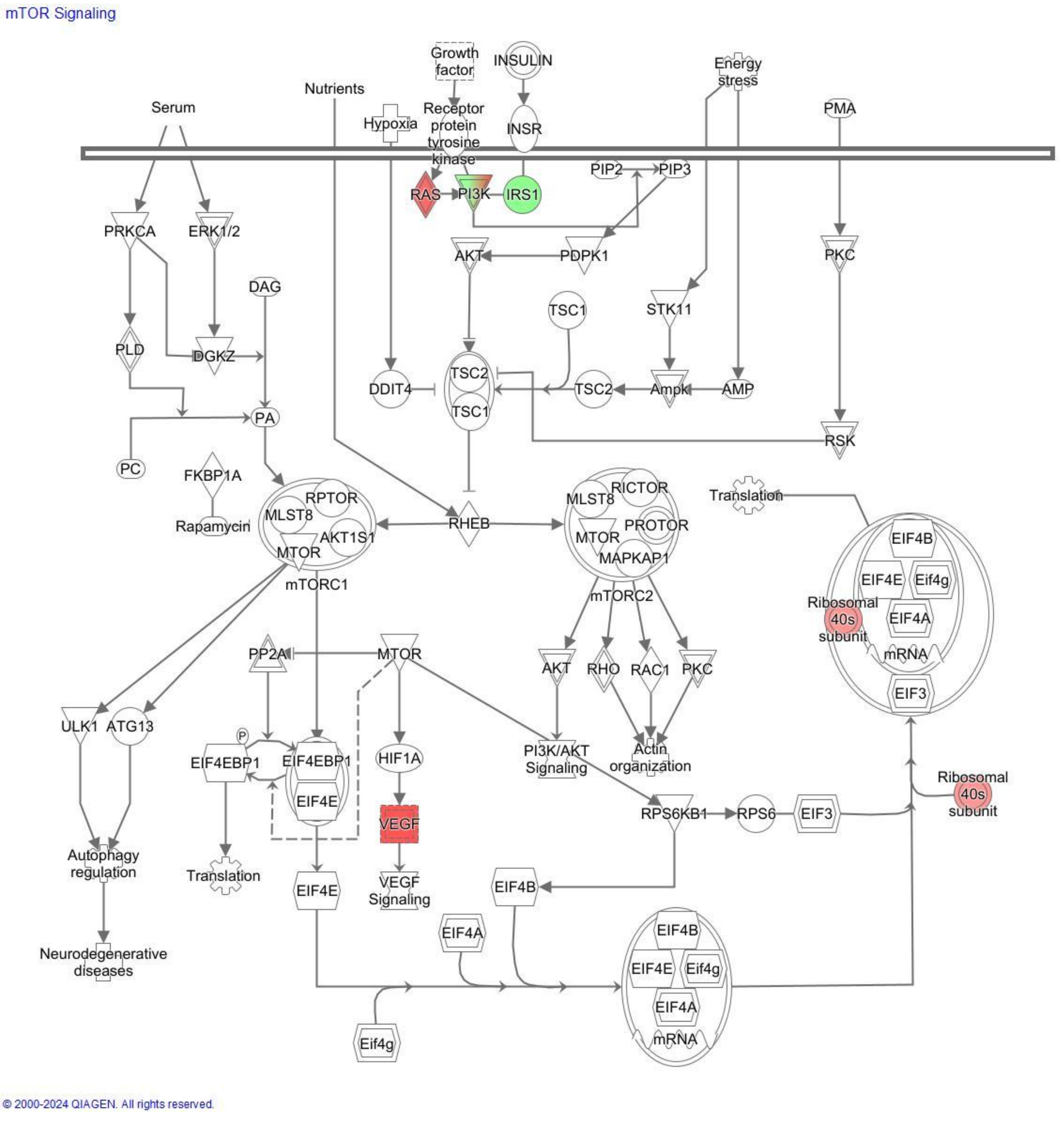

© 2000-2024 QIAGEN. All rights reserved.

Figure S102

PI3K-AKT Signaling

1 hour
CAGE analysis

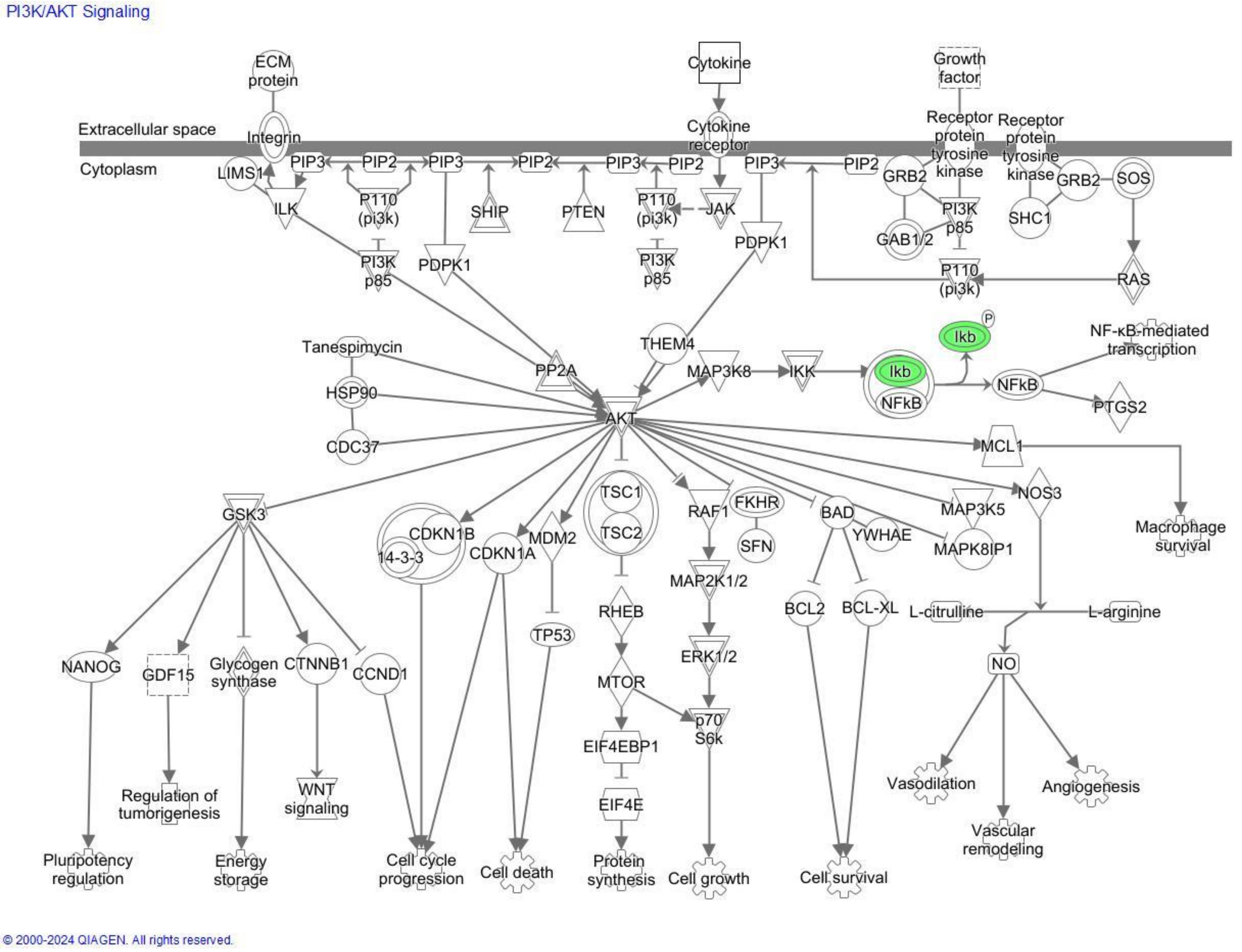

© 2000-2024 QIAGEN. All rights reserved.

Figure S103

PI3K-AKT Signaling

6 hours
CAGE analysis

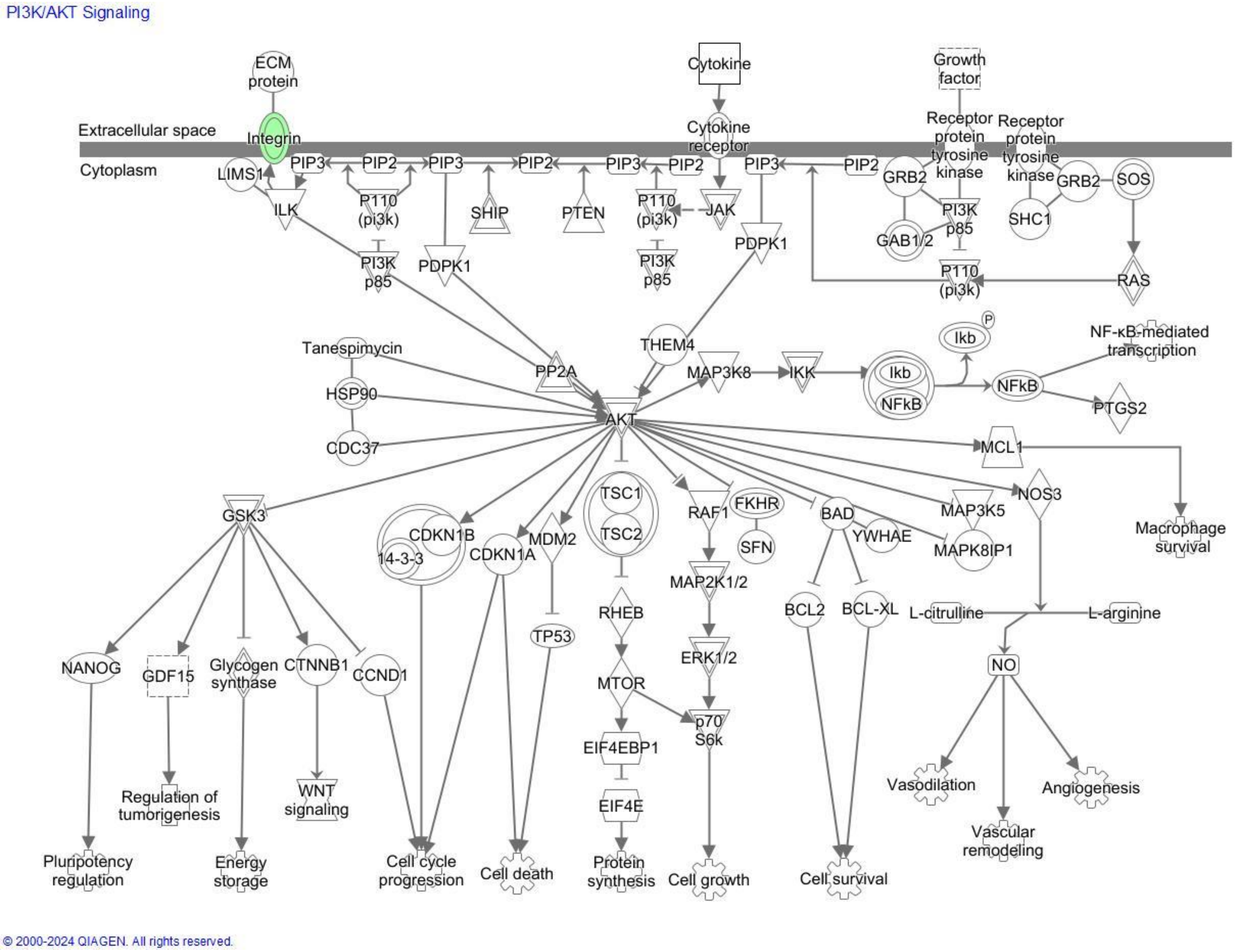

© 2000-2024 QIAGEN. All rights reserved.

Figure S104

PI3K-AKT Signaling

24 hours
CAGE analysis

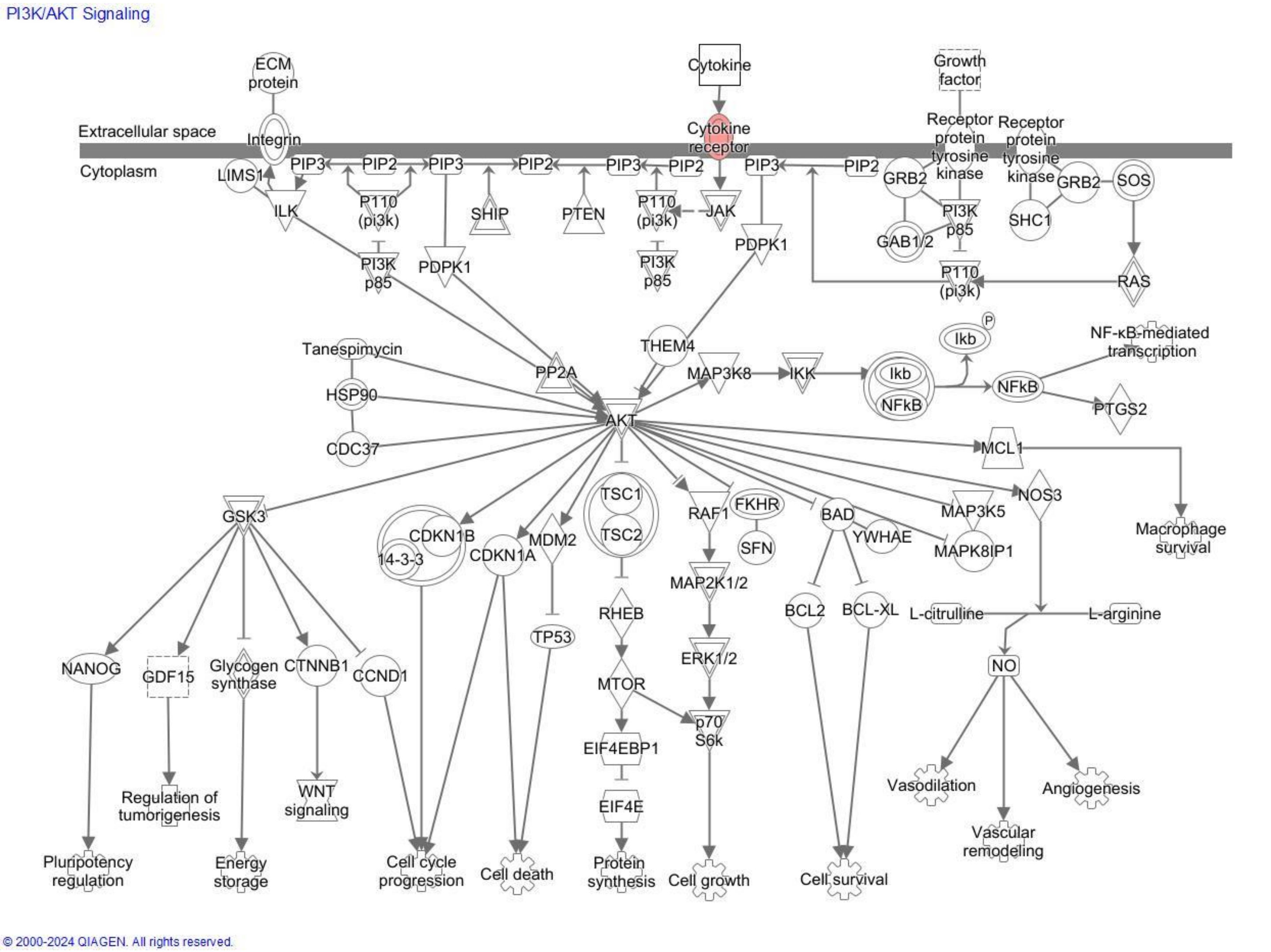

© 2000-2024 QIAGEN. All rights reserved.

Figure S105

PI3K-AKT Signaling

8 days
CAGE analysis

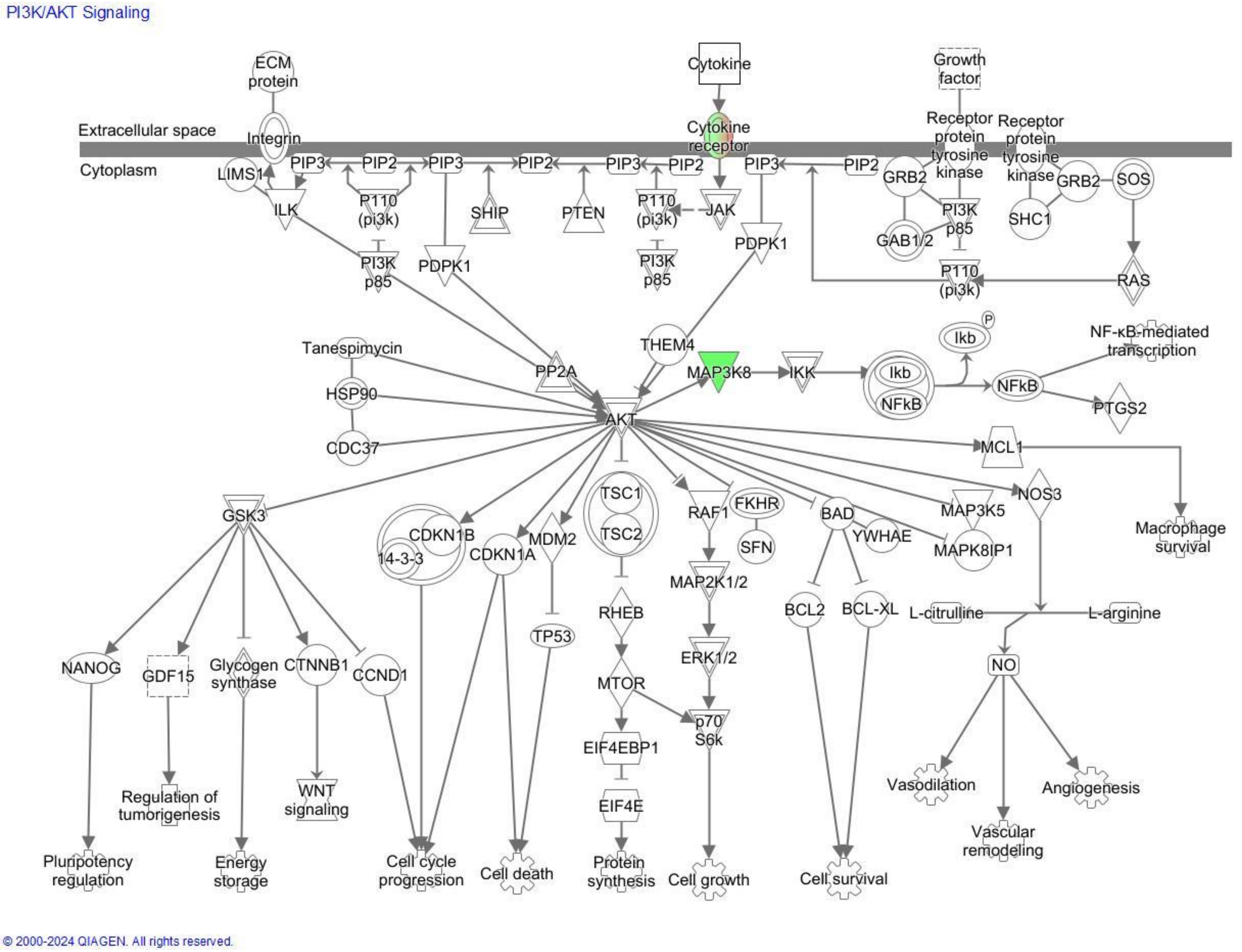

© 2000-2024 QIAGEN. All rights reserved.

Figure S106

PI3K-AKT Signaling

day 21
CAGE analysis

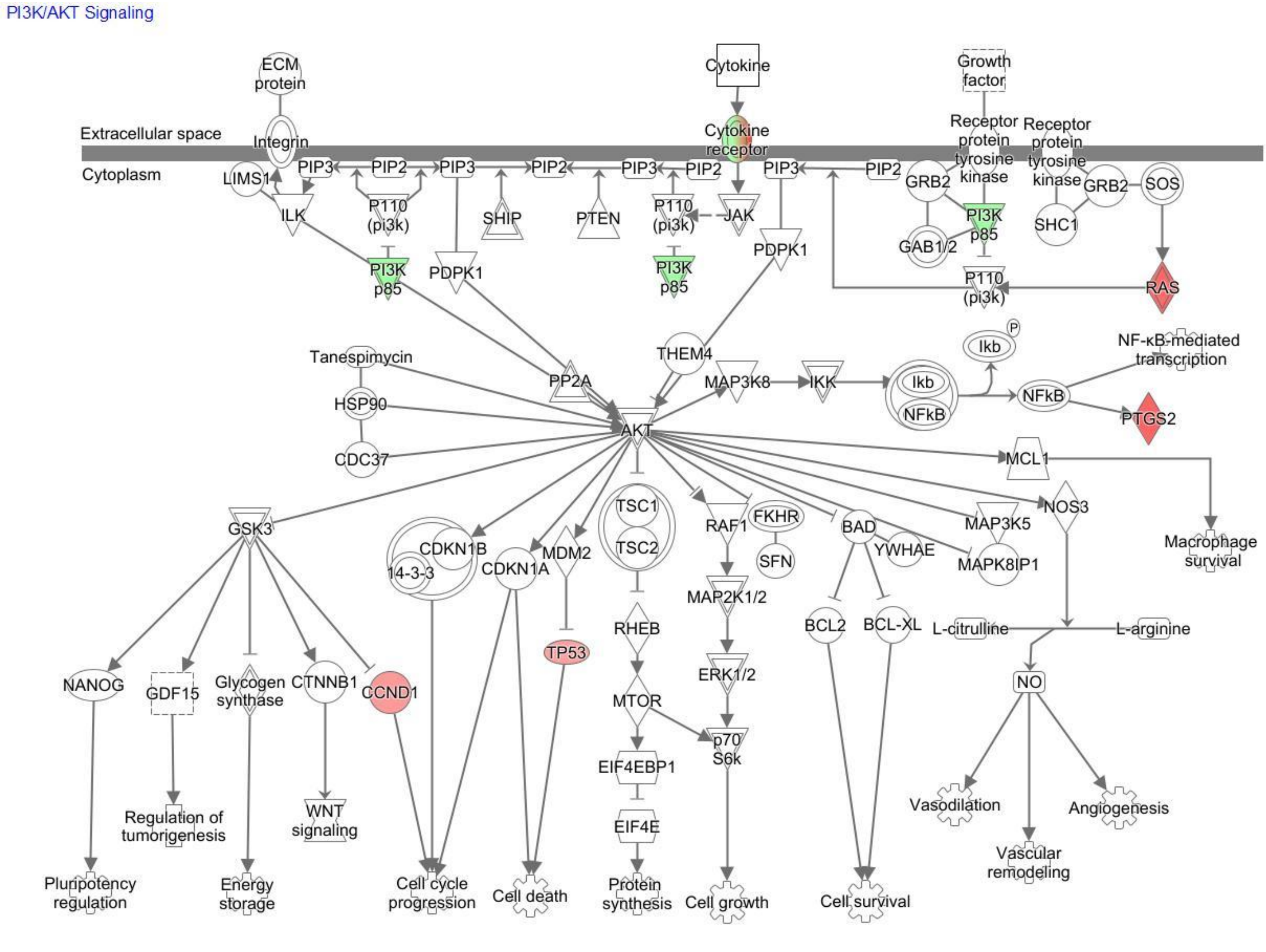

© 2000-2024 QIAGEN. All rights reserved.

Figure S107

WNT/β catenin signaling

8 days
CAGE analysis

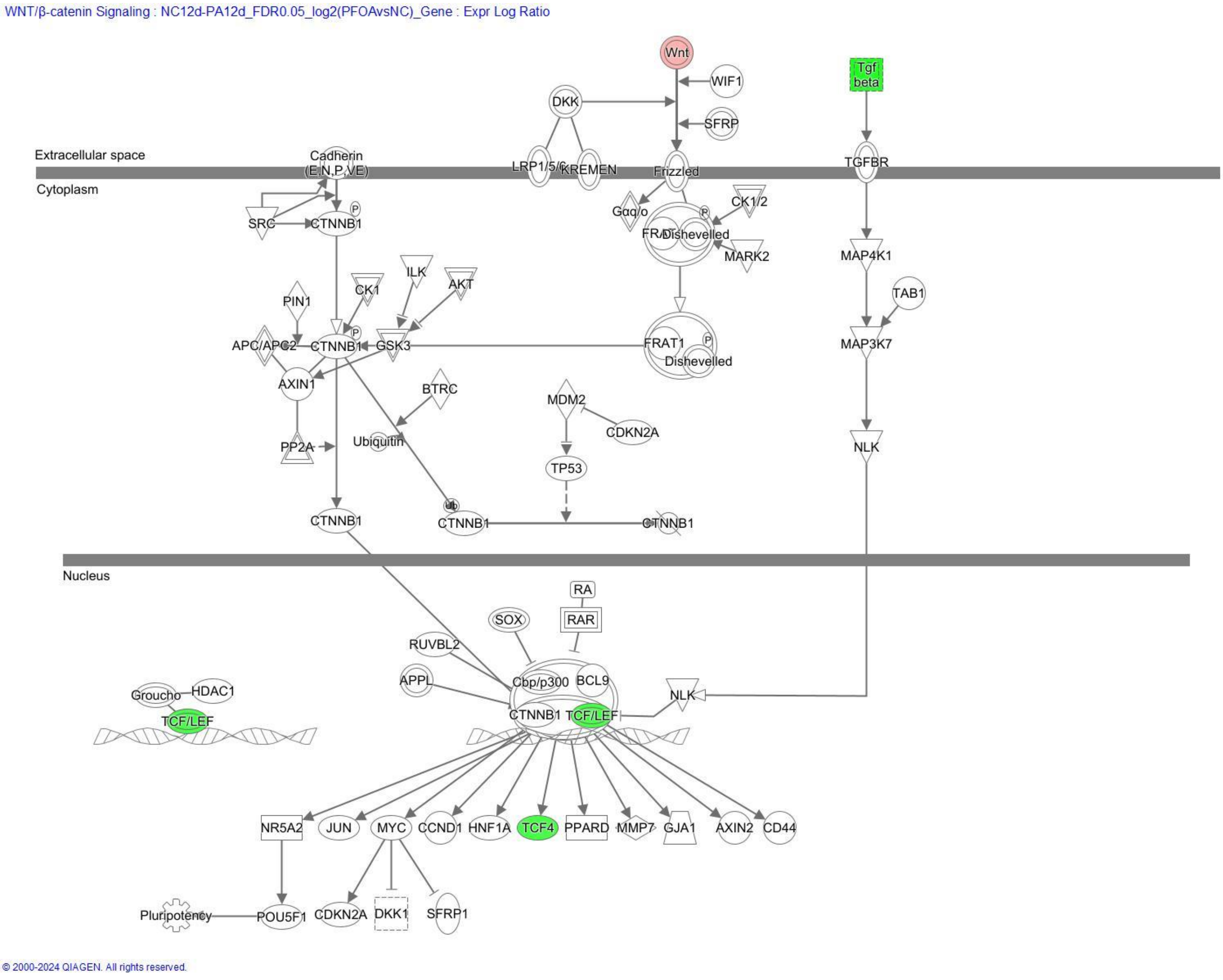

© 2000-2024 QIAGEN. All rights reserved.

Figure S108

WNT/β catenin signaling

Day 21
CAGE analysis

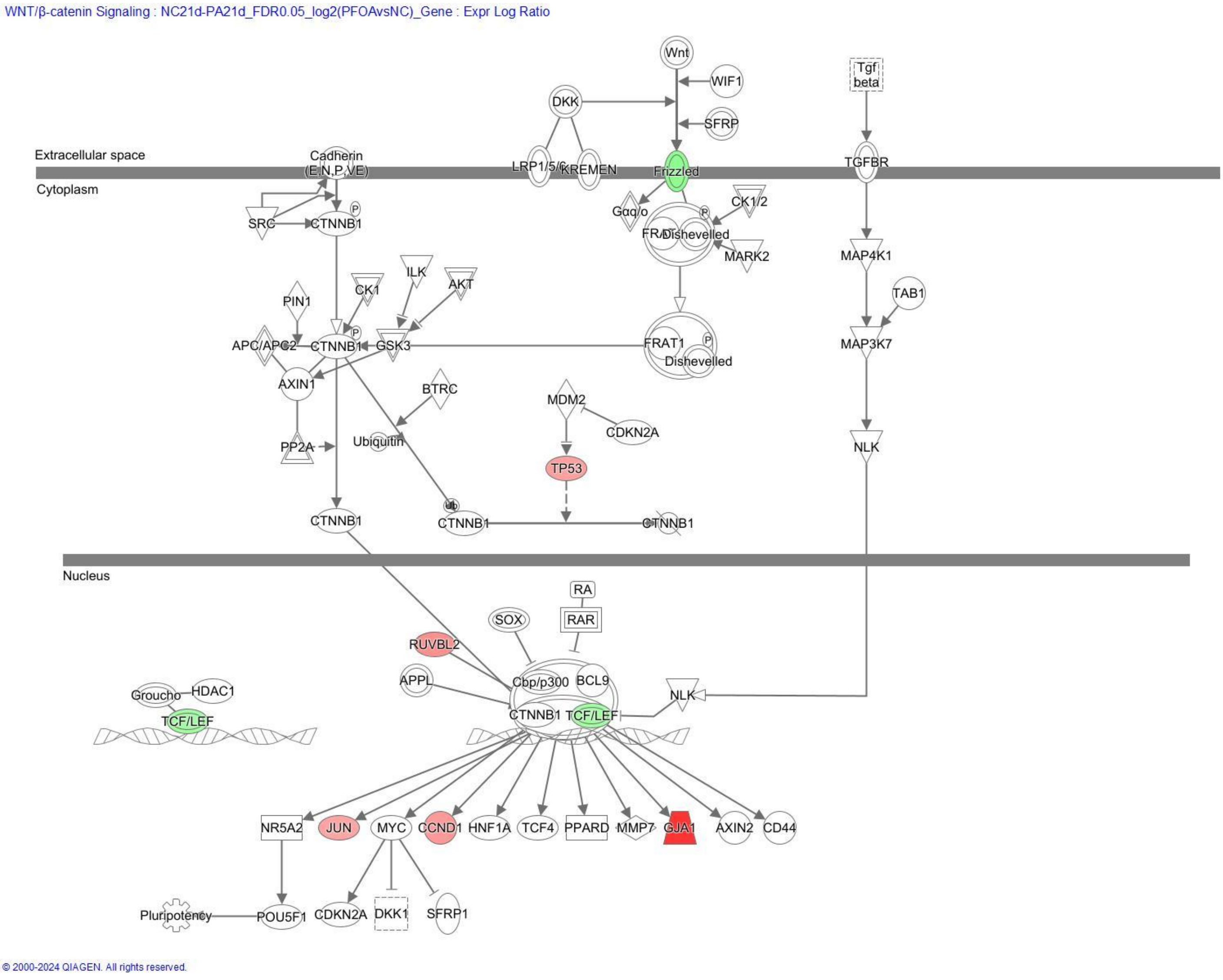

© 2000-2024 QIAGEN. All rights reserved.

Figure S109

ATM Signaling

1 hour
CAGE analysis

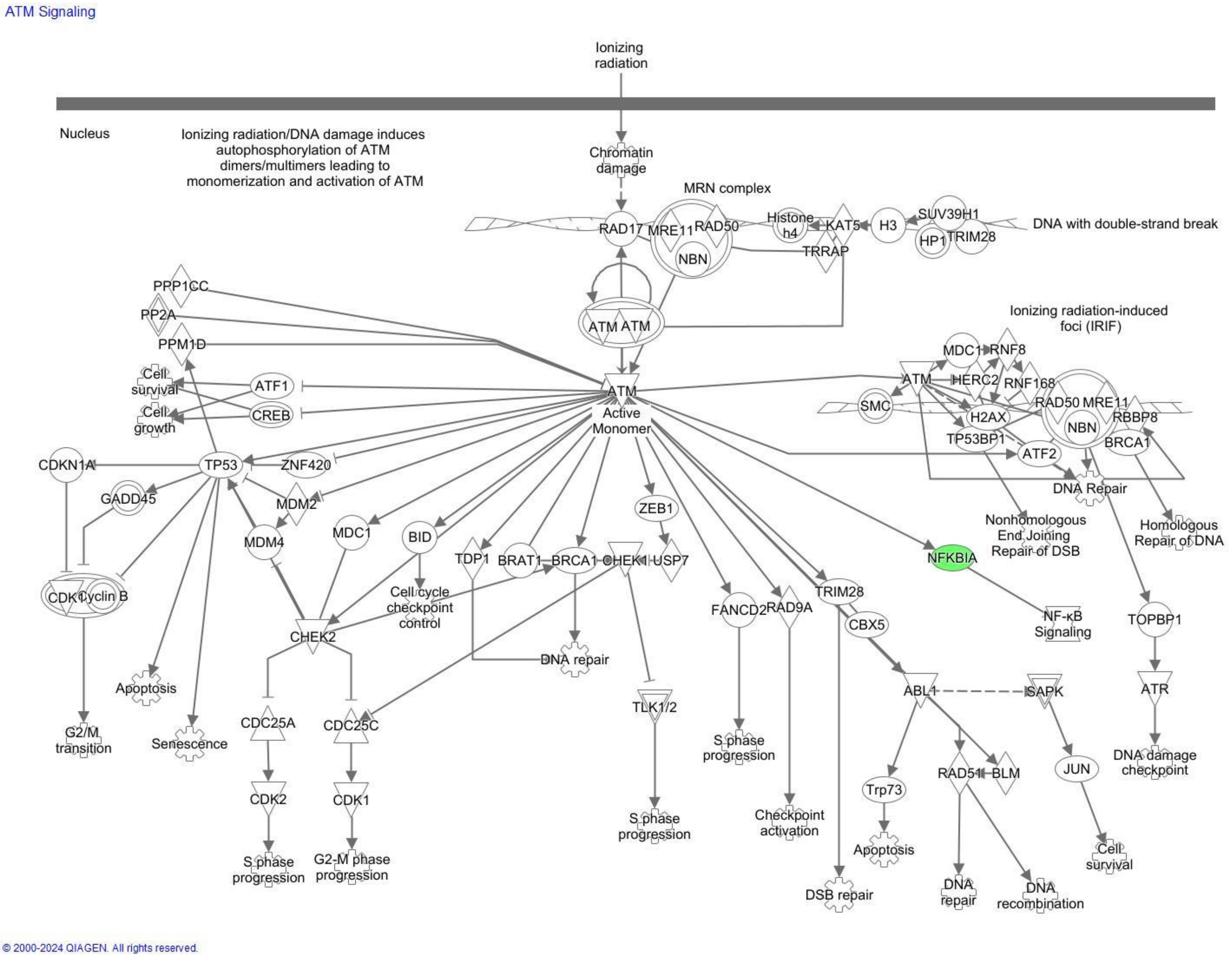

© 2000-2024 QIAGEN. All rights reserved.

Figure S110

ATM Signaling

8 days
CAGE analysis

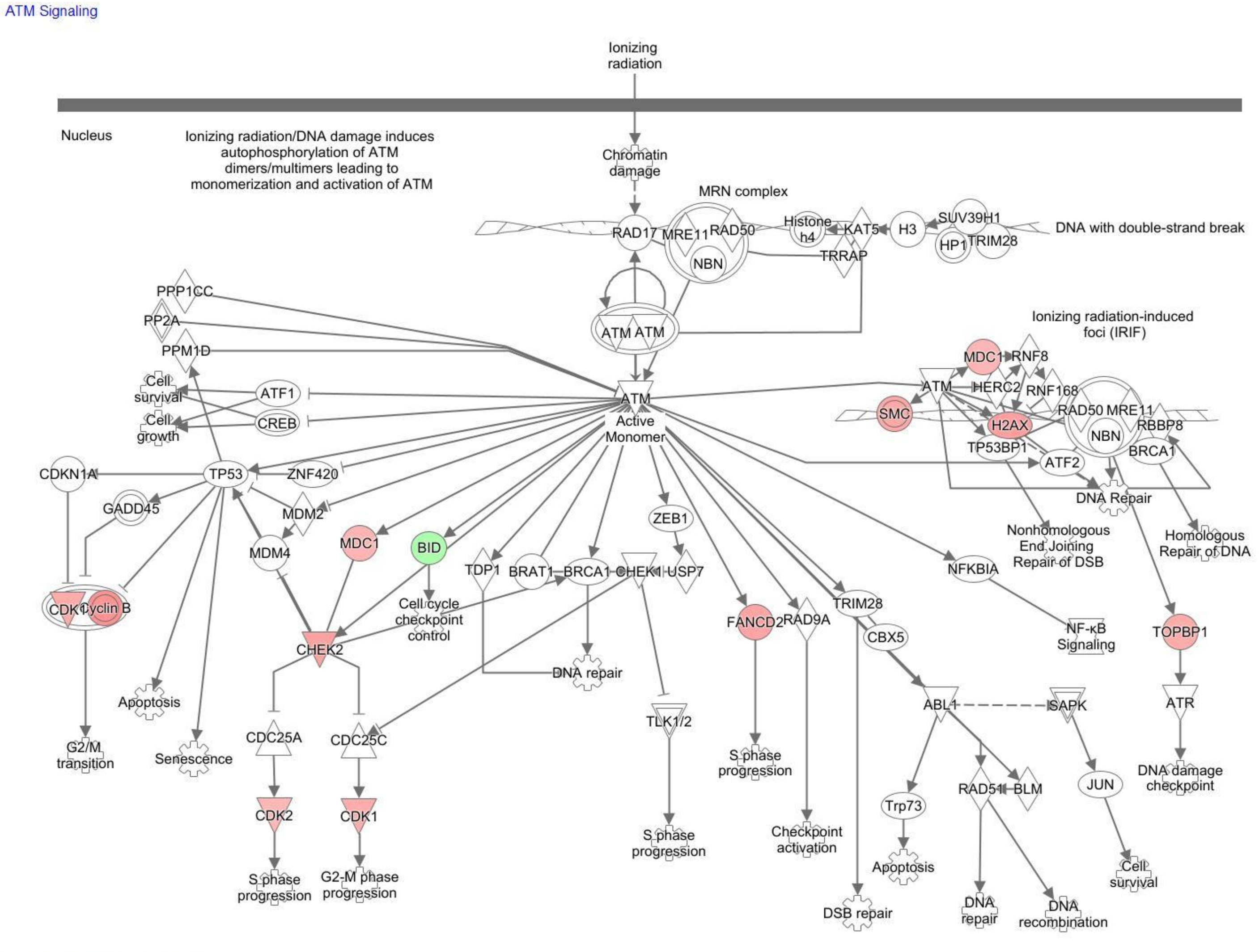

© 2000-2024 QIAGEN. All rights reserved.

Figure S111

ATM Signaling

Day 21
CAGE analysis

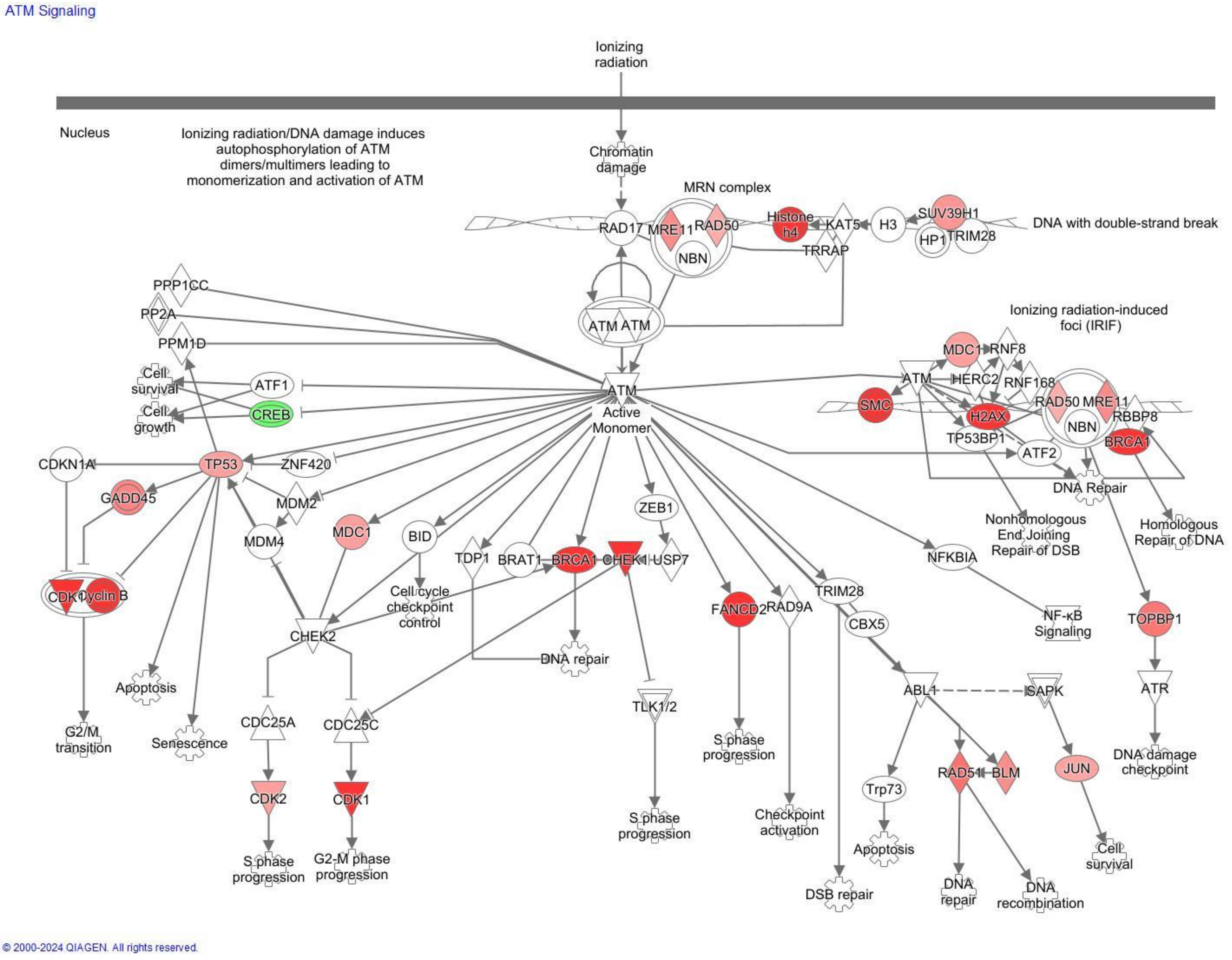

Figure S112

BRCA1 in DNA Damage Response

24 hours
CAGE analysis

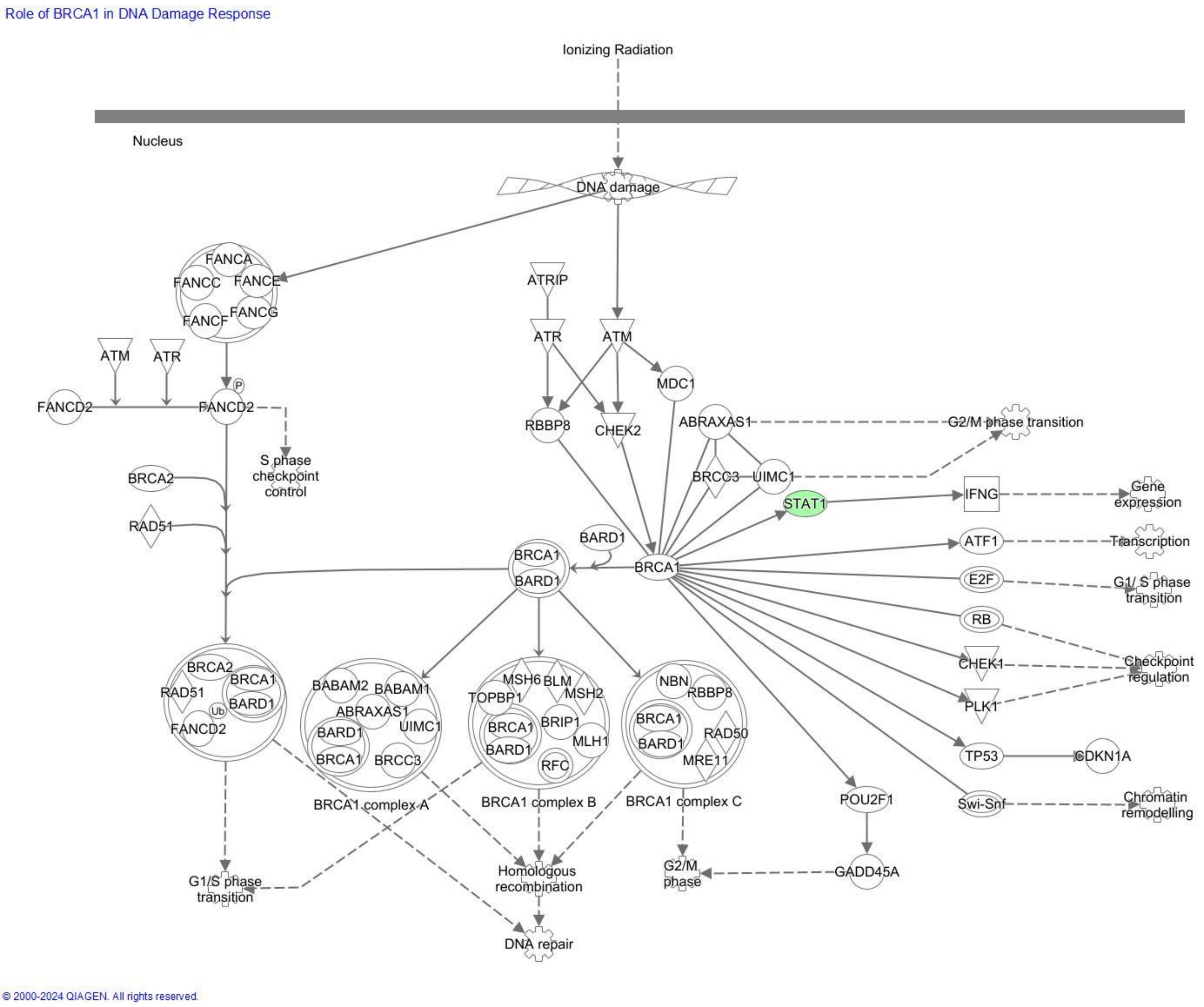

Figure S113

BRCA1 in DNA Damage Response

8 days
CAGE analysis

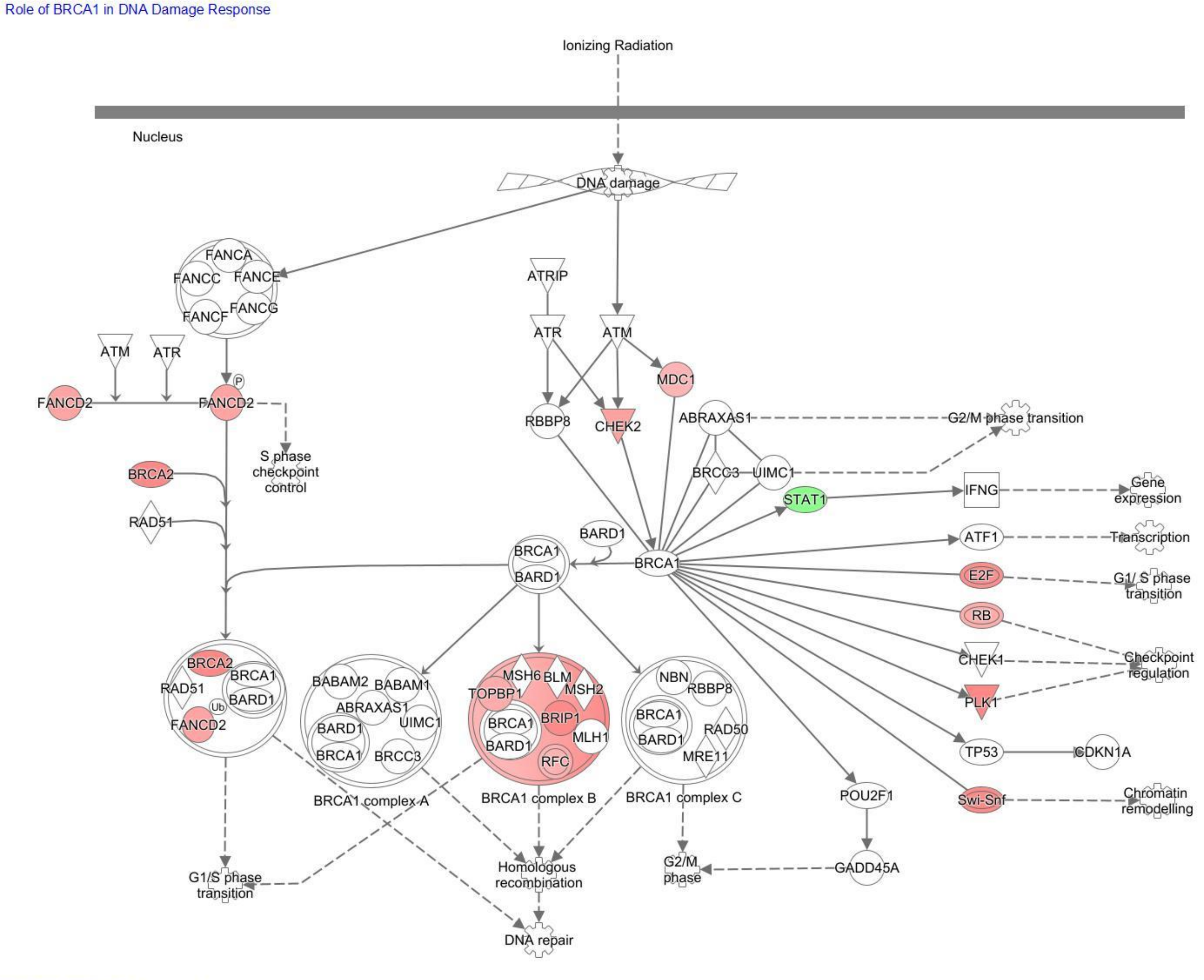

© 2000-2024 QIAGEN. All rights reserved.

Figure S114

BRCA1 in DNA Damage Response

Day 21
CAGE analysis

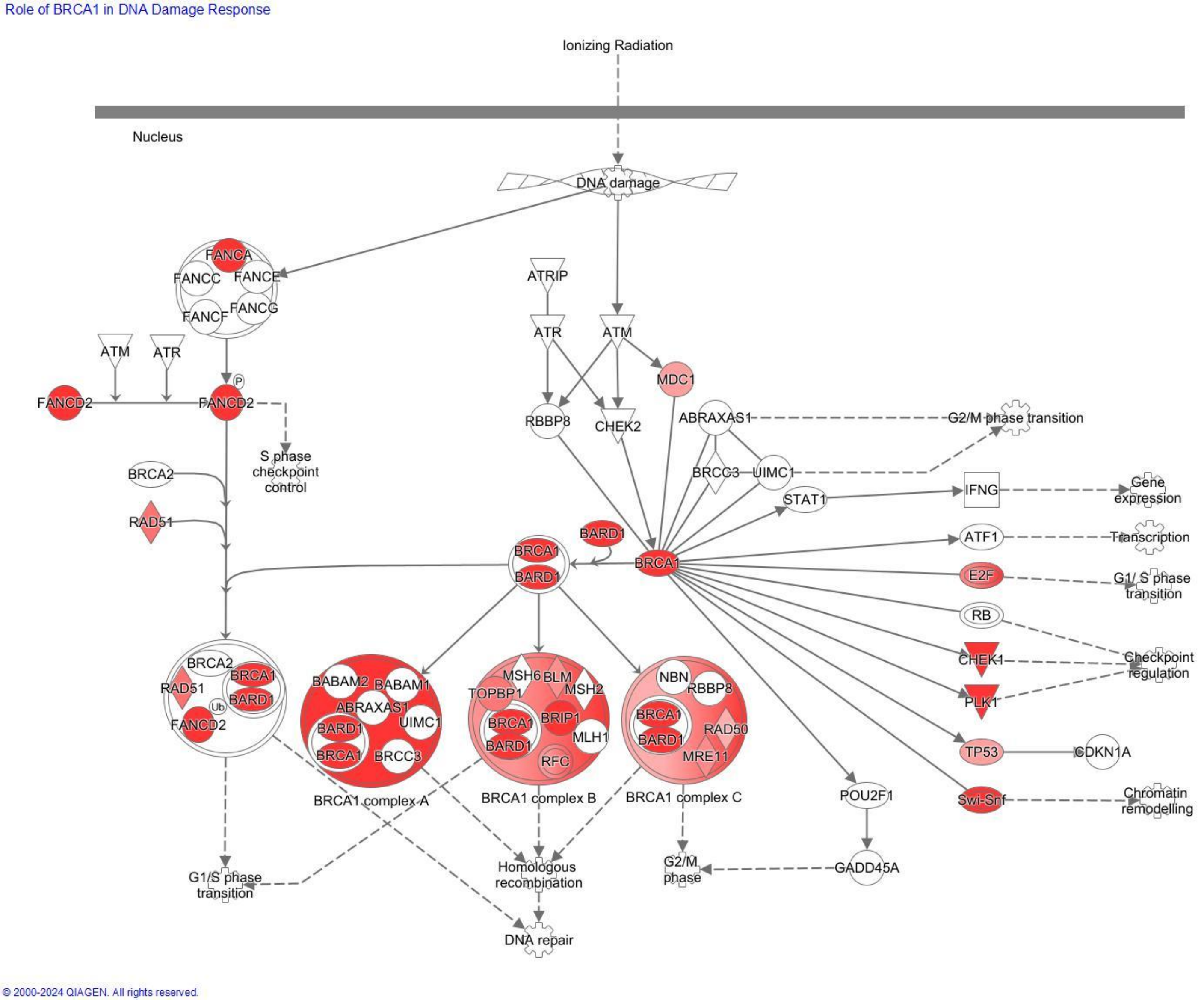

© 2000-2024 QIAGEN. All rights reserved.

Figure S115

Role of CHK Proteins in Cell Cycle Checkpoint Control

8 days
CAGE analysis

Role of CHK Proteins in Cell Cycle Checkpoint Control

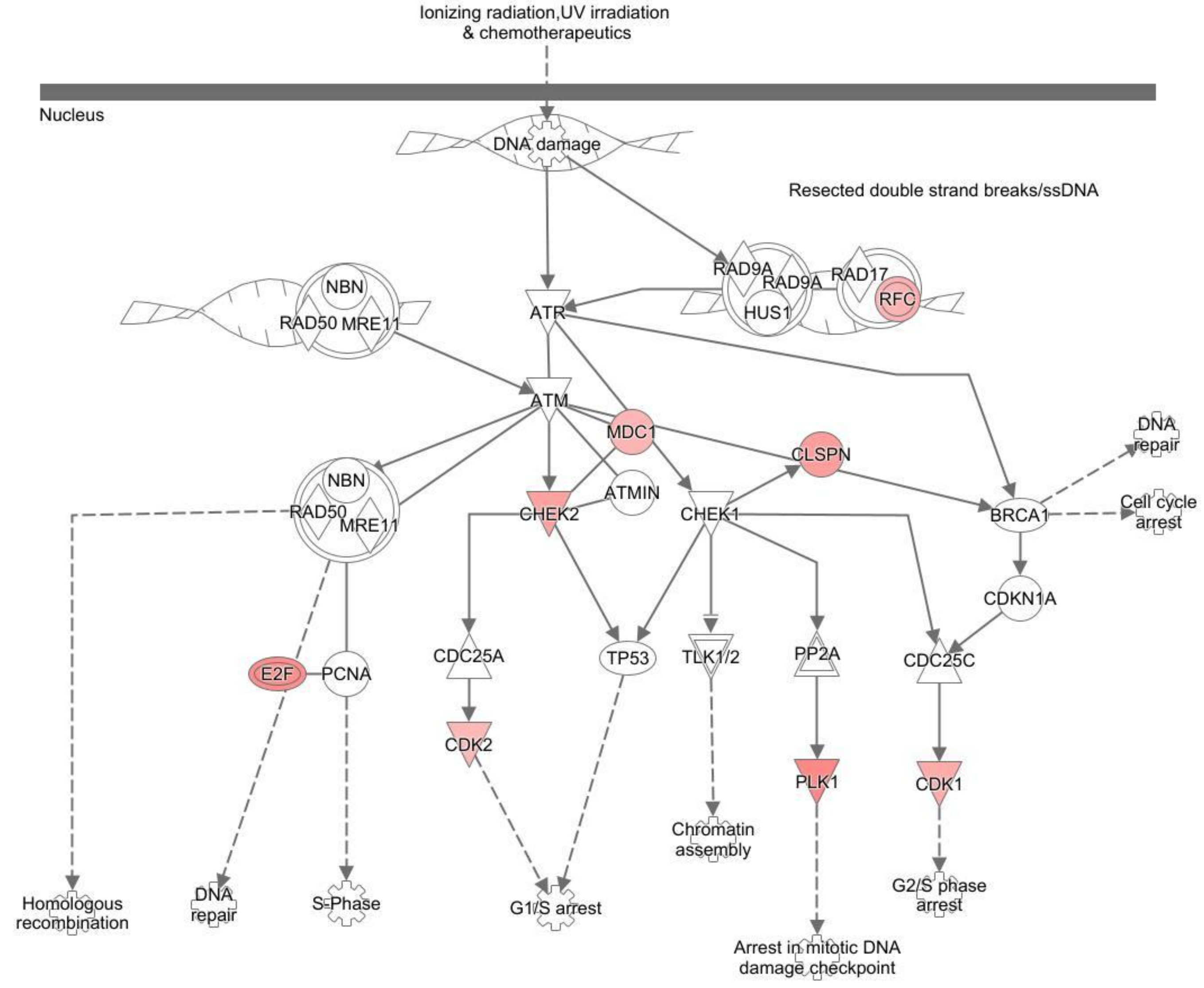

© 2000-2024 QIAGEN. All rights reserved.

Figure S116

Role of CHK Proteins in Cell Cycle Checkpoint Control

Day 21
CAGE analysis

Role of CHK Proteins in Cell Cycle Checkpoint Control

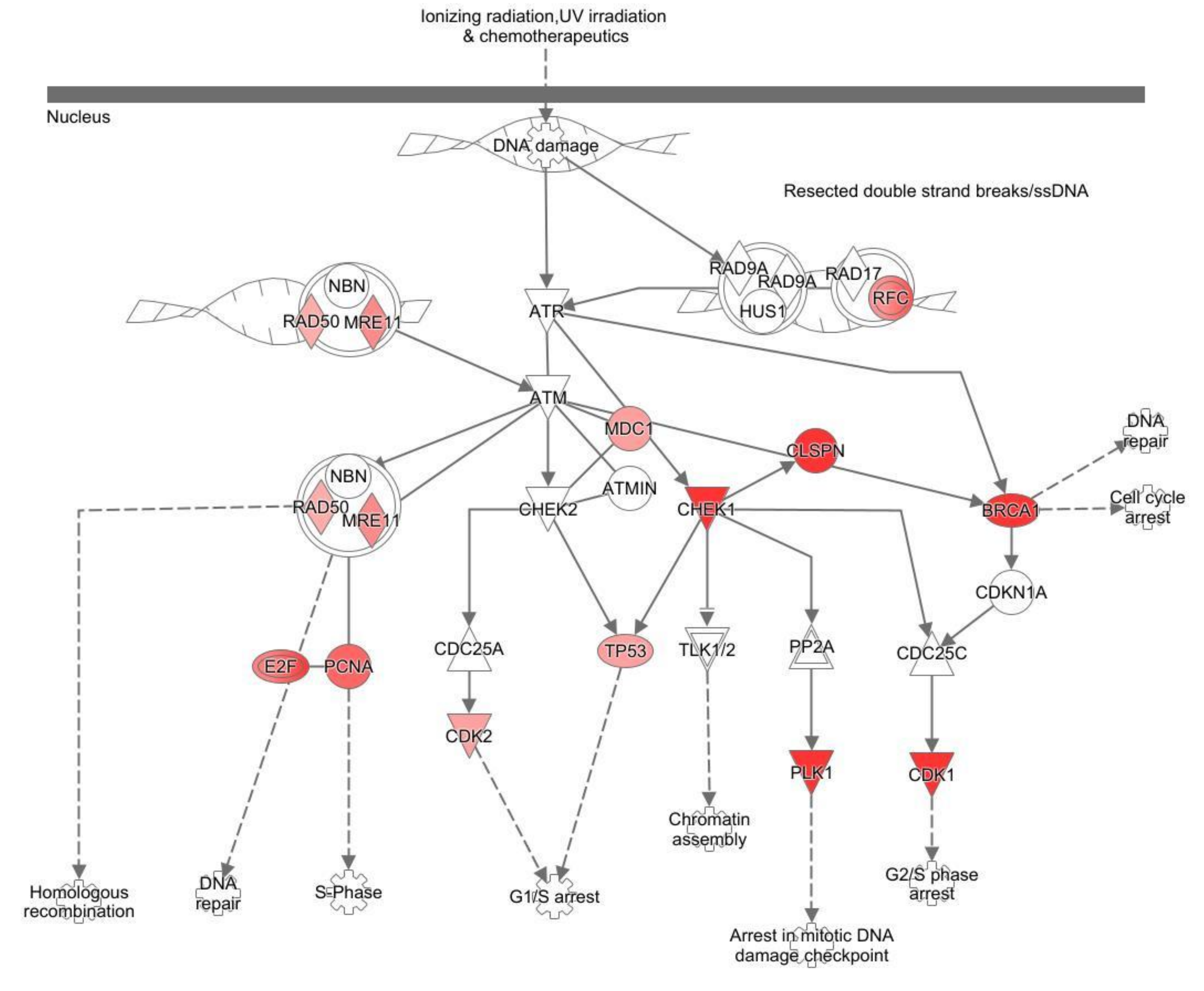

© 2000-2024 QIAGEN. All rights reserved.

Figure S117

Cell cycle: G2/M DNA Damage Checkpoint Regulation

8 days
CAGE analysis

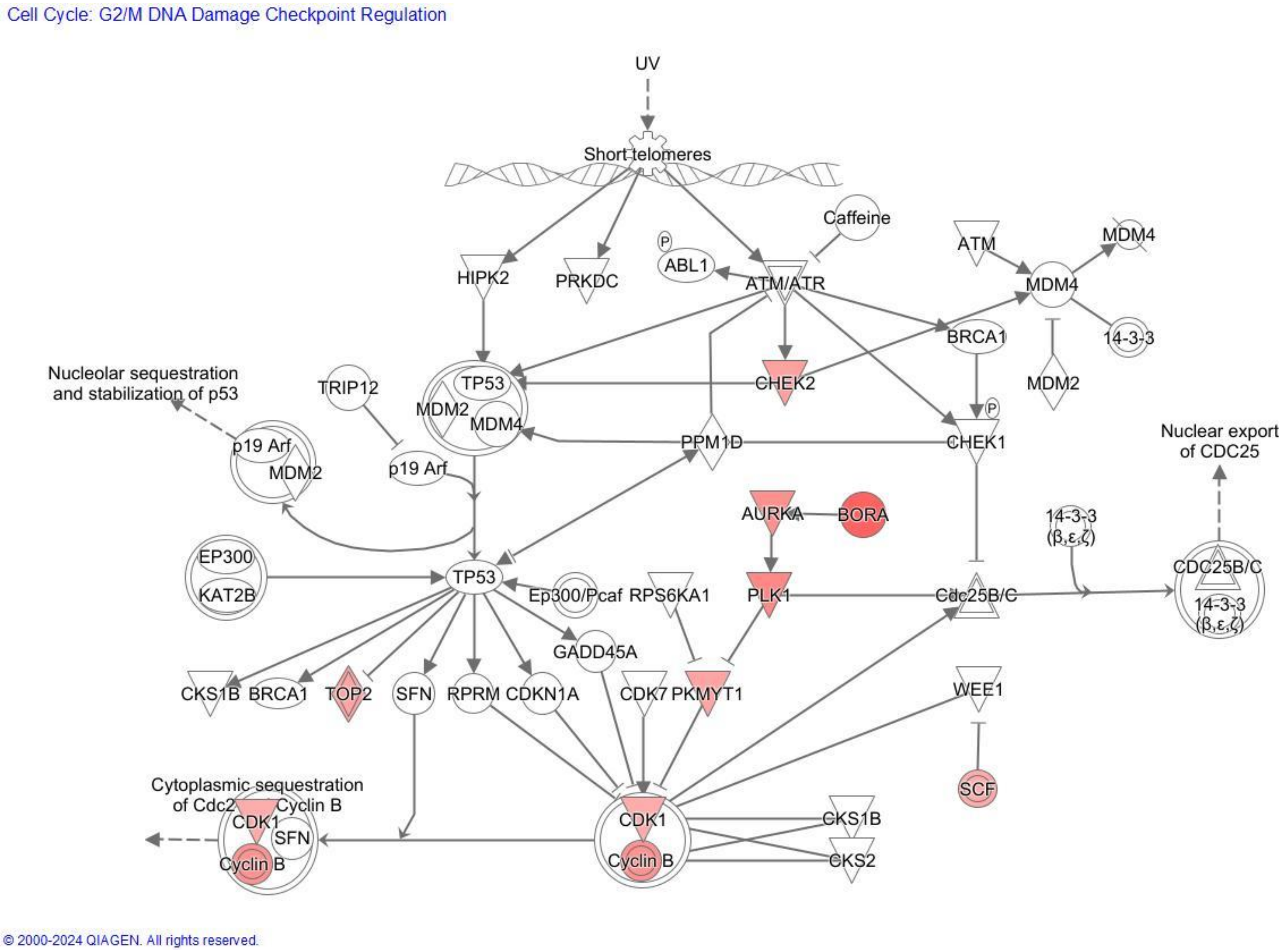

© 2000-2024 QIAGEN. All rights reserved.

Figure S118

Cell cycle: G2/M DNA Damage Checkpoint Regulation

Day 21
CAGE analysis

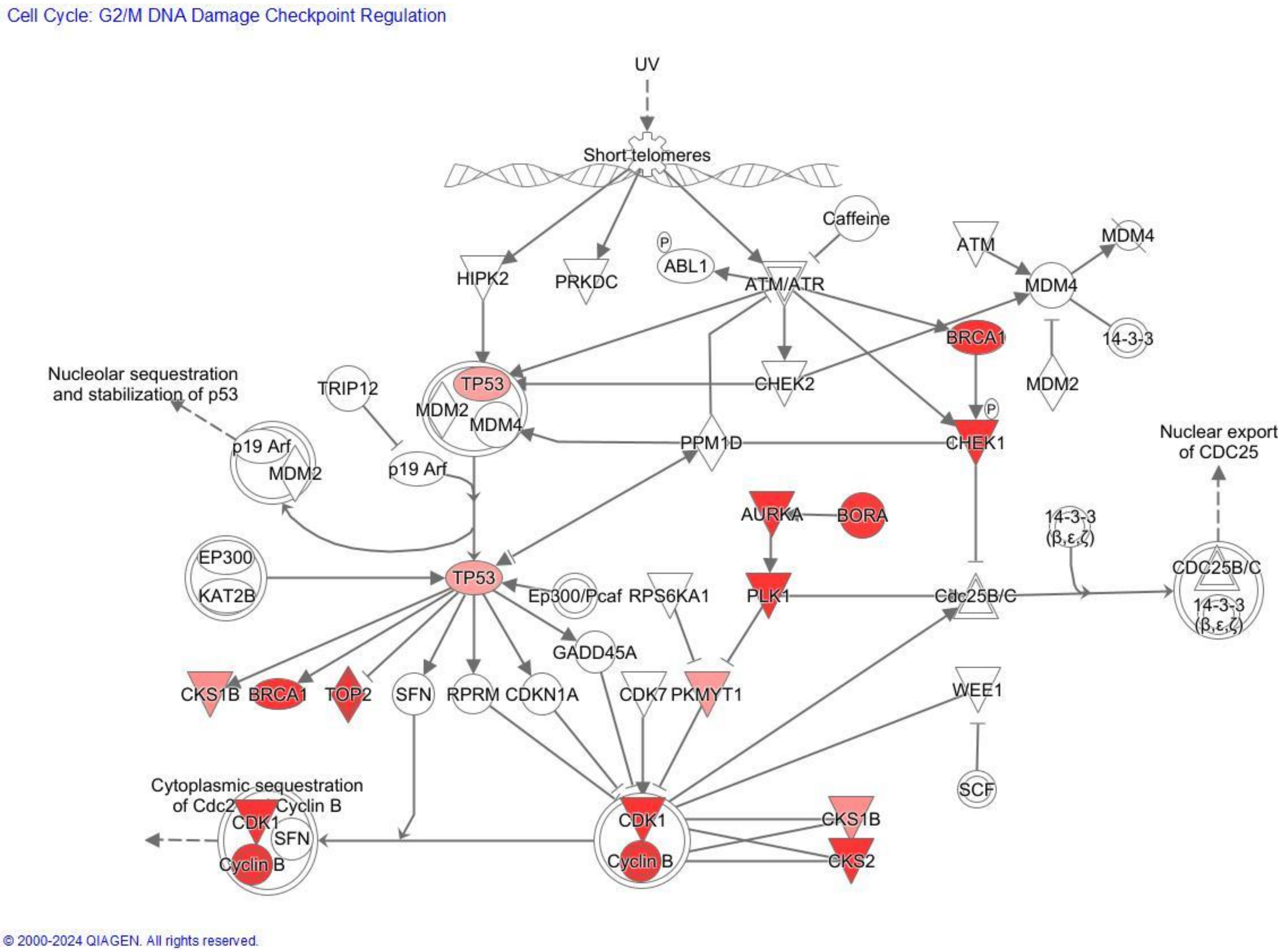

© 2000-2024 QIAGEN. All rights reserved.

Figure S119

DNA Methylation and Transcriptional Repression Signaling

8 days
CAGE analysis

DNA Methylation and Transcriptional Repression Signaling

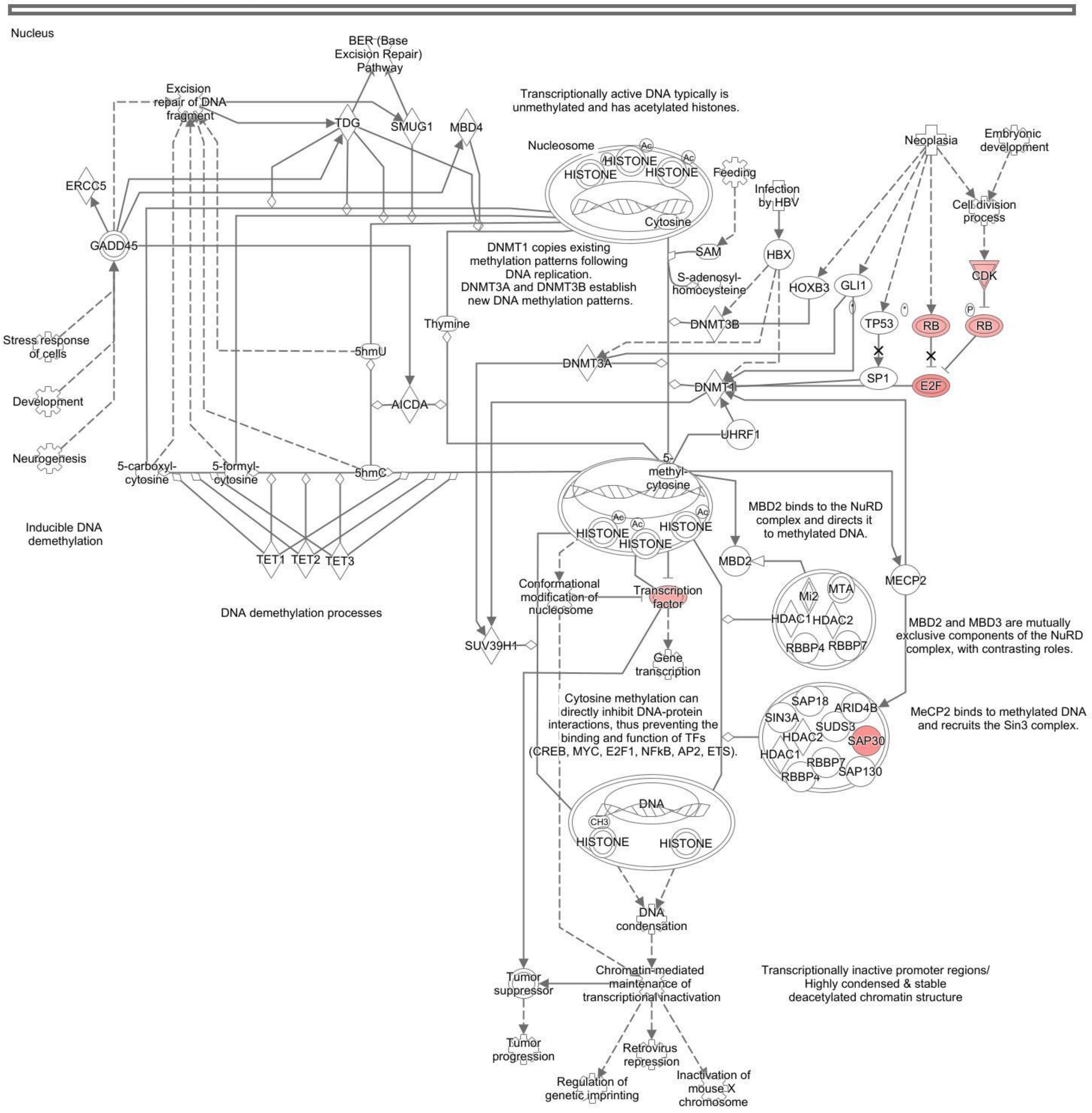

© 2000-2024 QIAGEN. All rights reserved.

Figure S120

DNA Methylation and Transcriptional Repression Signaling

Day 21
CAGE analysis

DNA Methylation and Transcriptional Repression Signaling

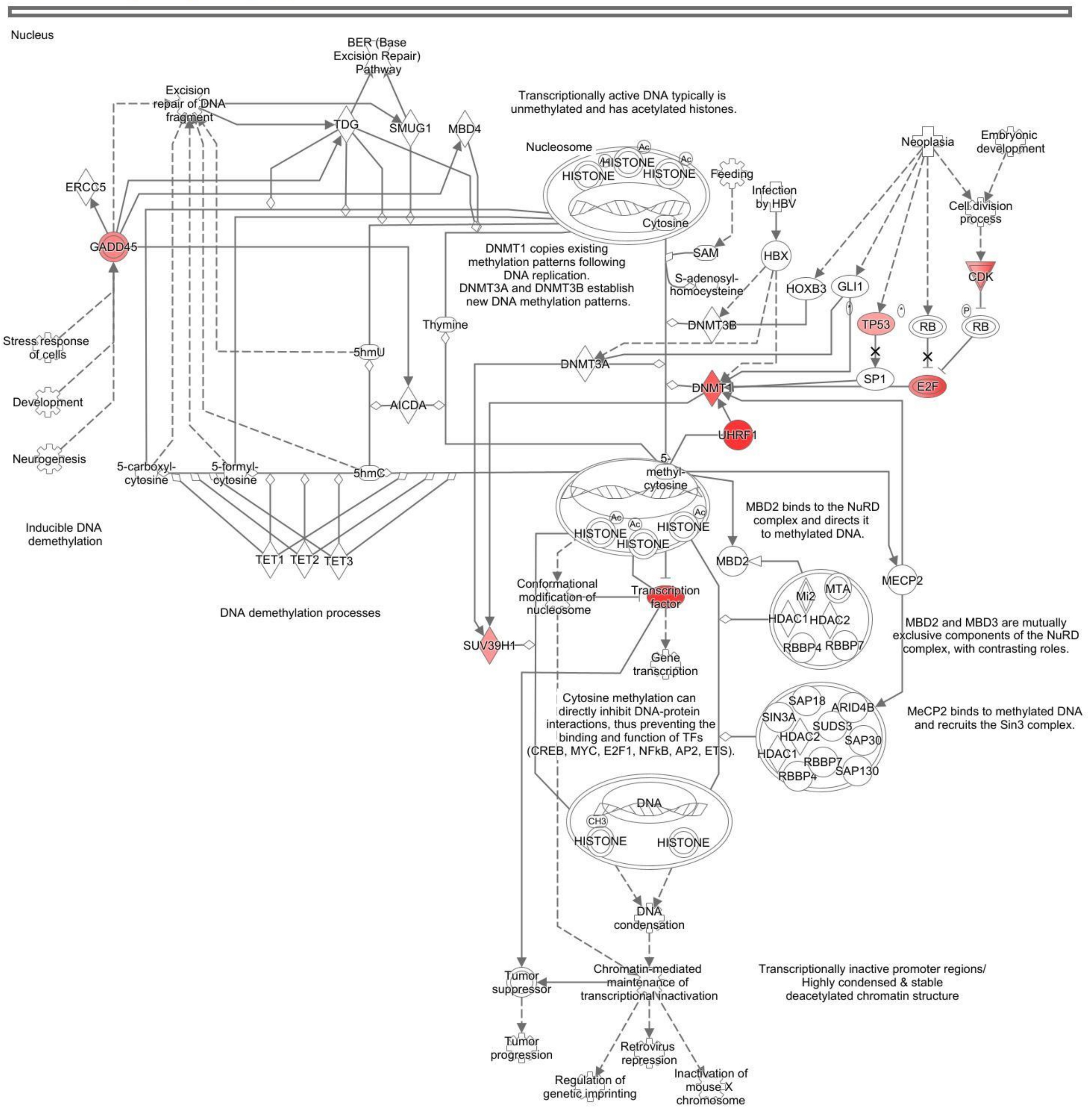

© 2000-2024 QIAGEN. All rights reserved.

Figure S121

Mismatch Repair in Eukaryotes

8 days
CAGE analysis

Mismatch Repair in Eukaryotes

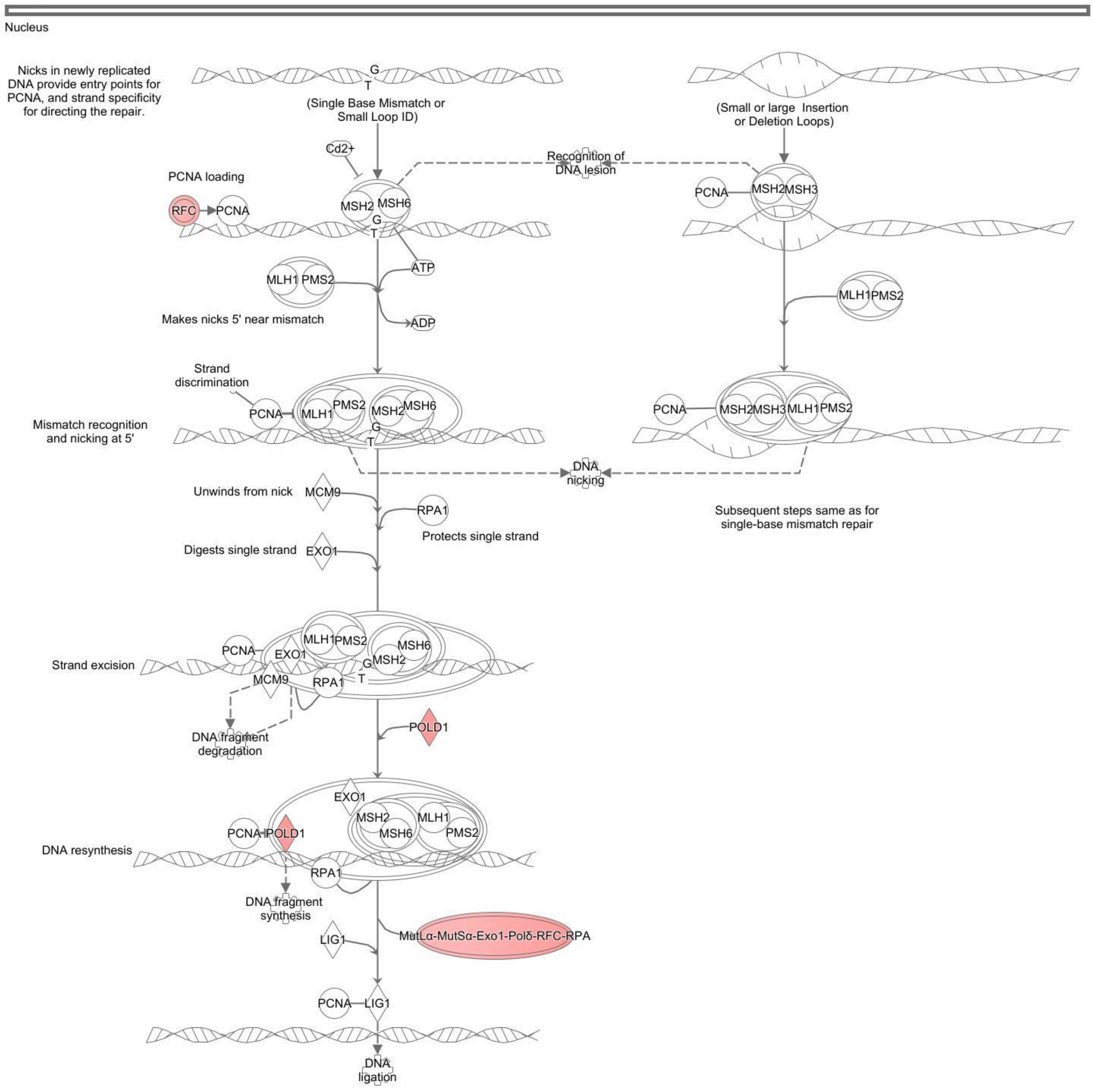

© 2000-2024 QIAGEN. All rights reserved.

Figure S122

Mismatch Repair in Eukaryotes

Day 21
CAGE analysis

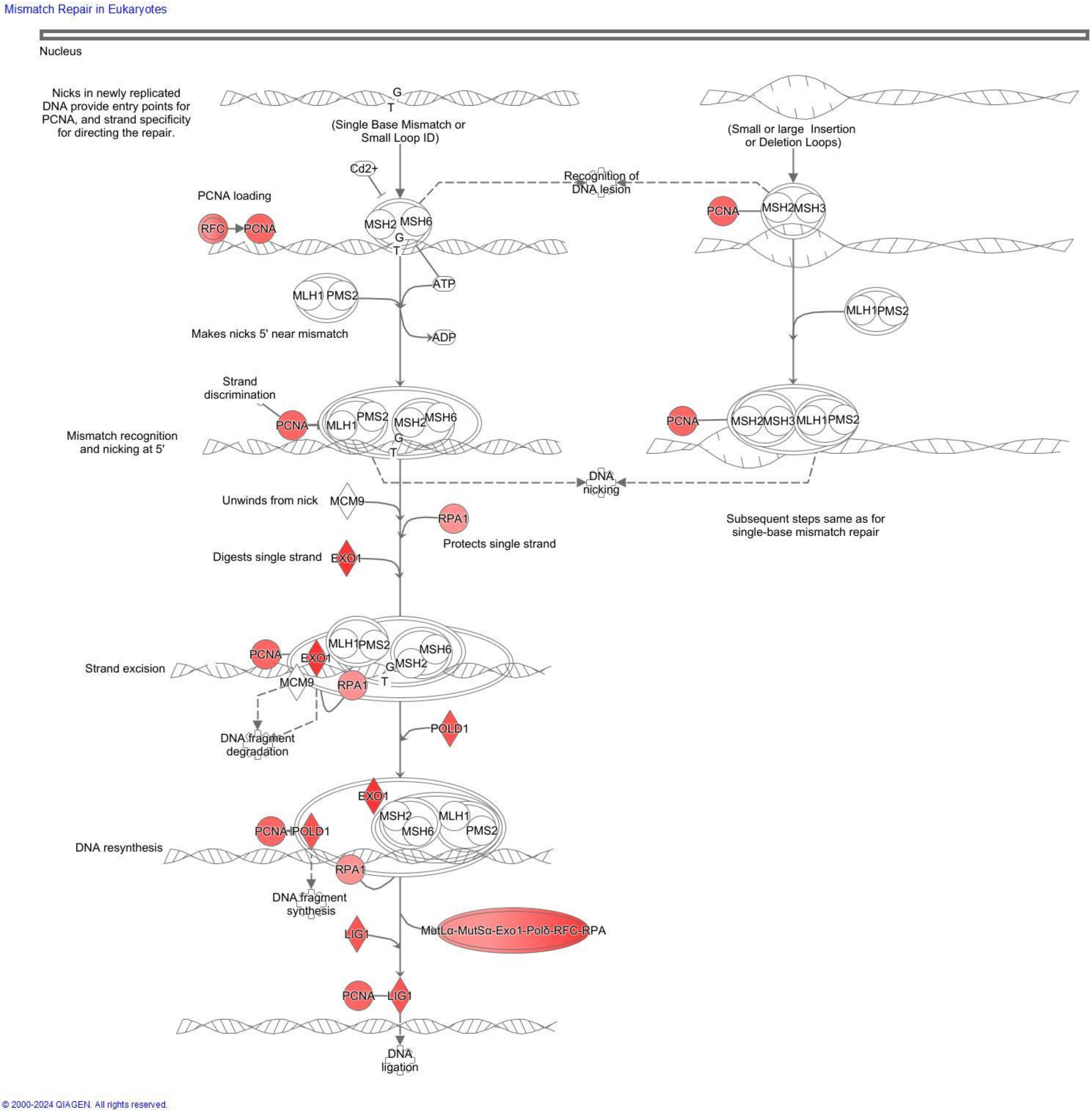

© 2000-2024 QIAGEN. All rights reserved.

Figure S123

Mitocondrial Dysfunction

6 hours
CAGE analysis

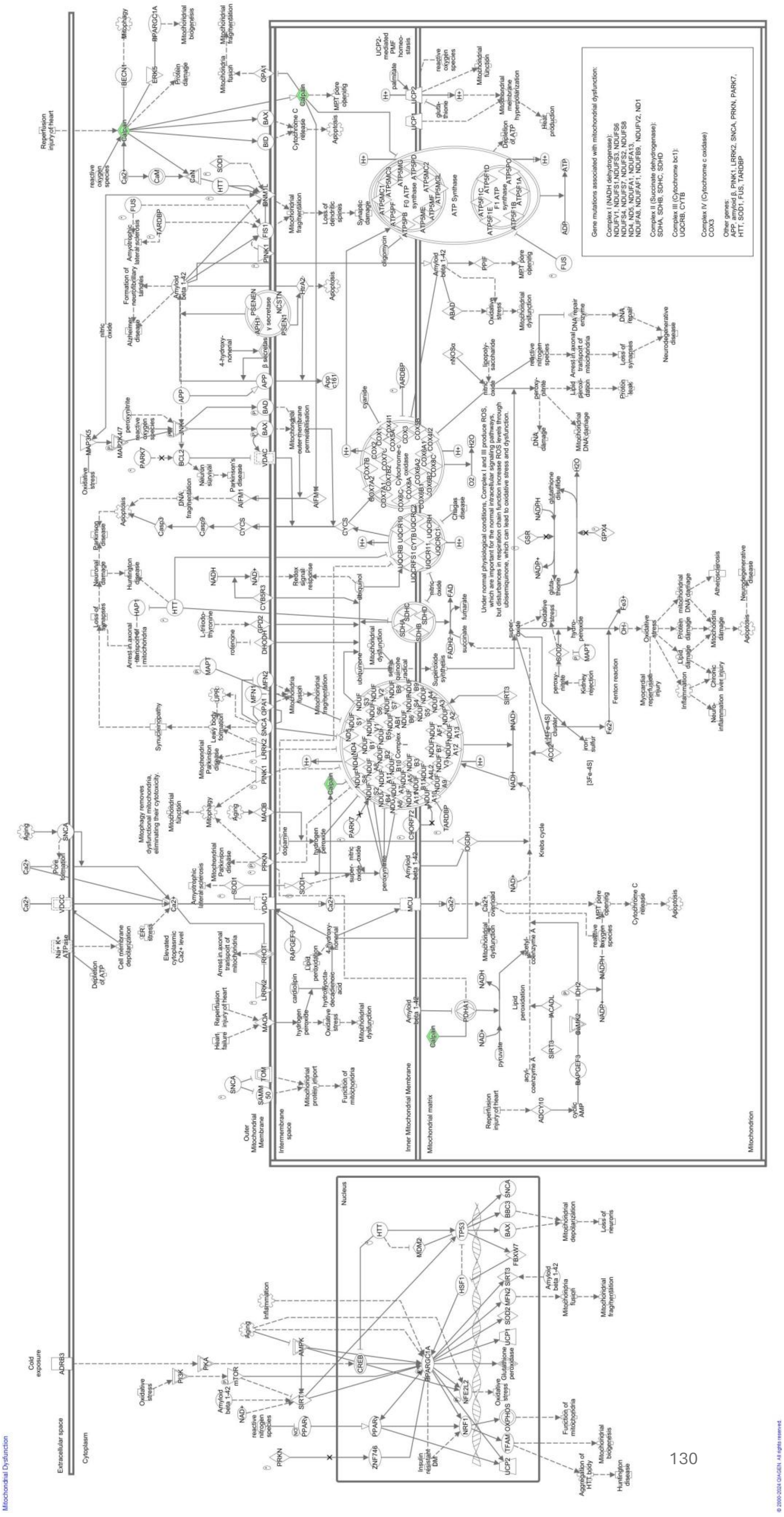

Figure S124

Mitocondrial Dysfunction

24 hours
CAGE analysis

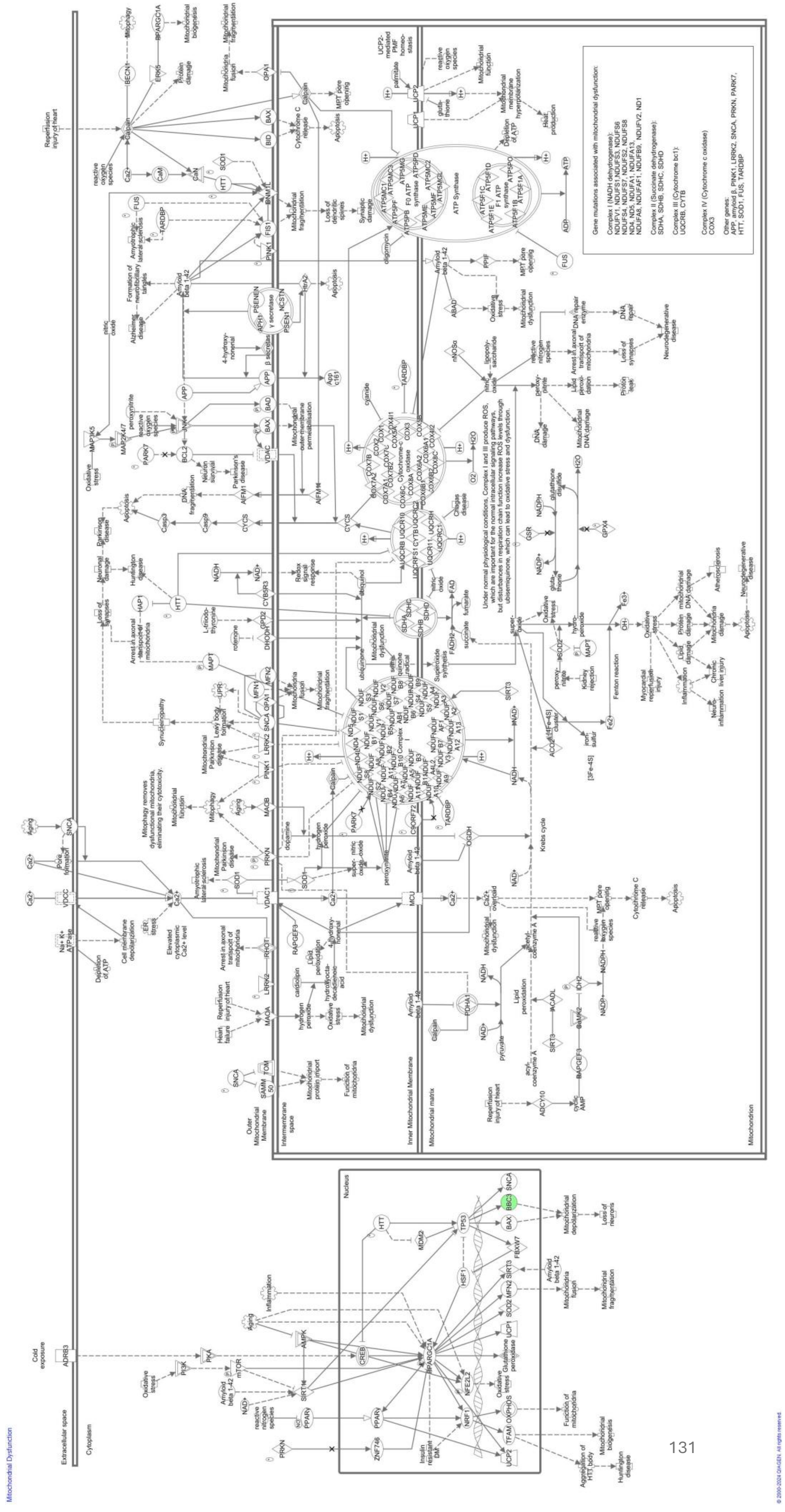

Figure S125

Mitocondrial
Dysfunction

8 days
CAGE analysis

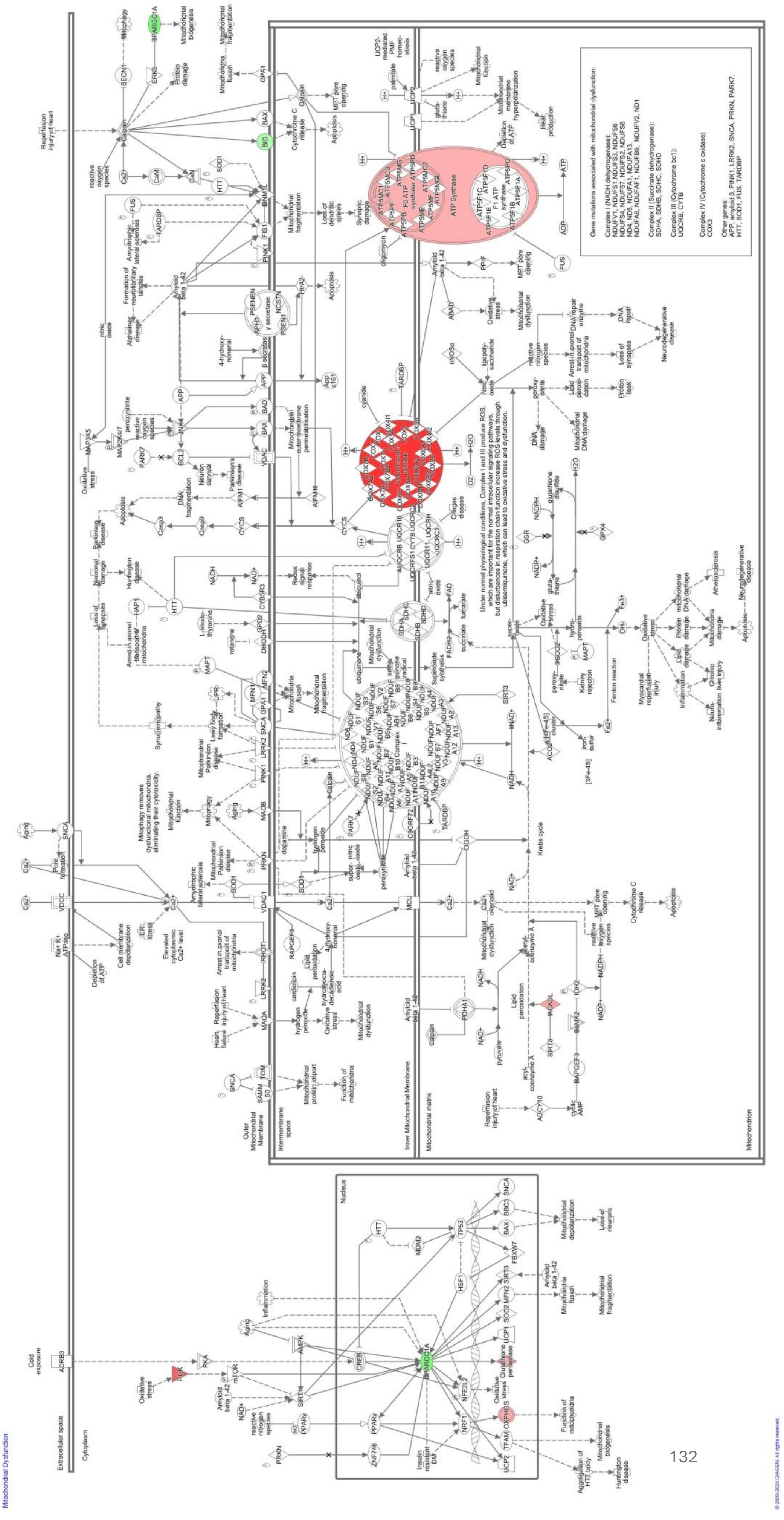

Figure S126

Mitocondrial Dysfunction

Day 21
CAGE analysis

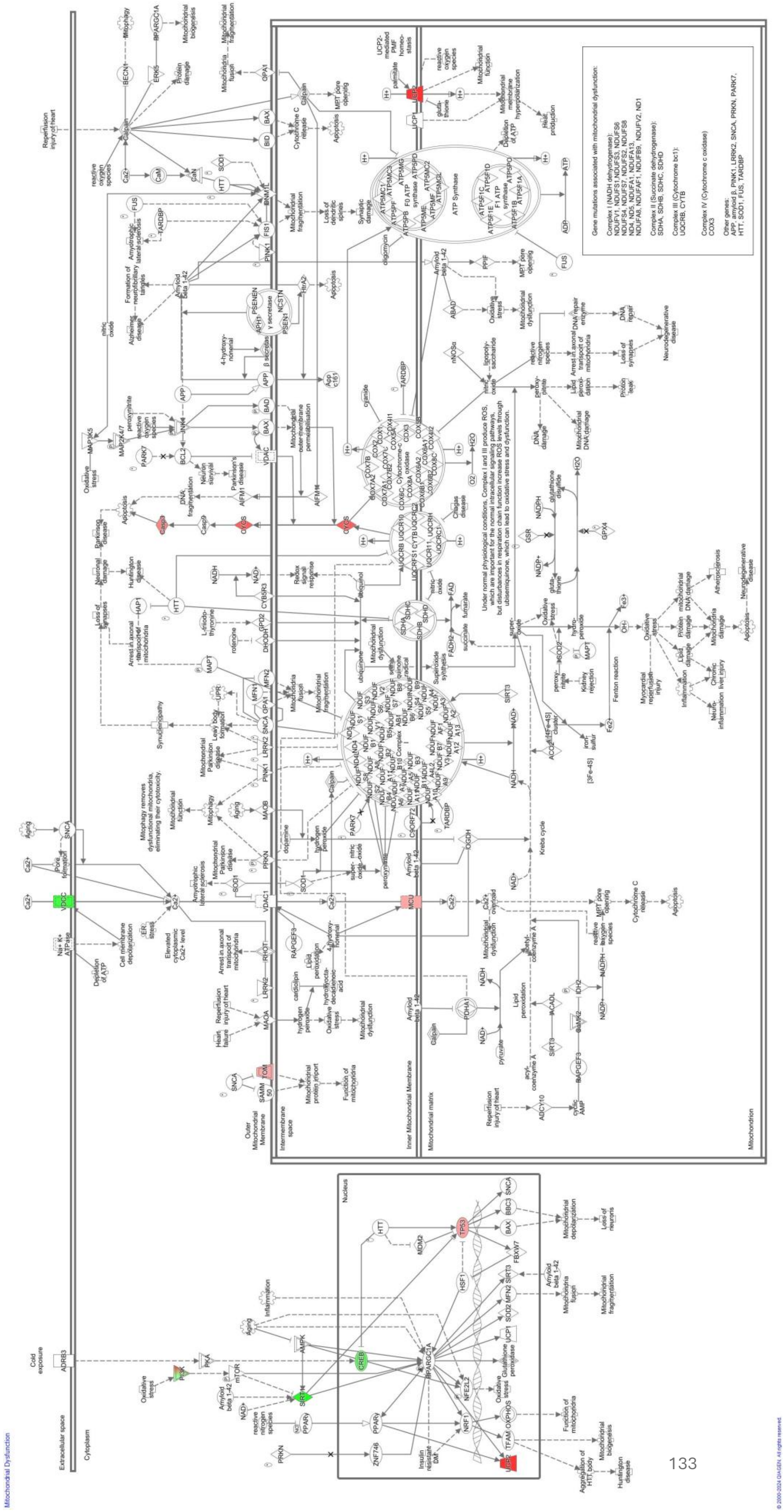

Figure S127

Senescence pathway

24 hours
CAGE analysis

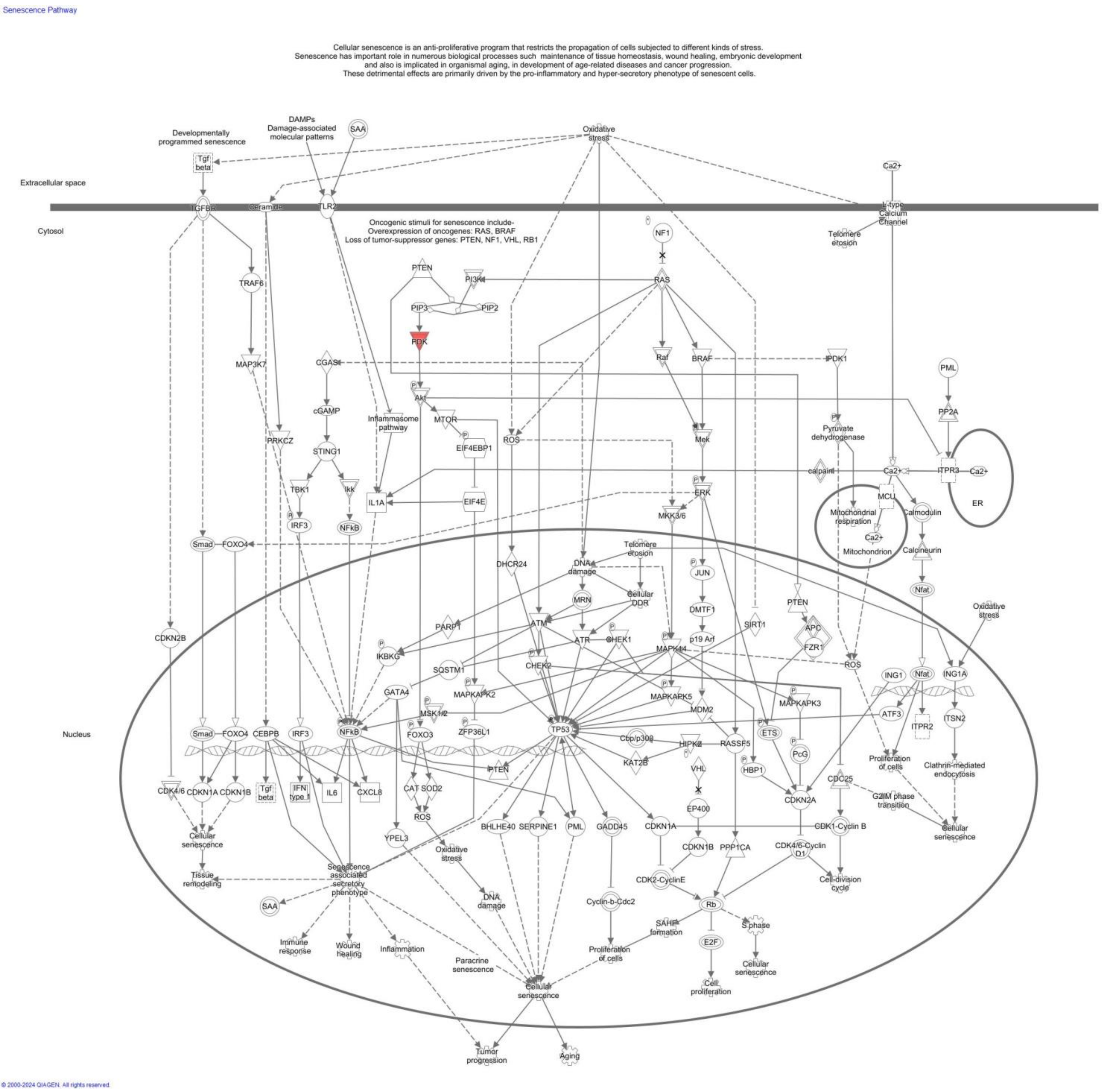

Figure S128

Senescence pathway

8 days
CAGE analysis

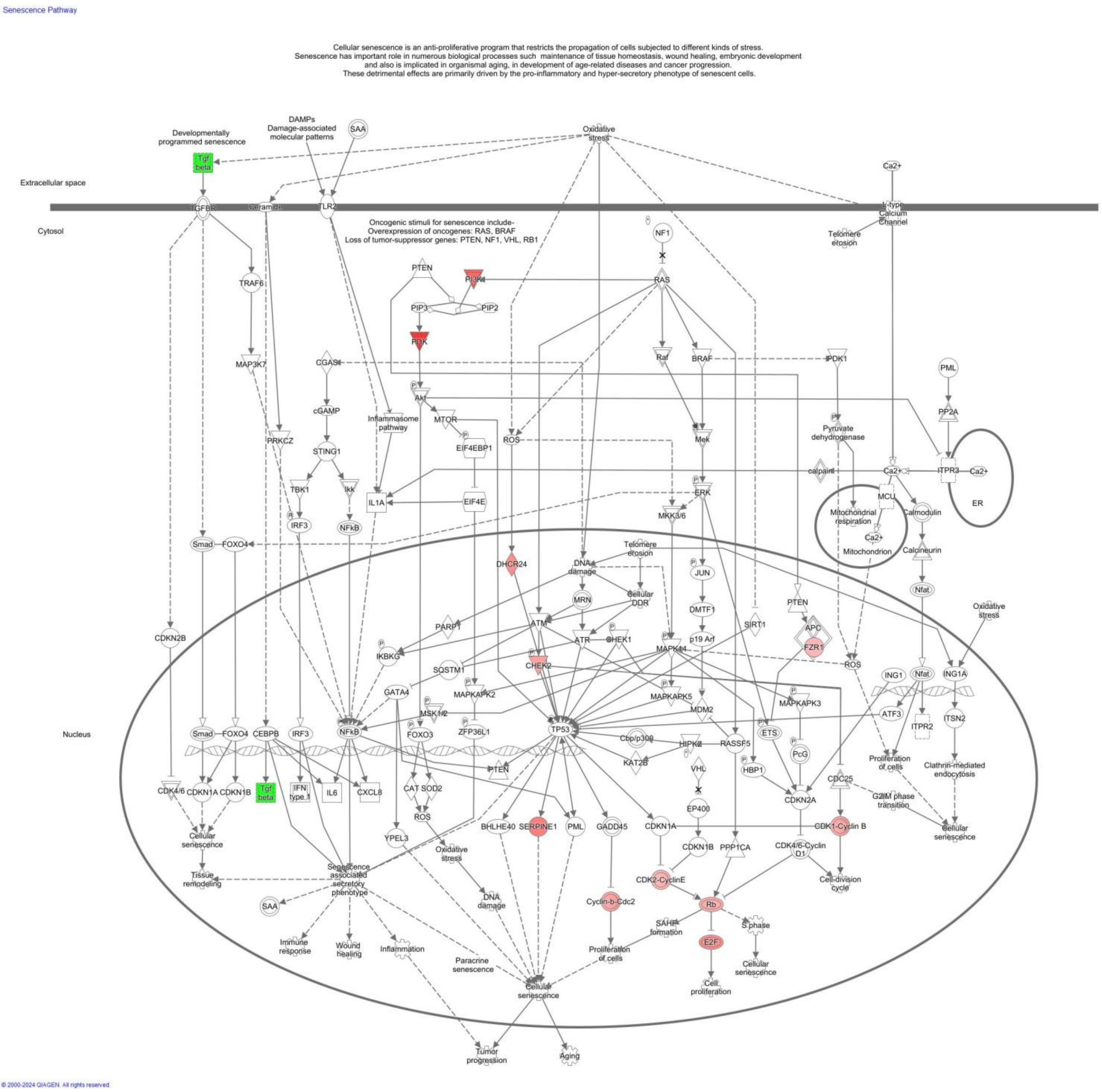

Figure S129

Senescence pathway

Day 21
CAGE analysis

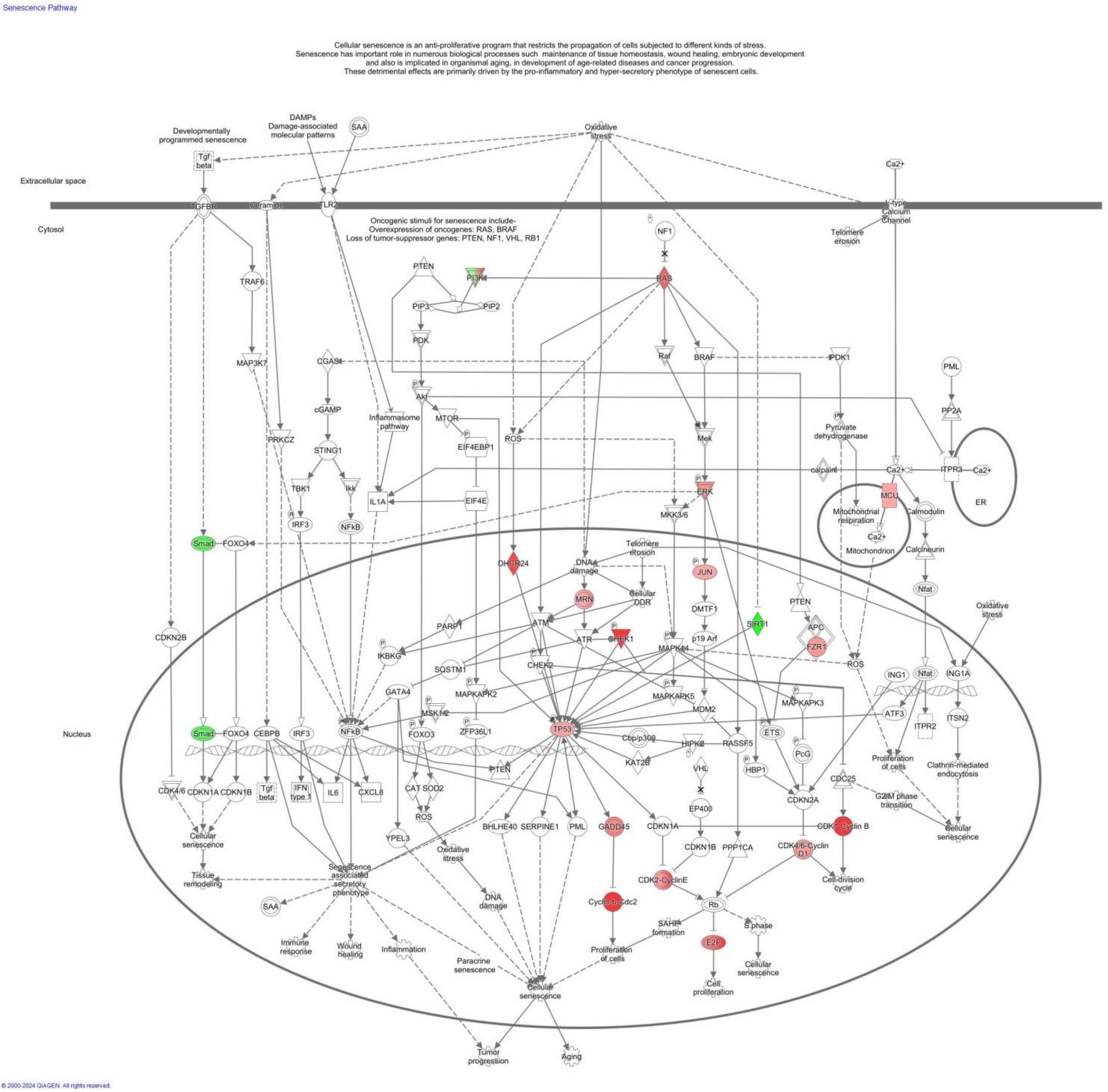

Figure S130

Senescence-Associated Secretory Phenotype (SASP)

24 hours
CAGE analysis

Senescence-Associated Secretory Phenotype (SASP) : NC24h-PA24h_FDR0.05_log2(PFOAvsNC)_Gene : Expr Log Ratio

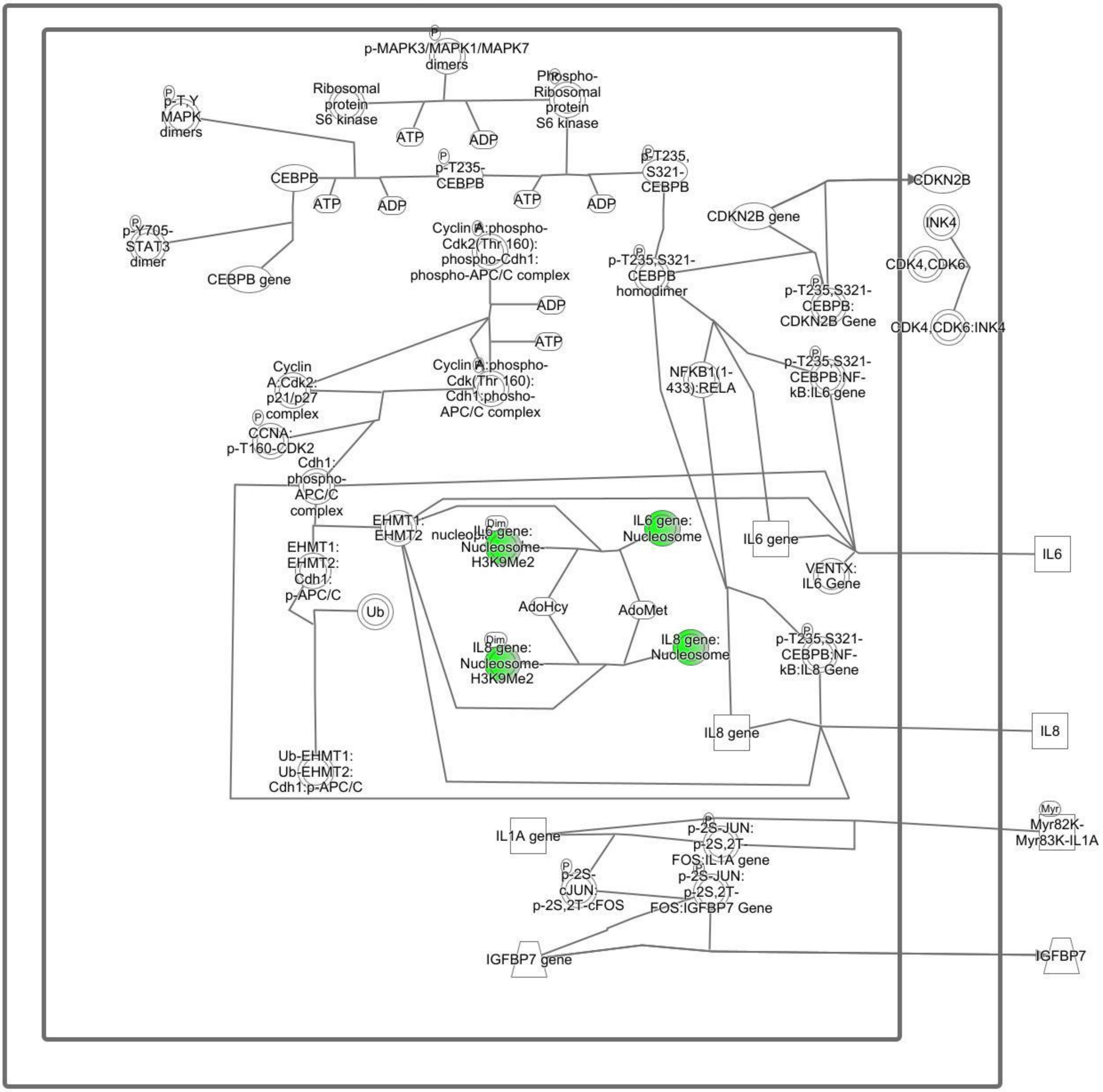

© 2000-2024 QIAGEN. All rights reserved.

Figure S131

Senescence-Associated Secretory Phenotype (SASP)

8 days
CAGE analysis

Senescence-Associated Secretory Phenotype (SASP) : NC12d-PA12d_FDR0.05_log2(PFOAvsNC)_Gene : Expr Log Ratio

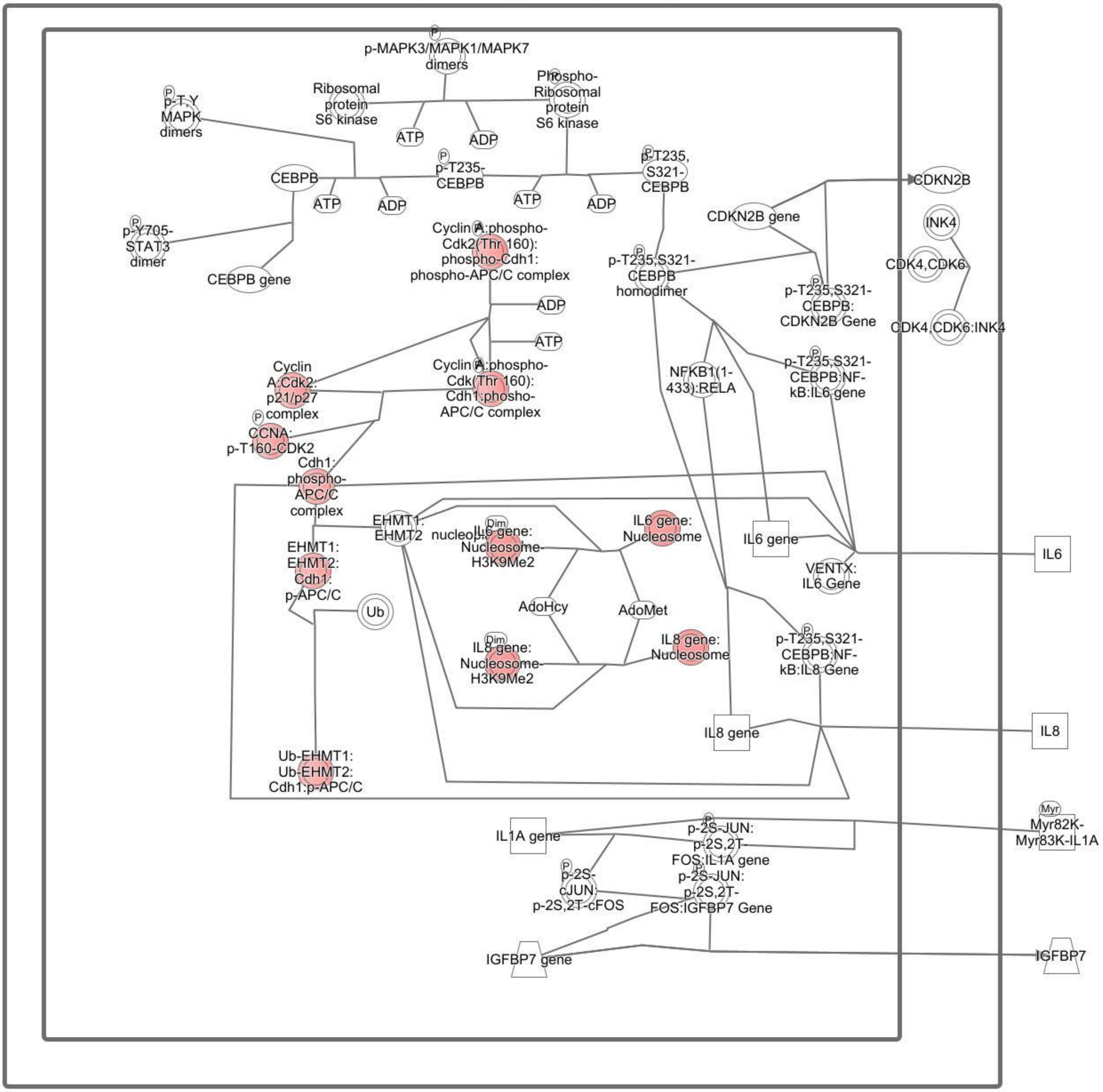

© 2000-2024 QIAGEN. All rights reserved.

Figure S132

Senescence-Associated Secretory Phenotype (SASP)

Day 21
CAGE analysis

Senescence-Associated Secretory Phenotype (SASP) : NC21d-PA21d_FDR0.05_log2(PFOAvsNC)_Gene : Expr Log Ratio

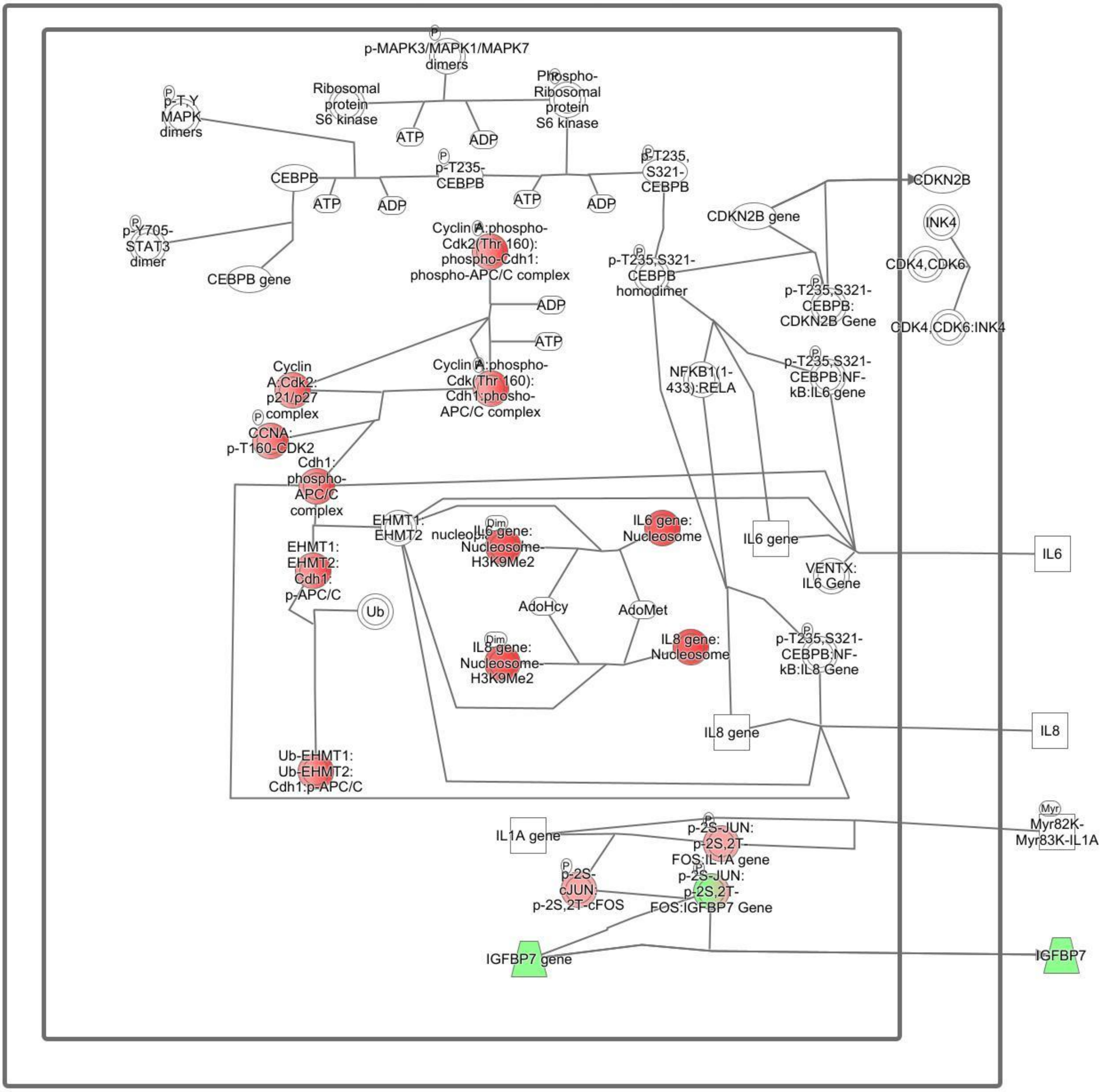

© 2000-2024 QIAGEN. All rights reserved.

Figure S133

Telomerase

8 days
CAGE analysis

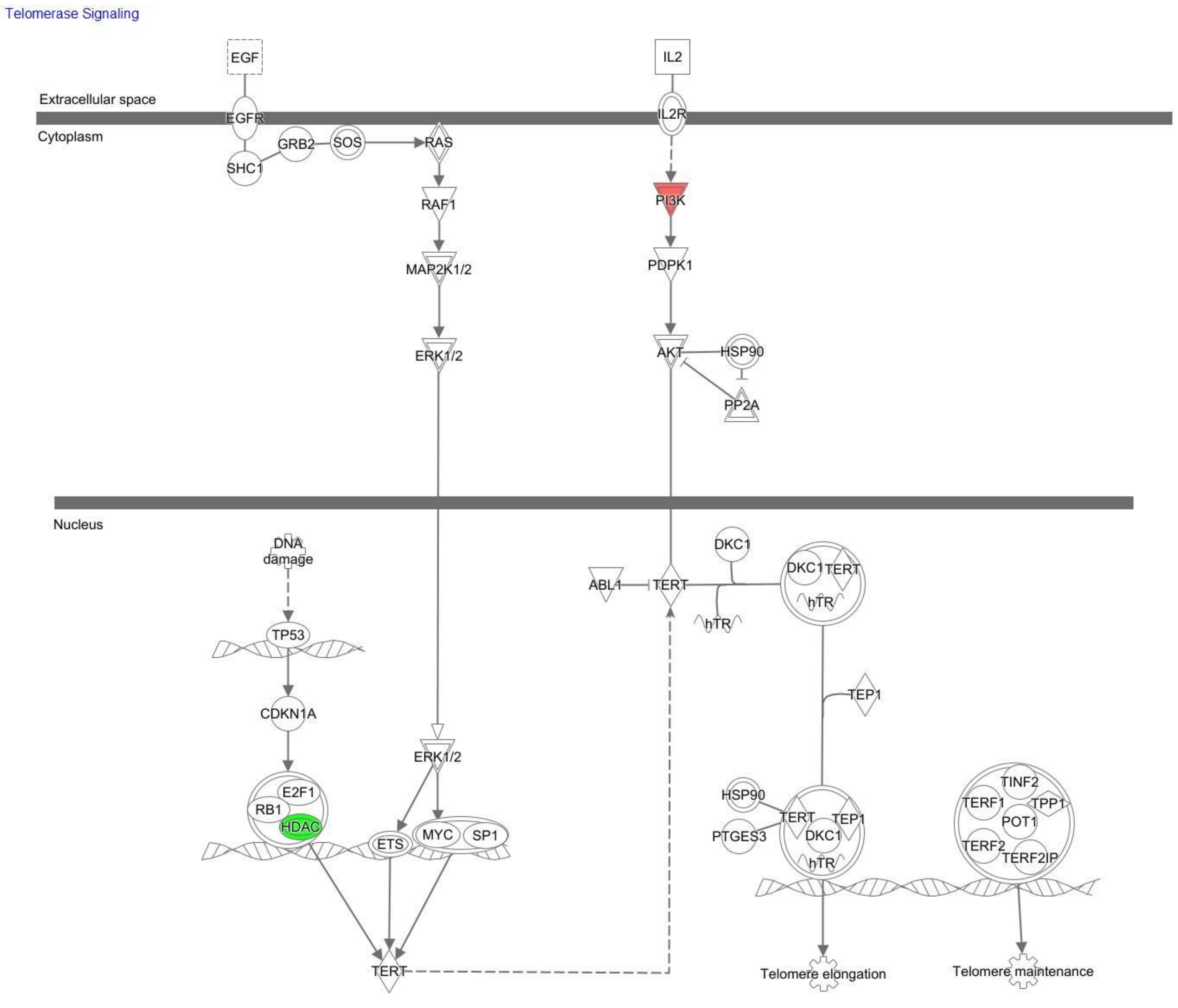

© 2000-2024 QIAGEN. All rights reserved.

Figure S134

Telomerase

Day 21
CAGE analysis

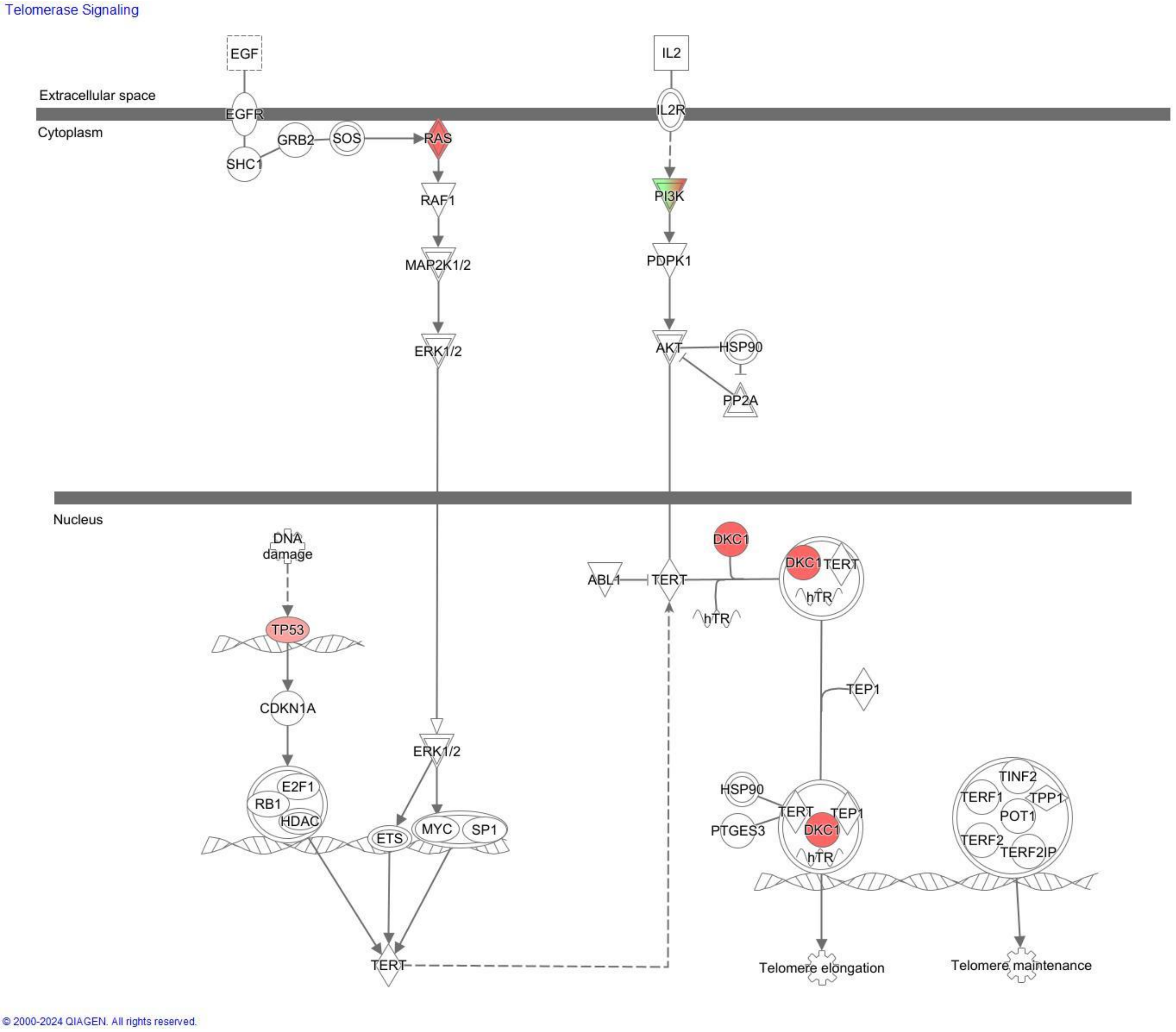

Figure S135

Telomere Extension by Telomerase

Day 21
CAGE analysis

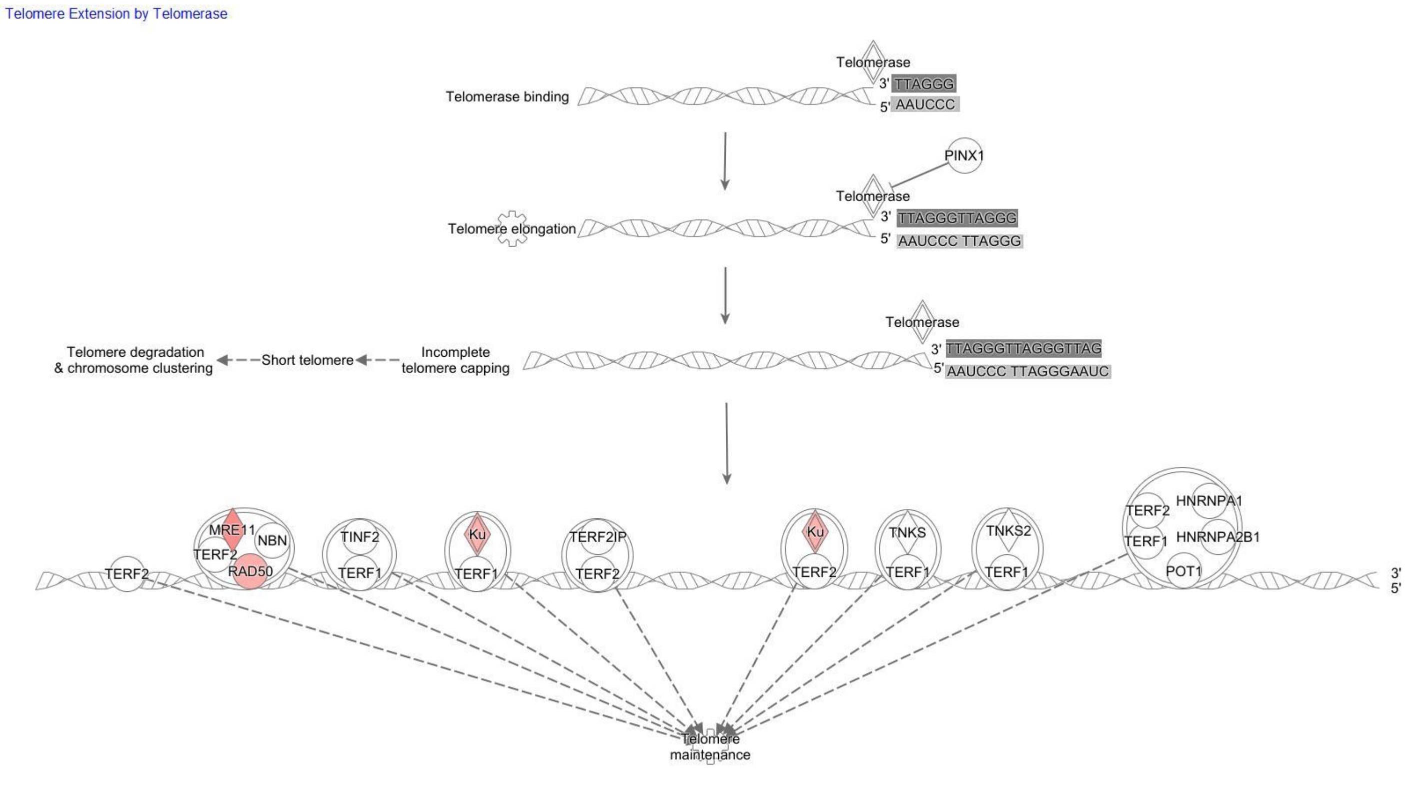

© 2000-2024 QIAGEN. All rights reserved.

Figure S136

Angiopoietin Signaling

1 hour
CAGE analysis

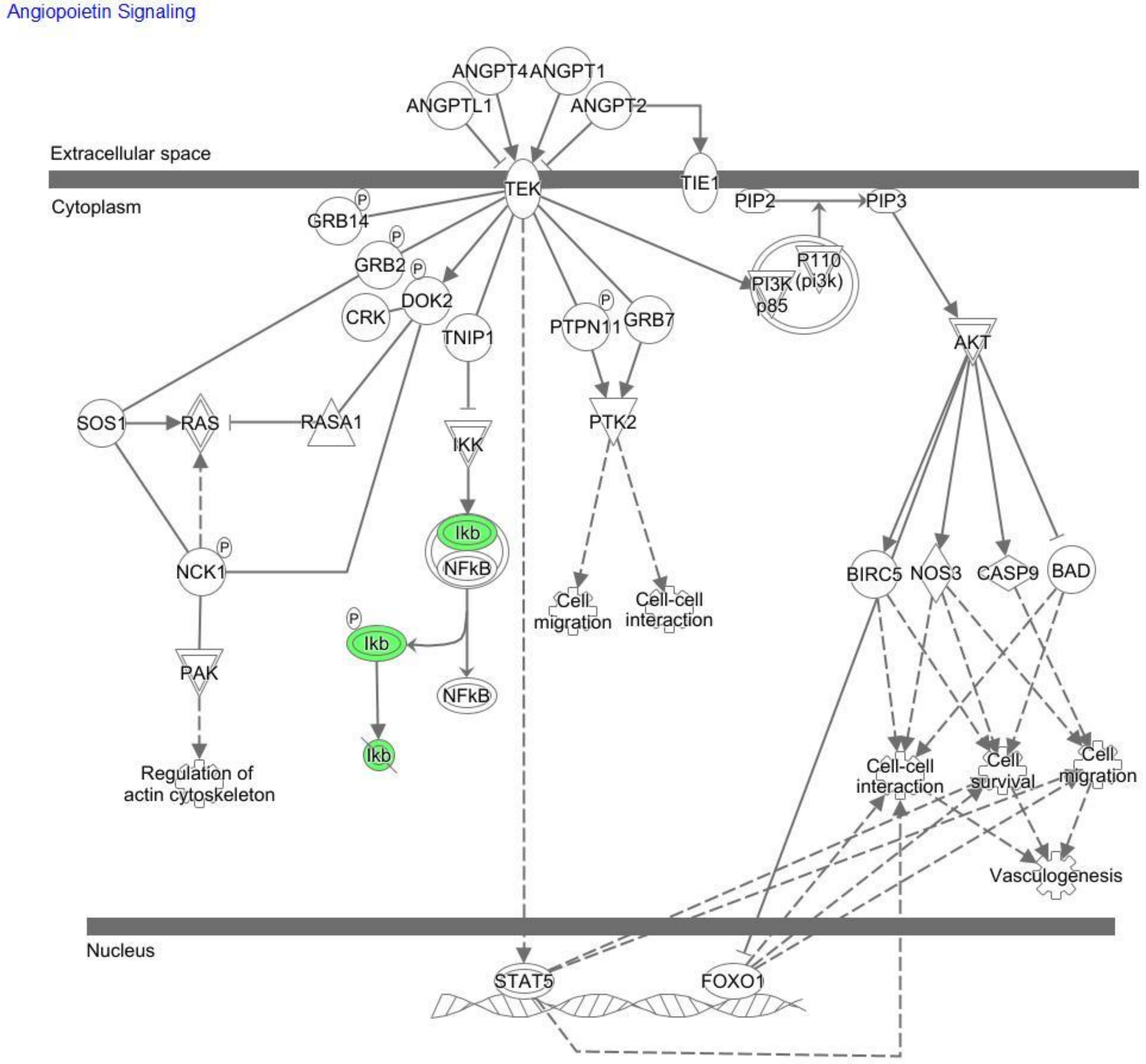

© 2000-2024 QIAGEN. All rights reserved.

Figure S137

Angiopoietin Signaling

8 days
CAGE analysis

Angiopoietin Signaling

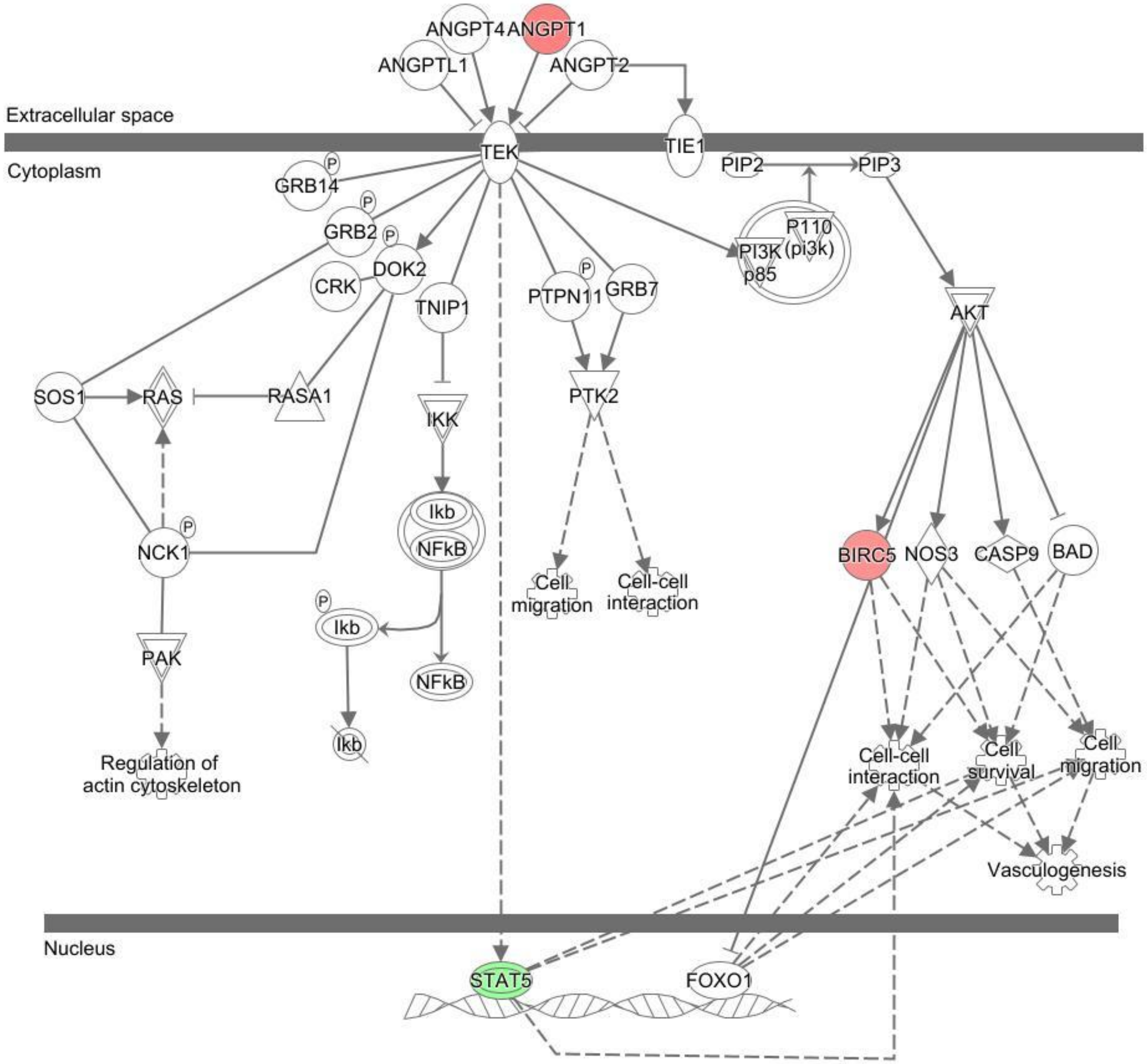

© 2000-2024 QIAGEN. All rights reserved.

Figure S138

Angiopoietin Signaling

Day 21
CAGE analysis

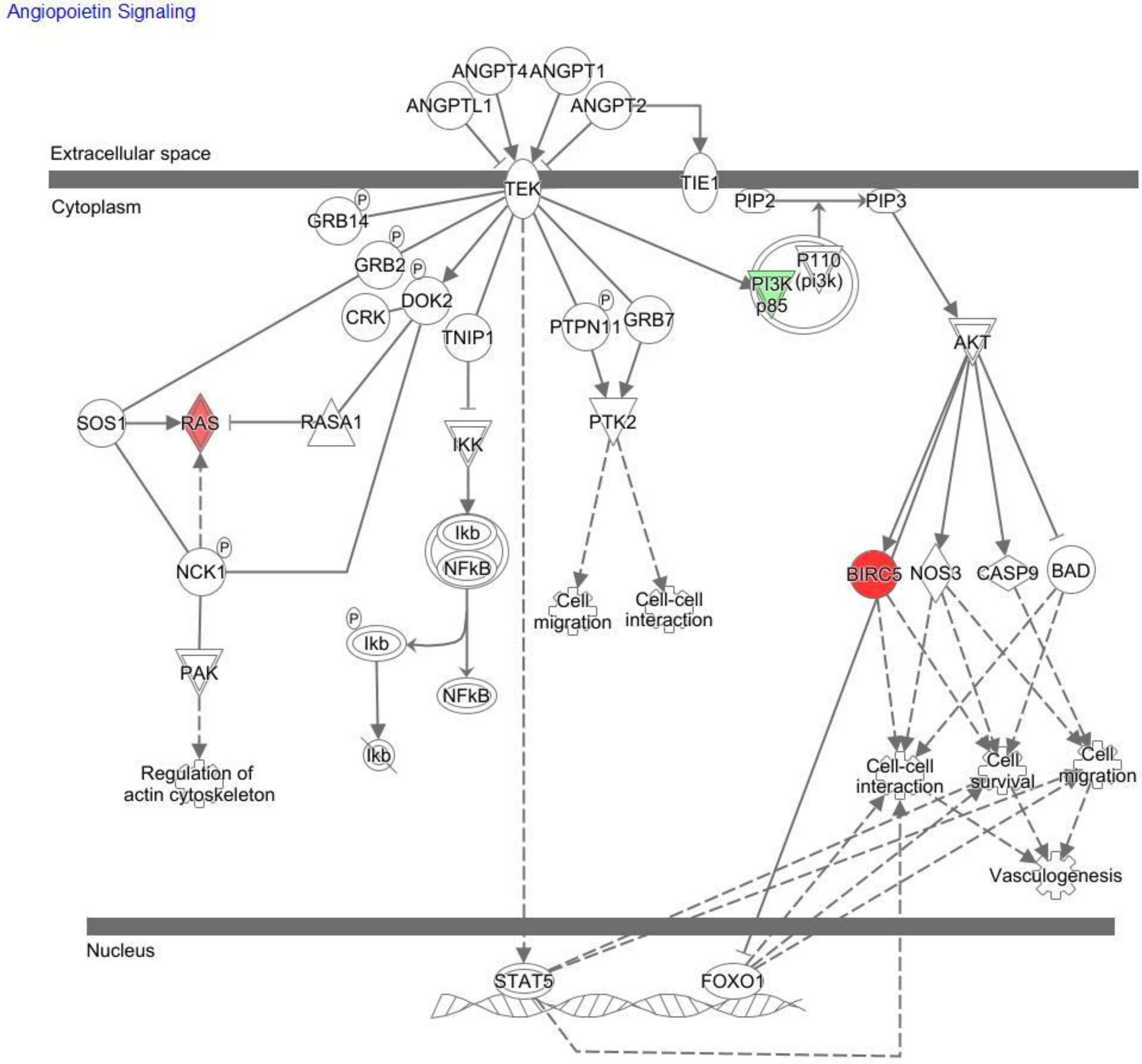

© 2000-2024 QIAGEN. All rights reserved.

Figure S139

Fatty acid β-oxidation I

24 hours
CAGE analysis

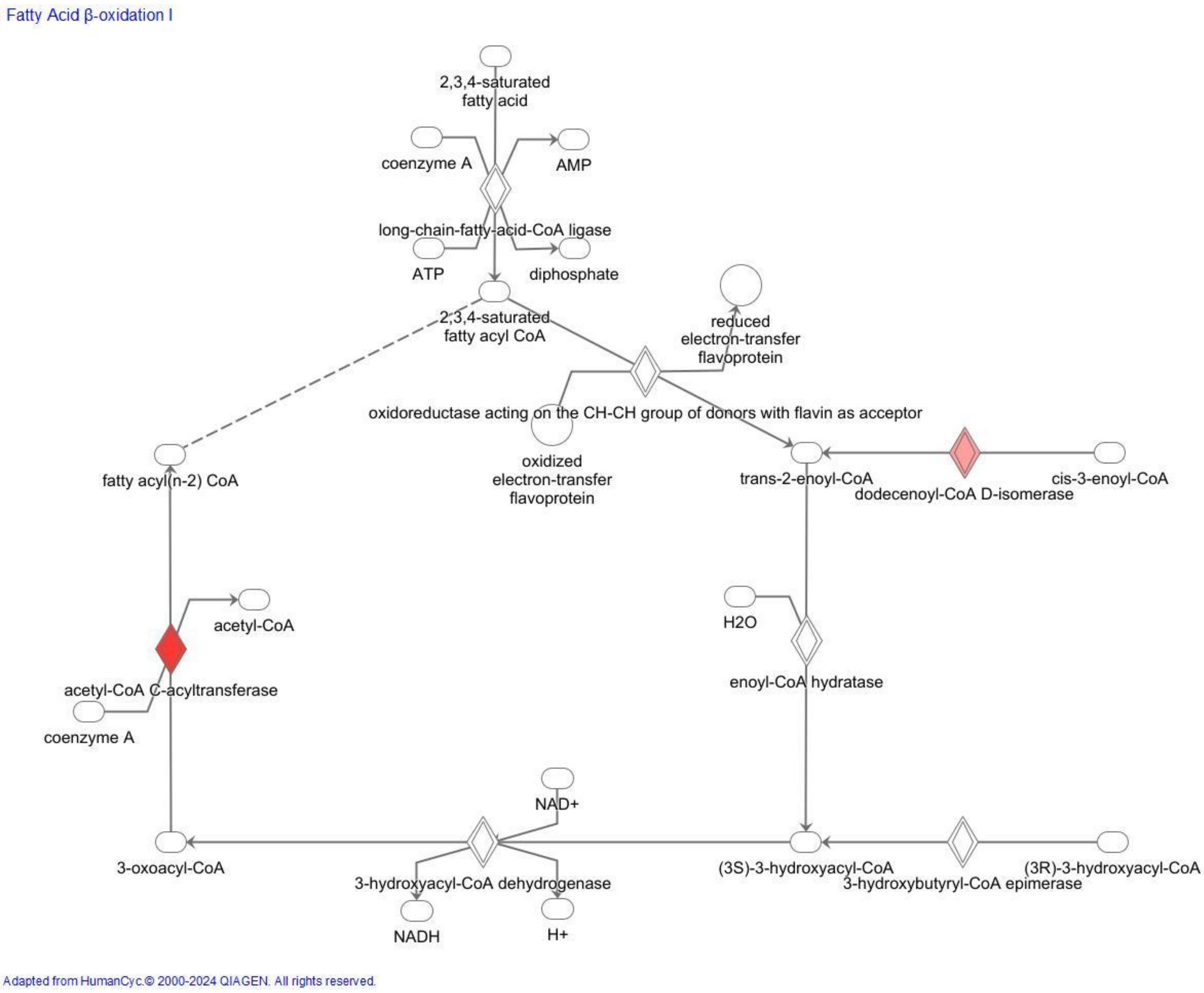

Figure S140

Fatty acid β-oxidation I

8 days
CAGE analysis

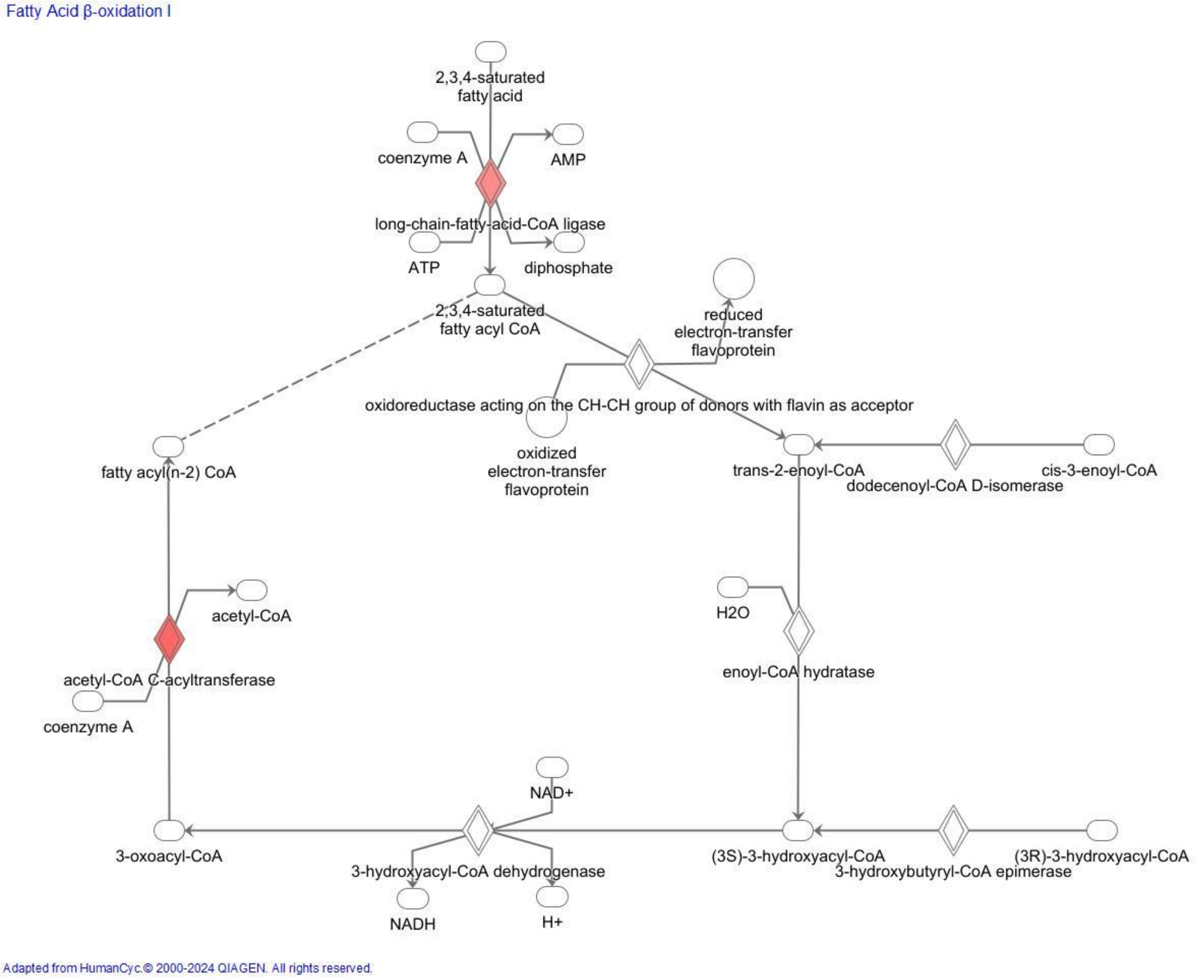

Adapted from HumanCyc.© 2000-2024 QIAGEN. All rights reserved.

Figure S141

Fatty acid β-oxidation I

Day 21
CAGE analysis

Fatty Acid β-oxidation I

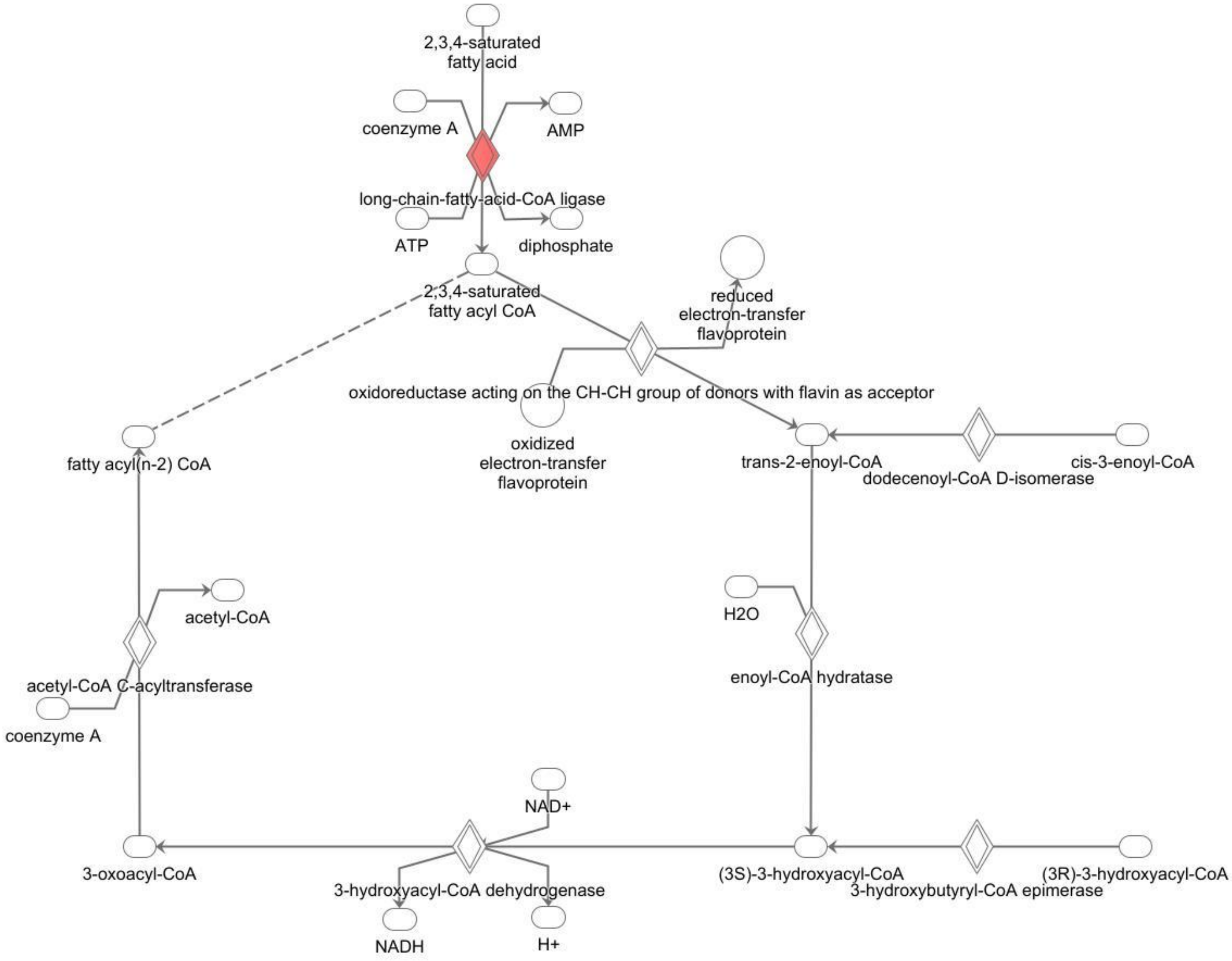

Adapted from HumanCyc.© 2000-2024 QIAGEN. All rights reserved.

Figure S142

Mitochondrial fatty acid beta oxidation

24 hours
CAGE analysis

Mitochondrial Fatty Acid Beta-Oxidation

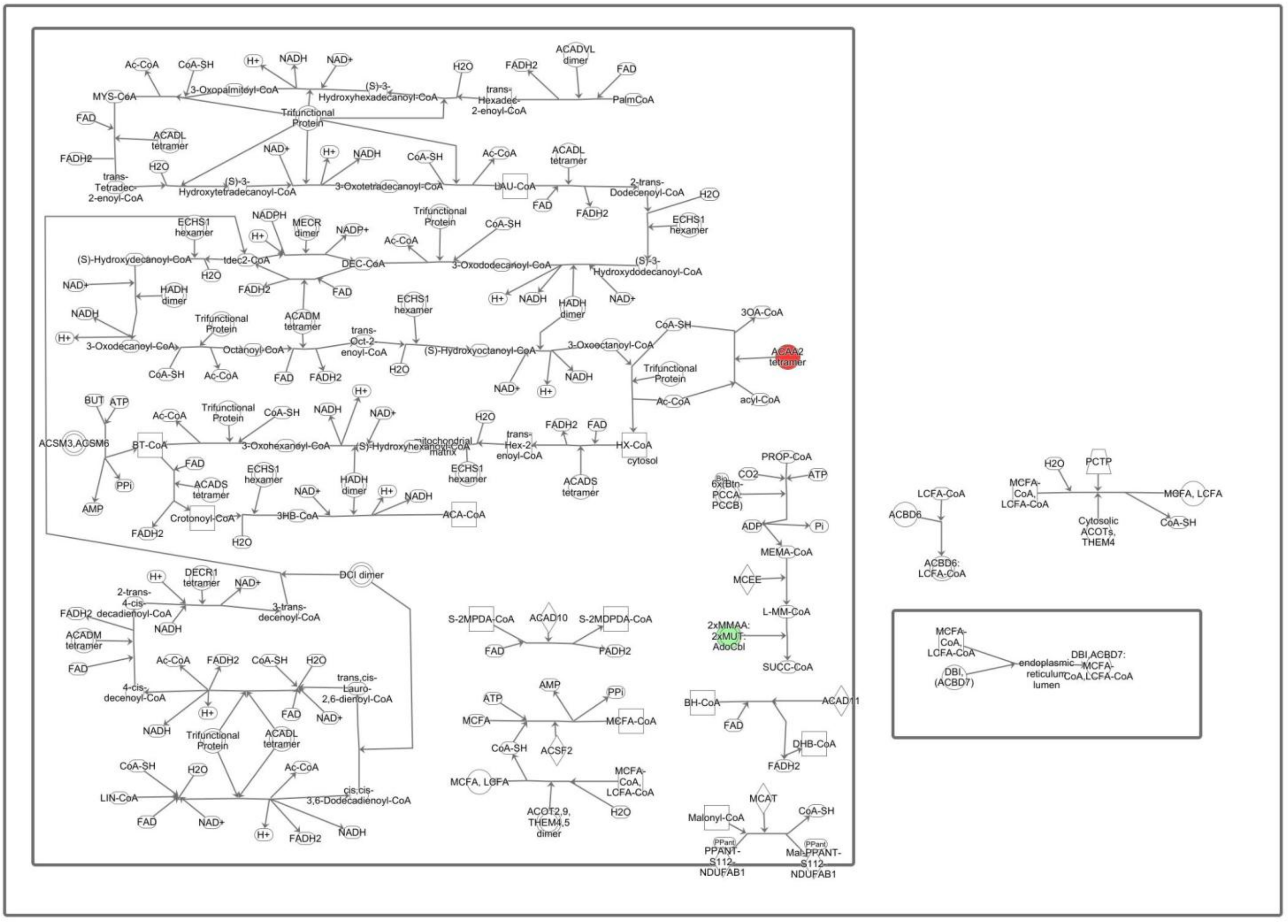

© 2000-2024 QIAGEN. All rights reserved.

Figure S143

Mitochondrial fatty acid beta oxidation

8 days
CAGE analysis

Mitochondrial Fatty Acid Beta-Oxidation

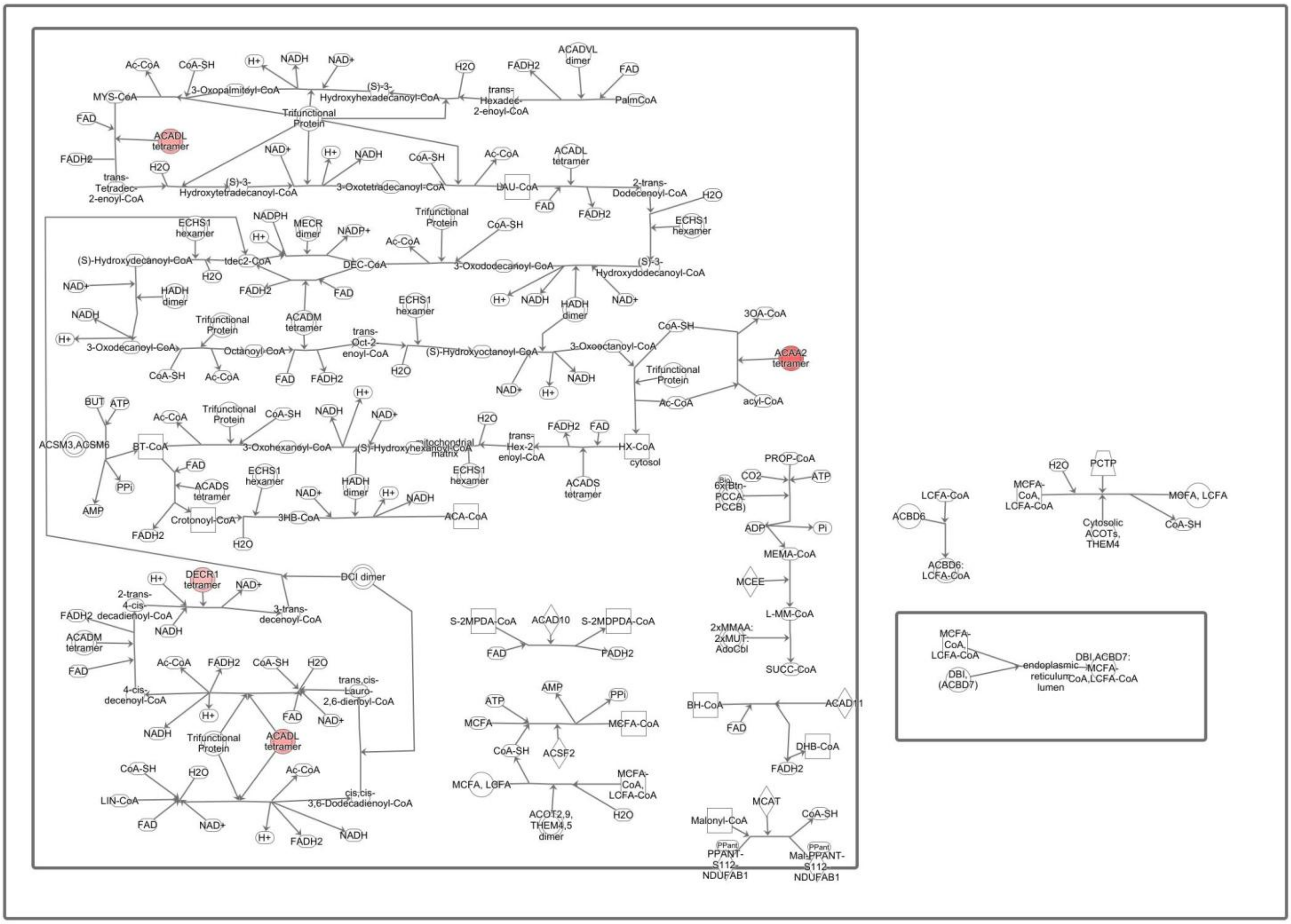

© 2000-2024 QIAGEN. All rights reserved.

Figure S144

Mitochondrial fatty acid beta oxidation

Day 21
CAGE analysis

Mitochondrial Fatty Acid Beta-Oxidation

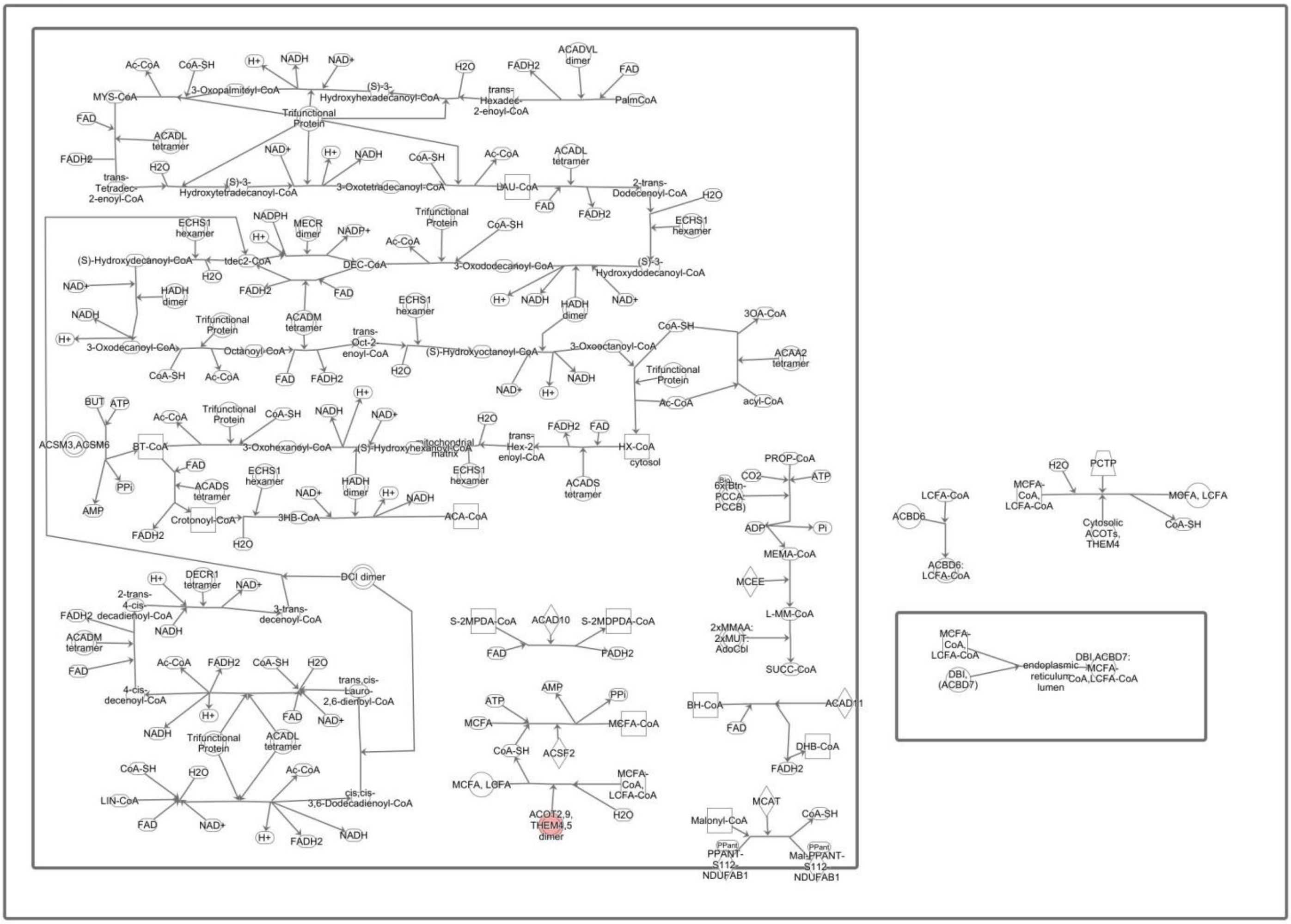

© 2000-2024 QIAGEN. All rights reserved.

Figure S145

PPAR signaling

1 hour
CAGE analysis

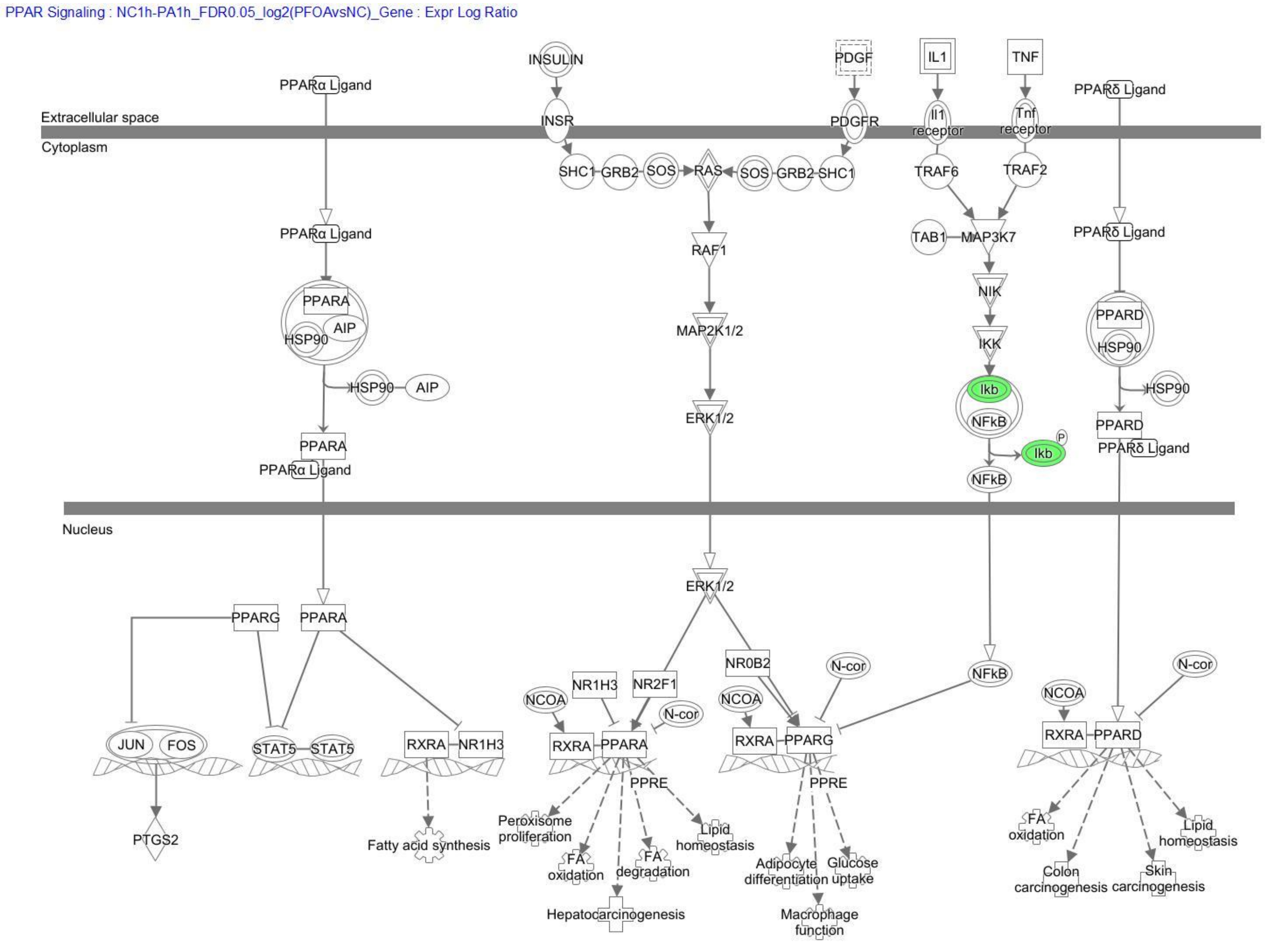

© 2000-2024 QIAGEN. All rights reserved.

Figure S146

PPAR signaling

8 days
CAGE analysis

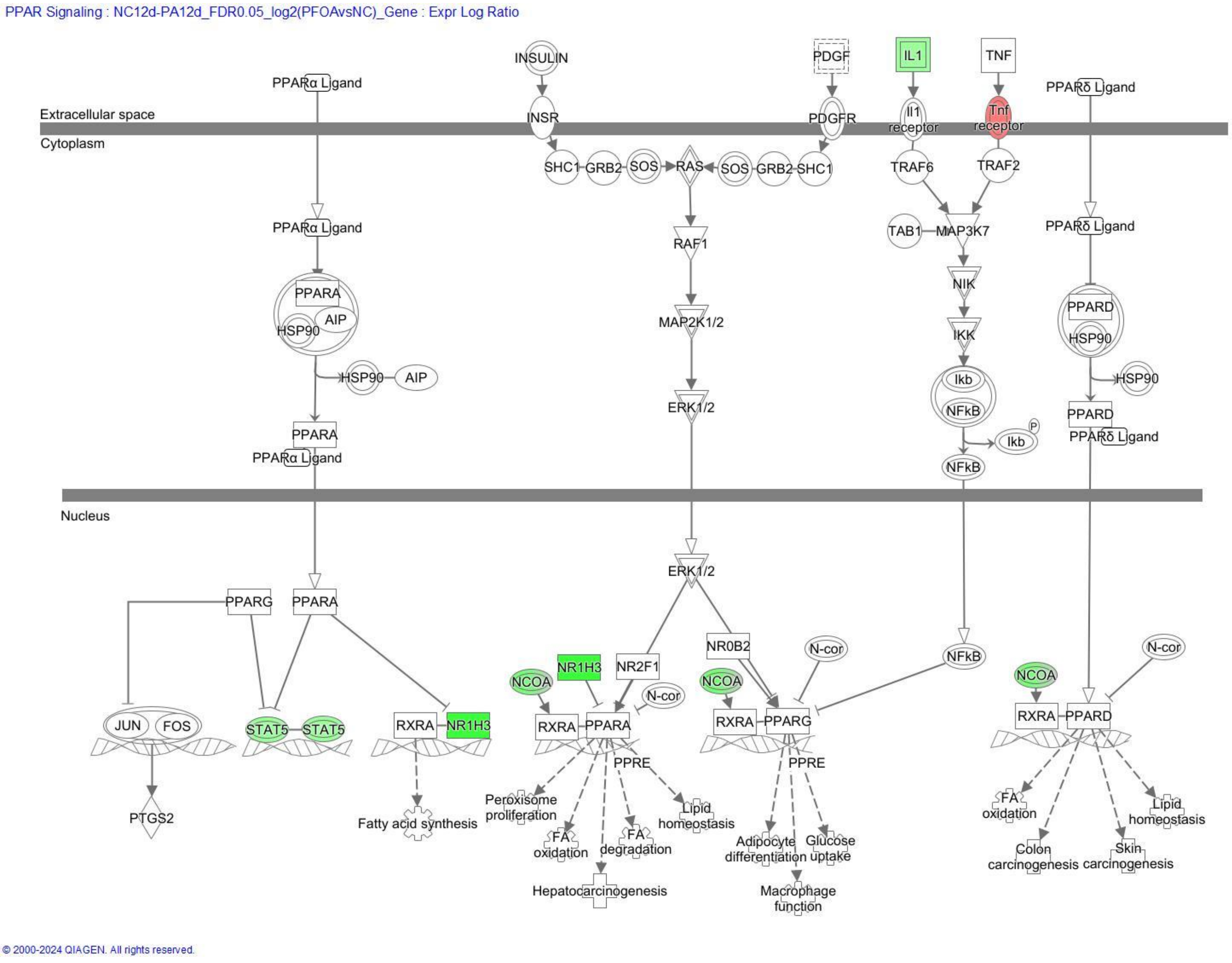

Figure S147

PPAR signaling

Day 21
CAGE analysis

PPAR Signaling : NC21d-PA21d_FDR0.05_log2(PFOAvsNC)_Gene : Expr Log Ratio

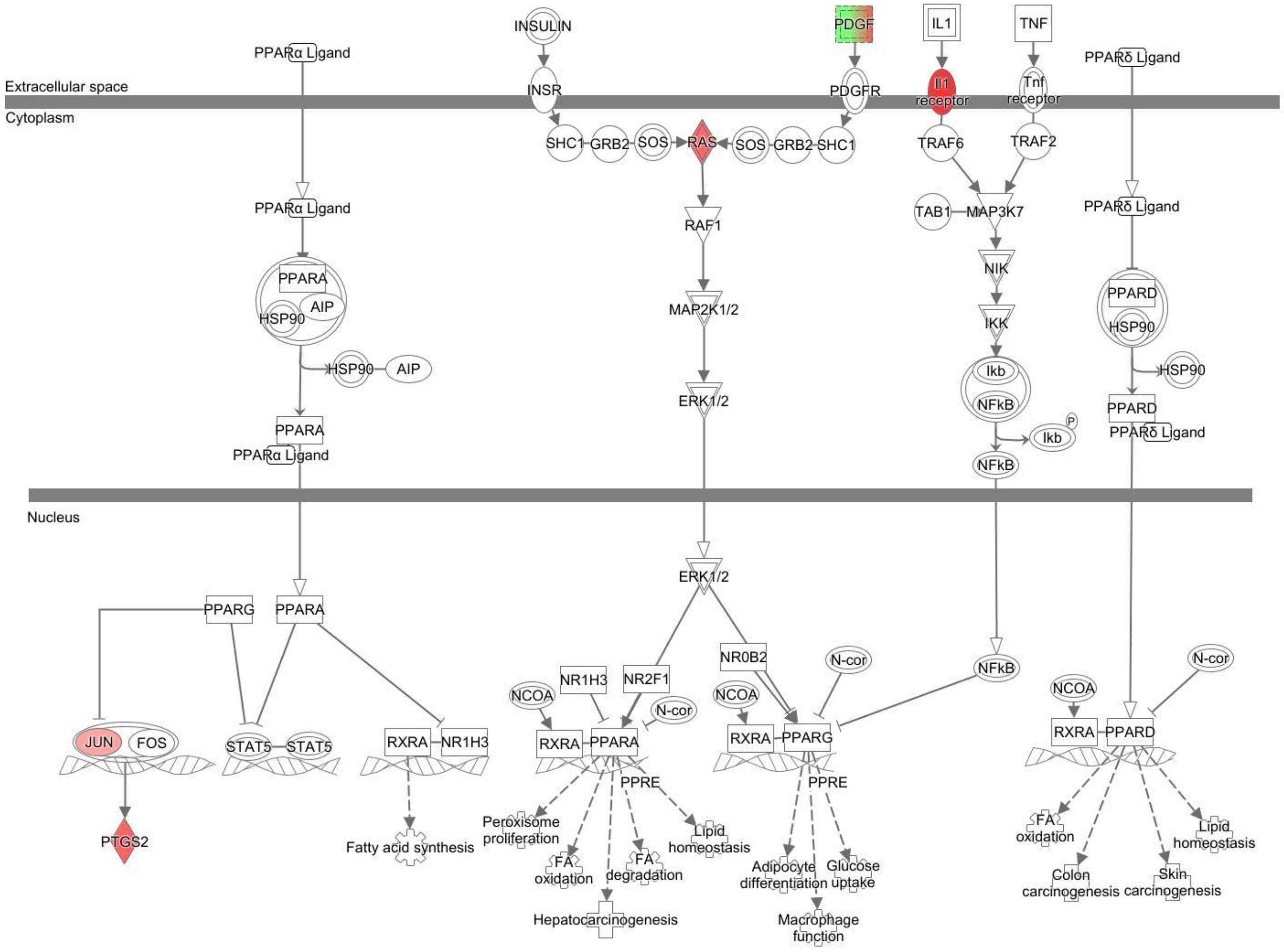

© 2000-2024 QIAGEN. All rights reserved.

Figure S148

AMPK signaling

6 hours
CAGE analysis

AMPK Signaling

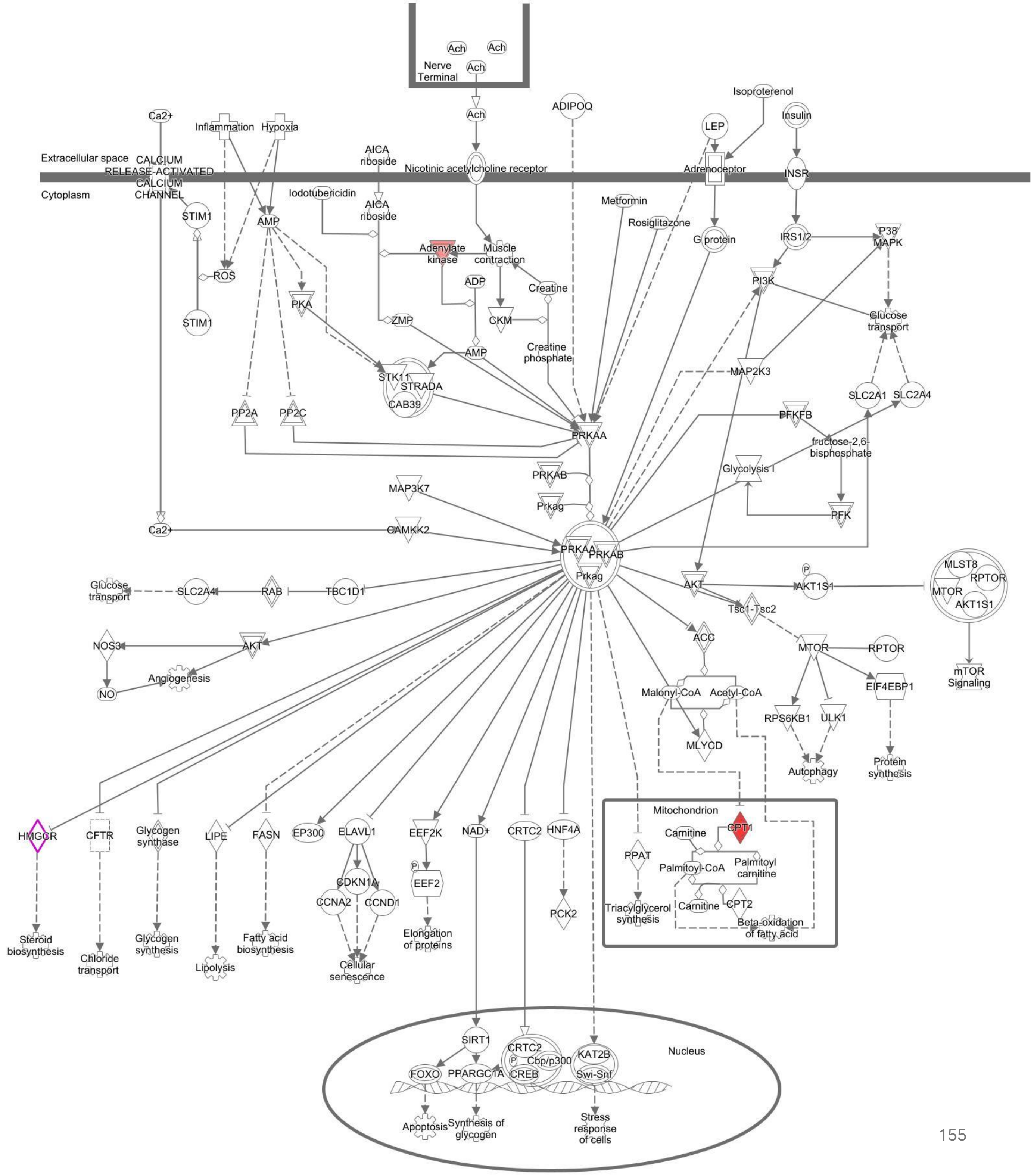

© 2000-2024 QIAGEN. All rights reserved.

Figure S149

AMPK signaling

24 hours
CAGE analysis

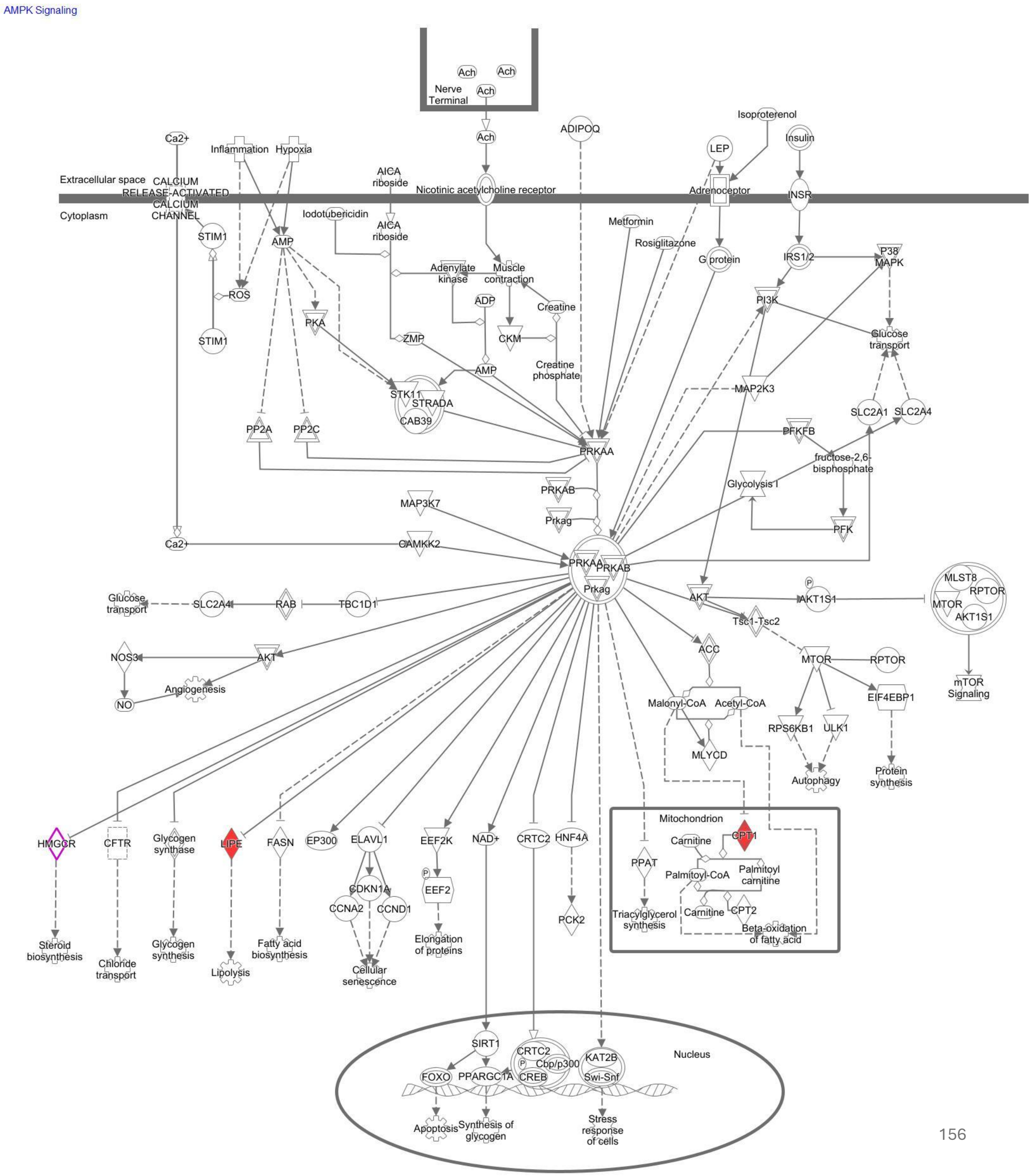

© 2000-2024 QIAGEN. All rights reserved.

Figure S150

AMPK signaling

8 days
CAGE analysis

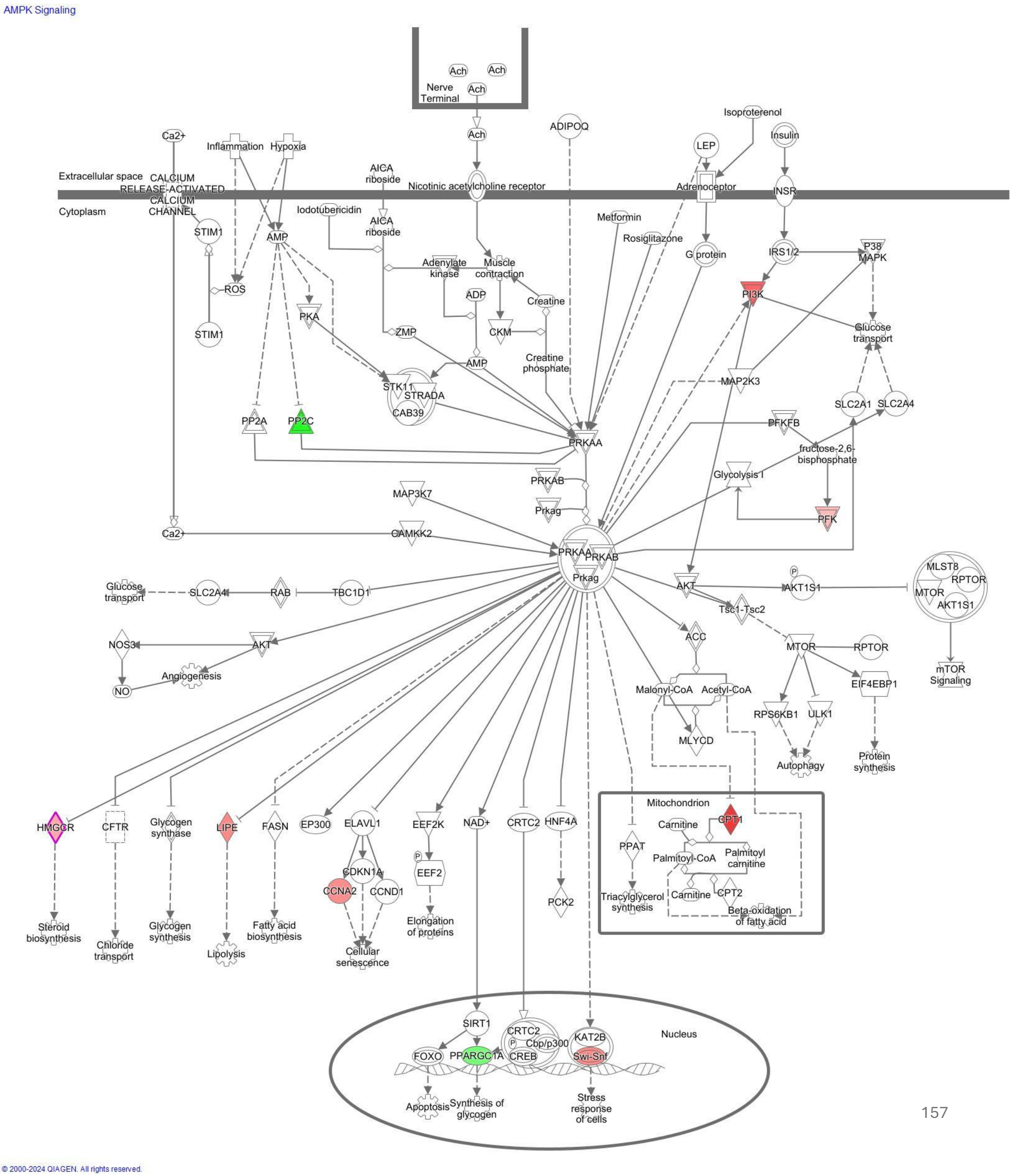

Figure S151

AMPK signaling

Day 21
CAGE analysis

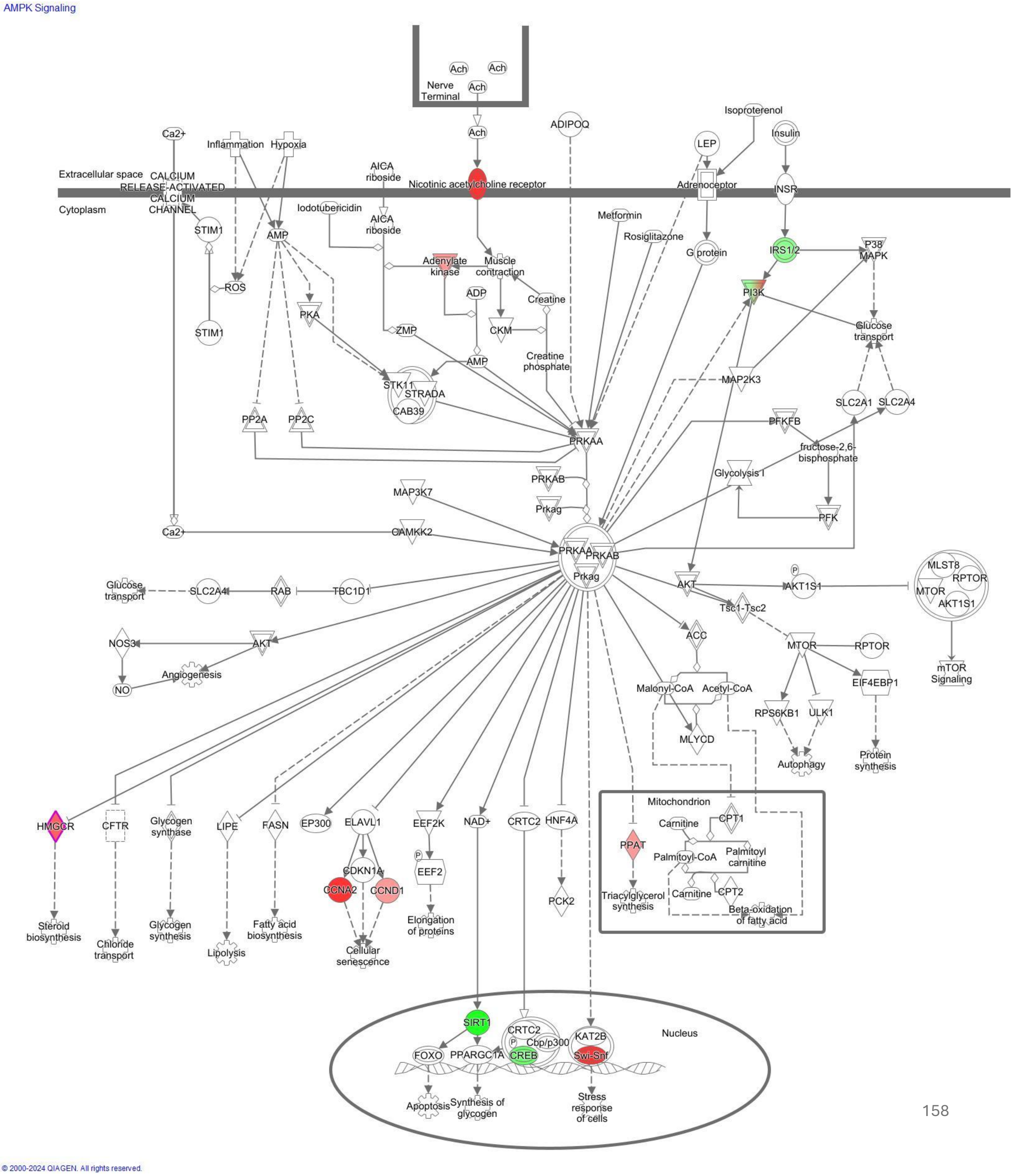

Figure S152

Superpathway of Cholesterol Biosynthesis

24 hours
CAGE analysis

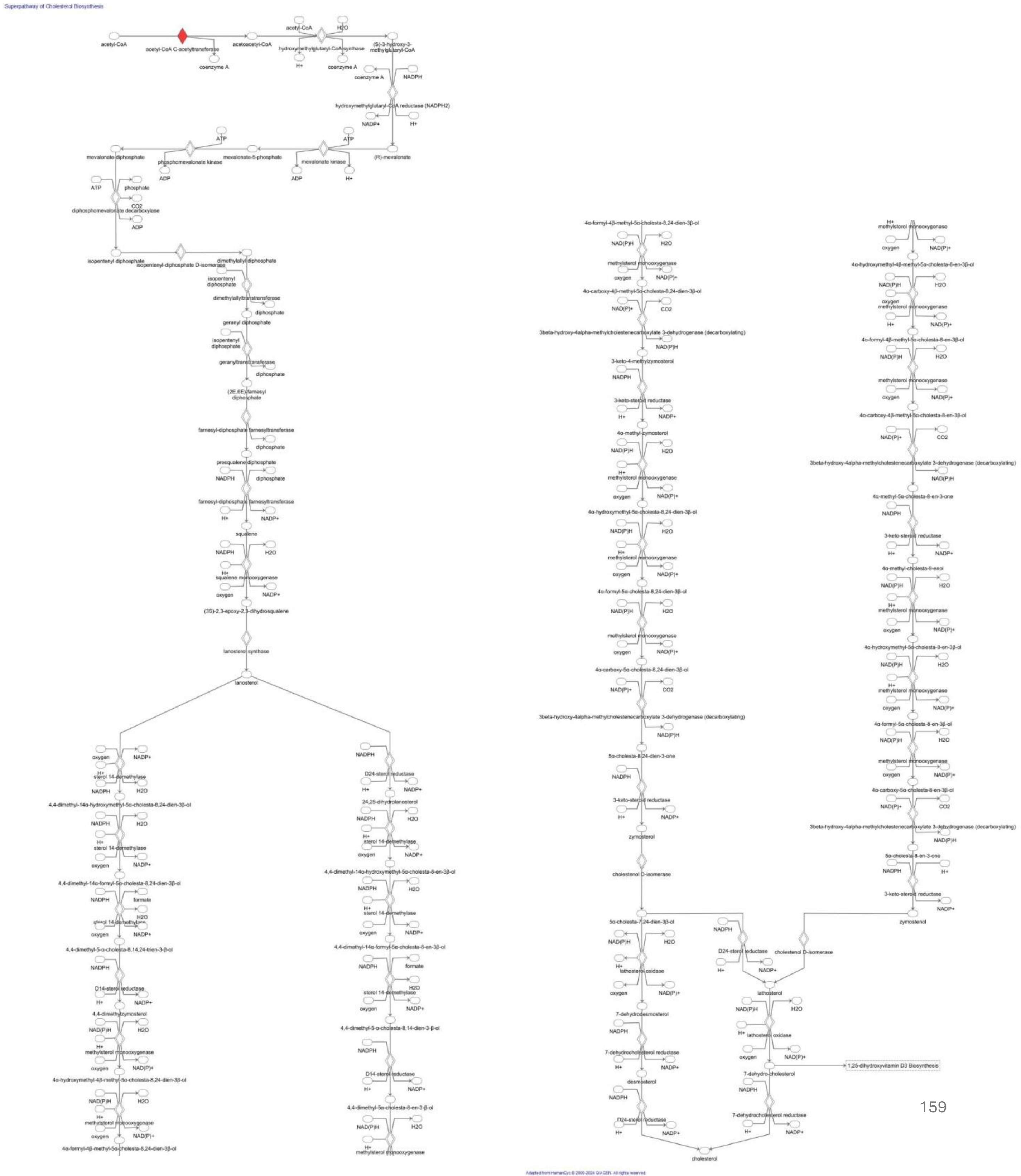

Figure S153

Superpathway of Cholesterol Biosynthesis

8 days
CAGE analysis

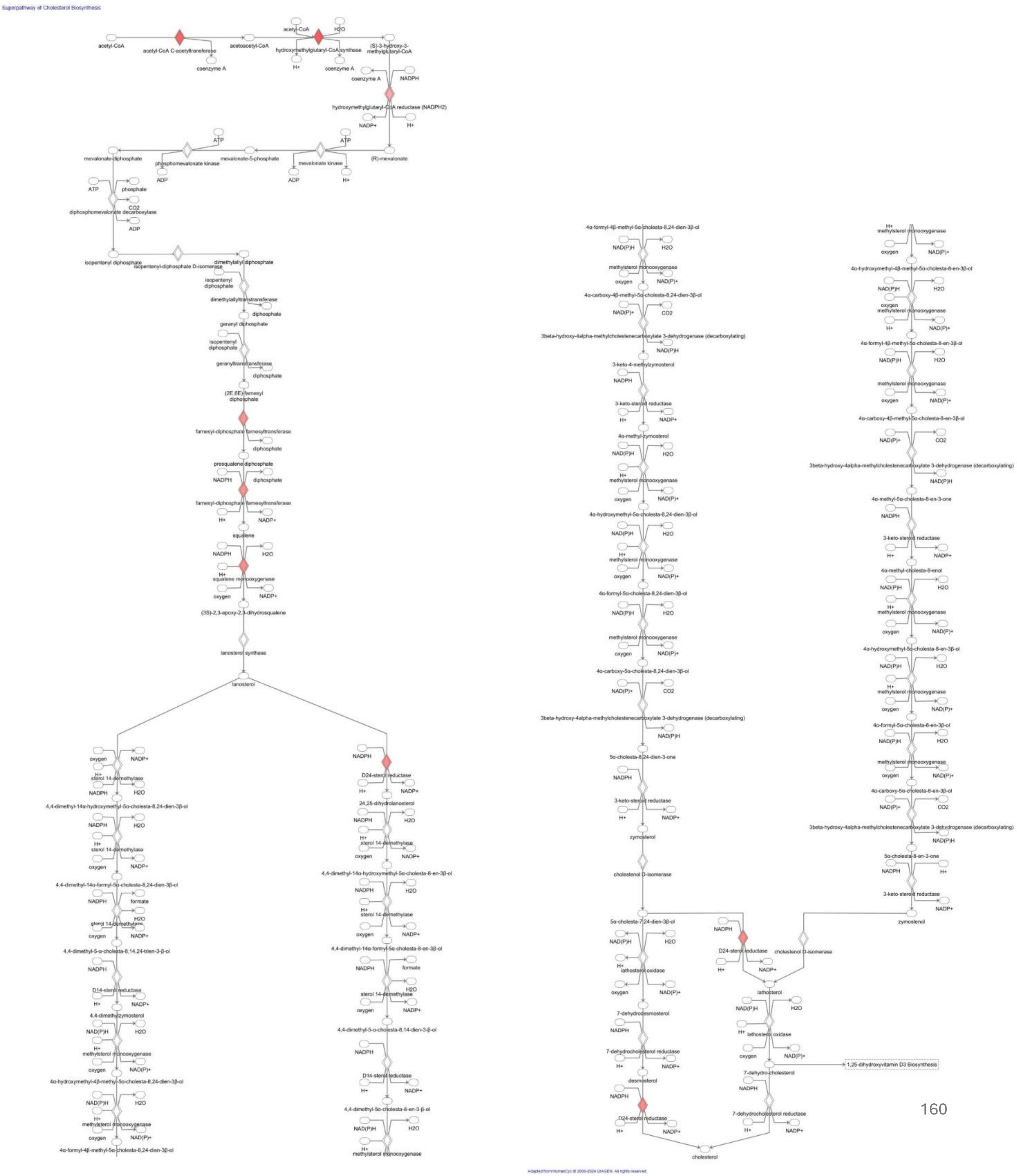

Figure S154

Superpathway of Cholesterol Biosynthesis

Day 21
CAGE analysis

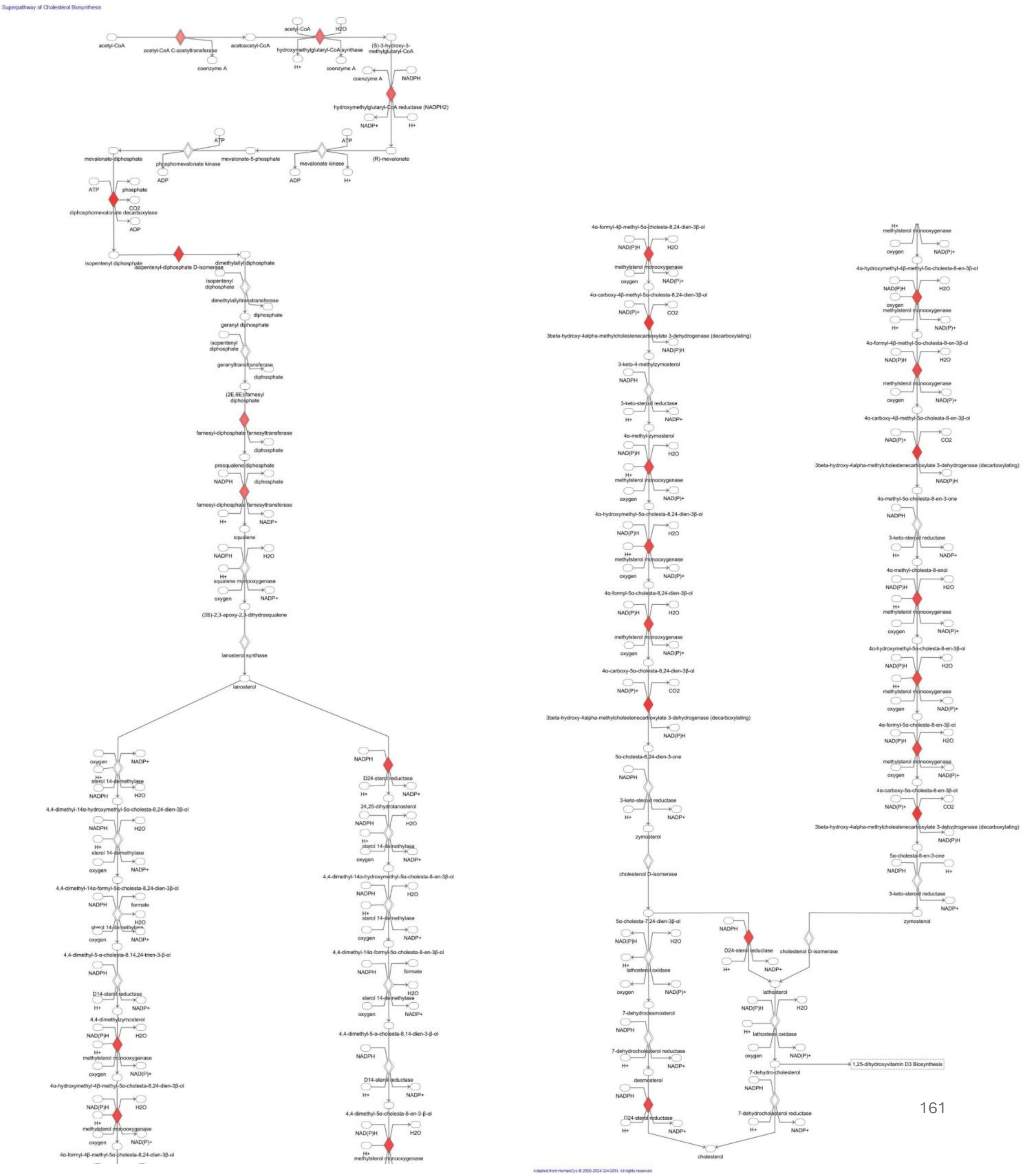

Figure S155

Folate signaling pathway

24 hours
CAGE analysis

Folate Signaling Pathway

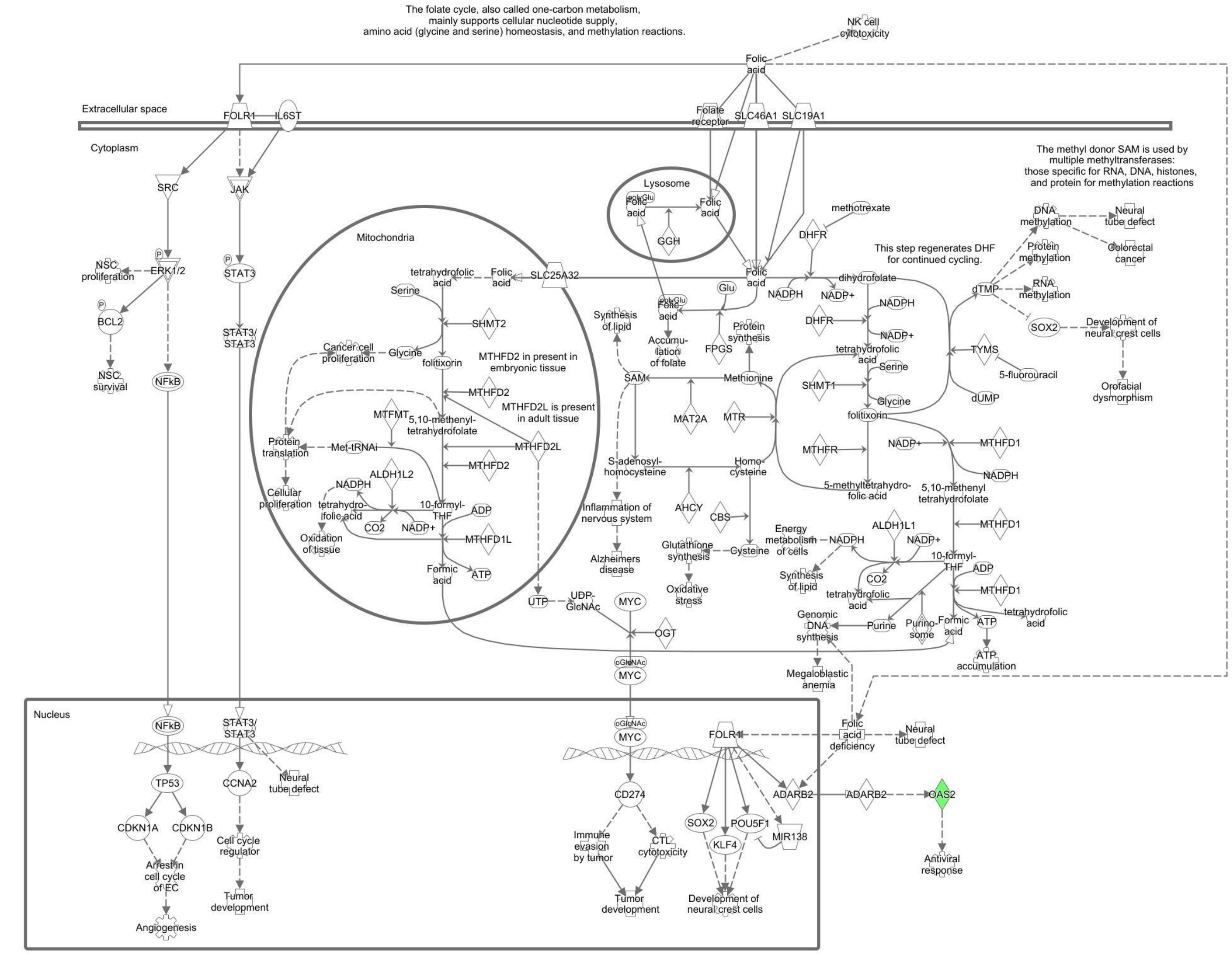

© 2000-2024 QIAGEN. All rights reserved.

Figure S156

Folate signaling pathway

8 days
CAGE analysis

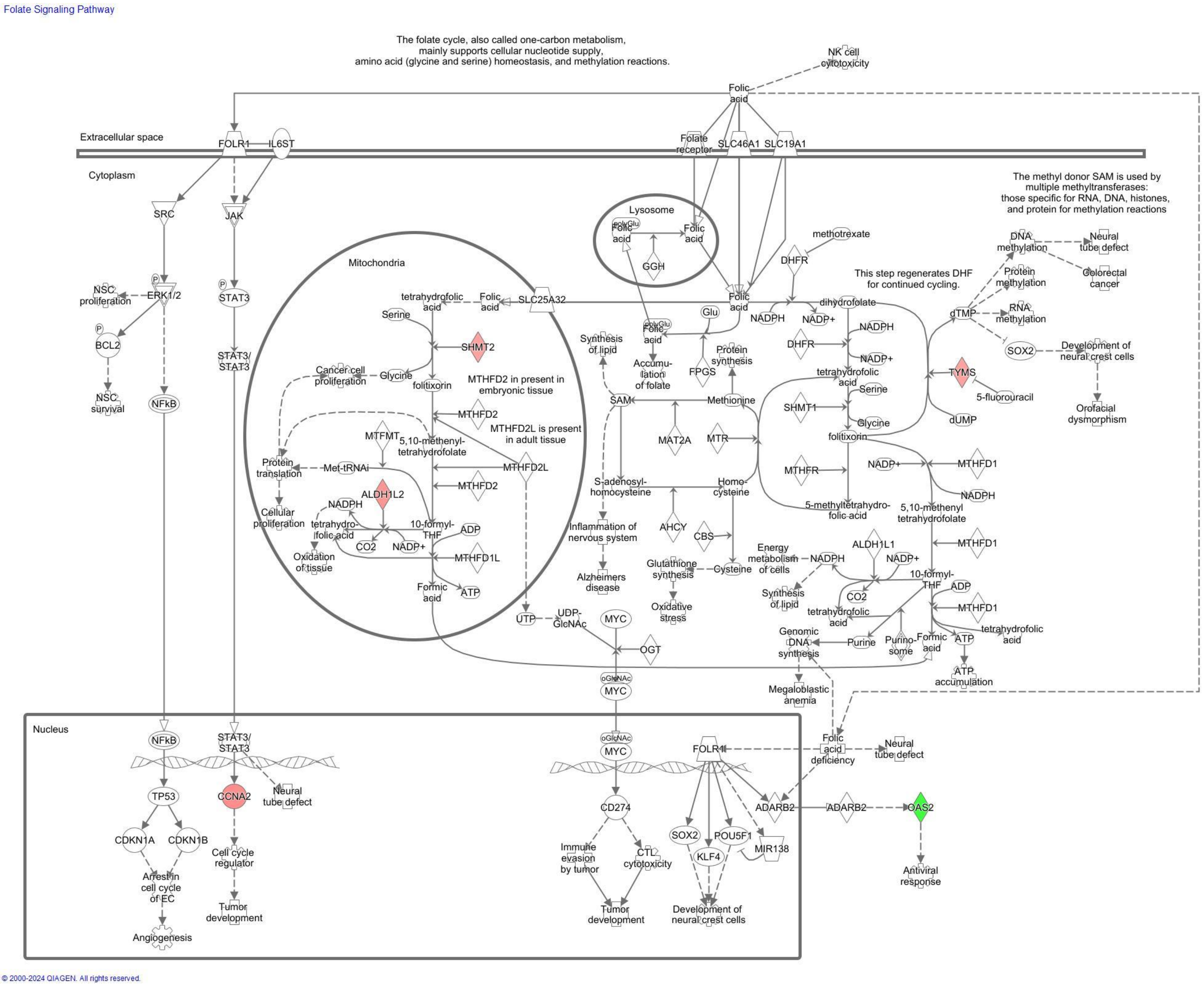

Figure S157

Folate signaling pathway

Day 21
CAGE analysis

Folate Signaling Pathway

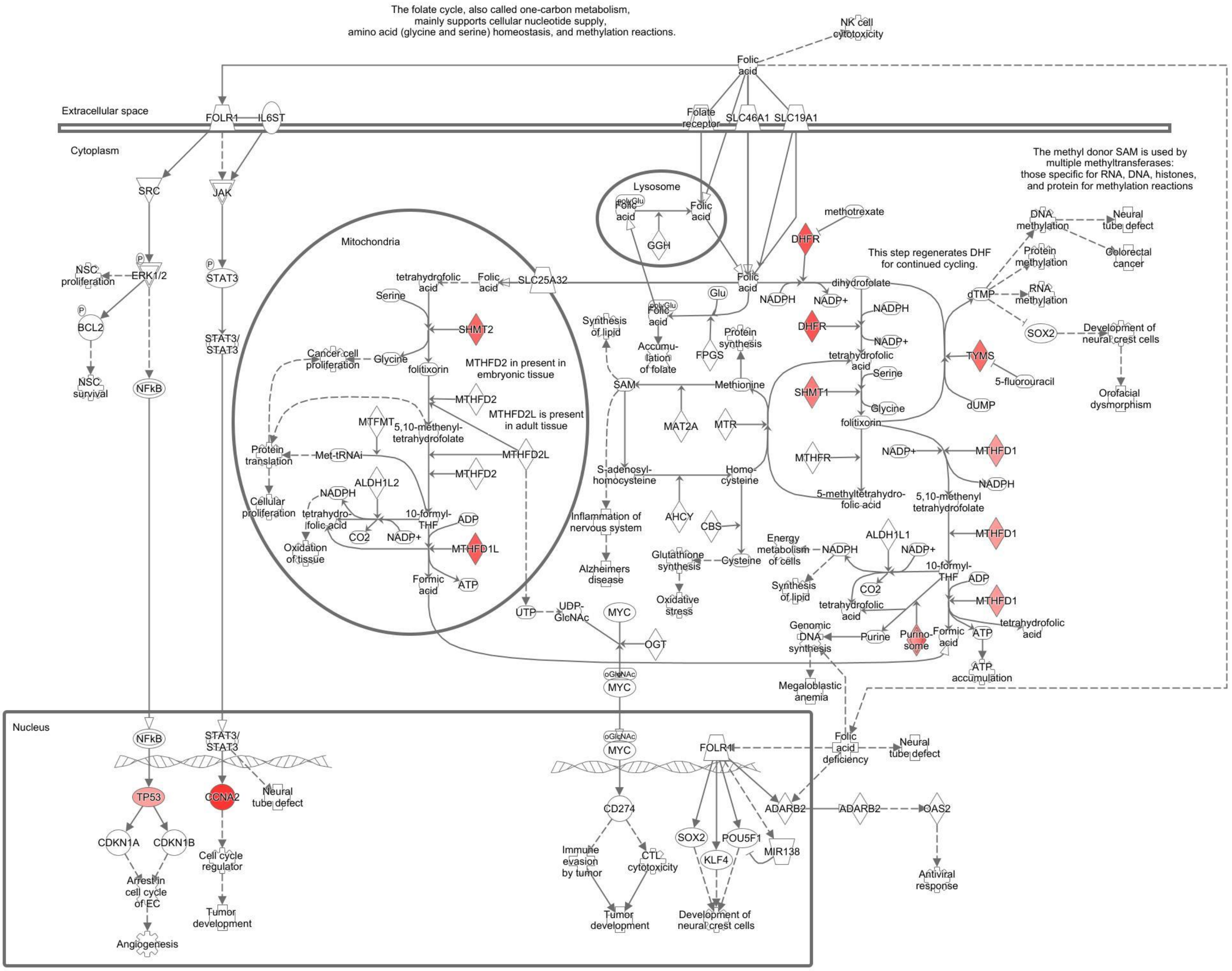

© 2000-2024 QIAGEN. All rights reserved.
